# Novel Molecular Synapomorphies Demarcate Different Main Groups/Subgroups of *Plasmodium* and Piroplasmida Species Clarifying Their Evolutionary Relationships

**DOI:** 10.3390/genes10070490

**Published:** 2019-06-28

**Authors:** Rahul Sharma, Radhey S. Gupta

**Affiliations:** Department of Biochemistry and Biomedical Sciences, McMaster University, Hamilton, ON L8N 3Z5, Canada

**Keywords:** genome sequences, molecular markers (synapomorphies), phylogenetic trees, conserved signature indels, Hematozoa, Piroplasmida, evolutionary relationships among *Plasmodium* subgenera *Haemamoeba*, *Laverania*, *Vinckeia* and *Plasmodium*

## Abstract

The class Hematozoa encompasses several clinically important genera, including *Plasmodium*, whose members cause the major life-threating disease malaria. Hence, a good understanding of the interrelationships of organisms from this class and reliable means for distinguishing them are of much importance. This study reports comprehensive phylogenetic and comparative analyses on protein sequences on the genomes of 28 hematozoa species to understand their interrelationships. In addition to phylogenetic trees based on two large datasets of protein sequences, detailed comparative analyses were carried out on the genomes of hematozoa species to identify novel molecular synapomorphies consisting of conserved signature indels (CSIs) in protein sequences. These studies have identified 79 CSIs that are exclusively present in specific groups of Hematozoa*/Plasmodium* species, also supported by phylogenetic analysis, providing reliable means for the identification of these species groups and understanding their interrelationships. Of these CSIs, six CSIs are specifically shared by all hematozoa species, two CSIs serve to distinguish members of the order Piroplasmida, five CSIs are uniquely found in all Piroplasmida species except *B. microti* and two CSIs are specific for the genus *Theileria*. Additionally, we also describe 23 CSIs that are exclusively present in all genome-sequenced *Plasmodium* species and two, nine, ten and eight CSIs which are specific for members of the *Plasmodium* subgenera *Haemamoeba, Laverania*, *Vinckeia* and *Plasmodium* (excluding *P. ovale* and *P. malariae*), respectively. Additionally, our work has identified several CSIs that support species relationships which are not evident from phylogenetic analysis. Of these CSIs, one CSI supports the ancestral nature of the avian-*Plasmodium* species in comparison to the mammalian-infecting groups of *Plasmodium* species, four CSIs strongly support a specific relationship of species between the subgenera *Plasmodium* and *Vinckeia* and three CSIs each that reliably group *P. malariae* with members of the subgenus *Plasmodium* and *P. ovale* within the subgenus *Vinckeia,* respectively. These results provide a reliable framework for understanding the evolutionary relationships among the *Plasmodium/*Piroplasmida species. Further, in view of the exclusivity of the described molecular markers for the indicated groups of hematozoa species, particularly large numbers of unique characteristics that are specific for all *Plasmodium* species, they provide important molecular tools for biochemical/genetic studies and for developing novel diagnostics and therapeutics for these organisms.

## 1. Introduction

The genus *Plasmodium* is comprised of eukaryotic unicellular parasites that parasitize a large variety of vertebrates [1,2,3]. Significantly, its members *P. falciparum*, *P. vivax, P. knowlesi, P. ovale* and *P. malariae*, are the causative agents of the major life-threating disease malaria [1,3]. The *Babesia* and *Theileria* genera, which are closely related to *Plasmodium,* are also of much interest and contain species that are clinically significant [4,5,6]. For example, *Babesia microti* is the primary causative agent of babesiosis which is a disease that manifests malaria-like symptoms in humans [6], whereas *Theileria* species are responsible for the disease theileriosis affecting domestic cattle causing significant economic losses within the farming industry [7]. The genera *Babesia* and *Theileria* form the majority of the named members within the order Piroplasmida [8,9,10] and this order along with the genus *Plasmodium* constitute the majority of species within the class Hematozoa (synonym Aconoidasida) in the phylum Apicomplexa [2,11,12]. In view of the enormous clinical significance and economic impact of the members of the class Hematozoa [12,13,14], it is of much importance to reliably understand the interrelationships of different parasitic organisms that are part of this class and develop reliable means for distinguishing them from each other.

The classification of species comprising the class Hematozoa was initially based on morphological characteristics such as the cellular ultrastructure, life-cycle details, and host range [2,11,15]. However, as most of these characteristics exhibit homoplasy, inferences based on them are considered unreliable [16,17,18]. In recent years, molecular sequenced data has been increasingly employed to understand hematozoa phylogeny [3,8,10,12,16,19,20,21,22]. In addition to the phylogenetic trees based on 18S rRNA sequences, *cytb* gene sequences have been widely used for phylogenetic analysis and description of novel members of the class Hematozoa [23,24,25,26]. Contemporary work has expanded on this by using multigene phylogenetic trees for the study of *Plasmodium* [12,16,19,27,28]. This includes one of the most intensive analyses done on *Plasmodium*, utilizing ~1000 genes shared amongst 12 *Plasmodium* species [29]. Similar approaches have also been applied for the study of Piroplasmida and also to a lesser extent on the class Hematozoa [8,10,30,31,32]. The results from these studies indicate that Piroplasmida and *Plasmodium* species are closely related and form two separate monophyletic clades. The two main genera of Piroplasmida *(Babesia* and *Theileria*) generally also form independent clades, except that *B. microti*, the primary agent of human babesiosis, branches distantly from other *Babesia* species [5,10,33]. These studies have also provided important insights into the interrelationships among the *Plasmodium* species. In general, the *Plasmodium* species parasitizing mammals versus those that parasitize other vertebrates (avian and lizard species) form two separate clades [16]. The latter group of species are placed into the subgenus *Haemamoeba* [34]. Within the mammalian-infecting species, 3 subgenera level groupings have been proposed [2,34,35,36]. Of these three subgroups, (i) subgenus *Laverania* contains *P. falciparum* and other great ape infecting species, (ii) subgenus *Plasmodium* includes *P. vivax* and other “Old World Monkey” infecting species, and (iii) subgenus *Vinckeia* is comprised of *P. berghei* and other non-primate infecting (rodent) species [36]. However, some human-infecting *Plasmodium* species e.g., *P. ovale* and *P. malariae,* do not consistently group with members of the subgenus *Plasmodium* and their phylogenetic placement remains uncertain [12,22,27,28,29,37]. Although recent phylogenetic studies have considerably advanced our understanding of the *Plasmodium* species, important questions remains concerning the interrelationships among different subgenera/groups within this genus [3,37,38,39]. Additionally, the genus *Plasmodium* and different subgroups within it are currently identified primarily on the basis of their branching in phylogenetic trees and the host specificity of the species, and no reliable molecular characteristics are known that are specific for these groups [3].

Genome sequences are currently available for large numbers of hematozoa species including 20 annotated genomes for *Plasmodium* species and 8 annotated genomes from the *Babesia* and *Theileria* genera. The available genomes provide a valuable resource for examining the evolutionary relationships amongst these species by construction of phylogenetic trees based on different datasets of genes/proteins sequences. More importantly, these genomes provide an extensive resource for comparative genomic studies for identifying novel molecular characteristics that are uniquely shared by members of the genera *Plasmodium, Babesia* and *Theileria* and could provide useful means for the demarcation of these taxa and for understanding their intra- and inter-relationships. One important class of molecular markers whose discovery has been facilitated by genome sequence analyses is comprised of conserved signature insertions/deletions (indels) (CSIs) in gene/protein sequences that are uniquely shared by an evolutionarily related group of species [40,41]. The CSIs that are useful for evolutionary studies are generally of definite lengths, present at specific positions in particular genes/proteins, and they are flanked on both sides by conserved regions to ensure that they constitute reliable characteristics [42,43,44,45]). The CSIs in genes/proteins sequences generally result from rare genetic changes and the most parsimonious explanation to account for their shared presence in a given gene or protein from a specific group of species is that the genetic change giving rise to the CSI first occurred in a common ancestor of the indicated group and then it was vertically inherited by the other group members [40,44,45,46,47]. Due to the discrete natures of the CSIs (even a one aa insertion or deletion in a protein sequence results from an in-frame three base pair insertion or deletion) and their location within conserved regions, their presence or absence in different lineages or proteins is generally not affected by factors such as differences in evolutionary rates among different species, or proteins, and long-branch attraction artefacts [40,41,45,48,49,50]. In view of these characteristics, the CSIs in gene/protein sequences have provided important means for demarcation of different groups of organisms in molecular terms [44,50]. Additionally, based upon the presence or absence of a CSI in outgroup species, it is possible to infer whether a given CSI represents an insert or a deletion in the protein sequence and thus infer the ancestral state of the protein. As a result, the CSIs in protein sequences can be used to infer rooted evolutionary relationships among a given group of species independently of the phylogenetic trees and they have proven very useful in clarifying a number of important relationships [40,41,42,43]. Although the shared presence of CSIs in protein sequences in some cases can result from homoplasy or lateral gene transfers [44,47,48], in general, when a conserved indel of a definite length is found uniquely in a phylogenetically related group of organisms, its most parsimonious explanation is inheritance from the most recent common ancestor [40,44,51]. Thus, the monophyletic group specific CSIs provide powerful means to support or refute a given phylogenetic hypothesis.

In the present study, we have used the available genome sequences to construct robust phylogenetic trees for hematozoa species based on two large datasets of concatenated protein sequences. In both trees, the *Plasmodium* species formed a strongly supported cluster that was separated from members of the order Piroplasmida by a long branch. Within the genus *Plasmodium*, monophyletic clades generally corresponding to the subgenera *Laverania*, *Plasmodium*, *Vinckeia* and *Haemamoeba* were also reliably observed. The trees also provide important information concerning the evolutionary relationships amongst the different *Plasmodium* species. Additionally, and more importantly, our comparative genomic analysis of the hematozoa genomes has identified 79 CSIs in different proteins that are uniquely shared by different members of this class. Of the identified molecular markers, 16 CSIs are specific for the class Hematozoa and either all or specific members of the order Piroplasmida. The remaining 63 CSIs are distinctive characteristics of either all 20 genome-sequenced *Plasmodium* species or specific subgroups/subgenera with the genus *Plasmodium* providing reliable molecular means for identification of these groups and clarifying their interrelationships. The described molecular markers in addition to their usefulness for evolutionary and taxonomic studies, due to their exclusivity for these clinically important groups of organisms, also provide potentially useful means for development of novel diagnostics and therapeutics that are specific for these organisms.

## 2. Materials and Methods

### 2.1. Construction of Phylogenetic Trees

Phylogenetic trees were constructed for 28 genome sequenced members of the class Hematozoa and eight species from the order Eucoccidiorida, which were used for rooting of the trees. Some characteristics of the genome sequences that were used for tree construction are listed in Table S1. The phylogenetic trees were constructed based on two separate datasets of concatenated protein sequences, which are conserved in different hematozoa as well as the outgroup species. The first tree utilized 14 proteins which are involved in transcription and translation related functions, whereas the second tree was based on 10 metabolism-related proteins. These proteins were selected based on the criteria that they were within the majority of the members within the group of interest and that only a single homolog (or gene) of these proteins was detected in different hematozoa species. These selection criteria ensured that only orthologous sequences were used for the construction of the phylogenetic trees. Information for the proteins that were used for tree construction is provided in Tables S2 and S3, respectively. The phylogenetic tree construction was carried out using an internally developed pipeline that we have described in earlier work [46,52]. Briefly, the Clustal Omega algorithm was used to generate multiple sequence alignments for all proteins in a given dataset which are present in at least 80% of the input genomes. The aligned sequences were trimmed with TrimAl [53] to remove poorly aligned regions before they were concatenated into separate files. The final concatenated alignments for the transcription-translation related proteins and the metabolism-related datasets of protein sequences consisted of 7700 and 4068 aligned amino acid residues, respectively. Maximum-likelihood trees based on these alignments were constructed using FastTree 2 [54] and optimized using RAxML 8 [55] as described in earlier work [46].

### 2.2. Identification of Conserved Signature Indels (CSIs)

The identification of CSIs was carried out as described in earlier work [40,46,47]. In brief, BLASTp searches were carried out on all protein sequences from *P. falciparum* that were >100 amino acids in length, against the NCBI non-redundant database. Based on these searches, for those proteins for which multiple hits were observed with E value <1 × e^−15^ for large numbers of species, protein sequences of 10–15 representative hematozoa species and 8–10 homologs from other Apicomplexa and/or other eukaryotic organisms were retrieved. Multiple sequence alignments of different proteins were created using Clustal Omega [56]. The alignments were visually inspected for insertions or deletions of fixed lengths which were flanked on both sides by at least 4–5 conserved amino acids (aa) in the adjacent 40–50 aa and appeared to be exclusive to some or all hematozoa. Query sequences encompassing the indel and its flanking 50–100 aa were collected for all potential CSIs. Afterwards, the query sequences underwent another BLASTp search. The resulting top 500 hits for all queries were examined to determine the presence or absence of CSIs in the homologs from different species and thus the group specificities of the CSIs. Signature files for all CSIs were created using SIG_CREATE and SIG_STYLE programs described in our earlier work [47] that are available on the GLEANS (Gleans.net) server. The CSIs reported here, unless otherwise indicated, are specific for all members of the indicated groups including additional strains for different species (not shown in main Figures but information for them is included in the Supplementary Figures), whose homologs were detected by BLASTp searches.

### 2.3. Homology Modelling and Analysis of Protein Structures

Homology models of two proteins containing the CSIs were created to map the locations of the CSIs within the proteins’ structures. A homology model of the 40S ribosomal protein S3 from *P. berghei*, which contains a one aa insertion specific for all *Plasmodium* species was constructed based on the solved structure of the homologous protein from *Toxoplasma gondii* (PDB ID: 5XXU_D) [57]. Similarly, a homology model of the leucine aminopeptidase protein from *P. vivax* was generated using the available crystal structure from *P. falciparum* (PDB ID: 4ZX8_A) [58]. Homology modeling was carried out using the MODELLER v9.11 program [59] and their stereochemical properties were assessed as described in our earlier work [60].

## 3. Results

### 3.1. Phylogenetic Analysis of Hematozoa

The evolutionary relationships among the genome-sequenced hematozoa species was examined based on two large datasets of concatenated protein sequences each consisting of multiple conserved proteins. The first dataset is composed of 14 proteins which are transcription and translation related, whereas the second dataset contains 10 proteins involved in metabolism and other cellular functions. The second dataset also included the protein adenylosuccinate lyase, which has been previously used in a phylogenetic study on *Plasmodium* [16,61]. As the two protein datasets utilize proteins involved in different functions, results from them should provide independent assessment of the evolutionary relationships among the hematozoa species.

**Figure 1:**
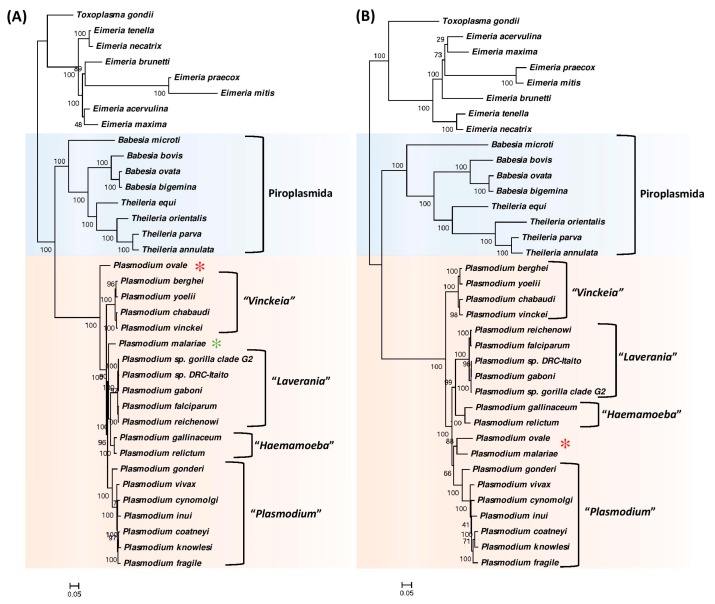
Maximum likelihood trees for the 28 genome sequenced members of the class Hematozoa (**A**) Tree based on concatenated sequence of 14 transcription and translation related proteins and (**B**) tree based on concatenated sequence of 10 conserved metabolism-related proteins. The trees were rooted using sequences from other Apicomplexa species. Numbers on the branches indicate bootstrap values for these nodes. Some of the known taxonomic groups are labelled and the *Plasmodium* species showing anomalous branching are marked with asterisks (*).

The phylogenetic trees based on the two protein datasets are shown in Figure 1A,B. Both trees demonstrate highly-supported and consistent interrelationships. In both trees, the Piroplasmida group of species are found to branch closest to the *Plasmodium* clade, which has also been observed previously [12,30,31]. Within the Piroplasmida cluster, species from the genera *Babesia* and *Theileria* also form distinct clades, except for the anomalous branching of *Babesia microti,* which branches deeply and separately from the other *Babesia* as well as *Theileria* species. These results are also consistent with earlier studies [5,8,10,12,33]. The *Plasmodium* species form a strongly supported monophyletic clade in both trees and this clade is separated from Piroplasmida by a long branch.

Although the species within the *Plasmodium* clade are tightly clustered, in both trees, at least four distinct clades of *Plasmodium* species are consistently observed. We have labelled these clades based on the names of the subgenera to which most of the species in these groups have been classified [2,19,23,27,29,34,35,36]. These clades include a clade of avian-infecting species labelled as the “*Haemamoeba*” group and three clades of mammalian-infecting species labelled as the *“Vinckeia”*, *“Laverania”* and the *“Plasmodium”* groupings. While all other *Plasmodium* species group within these four clades, the species *P. ovale* and *P. malariae* group separately and their branching positions also differ in the two constructed trees. Whereas in the tree based on transcription-translation related proteins (Figure 1A), *P. ovale* formed the earliest branching lineage within the genus *Plasmodium*, in the tree based on metabolism related proteins, *P. ovale* clustered with *P. malariae* and this clade branched in the vicinity of the “*Vinckeia*” subgenus. Although, presently both *P. ovale* and *P. malariae* are classified in the subgenus *Plasmodium*, their taxonomic affiliation or phylogenetic affinity has not been resolved by earlier studies [12,22,23,27,28,29,62]. Another observation of interest is that in both phylogenetic trees the “*Haemamoeba*” clade corresponding to the two avian-infecting species does not branch separately from the clades of mammalian-infecting *Plasmodium* species. Similar branching of the avian infecting *Plasmodium* species has also been seen in some earlier studies [12,38,39].

### 3.2. Identification of Molecular Markers Specific for Hematozoa and its Main Groups

Based on the phylogenetic trees shown in Figure 1, some inferences regarding the evolutionary relationships amongst the hematozoa species can be drawn. However due to the tight clustering of *Plasmodium* species in these trees and the short branches which separate the identified clades, it is important to confirm these inferences by other independent approaches. As noted in the introduction, conserved signature indels (CSIs) in protein sequences that are uniquely shared by a given group of organisms provide an important class of molecular markers that have been proven very useful for evolutionary/taxonomic studies [40,41,43]. Therefore, a major focus of the present work was to perform comprehensive genomic analysis on protein sequences from hematozoa species to identify CSIs that are specific for different groups of organisms comprising this class. Our analysis has identified a total of 79 CSIs that are specific for multiple clades and taxonomic groupings of Hematozoa. The characteristics of the identified CSIs and their significance concerning evolutionary relationships amongst the class Hematozoa and the genus *Plasmodium* are described below.

### 3.3. Molecular Markers Specific for the Class Hematozoa

**Figure 2:**
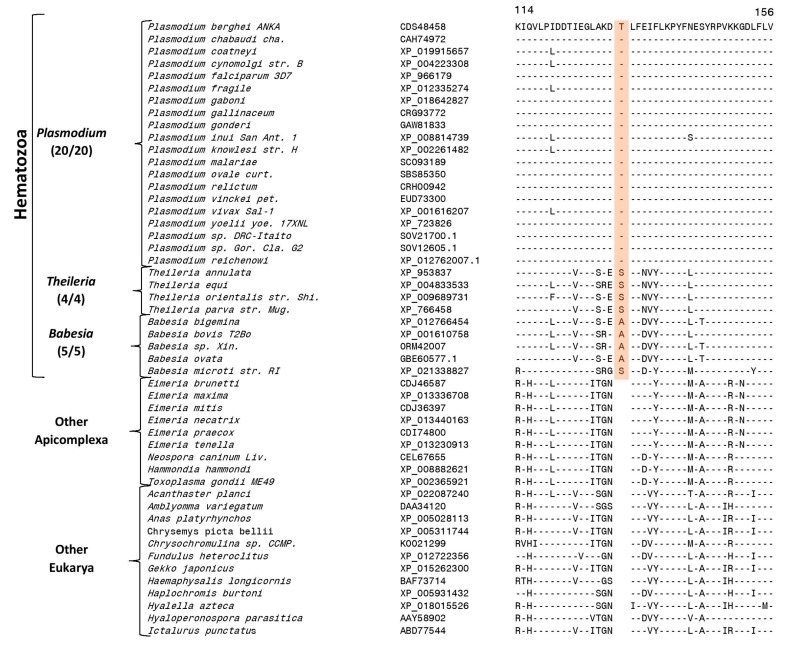
Partial sequence alignment of the cell division cycle protein Cdc48 showing a 1-aa insertion in a conserved region (boxed) which is exclusively found in all available homologs from members of the class Hematozoa. The dashes (-) in the alignment indicate identity with the amino acid residues shown in the top sequence. Accession numbers for each sequence are indicated in the second column. Information for five additional CSIs that are also specific for the class Hematozoa are shown in Figures S2–S6 and information for them is summarized in Table 1. The numbers with the group names in all figures indicate the presence/absence of the CSIs in different species which are shown in Figure 1. In cases, where sequences for additional strains from these groups were detected by BLASTp searches, they also contained the indicated CSIs (see Supplementary Figures).

Our work has identified six CSIs in six different proteins which are specific for the class Hematozoa. An example of one of these CSIs, consisting of a one aa insertion (highlighted) in the cell division cycle protein 48 (Cdc48), is presented in Figure 2. The one aa CSI in this protein is present in a conserved region and it is commonly shared by all homologs from the class Hematozoa for which sequence information is available, but it is not present in any other Apicomplexa species or the homologs of other eukaryotic organisms present in top BLASTp searches. The Cdc 48 protein is an ubiquitin-dependent molecular chaperone, which help mediate a variety of degradative and regulatory processes in order to maintain cellular homoeostasis [63]. Similar to this CSI, five other CSIs which are also specific for the class Hematozoa were identified the present work. These CSIs are found in the proteins 20S proteasome β 4 subunit, a putative 30S ribosomal protein S9, a putative 40S ribosomal protein S12, Golgi reassembly-stacking protein 1 and pyruvate kinase 2A. Some characteristics of these CSIs are summarized in Table 1 and detailed sequence information for them is provided in Figures S1–S6. Due to the specificities of these CSIs for the class Hematozoa, the genetic changes associated with these CSIs likely occurred in a common ancestor of this class.

### 3.4. Molecular Signatures Specific for the Order Piroplasmida

**Figure 3:**
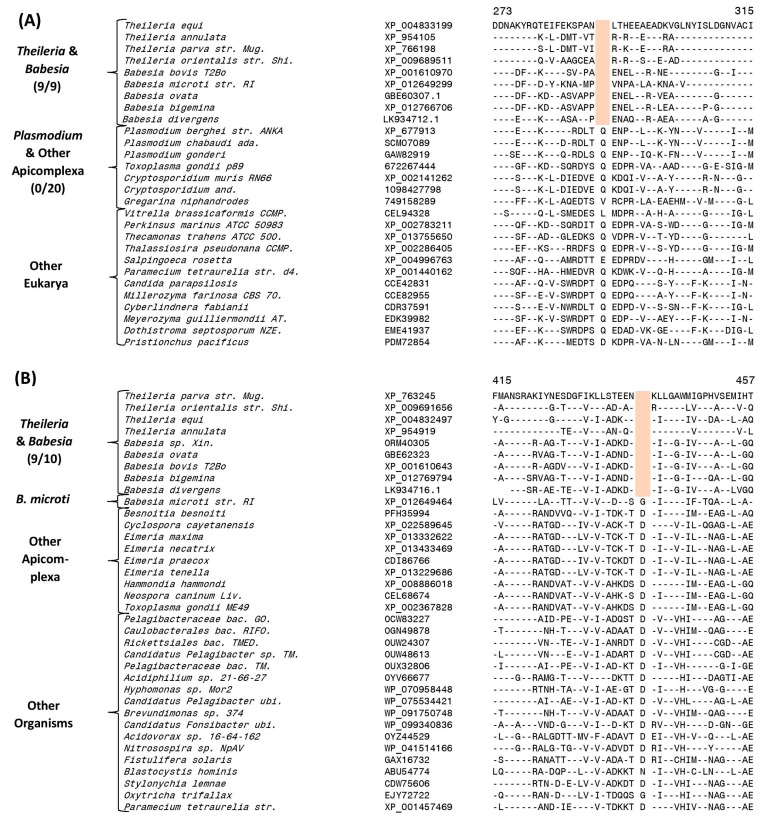
Sequence alignments showing conserved signature indels specific for members of the order Piroplasmida. (**A**) Partial sequence alignment of the protein succinyl-CoA synthetase β chain showing a 1-aa deletion that is uniquely shared by all available homologs from the order Piroplasmida. (**B**) Partial sequence alignment of the protein dihydrolipoamide dehydrogenase showing a 1-aa deletion in a conserved region that is present in all Piroplasmida homologs except *Babesia microti*. Sequence alignments for multiple other CSIs exhibiting similar specificities is provided in Supplementary Figures S7–S13 and their main characteristics are summarized in Table 1.

Our analysis has also identified two CSIs which are exclusively found in all available Piroplasmida homologs. An example of one of the CSIs specific for the order Piroplasmida is shown in Figure 3A. In this case, a one aa deletion in the protein succinyl-CoA synthetase β chain is uniquely present in the homologs of all Piroplasmida species for which sequence information is available, but this deletion is not found in the protein homologs from other Apicomplexa and Eukarya. Sequence information for this CSI and one other CSI showing similar specificity is provided in Figures S7 and S8 and their main characteristics are summarized in Table 1. The genetic changes leading to these CSIs have likely occurred in a common ancestor of the order Piroplasmida. We have also identified five other CSIs that are commonly shared by all Piroplasmida species except *B. microti*, which shows deeper branching in the trees in comparison to the other species from this order (Figure 1). One example of a CSI showing this pattern is shown in Figure 3B. In this case, a one aa deletion in a conserved region of the protein dihydrolipoamide dehydrogenase is commonly and uniquely shared by all other *Babesia* and *Theileria* species, except *B. microti*. More detailed sequence information for this CSI and the other four CSIs showing similar species distribution pattern is presented in Figures S9–S13 and some of their characteristics are summarized in Table 1. The genetic changes responsible for these CSIs are postulated to have occurred in a common ancestor of the other Piroplasmida, after the branching of *B. microti*.

Our analysis has also identified three CSIs which are exclusively found in all sequenced species of either *Theileria* or *Babesia* (excluding *B. microti*) genera. Sequence information for these CSIs is presented in Figures S14 and S15, their main characteristics are also summarized in Table 1. These CSIs serve to distinguish members of the genera *Babesia* or *Theileria* from other Apicomplexa and they also provide strong evidence that *B. microti* is genetically distinct from other members of the genus *Babesia* and makes a case for its placement into a separate genus.

### 3.5. Molecular Signatures Specific for the Genus Plasmodium and Its Subgenera

**Figure 4:**
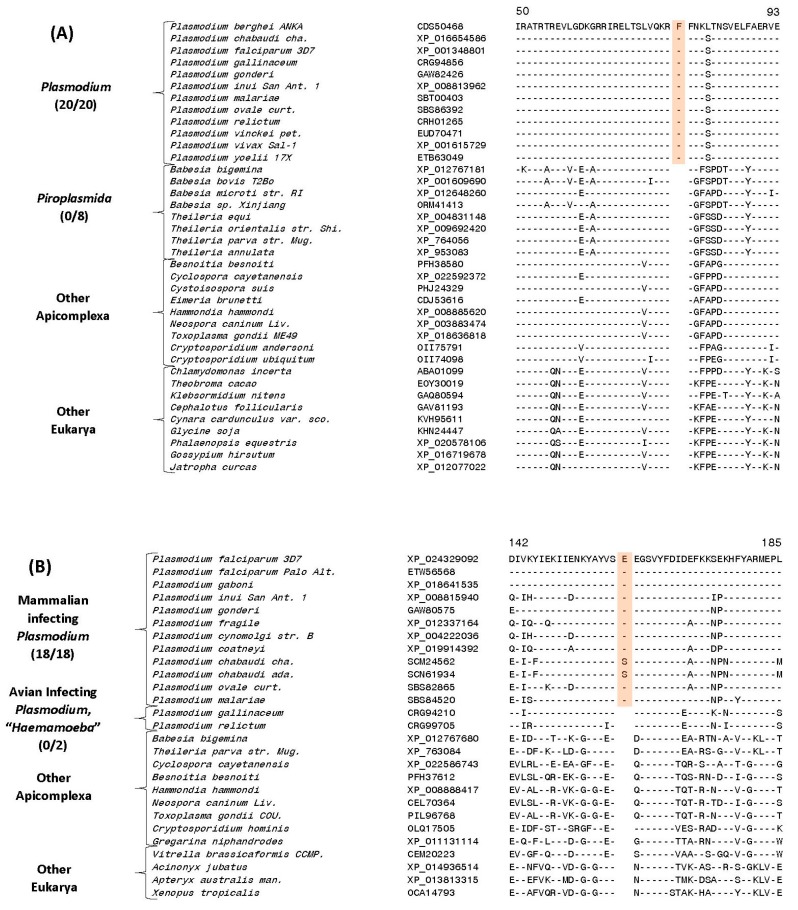
Sequence alignments of conserved signature indels specific for the genus *Plasmodium* and one that is exclusively found in the mammalian-infecting species. (**A**) Partial sequence alignment of the 40S ribosomal protein S3 showing a 1-aa insertion in a conserved region that is uniquely found in all available *Plasmodium* homologs. Sequence information for 22 other CSI in other proteins that are also specific for the genus *Plasmodium* is shown in Figures S17–S38 and their summary is provided in Table 2. (**B**) Partial sequence alignment of the protein cysteine-tRNA ligase showing a 1-aa insertion that is uniquely shared by all other *Plasmodium* homologs, but lacking in the avian-infecting species (subgenus *Haemamoeba*) as well as other eukaryotic organisms.

The present work has also identified large numbers of CSIs which are specifically shared by either all or specific groups of *Plasmodium* species providing novel means for their identification and understanding their interrelationships. Of these CSIs, 23 CSIs present in different proteins are exclusively shared by the homologs of all genome sequenced *Plasmodium* species, but not found in any other Apicomplexa or metazoan species. Figure 4A shows one example of a CSI, consisting of a one aa insertion in a highly conserved region of the 40S ribosomal protein S3, which is specifically shared by all available *Plasmodium* homologs, but not found in any other eukaryotic organism.

Sequence information for the 22 other CSIs present in other important proteins that are also distinctive characteristics of the genus *Plasmodium* is presented in Supplementary Figures S16–S38 and some of their characteristics are summarized in Table 2. Due to the exclusive presence of these CSIs in the homologs from all sequenced *Plasmodium* species, the genetic changes leading to these CSIs have likely occurred in a common ancestor of the genus *Plasmodium*, providing important molecular characteristics distinguishing this group of organisms from all others.

Another interesting CSI identified by our analysis found in a highly conserved region of the protein cysteine-tRNA ligase is uniquely shared by all mammalian-infecting *Plasmodium* species, but it is not found in the members of the subgenus “*Haemamoeba*” consisting of the two avian infecting species viz. *P. gallinaceum* and *P. relictum* (Figure 4B) (a more detailed alignment can be found in Figure S39).

The absence of this CSI in all other Apicomplexa as well as eukaryotic organisms indicates that this CSI represents an insert and the genetic change responsible for this CSI occurred in a common ancestor of the mammalian-infecting *Plasmodium* species after this group of organisms diverged from the avian-infecting *Plasmodium* species. Thus, this CSI supports the inference that the avian-infecting species are ancestral to the mammalian-infecting *Plasmodium* species [39,64].

We have also identified multiple CSIs that are specific for the four main groupings or subgenera of *Plasmodium* species. Two of these CSIs are specific for the subgenus “*Haemamoeba*” comprised of *P. relictum* and *P. gallinaceum* species.

**Figure 5:**
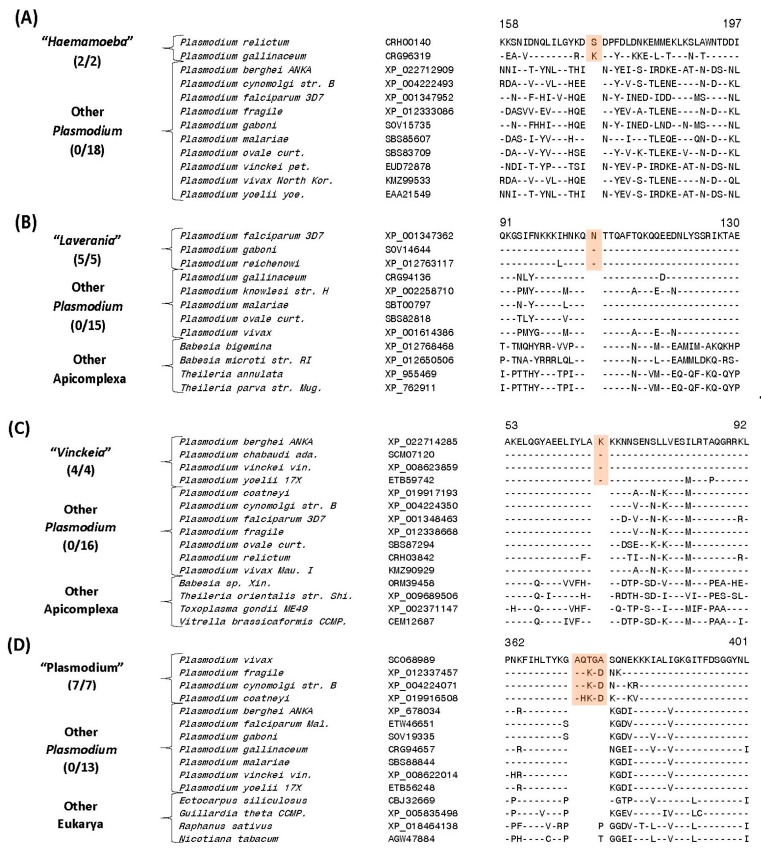
Examples of CSIs that are specific for different groups/subgenera of *Plasmodium* species (**A**) Partial sequence alignment of a protein phosphatase showing a 1-aa insertion that is specific for the subgenus *Haemamoeba*. (**B**) Partial sequence alignment of the eukaryotic translation initiation factor 3 (subunit D) showing a 1-aa insertion that is specific for members of the subgenus *Laverania*. (**C**) Partial sequence alignment of the mitochondrial ribosomal protein L17-2 showing a 1-aa insertion that is specific for the subgenus *Vinckeia*. (**D**) Partial sequence alignment of the protein leucine aminopeptidase containing a 5-aa insertion exclusively found in members of the subgenus *Plasmodium* except *P. ovale* and *P. malariae*. Sequence alignments for multiple other CSIs that are also specific for these subgenera are provided in Figures S41, S60–S66 and information for them is summarized in Table 3.

Sequence information for one of these CSIs consisting of a one aa insert in a protein phosphatase that is exclusively found in these two avian-infecting *Plasmodium* species, but not in any other *Plasmodium* or Apicomplexa species is presented in Figure 5A. Sequence information for the other CSI specific for the “*Haemamoeba*” clade is provided in Supplementary Figure S41.

Nine other CSIs identified in this work are exclusively shared by members of the subgenus *“Laverania”*. This group is made up of the following genome sequenced species: *P. falciparum, P. reichenowi, P. gaboni* and two unnamed species *P. sp. gorilla clade G2* and *P. sp. DRC-Italio*. One example of a CSI consisting of a one aa insertion present in the eukaryotic translation initiation factor 3 subunit D, which is specific for this group is shown in Figure 5B. More detailed sequence information for this CSI as well as the remaining eight CSIs that are specific for the “*Laverania”* clade is provided in Figures S42–S50 and some characteristics of these CSIs are summarized in Table 3. It is of interest to note that for one of the CSIs specific for this group, found in the protein pre-mRNA processing splicing factor 8 (Figure S50), is uniquely shared by all other members of this group except *P. gaboni*, *P. sp. gorilla clade G2* and *P. sp. DRC-Italio*. This CSI suggests that within the *“Laverania”* group, *P. gaboni P. sp. gorilla clade G2* and *P. sp. DRC-Italio* possibly constitutes an earlier diverging clade in comparison to the other group members.

Ten CSIs identified in this work are exclusively shared by members of the subgroup *“Vinckeia”* comprising of the following four genome-sequenced species *P. berghei, P. chabaudi, P. vinckei* and *P. yoelii*. One example of a CSIs specific for this clade is provided in Figure 5C. In this case, in a highly conserved region of the mitochondrial ribosomal protein L17-2, a one aa insert is specifically present in all members of *“Vinckeia”* group of species, but not in any other *Plasmodium* or Apicomplexa species. More detailed sequence information for this CSI and 9 other CSIs that are also specific for the *“Vinckeia”* clade is provided in Figures S51–S60 and their main characteristics are summarized in Table 3. Similarly, we have also identified eight CSIs that are specific for the *“Plasmodium”* subclade consisting of the following genome sequenced species *P. vivax, P. gonderi, P. fragile, P. inui, P. knowlesi, P. coatneyi* and *P. cynomolgi*. One example of a CSI consisting of a 5 aa insertion found in the protein leucine aminopeptidase, which is specific for this group is shown in Figure 5D. More detailed sequence information for this CSI as well as seven other CSIs which are also specifically found in all members of the *“Plasmodium”* clade is presented in Figures S41 and S60–S66 and information for these CSIs is summarized in Table 3.

### 3.6. Molecular Signatures for the Mammalian-Infecting Plasmodium Species Clarifying their Evolutionary Relationships

**Figure 6:**
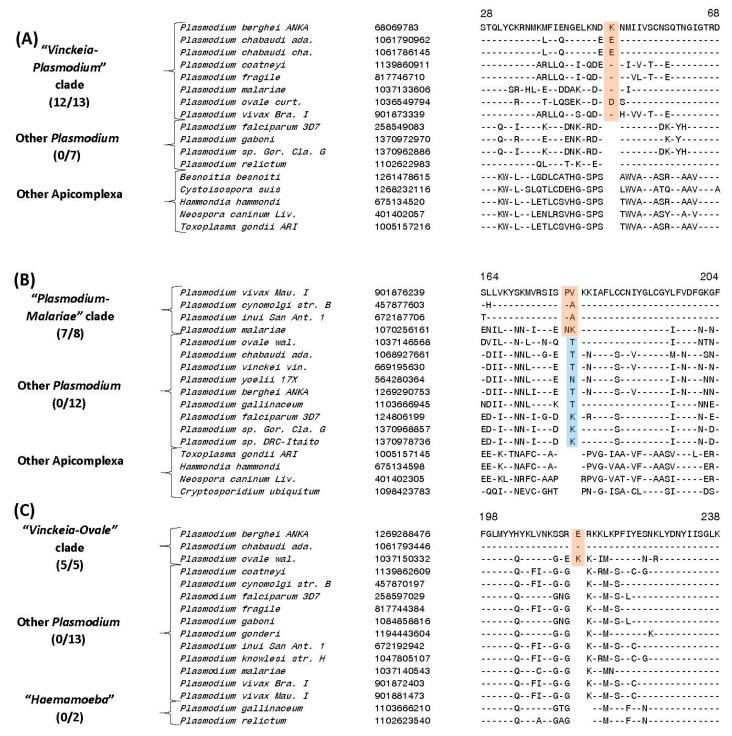
Examples of CSIs specific for the mammalian *Plasmodium* groups clarifying the genetic association of *P. ovale* and *P. malariae* species (**A**) Partial sequence alignment of the protein biotin-acetyl-CoA-carboxylase ligase showing a 1-aa insertion that is exclusively shared by all species from the subgenera *Plasmodium* and *Vinckeia*. (**B**) Partial sequence alignment of the ubiquitin-activating enzyme E1 protein showing a 2-aa insertion that is uniquely shared by different members of the subgenus *Plasmodium* but lacking in *P. ovale*. *P. knowlesi* contains a three aa insert in this position (not shown). (**C**) Partial sequence alignment of the phosphoinositide-specific phospholipase C protein showing a 1-aa insertion which is exclusively shared by members of the subgenus *Vinckeia* and *P. ovale*. Sequence information for multiple other CSIs exhibiting similar specificity is presented in Figures S67–S75 and their main characteristics are summarized in Table 3.

In addition to the CSIs that are specific for the genus *Plasmodium* and its four main subgenera, our analysis has also identified several CSIs that are helpful in clarifying the evolutionary relationships among the mammalian-infecting *Plasmodium* species, including *P. malariae* and *P. ovale*, whose association with the other subgenera is uncertain [23,27,28,29,62]. Currently both these species are taxonomically classified within the subgenus *Plasmodium* [17]. Of these other CSIs, four CSIs identified by our analysis are specifically shared by all *Plasmodium* species except those from “*Haemamoeba”* and “*Laverania”* clades. One example of a CSI exhibiting this kind of specificity is presented in Figure 6A. In this case, in the sequence alignment of the protein biotin-acetyl-CoA-carboxylase ligase protein, in a conserved region, a one aa insertion is commonly and exclusively shared by all available homologs from the “*Plasmodium”* and “*Vinckeia”* clades as well as *P. malariae* and *P. ovale*, but this CSI is absent in all other *Plasmodium* or Apicomplexa species. We refer to the group demarcated by these CSIs as the “Vinckeia-Plasmodium” clade. More detailed sequence information for the CSI shown in Figure 6A and three other CSIs which also exhibit similar specificities is provided in Figures S67–S70 and some of their characteristics are summarized in Table 3. The specific presence of these CSIs in members of the subgenera *Plasmodium* and *Vinckeia* as well as *P. malariae* and *P. ovale*, strongly suggests that these species shared a common ancestor exclusive of the other *Plasmodium* species, and the genetic changes responsible for these CSIs occurred in a common ancestor of this group after its divergence from other *Plasmodium* species.

The present study has also identified three CSIs that are exclusively shared by members of the “*Plasmodium”* clade and *P. malariae*, but not in any other *Plasmodium* or Apicomplexa species (the clade demarcated by these CSIs will be referred to here as the “Plasmodium-Malariae” clade). An example of a CSI consisting of a two aa insertion in the protein ubiquitin-activating enzyme E1, which is specifically shared by the above groups of species is shown in Figure 6B. Detailed sequence information for this CSI as well as two other CSIs exhibiting similar specificities is provided in Figures S70–S72 and some of their characteristics are summarized in Table 3. The unique shared presence of these CSIs in members of the subgenus *Plasmodium* and *P. malariae* strongly indicates that *P. malariae* is specifically related to the species which are part of this subgenus.

Lastly, we also report here identification of 3 CSIs that are specifically shared by members of the subgenus *Vinckeia* and *P. ovale* but not by any other *Plasmodium* or Apicomplexa species (this grouping will be referred to here as the “Vinckeia-Ovale” clade). An example of a CSI that is exclusively shared by these species is presented in Figure 6C. In this instance, this figure shows partial sequence alignment of the protein phosphoinositide-specific phospholipase C, where a one aa insertion is uniquely present in all members of the subgenus *Vinckeia* as well as in *P. ovale*, but not in any other species.

A more detailed description of this CSI and the two other CSIs exhibiting similar specificities is provided in Figures S73–S75 and information for them is summarized in Table 3. These three CSIs strongly indicate a close and specific relationship of *P. ovale* to members of the subgenus *Vinckeia.* A specific relationship between *P. ovale* and rodent-infecting *Plasmodium* species has also been observed in earlier work [27].

### 3.7. Localizations of the CSIs in Protein Structures

**Figure 7:**
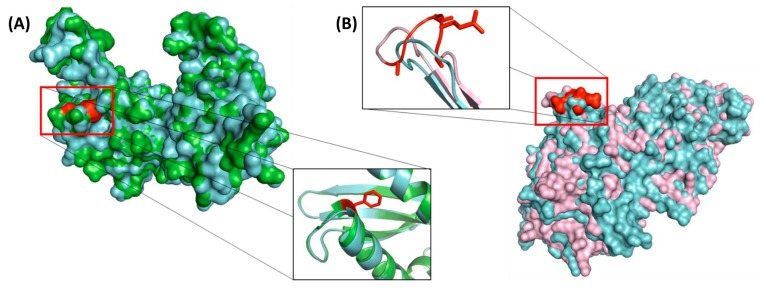
Structural localization of the identified CSIs in two of the studied proteins. (**A**) Structural overlap of a homology model of the 40S ribosomal protein S3 from *P. falciparum* containing (shown in green) a 1 aa insertion specific for the genus *Plasmodium* (shown in Figure 4A) with the solved structure of the homologous protein from *T. gondii* (PDB ascension: 5XXU_D) lacking the CSI (shown in cyan). The CSI in the protein shown in red is present in a surface-exposed loop. (**B**) Structural overlap of a homology model of the protein leucine aminopeptidase from *P. vivax* containing a 5 aa insertion (see Figure 5D) (shown in pink) with the solved structure of the protein from *P. falciparum* (PDB ascension: 4ZX8_A) (shown in cyan). The five aa CSI is highlighted in red and it is present on the surface of the protein. As shown in the close up, it extends the length of an existing loop.

Earlier work on CSIs in protein sequences show that most, if not all, of the previously studied CSIs are located on the surface exposed loops of different proteins [60,65]. The surface exposed loops are known to play important roles in mediating novel protein-protein or protein-ligand interaction [60,66,67]. In view of these earlier studies, it was of interest to determine the locations of some of the *Plasmodium*-specific CSIs in the structures of proteins. In this regard, we have determined the structural locations of the CSIs in two proteins, 40S ribosomal protein S3 containing a one aa insertion specific for all *Plasmodium* (Figure 4A) and the protein leucine aminopeptidase, which contains a five aa insertion specific for subgenus “*Plasmodium*” (Figure 5D). The structure of the 40S ribosomal protein S3, which provides an important target for the binding of several drugs (such as protein synthesis inhibitor emetine) is available from *Plasmodium* species as well as other Apicomplexa species [58]. In Figure 7A we show the structural overlap of a homology model of the 40S ribosomal protein S3 from *P. falciparum* (colored in green) with the resolved structure of the homologous protein from *T. gondii* (colored in cyan). As seen from the structural overlap of the two proteins and a close up of the CSI region of the protein (Figure 7A), where the CSI is shown in red, the inserted phenylalanine residue is present on the surface of the protein and it leads to lengthening of a surface exposed α helix.

To examine the structural location of the CSI in leucine aminopeptidase protein (Figure 5D) a homology model of this protein from *P. vivax* was constructed as described in the Methods section. This model contains a five aa insertion which is specific for the “*Plasmodium*” clade (see Figure 5D for partial sequence alignment). Figure 7B shows a structural overlap of the homology model for *P. vivax* protein (colored pink) with the resolved structure of this protein form *P. falciparum* (colored in cyan). As seen, the residues corresponding to this CSI are also present in a surface loop lengthening this region of the protein.

## 4. Discussion

The class Hematozoa harbors several clinically important genera including the genus *Plasmodium*, which is responsible for the widely-prevalent and major life-threating disease malaria [1,3,68,69]. Hence, a good understanding of the interrelationships among different organisms comprising this class and reliable means for distinguishing them are of much importance [37]. In recent years, phylogenetic studies based on different gene/protein sequences have significantly advanced our understanding of the interrelationships among the hematozoa species [3,8,10,16,19,20]. However, due to the tight clustering of *Plasmodium* species in phylogenetic trees and the dependence of branching in phylogenetic trees on a large numbers of variables [44,70,71], several relationships among the *Plasmodium* species remain ambiguous [12,27,37]. Hence, it is important to evaluate and confirm the interrelationships among Hematozoa/*Plasmodium* species by means of other sequence-based approaches that can provide more reliable information in these regards [37,41,43,44,47,50].

The present study reports detailed phylogenetic and comparative genomic analyses on protein sequences from 28 genome-sequenced hematozoa species to understand their interrelationships. Phylogenetic trees were constructed based on two independent datasets of protein sequences comprising of either 14 transcription-translation related proteins or 10 metabolism-related proteins. Both these trees showed very similar branching pattern and they support a number of previously observed relationships. These include: (i) Distinct branching of the Piroplasmida species from the genus *Plasmodium*; (ii) Distant branching of *B. microti*, the causative agent of human babesiosis [6], from other members of the genus *Babesia*; (iii) Observance of three clades within the genus *Plasmodium,* “*Haemamoeba”, “Laverania”* and “*Vinckeia”,* which correspond to the similarly named subgenera. Additionally, a clade corresponding to the subgenus *Plasmodium,* (referred to as “*Plasmodium*” in the present work) was also observed but the species *P. ovale* and *P. malariae*, which according to the current classification are also part of this subgenus [17], did not group within this clade or showed any specific association with any of the other observed clades. Additionally, in the constructed phylogenetic trees, while there was a clear separation of the Piroplasmida clade from *Plasmodium*, the latter species formed a tight cluster and their interrelationships were not reliably resolved.

**Figure 8:**
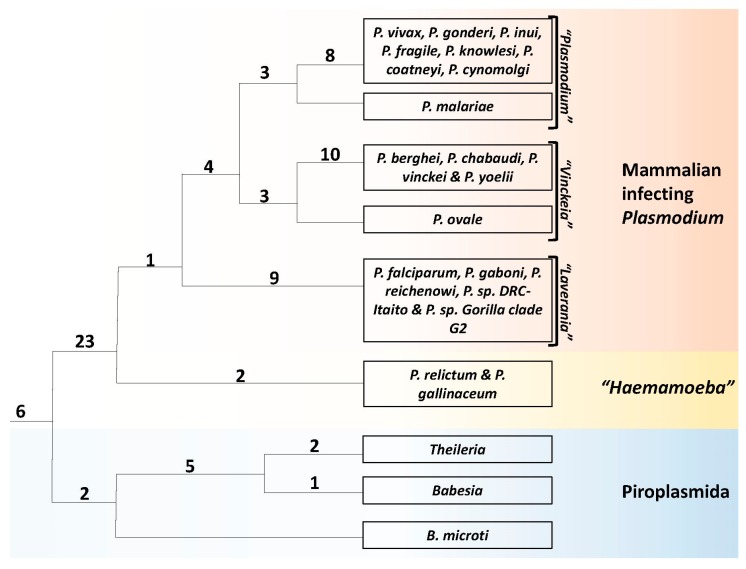
A conceptual diagram summarizing the evolutionary relationships among members of the class Hematozoa and the genus *Plasmodium* based on phylogenetic analysis and different identified molecular signatures (synapomorphies). The numbers of CSIs that are specific for different clades or species-groupings are noted on the respective nodes.

In view of these limitations of the phylogenetic tree construction approaches, a major focus of this work was on identifying CSIs that are uniquely shared by specific members of the class Hematozoa and using these characteristics for independently assessing the interrelationships among these species. As noted earlier, the CSIs represent an important class of molecular synapomorphies for reliable identification/demarcation of different monophyletic clades of organisms and assessing their interrelationships [40,41,43,44,47,50]. Extensive earlier work on these markers show that the relationships based on these rare genetic changes are minimally (and generally not) affected by different factors which confound inferences from phylogenetic approaches [44,50]. As a result, these markers have proven instrumental in resolving a number of important relationships which could not be resolved by phylogenetic approaches [40,41,43,47]. In cases where multiple CSIs supporting a given relationship are found, each CSI, present in a different gene/protein, provides independent evidence supporting the observed relationship. The present work has identified 79 CSIs that are exclusively present in specific groups of Hematozoa*/Plasmodium* species. A summary diagram showing the group-specificities of these CSIs is presented in Figure 8. It should be noted that in contrast to these CSIs, which are specific for the described groups/clades of species, our analysis has not identified any other reliable CSI(s) contradicting these relationships. In a few cases, as noted in the footnotes to the Tables and figure legends, a particular CSI in addition to being shared by different members of a given group was also present in an isolated species from another group. However, such cases were limited and showed no specific pattern or relationship. Further, for a number of *Plasmodium* species viz. *P. falciparum, P. chabaudi, P. ovale, P. yoelii, P. vinckei* and *P. vivax*, sequence information was available from a number of different strains. In these cases, as shown in the Supplementary Figures, the CSIs encompassing these species were present in all of the strains from these species and no intra-species variation in this regard was observed.

In this work (Figure 8) we have identified six CSIs that are exclusively found in all hematozoa species, two CSIs which are unique to members of the order Piroplasmida, five CSIs that are commonly shared by different *Theileria* and *Babesia* species except *B. microti*, and two CSIs which are specific for the genus *Theileria*. Additionally, large numbers of CSIs are specific for different members of the genus *Plasmodium.* Of these, 23 CSIs are distinctive characteristics of all *Plasmodium* species and two, nine, ten and eight CSIs are specifically shared by members of the subgenera “*Haemamoeba”, “Laverania”*, “*Vinckeia”* and “*Plasmodium”* (excepting *P. ovale* and *P. malariae*), respectively. Due to the exclusivity of the identified CSIs for the species from these clades, they provide novel and reliable molecular means for the demarcation of genus *Plasmodium* and a number of important groupings within it in more definitive terms.

In addition to the CSIs which are specific for different clades that are observed in phylogenetic trees, our analysis has also identified several other CSIs which support species relationships that are not evident from our trees. Of these CSIs, one CSI in the protein cysteine-tRNA ligase is uniquely shared by all mammalian-infecting *Plasmodium* species, but absent in the two avian infecting species (“*Haemamoeba”*) as well as all other Apicomplexa species. The absence of this CSI in the outgroup species (Apicomplexa as well as other eukaryotic organisms) indicates that this CSI is an insert and the genetic change responsible for this insertion occurred in a common ancestor of the mammalian-infecting *Plasmodium* species after this group diverged from other Apicomplexa-*Plasmodium* species. Although there was only one CSI identified of this kind, due to its high degree of conservation and the presence of this protein in different Apicomplexa species as well as other eukaryotic organisms, this CSI constitutes a reliable molecular characteristic. The species distribution of this CSI provides molecular evidence that the avian-infecting *Plasmodium* species are ancestral to the mammalian-infecting group of species. Although, the avian origin of mammalian-infecting species *Plasmodium* species has been suggested previously [39,64], this inference was not supported by other studies [3,12,37,72,73].

Another important inference supported by CSIs, which is not evident from phylogenetic trees, is a specific relationship of the species from the subgenera *Plasmodium* and *Vinckeia* (including *P. ovale* and *P. malariae*). The shared presences of four CSIs in 4 different proteins that are uniquely found in the members of these two subgenera provide strong evidence that the species from these two subgenera are specifically related and that they shared a common ancestor exclusive of the other *Plasmodium*-Apicomplexa species. Further, the absence of these CSIs in the outgroup species (other Apicomplexa species or eukaryotic organisms), strongly suggests that these two subgenera of *Plasmodium* have diverged subsequent to the branching/evolution of species from the subgenera *Haemamoeba* and *Laverania*, which lack these CSIs. These results indicate that the *Plasmodium* species infecting other mammals have originated after the divergence (evolution) of avian- and great apes-infecting *Plasmodium* species.

Several other CSIs identified in this work serve to clarify the evolutionary relationships of *P. ovale* and *P. malariae*, which do not group specifically with any of the four observed clades of *Plasmodium* species seen in the phylogenetic trees (Figure 1). The phylogenetic affiliation of these two species, which although are placed in the subgenus *Plasmodium,* is found to be highly variable and not resolved by earlier studies [12,23,27,28,29,62]. In this context, our identification of three CSIs, which are uniquely shared by *P. malariae* as well as by different species from the subgenus *Plasmodium* (except *P. ovale*), provides reliable evidence that *P. malariae* is specifically related to the subgenus *Plasmodium* (except *P. ovale*) and it shared a common ancestor with this group exclusive of the other *Plasmodium* genus species including *P. ovale.* Similarly, the three CSIs identified in other proteins, which are exclusively shared by *P. ovale* and different members of the subgenus *Vinckeia,* support a specific association of this species with this latter subgenus. It should be noted that while the branching of *P. malariae* with members of the subgenus *Plasmodium* and an association of *P. ovale* with *Vinckeia* subgenus has been observed in some earlier studies [12,23,27,28,29,62], it is for the first time that based on the identification of multiple CSIs, we are able to reliably show in the same study the specific associations of *P. malariae* and *P. ovale* with the subgenera *Plasmodium* and *Vinckeia*, respectively. These results indicate that *P. ovale* should be moved from the subgenus *Plasmodium* to the subgenus *Vinckeia*.

Comprehensive analysis of the hematozoa*/Plasmodium* genomes presented in this study has enabled us to develop a reliable framework for understanding the evolutionary relationships among these organisms. Although our analysis is limited by the number of genome sequences currently available for the species from this class/genus, extensive earlier work on CSIs for other groups of organisms strongly indicate that these molecular characteristics exhibit a high degree constancy and predictive ability to be found in other members of the indicated groups [40,44,49,51]. Hence, they provide us important means for clarifying the evolutionary relationships of other species related to these groups and for taxonomic studies [44,46,52]. A recent comprehensive study which has examined a broad range of *Plasmodium* species from diverse vertebrate hosts has suggested that the genus *Plasmodium* may be polyphyletic [37]. It will be of much interest to examine the presence/absence of some of the described CSIs, which are specific for the genus *Plasmodium*, in these studied species to further confirm this inference and to investigate the relationships of species within this genus.

Members of the genus *Plasmodium*, particularly the species *P. falciparum*, are the primary causative agents of the highly life-threating disease malaria [3,4,68,69,74]. Malaria is widely prevalent in Sub-Saharan Africa, Asia, and Latin America and in the year 2016 alone, 216 million people were infected with the disease leading to over 400,000 deaths [13,68,69,75]. As infections with *Plasmodium* species differ greatly in terms of the symptoms and severity of the disease, accurate identification of the causative species in the infected individual is of major importance. Diagnosis of malaria is currently based primarily on microscopic examination of the blood for infective organism or antigen-based tests [76]. However, it is of much importance to develop other rapid and reliable tests that can accurately identify the infective species. The molecular markers identified in the present study, which are specific for the genus *Plasmodium* as well as its different subgroups (subgenera), due to their presence in conserved regions and exclusivity for these groups, provide important means for developing novel and reliable diagnostic methods for the identification of different groups of *Plasmodium* species. Based on the sequence regions encompassing these CSIs, novel diagnostic tests can be developed by means of different commonly used techniques such as PCR-based, q-PCR-based, pyrosequencing, immunological or antibody-based methods, MALDI-TOF, aptamer-based methods, as well as rapid in silico identification of the CSI-containing organisms in genomic and metagenomic sequences by means of BLAST searches. In earlier work, the CSIs have been used for developing novel and highly-specific diagnostic tests for the important bacterial pathogens *Bacillus anthracis* and *Escherichia coli* O157/H7 [77,78].

A large number of the CSIs identified in this work are found in important proteins which carry out essential functions in Apicomplexa as well as other eukaryotic organisms. Although the cellular functions of these CSIs are currently not known, earlier work on CSIs in other organisms has shown that these molecular characteristics are essential or play important functional roles in the organisms where they are found [60,79,80]. The CSIs in protein sequences are generally found in the protein surface loops, which are indicated to play important roles in mediating novel protein-protein or protein-ligand interactions that are essential or important for the CSI-containing organisms [60,66,80]. The *Plasmodium*-specific CSIs in the structures of two proteins i.e., 40S ribosomal protein S3 and the leucine aminopeptidase protein, which were studied in this regard, are also both found in the surface-exposed loops of these proteins. In view of the specificity of these CSIs for *Plasmodium* or Hematozoa species, it is of much interest to determine the cellular functions of these novel molecular characteristics. Such studies could lead to discovery of novel biochemical and/or other properties that are specific and important for different groups of organisms within the class Hematozoa and the genus *Plasmodium*.

Development of resistance to existing drugs in *P. falciparum* poses one of the greatest threats in the control and treatment of malaria [68,75,81,82]. Thus, for protecting human populations from malaria, particularly in malaria endemic countries, development of new drugs that are effective in the treatment/control of malaria is a top public health priority. Therefore, identification of potential targets that could be exploited for development of new antimalarial drugs is of much importance. In this context, it is important to point out that the CSIs in protein sequences exhibit a number of characteristics, which make them potentially useful targets for development of a new class of therapeutics [83,84]. The potential usefulness of the CSIs as drug targets stems from a number of key observations: (i) they exhibit high degree of specificity for a given group of organism (e.g., *Plasmodium*); (ii) earlier work on CSIs strongly suggests that these molecular characteristics play important/essential functions in the CSI-containing organisms [80]; (iii) The CSIs in protein sequences, as demonstrated for the two CSIs examined in this study, are located in surface-exposed loops of the proteins, which are implicated in mediating novel protein-protein or protein-ligand interactions that are important for the CSI-containing organisms; (iv) Homologs of the CSI-containing proteins, or the CSIs in such homologs, are generally not found in humans. Based on these characteristics, one expects that screening for compounds, which bind specifically to the CSIs (or CSI- containing regions) and thereby inhibit the cellular functions of the CSIs could lead to the discovery of a novel class of drugs that will specifically target the *Plasmodium* species. Thus, the molecular markers identified in the present study, in addition to providing a reliable framework for understanding the evolutionary relationships among the *Plasmodium* species, provide novel means for exploring several important aspects of these clinically important parasitic organisms.

## Figures and Tables

**Table 1:** Summary of CSIs specific for the class Hematozoa and order Piroplasmida.

Protein Name	Accession Number	Figure Number	Indel Size	Indel Position	Specificity
Cell division cycle protein 48 homologue	CDS48458	2, S1	1 aa ins	106–156	Class Hematozoa
30S ribosomal protein S9, putative	CDU21364	S2	1 aa ins	449–503	
40S ribosomal protein S12, putative	XP_677491	S3	1 aa ins	62–109	
20S proteasome β 4 subunit	CDS50559	S4	1 aa ins	04–59	
Golgi reassembly-stacking protein 1	XP_012769739	S5	1 aa del	90–136	
Pyruvate kinase 2	XP_680419	S6	1 aa ins	461–515	
Succinyl-CoA synthetase β chain	XP_004833199	3 (A), S7	1 aa del	273–315	Order Piroplasmida
Hypothetical protein, conserved	XP_954617	S8	1 aa ins	1856–1907	
Dihydrolipoamide dehydrogenase	XP_763245	3 (B), S9	1 aa del	415–465	Order Piroplasmida, except *B. microti*
Conserved hypothetical protein	XP_004832720	S10	1 aa del	19–74	
Conserved hypothetical protein	XP_004832649	S11	2 aa del	334–388	
Intron-binding aquarius β like	XP_012767741	S12	2 aa del	1280–1322	
Intron-binding aquarius β like	XP_012767742	S13	5/8 aa del	1303–1346	
Cysteinyl-tRNA synthetase	XP_001608890	S14	2 aa ins	261–316	Genus *Babesia* (except *B. microti)*
Cysteinyl-tRNA synthetase	XP_001608890	S14	1 aa ins	261–316	Genus *Theileria*
Eukaryotic translation initiation factor 4a	XP_764692	S15	1 aa del	225–279	

**Table 2:** Summary of CSIs specific for the Genus *Plasmodium* and some of its clades/groups.

Protein Name	Accession number	Figure Number	Indel Size	Indel Position	Specificity
40S ribosomal protein S3	CDS50468	4 (A), S16	1 aa ins	50–103	Genus *Plasmodium*
26S proteasome regulatory subunit RPN2	CDS50476	S17	2 aa ins	777–826	
26S proteasome regulatory subunit 4	XP_673015	S18	1 aa del	64–116	
40S ribosomal protein S25, putative	XP_001348379	S19	1 aa del	35–79	
50S ribosomal protein L1, mitochondrial, putative *	XP_674529	S20	1 aa del	126–167	
60S ribosomal protein L35, putative	XP_001347931	S21	1 aa del	25–64	
Asparagine-tRNA ligase, putative*	XP_680201	S22	1 aa del	436–487	
ATP-dependent RNA helicase DBP10	CDS45652	S23	1 aa ins	385–438	
Alternative splicing regulator, putative	XP_001349840	S24	1 aa del	296–338	
Adenosinetriphosphatase protein	XP_677822	S25	1 aa del	387–437	
DNA2/NAM7 helicase	CDS49097	S26	3/4 aa ins	950–1013	
Elongation factor Tu family	CDS46726	S27	1 aa ins	335–380	
Multidrug resistance protein 2	CDS50130	S28	1 aa del	438–488	
Pre-mRNA-processing-splicing factor 8	XP_022714222	S29	2 aa ins	1174–1230	
Pyruvate dehydrogenase E1 component, α subunit	CDS46715	S30	1 aa ins	218–272	
Pyruvate dehydrogenase E1 component, α subunit	CDS46715	S31	1 aa ins	406–451	
Ras-related protein Rab-5A	XP_677676	S32	2 aa del	103–162	
Ribosomal protein L27a, putative	XP_677367	S33	1 aa ins	97–142	
RuvB-like helicase isoform 1	XP_673190	S34	1 aa ins	121–178	
RuvB-like helicase isoform 1	XP_673190	S35	1 aa del	96–144	
RuvB-like helicase isoform 2	CDS46888	S36	1 aa ins	136–194	
Splicing factor 3A subunit 2	XP_676664	S37	1 aa del	111–157	
Translation initiation factor SUI1	SCM24886	S38	1 aa del	181–221	
Cysteine-tRNA ligase protein	XP_024329092	4 (B), S39	1 aa ins	135–185	Mammalian infecting clades
Protein phosphatase	CRH00140	5 (A), S40	1 aa ins	146–197	Avian clade, *“Haemamoeba*”
Conserved Plasmodium protein	CRG96454	S41	1 aa ins	5–58	

* CSI region is only conserved within *Plasmodium* and various bacteria.

**Table 3:** Summary of CSIs specific for mammalian-infecting *Plasmodium* clades.

Protein Name	Accession number	Figure Number	Indel Size	Indel Position	Specificity
Eukaryotic translation initiation factor 3 subunit D	XP_001347362	5 (B), S42	1 aa ins	82–130	Subgenus *“Laverania*” *a*
Conserved hypothetical protein	XP_001349841	S43	1 aa ins	322–365	
Aconitate hydratase protein	XP_001350142	S44	1 aa ins	450–494	
Conserved Plasmodium protein	XP_024329193	S45	1 aa ins	528–575	
Cation-transporting ATPase	XP_001349175.1	S46	1 aa ins	576–633	
Serine/threonine protein kinase	XP_001349887	S47	2 aa ins	175–222	
Tetratricopeptide repeat family protein	KOB86259	S48	1 aa ins	548–594	
Thioredoxin-like protein	XP_001348359	S49	1 aa ins	125–171	
Pre-mRNA-processing-splicing factor 8 *	XP_001351366	S50	1 aa ins	2635–2685	
Mitochondrial ribosomal protein L17-2 precursor	XP_022714285	5 (C), S51	1 aa ins	44–92	Subgenus *“Vinckeia*” *P.*
Gdp-mannose 4,6 dehydratase	XP_678388	S52	1 aa ins	119–156	
14-3-3 protein	XP_022714317	S53	1 aa ins	385–431	
Conserved Plasmodium protein	XP_679884	S54	1 aa del	380–429	
Conserved Plasmodium protein	XP_022714261	S55	4 aa ins	113–167	
Conserved Plasmodium protein	XP_678492	S56	1 aa ins	893–932	
M17 leucyl aminopeptidase protein	XP_678034	S57	1 aa ins	199–249	
PelOta protein homologue, putative	CAH99124.1	S58	1 aa del	181–232	
LCCL domain-containing protein	XP_679041	S59	1 aa ins	166–212	
DEAD-box family helicase 4 protein	XP_019913407	S60	1 aa ins	442–491	
DEAD-box family helicase 4 protein	XP_019913407	S60	2 aa ins	442–491	Subgenus *“Plasmodium”*
Conserved Plasmodium protein	CRG96454	S41	1 aa del	5–58	
Leucine aminopeptidase protein	SCO68989	5 (D), S61	4/5 aa ins	346–401	
Conserved hypothetical protein	XP_001616802	S62	1 aa ins	384–433	
Hypothetical protein PVBG_03892	KMZ97769	S63	1 aa ins	879–922	
Hypothetical protein PVMG_00581	KMZ97919	S64	1 aa ins	140–179	
Hypothetical protein PVNG_02680	KMZ97919	S65	1 aa ins	278–323	
Serine/threonine protein kinase ^+^	SCO74371	S66	1 aa ins	546–598	
Biotin-acetyl-CoA-carboxylase ligase protein	68069783	6 (A), S67	1 aa ins	28–68	“Vinckeia-Plasmodium” clade
Hypothetical protein PVIIG_05030	KMZ81663	S68	1 aa ins	347–394	
Conserved Plasmodium protein	XP_679973	S69	1 aa del	227–276	
Conserved Plasmodium protein	KMZ97769	S70	2 aa ins	688–737	
Conserved Plasmodium protein	KMZ97769	S70	1 aa ins	688–737	“Plasmodium-Malariae” clade
Ubiquitin-activating enzyme E1 protein	901876239	6 (B), S71	2 aa ins	153–204	
Hypothetical protein PVNG_01558	KNA00692	S72	1 aa ins	277–323	
Phosphoinositide-specific phospholipase C protein	1269288476	6 (C), S73	1/2 aa ins	190–238	“Vinckeia-Ovale” clade
DEAD/DEAH helicase protein	1269289107	S74	1 aa ins	144–192	
ATPase protein	XP_022712410	S75	2 aa ins	764–811	

* CSI is specific for a subclade of *Laverania* consisting of *P. falciparum* and *P. reichenowi*; ^+^ CSI is also shared by some *Laverania* species.

## Supplementary Materials

The following are available online at https://www.mdpi.com/2073-4425/10/7/490/s1, **Table S1.** Some characteristics of the Hematozoa genomes used in Phylogenetic/Comparative Genomic Studies. **Table S2.** *P. falciparum* protein sequences utilized to construct the tree shown in Figure 1A. **Table S3.** *P. falciparum* protein sequences utilized to construct the tree shown in Figure 1B. **Table S4.** Summary of some characteristics of *Plasmodium* species featured in Figure 1. **Figure S1.** Partial sequence alignment of Cell division cycle protein 48 homologue showing a Hematozoa specific CSI. **Figure S2.** Partial sequence alignment of 30S ribosomal protein S9 showing a Hematozoa specific CSI. **Figure S3.** Partial sequence alignment of 40S ribosomal protein S12 showing a Hematozoa specific CSI. **Figure S4.** Partial sequence alignment of 20S proteasome β 4 subunit protein showing a Hematozoa specific CSI. **Figure S5.** Partial sequence alignment of Golgi reassembly-stacking protein 1 showing a Hematozoa specific CSI. **Figure S6.** Partial sequence alignment of Pyruvate kinase 2 protein showing a Hematozoa specific CSI. **Figure S7.** Partial sequence alignment of Succinyl-CoA synthetase β chain protein showing a Piroplasmida specific CSI. **Figure S8.** Partial sequence alignment of a Hypothetical protein showing a Piroplasmida specific CSI. **Figure S9.** Partial sequence alignment of Dihydrolipoamide dehydrogenase protein showing a Piroplasmida (except *B. microti*) specific CSI. **Figure S10.** Partial sequence alignment of a Hypothetical protein showing a Piroplasmida (except *B. microti*) specific CSI. **Figure S11.** Partial sequence alignment of a Hypothetical protein showing a Piroplasmida (except *B. microti*) specific CSI. **Figure S12.** Partial sequence alignment of Intron-binding aquarius β like protein showing a Piroplasmida (excluding *B. microti*) specific CSI. **Figure S13.** Partial sequence alignment of Intron-binding aquarius β like protein showing a Piroplasmida (excluding *B. microti*) specific CSI. **Figure S14.** Partial sequence alignment of Cysteinyl-tRNA synthetase protein showing a *Babesia* (excluding *B. microti*) specific CSI and a *Theileria* specific CSI. **Figure S15.** Partial sequence alignment of Eukaryotic translation initiation factor 4a protein showing a *Theileria* specific CSI. **Figure S16.** Partial sequence alignment of 40S ribosomal protein S3 showing a *Plasmodium* specific CSI. **Figure S17.** Partial sequence alignment of 26S proteasome regulatory subunit RPN2 protein showing a *Plasmodium* specific CSI. **Figure S18.** Partial sequence alignment of 26S proteasome regulatory subunit 4 protein showing a *Plasmodium* specific CSI. **Figure S19.** Partial sequence alignment of 40S ribosomal protein S25 showing a *Plasmodium* specific CSI. **Figure S20.** Partial sequence alignment of 50S ribosomal protein L1, mitochondrial showing a *Plasmodium* specific CSI. **Figure S21.** Partial sequence alignment of 60S ribosomal protein L35 showing a *Plasmodium* specific CSI. **Figure S22.** Partial sequence alignment of Asparagine-tRNA ligase protein showing a *Plasmodium* specific CSI. **Figure S23.** Partial sequence alignment of ATP-dependent RNA helicase DBP10 protein showing a *Plasmodium* specific CSI. **Figure S24.** Partial sequence alignment of Alternative splicing regulator protein showing a *Plasmodium* specific CSI. **Figure S25.** Partial sequence alignment of Adenosinetriphosphatase protein showing a *Plasmodium* specific CSI. **Figure S26.** Partial sequence alignment of DNA2/NAM7 helicase protein showing a *Plasmodium* specific CSI. **Figure S27.** Partial sequence alignment of Elongation factor Tu family protein showing a *Plasmodium* specific CSI. **Figure S28.** Partial sequence alignment of Multidrug resistance protein 2 showing a *Plasmodium* specific CSI. **Figure S29.** Partial sequence alignment of Pre-mRNA-processing-splicing factor 8 protein showing a *Plasmodium* specific CSI. **Figure S30.** Partial sequence alignment of Pyruvate dehydrogenase E1 component, α subunit protein showing a *Plasmodium* specific CSI. **Figure S31.** Partial sequence alignment of Pyruvate dehydrogenase E1 component, α subunit protein showing a *Plasmodium* specific CSI. **Figure S32.** Partial sequence alignment of Ras-related protein Rab-5A showing a *Plasmodium* specific CSI. **Figure S33.** Partial sequence alignment of Ribosomal protein L27a showing a *Plasmodium* specific CSI. **Figure S34.** Partial sequence alignment of RuvB-like helicase isoform 1 protein showing a *Plasmodium* specific CSI. **Figure S35.** Partial sequence alignment of RuvB-like helicase isoform 1 protein showing a *Plasmodium* specific CSI. **Figure S36.** Partial sequence alignment of RuvB-like helicase isoform 2 protein showing a *Plasmodium* specific CSI. **Figure S37.** Partial sequence alignment of Splicing factor 3A subunit 2 protein showing a *Plasmodium* specific CSI. **Figure S38.** Partial sequence alignment of Translation initiation factor SUI1 protein showing a *Plasmodium* specific CSI. **Figure S39.** Partial sequence alignment of Cysteine-tRNA ligase protein showing a Mammalian infecting clades specific CSI. **Figure S40.** Partial sequence alignment of Protein phosphatase protein showing a subgenus “*Haemamoeba*” specific CSI. **Figure S41.** Partial sequence alignment of Conserved *Plasmodium* protein showing a subgenus “*Haemamoeba*” specific CSI and a subgenus “*Plasmodium*” specific CSI. **Figure S42.** Partial sequence alignment of Eukaryotic translation initiation factor 3 subunit D protein showing a subgenus “*Laverania*” specific CSI. **Figure S43.** Partial sequence alignment of Conserved hypothetical protein showing a subgenus “*Laverania*” specific CSI. **Figure S44.** Partial sequence alignment of Aconitate hydratase protein showing a subgenus “*Laverania*” specific CSI. **Figure S45.** Partial sequence alignment of Conserved *Plasmodium* protein showing a subgenus “*Laverania*” specific CSI. **Figure S46.** Partial sequence alignment of Cation-transporting ATPase protein showing a subgenus “*Laverania*” specific CSI. **Figure S47.** Partial sequence alignment of Serine/threonine protein kinase showing a subgenus “*Laverania*” specific CSI. **Figure S48.** Partial sequence alignment of Tetratricopeptide repeat family protein showing a subgenus “*Laverania*” specific CSI. **Figure S49.** Partial sequence alignment of Thioredoxin-like protein showing a subgenus “*Laverania*” specific CSI. **Figure S50.** Partial sequence alignment of Pre-mRNA-processing-splicing factor 8 protein showing a subgenus “*Laverania*” specific CSI. **Figure S51.** Partial sequence alignment of Mitochondrial ribosomal protein L17-2 precursor protein showing a subgenus “*Vinckeia*” specific CSI. **Figure S52.** Partial sequence alignment of Gdp-mannose 4,6 dehydratase protein showing a subgenus “*Vinckeia*” specific CSI. **Figure S53.** Partial sequence alignment of 14-3-3 protein showing a subgenus “*Vinckeia*” specific CSI. **Figure S54.** Partial sequence alignment of Conserved *Plasmodium* protein showing a subgenus “*Vinckeia*” specific CSI. **Figure S55.** Partial sequence alignment of Conserved *Plasmodium* protein showing a subgenus “*Vinckeia*” specific CSI. **Figure S56.** Partial sequence alignment of Conserved *Plasmodium* protein showing a subgenus “*Vinckeia*” specific CSI. **Figure S57.** Partial sequence alignment of M17 leucyl aminopeptidase protein showing a subgenus “*Vinckeia*” specific CSI. **Figure S58.** Partial sequence alignment of PelOta protein homologue showing a subgenus “*Vinckeia*” specific CSI. **Figure S59.** Partial sequence alignment of LCCL domain-containing protein showing a subgenus “*Vinckeia*” specific CSI. **Figure S60.** Partial sequence alignment of DEAD-box family helicase 4 protein showing a subgenus “*Vinckeia*” specific CSI and a subgenus “*Plasmodium*” specific CSI. **Figure S61.** Partial sequence alignment of Leucine aminopeptidase protein showing a subgenus “*Plasmodium*” specific CSI. **Figure S62.** Partial sequence alignment of Conserved hypothetical protein showing a subgenus “*Plasmodium*” specific CSI. **Figure S63.** Partial sequence alignment of Hypothetical protein PVBG_03892 showing a subgenus “*Plasmodium*” specific CSI. **Figure S64.** Partial sequence alignment of Hypothetical protein PVMG_00581 showing a subgenus “*Plasmodium*” specific CSI. **Figure S65.** Partial sequence alignment of Hypothetical protein PVNG_02680 showing a subgenus “*Plasmodium*” specific CSI. **Figure S66** Partial sequence alignment of Serine/threonine protein kinase showing a subgenus “*Plasmodium*” specific CSI. **Figure S67.** Partial sequence alignment of Biotin-acetyl-CoA-carboxylase ligase protein showing a “*Vinckeia*-*Plasmodium*” clade specific CSI. **Figure S68.** Partial sequence alignment of Hypothetical protein PVIIG_05030 showing a “*Vinckeia*-*Plasmodium*” clade specific CSI. **Figure S69.** Partial sequence alignment of Conserved *Plasmodium* protein showing a “*Vinckeia*-*Plasmodium*” clade specific CSI. **Figure S70.** Partial sequence alignment of Conserved *Plasmodium* protein showing a “*Vinckeia*-*Plasmodium*” clade specific CSI and a “*Plasmodium*-*Malariae*” clade specific CSI. **Figure S71.** Partial sequence alignment of Ubiquitin-activating enzyme E1 protein showing a “*Plasmodium*-*Malariae*” clade specific CSI. **Figure S72.** Partial sequence alignment of Hypothetical protein PVNG_01558 showing a “*Plasmodium*-*Malariae*” clade specific CSI. **Figure S73.** Partial sequence alignment of Phosphoinositide-specific phospholipase C protein showing a “*Vinckeia*-*Ovale*” clade specific CSI. **Figure S74.** Partial sequence alignment of DEAD/DEAH helicase protein showing a “*Vinckeia*-*Ovale*” clade specific CSI. **Figure S75.** Partial sequence alignment of ATPase protein showing a “*Vinckeia*-*Ovale*” clade specific CSI.
