# Supplementary material for: Novel Molecular Synapomorphies Demarcate Different Main Groups/Subgroups of Plasmodium and Piroplasmida Species Clarifying Their Evolutionary Relationships
Source: Genes (Basel). 2019 Jun 28;10(7):490. doi: 10.3390/genes10070490 (PMC6678196; doi:10.3390/genes10070490)
Supplement: Supplementary file 1 [file genes-10-00490-s001.pdf]

Group	Species	Accession	106		156
Hematozoa — ***Plasmodium* (20/20)**	*Plasmodium berghei ANKA*	CDS48458	CPEIPYGKKIQVLPIDDTIEGLAKD	T	LFEIFLKPYFNESYRPVKKGDLFLV
	Plasmodium sp. DRC-Itaito	SOV21700.1	-------------------------	-	-------------------------
	Plasmodium sp. Gor. Cla. G2	SOV12605.1	-------------------------	-	-------------------------
	Plasmodium reichenowi	XP_012762007.1	-------------------------	-	-------------------------
	Plasmodium chabaudi cha.	CAH74972	-------------------------	-	-------------------------
	Plasmodium coatneyi	XP_019915657	--------------L----------	-	-------------------------
	Plasmodium cynomolgi str. B	XP_004223308	--------------L----------	-	-------------------------
	Plasmodium falciparum 3D7	XP_966179	-------------------------	-	-------------------------
	Plasmodium falciparum Dd2	KOB85869	-------------------------	-	-------------------------
	Plasmodium falciparum RAJ116	KNC35528	-------------------------	-	-------------------------
	Plasmodium falciparum Tan.	ETW33446	-------------------------	-	-------------------------
	Plasmodium fragile	XP_012335274	--------------L----------	-	-------------------------
	Plasmodium gaboni	XP_018642827	-------------------------	-	-------------------------
	Plasmodium gallinaceum	CRG93772	-------------------------	-	-------------------------
	Plasmodium gonderi	GAW81833	-------------------------	-	-------------------------
	Plasmodium inui San Ant. 1	XP_008814739	--------------L----------	-	----------S--------------
	Plasmodium knowlesi str. H	XP_002261482	--------------L----------	-	-------------------------
	Plasmodium malariae	SCO93189	-------------------------	-	-------------------------
	Plasmodium ovale curt.	SBS85350	-------------------------	-	-------------------------
	Plasmodium ovale wal.	SBT38154	-------------------------	-	-------------------------
	Plasmodium relictum	CRH00942	-------------------------	-	-------------------------
	Plasmodium vinckei pet.	EUD73300	-------------------------	-	-------------------------
	Plasmodium vinckei vin.	XP_008624401	-------------------------	-	-------------------------
	Plasmodium vivax Sal-1	XP_001616207	--------------L----------	-	-------------------------
	Plasmodium yoelii yoe. 17XNL	XP_723826	-------------------------	-	-------------------------
***Piroplasmida* (8/8)**	*Theileria annulata*	XP_953837	--DV--------------V---S-E	S	--NVY-----L--------------
	Theileria equi	XP_004833533	--DV----------L---V---SRE	S	--NVY-----L--------------
	Theileria orientalis str. Shi.	XP_009689731	--DV----------F---V---S-E	S	--NVY-----L--------------
	Theileria parva str. Mug.	XP_766458	--DV--------------V---S-E	S	--NVY-----L--------------
	Babesia bigemina	XP_012766454	-SDV----------L---V---S-E	A	--DVY-----L-T------------
	Babesia bovis T2Bo	XP_001610758	-SDV----------L---V---SR-	A	--DVY-----L--------------
	Babesia sp. Xin.	ORM42007	-SDV----------L---V---SR-	A	--DVY-----L-T------------
	Babesia ovata	GBE60577.1	-SDV--------------V---S-E	A	--DVY-----L-T------------
	Babesia microti str. RI	XP_021338827	-ADV----R-------------SRG	S	--D-Y-----M----------Y---
**Other Apicomplexa**	*Eimeria brunetti*	CDJ46587	-ADC----R-H---L------ITGN		----Y-----M-A----R-N-----
	Eimeria maxima	XP_013336708	-ADC----R-H---L------ITGN		----Y-----M-A----R-N-----
	Eimeria mitis	CDJ36397	-ADC----R-H---L------ITGN		----Y-----M-A----R-N-----
	Eimeria necatrix	XP_013440163	-ADC----R-H---L------ITGN		----Y-----M-A----R-N-----
	Eimeria praecox	CDI74800	-ADC----R-H---L------ITGN		----Y-----M-A----R-N-----
	Eimeria tenella	XP_013230913	-ADC----R-H---L------ITGN		----Y-----M-A----R-N-----
	Neospora caninum Liv.	CEL67655	--DC----R-H---L------ITGN		--D-Y-----M-A----R-------
	Hammondia hammondi	XP_008882621	--DC----R-H---L------ITGN		--D-Y-----M-A----R-------
	Toxoplasma gondii ME49	XP_002365921	--DC----R-H---L------ITGN		--D-Y-----M-A----R-------
**Other Eukarya**	*Acanthaster planci*	XP_022087240	--DVK---R-H---L---V---SGN		---VY-----T-A----R---I---
	Acanthochromis polyacanthus	XP_022072805	--D-K-----H-----------TGN		---V------L-A---IH---I---
	Achlya hypogyna	OQR93065	-GDV----R-H----------VTGN		--DVY-----L-A------------
	Acropora digitifera	XP_015774896	--DVK---R-H-------V---TGN		--DVY-----L-A---IR---M---
	Albugo candida	CCI43479	-N-V----R-H----------VSGN		--DVY-----V-A------------
	Albugo laibachii Nc14	CCA19151	-N-V----R-H----------VSGN		--DVY-----V-A------------
	Alligator sinensis	XP_006026624	--DVK---R-H-------V--ITGN		---VY-----L-A---IR---I---
	Amazona aestiva	KQK82914	--DVK---R-H-------V--ITGN		---VY-----L-A---IR---I---
	Amblyomma variegatum	DAA34120	--DVK---R-H-------V---SGS		---VY-----L-A---IH-------
	Anas platyrhynchos	XP_005028113	--DVK---R-H-------V--ITGN		---VY-----L-A---IR---I---
	Anolis carolinensis	XP_008102421	--DVK---R-H--------A--TGN		---VY-----L-A----H---I---
	Anser cygnoides dom.	XP_013055945	--DVK---R-H-------V--ITGN		---VY-----L-A---IR---I---
	Aphanomyces astaci	XP_009838057	-GDV----R-H----------VTGN		--DVY-----L-A------------
	Gekko japonicus	XP_015262300	--DVK---R-H-------V--ITGN		---VY-----L-A---IR---I---
	Haemaphysalis longicornis	BAF73714	--DVK---RTH-------V----GS		---VY-----L-A---IH-------
	Haplochromis burtoni	XP_005931432	--D-K-----H-----------SGN		--DV------L-A----H---I---
	Hyalella azteca	XP_018015526	--DVK---R-H-----------SGN		I--VY-----L-A---IH-----M-
	Hyaloperonospora parasitica	AAY58902	-GDV----R-H----------VTGN		--DVY-----V-A------------
	Ictalurus punctatus	ABD77544	--DVK---R-H-------V--ITGN		---VY-----L-A---IR---I---

Figure S1. A partial sequence alignment of the Cell division cycle protein 48 homologue showing a one amino acid insertion that is specific for members of *Hematozoa*. Expanded alignment of Figure 2.

Group	Organism	Accession	449		503
Hematozoa – **Plasmodium (20/20)**	*Plasmodium berghei ANKA*	CDU21364	FNVVAHVEGGGLTGQSVAIFHALVKYIVY	N	FSLKIKPFFRSFKFMTVDSRKVERK
	Plasmodium chabaudi ada.	SCM08058	-----------------------------	-	-------------------------
	Plasmodium chabaudi cha.	CAH81800	-----------------------------	-	-------Y-----------------
	Plasmodium coatneyi	XP_019917249	-----R-V----S----------------	-	L------Y-----------------
	Plasmodium cynomolgi str. B	XP_004224506	-----R-V----S----------------	-	-------Y-----------------
	Plasmodium falciparum 3D7	XP_001348305	--I--K------S----------------	-	-----------LN-----T------
	Plasmodium falciparum HB3	KOB63256	--I--K------S----------------	-	-----------LN-----T------
	Plasmodium falciparum Mal.	ETW46996	--I--K------S----------------	-	-----------LN-----T------
	Plasmodium falciparum RAJ.	KNC37212	--I--K------S----------------	-	-----------LN-----T------
	Plasmodium fragile	XP_012338246	-----R-V----S----------------	-	L------Y-----------------
	Plasmodium gaboni	XP_018639212	--II-T------S----------------	-	-------Y---LN------------
	Plasmodium gallinaceum	CRG95653	-----R-K----S--------------T-	-	------------N------------
	Plasmodium gonderi	GAW83082	-----K-I----S--------------A-	-	L------Y-----------------
	Plasmodium inui San Ant. 1	XP_008817531	-----K-V----S----------------	-	L------Y-----------------
	Plasmodium knowlesi str. H	XP_002260863	-----R-V----S----------------	-	L------Y-----------------
	Plasmodium malariae	SBS89743	-----K------S--------------T-	-	-------Y-----------------
	Plasmodium ovale curt.	SBS87590	-D---K-----------------------	-	-------------------------
	Plasmodium ovale wal.	SBT46005	-D---K-----------------------	-	-------------------------
	Plasmodium reichenowi	CDO66618	--I--K------S----------------	-	-----------LN-----T------
	Plasmodium relictum	CRH04003	-----K------S--------------T-	-	-----------LN------------
	Plasmodium vinckei pet.	EUD73728	-----------------------------	-	-------------------------
	Plasmodium vinckei vin.	XP_008624014	-----------------------------	-	-------------------------
	Plasmodium vivax Ind. VII	KMZ78282	-----R-V----S----------------	-	L------Y-----------------
	Plasmodium vivax Mau. I	KMZ90724	-----R-V----S----------------	-	L------Y-----------------
	Plasmodium vivax Sal-1	XP_001616819	-----R-V----S----------------	-	L------Y-----------------
	Plasmodium yoelii 17X	ETB59524	-----------------------------	-	----------------I--------
	Plasmodium yoelii yoe. 17XNL	XP_727975	-----------------------------	-	----------------I--------
	Plasmodium sp. DRC-Itaito	SPJ12531.1	--I--K------S----------------	-	-------Y---LN------------
	Plasmodium sp. Gor. Cla. G2	SOV18789.1	--II-T------S----------------	-	-------Y---LN------------
Hematozoa – **Piroplasmida (8/8)**	*Theileria equi*	XP_004828890	--I--Q-R----S------R--V----YT	I	LAP-L---L-ICDLV-P-I-RT---
	Theileria orientalis str. Shi.	XP_009690373	--I--KAH---IS------R------LYR	I	L-P-L--IL-K-DLVSI-R-R----
	Theileria annulata	XP_952372	--I--MTQ---IA------R----R-LYR	I	L-P-L--IL-K-NLVSI-R-R----
	Theileria parva str. Mug.	XP_764913	--I--KTK---IS------R----R-LYR	I	L-P-L-YIL-K-DLVSI-R-RT---
	Babesia microti str. RI	XP_012649249	---I-TAR---IS--AN------ST--YT	T	-LPIS--IL-Y-GLV-R-L-R----
	Babesia bigemina	XP_012769112	--I--VAR---IS--TG--R---AR-LTK	V	LAP--E-YLSMRDLTKA-T-Q----
	Babesia sp. Xin.	ORM41942	---I-V-H---I---AG--R---SR--TK	I	LAP-LE-YLSMRDLTRA-T-Q----
	Babesia bovis T2Bo	XP_001611744	--I--R-N---IS---G--R---CR--SR	V	LAP-LDSYLSMRGLGKA-T-Q----
	Babesia ovata	GBE59003.1	--I--VAR---IS--TG--R---AR-LTK	V	LAP-LE-YLSMRDLTKA-T-Q----
**Other Apicomplexa**	*Toxoplasma gondii ME49*	237842723	-DII-EAH----G---G--ML-VARE--R		QRPELR-PL-RAG-L---A------
	Hammondia hammondi	675134962	-DII-EAH----G---G--ML-VARE--R		QRPELR-PL-RAG-L---A------
	Neospora caninum Liv.	401412614	-D-I-EAH----G---G--ML-VARE--R		QRPELR-PL-RAG-L---A------
	Besnoitia besnoiti	1261484042	-DII-QAH----G---G--ML-VARE--R		QRPELRSPL-RAG-L---A------
**Proteo-bacteria**	*Agrobacterium radiobacter K84*	ACM26154	-DII-T-A----S--AG-VR-G-S-ALT-		-EPGLRSVLKKGG-L-R---V----
	Ehrlichia chaffeensis str. Osc	AHX07647	Y----T-S---IS--AG-VA-GIS-ALKD		INPSLH-IL--GG-L-R---V----
	Rhizobium sp. ACO-34A	ATN33929	-DII-T-T----S--AG-VR-G-S-ALT-		-EPGLRSVLKKGG-L-R---V----
	Azospirillum sp. B510	BAI73563	-D---T-A----S--AG-VR-GIS-ALT-		-EPALR-PLKAAG-L-R-A-T----
	Rhizobium sp. Pop5	EJZ23188	-DI--T-A----S--AG-VR-G-S-ALT-		-EPGLRSVLKKGG-L-R---V----
	Kiloniella spongiae	KLN59395	-D--CT-K----S--AG-VR-GIS-ALT-		-EPGLR-ALKARG-L-R-A-V----

Figure S2. A partial sequence alignment of the 30S ribosomal protein S9 showing a one amino acid insertion that is specific for members of *Hematozoa*. Some exceptions were seen in class *Phaeophyceae*.

Group	Species	Accession	62		109
Hematozoa – *Plasmodium* (20/20)	*Plasmodium berghei ANKA*	XP_677491	VCSEPAYKKLVTALCAEKQIPLFMI	D	NDSKDLGQWSGLFKVDKEGNAR
	Plasmodium sp. DRC-Itaito	SOV20819.1	------------S-----N-----V	Q	-------H-A----L-N-----
	Plasmodium sp. Gor. Cla. G2	SOV10760.1	------------S-----N-----V	Q	-------H-A----L-N-----
	Plasmodium reichenowi	XP_012761146.1	----------I-T-----N-----V	Q	-------H-A----L-N-----
	Plasmodium chabaudi cha.	XP_746161	-------------------------	-	----------------------
	Plasmodium coatneyi	XP_019914394	----------I----T--N-----V	E	---------V----L-------
	Plasmodium cynomolgi str. B	XP_004222208	----------I----T--N-----V	E	---------V----L-------
	Plasmodium falciparum 3D7	XP_001351146	----------I-T-----N-----V	Q	-------H-A----L-N-----
	Plasmodium falciparum FCH/4	ETW32176	----------I-T-----N-----V	Q	-------H-A----L-N-----
	Plasmodium falciparum NF54	EWC88016	----------I-T-----N-----V	Q	-------H-A----L-N-----
	Plasmodium falciparum Palo Alt.	ETW52259	----------I-T-----N-----V	Q	-------H-A----L-N-----
	Plasmodium fragile	XP_012333752	----------I----T--N-----V	E	---------V----L-------
	Plasmodium gaboni	XP_018643510	------------S-----N-----V	Q	-------H-A----L-N-----
	Plasmodium gallinaceum	CRG95298	----------I----T--N----LV	Q	-------H-A--Y-L-------
	Plasmodium gonderi	GAW80748	----------I-------N------	-	---------V----L-------
	Plasmodium inui San Ant. 1	XP_008817753	----------I----T--N-----V	E	---------V----L-------
	Plasmodium knowlesi str. H	XP_002258974	----------I----T--N-----V	E	---------V----L-------
	Plasmodium malariae	SBS91739	------------------N-----V	-	---------V----L-------
	Plasmodium ovale curt.	SBS83114	Q-----------------N----E-	E	-G-------V----L-------
	Plasmodium relictum	CRG99857	----------I-------N----LV	Q	-------H-A--Y-L--D----
	Plasmodium vinckei pet.	EUD70784	-------------------------	-	---------A----I-------
	Plasmodium vivax Ind. VII	KMZ80787	----------I----T--N-----V	E	---------V----L-------
	Plasmodium vivax North Kor.	KMZ99880	----------I----T--N-----V	E	---------V----L-------
	Plasmodium vivax Sal-1	XP_001613092	----------I----T--N-----V	E	---------V----L-------
	Plasmodium yoelii	XP_726264	-------------------------	-	----------------------
Hematozoa – *Piroplasmida* (8/8)	*Theileria annulata*	XP_955410	G------V---Q---K-HS---IET	-	V---T-------C-Y-I--KP-
	Theileria equi	XP_004832661	G------V---Q---K-HS---IET	-	C---T----A--C-Y-V--KP-
	Theileria orientalis str. Shi.	XP_009691194	E------V---QS--K-HS---IET	-	V---T-------C-Y-I--KP-
	Theileria parva str. Mug.	XP_762841	G------V---Q---K-HS---IET	-	V---T-------C-Y-I--KP-
	Babesia ovata	GBE63056.1	--EG-L--IL---K-HGV-CIET	-	A-C-TV-M-A--C-Y-I--V--
	Babesia bovis T2Bo	XP_001611245.1	--E--L--IR---K-HGV-CIET	E	F--EKI-E-A--C-Y-I--V--
	Babesia bigemina	XP_012768531.1	--EG-L--IL---K-HGV-CIET	-	A-C-TV-K-A--C-Y-I--V--
	Babesia microti str. RI	XP_012648096	S------T--IN---K-HNV--IE-	-	G---T----T--C-I-P--KP-
Other Apicomplexa	*Besnoitia besnoiti*	PFH31046	G----------QG--K-HG---LDV		T---E--E-A--C----D-T--
	Cyclospora cayetanensis	XP_022586488	S----------GG--K-HG---IEV		AE-----E-A--CRI-H--AP-
	Cystoisospora suis	PHJ19249	S---A------QG--K-HG---IDV		L---E----A--C-A----VP-
	Eimeria acervulina	XP_013248988	S----------EG--K-HK---IEV		A---E----A--CRI-N--A--
	Eimeria brunetti	CDJ51888	S-N--------EG--K-HK---IEV		AN--E----A--CRI-S--VP-
	Eimeria maxima	XP_013335029	S----E----IKG--K-NK---IEV		-N--E--EY---CRI-N--LP-
	Eimeria praecox	CDI87473	S----------EG--K-HK---IEV		A-RLE----A--CRI-S--AP-
	Eimeria tenella	XP_013228879	S----------EG--K-HG---IEV		A---E----A--CRI-N--A--
	Neospora caninum Liv.	XP_003882281	S----------QG--K-HG---LDV		T---E--E-A--C----D-T--
	Toxoplasma gondii ME49	XP_002367765	S----------QG--K-HG---LDV		T---E--E-A--C----D-T--
	Cryptosporidium andersoni	OII77439	S-E-DC--Q--E---R-RN---ITV		P---E--EMA--C---R---P-
	Cryptosporidium muris RN66	XP_002142505	S-E-DC--Q--E---R-RN---ITV		P---E--EMA--C---R---P-
	Cryptosporidium parvum Io. II	XP_625539	S-E-DC--Q--E---R-RG---I-V		P---E--EMA--C---R---P-
	Cryptosporidium ubiquitum	OII75545	S-E-DC--Q--E---R-RG---I-V		P---E--EMA--C---R---P-
Other Eukarya	*Thecamonas trahens ATCC 50062*	XP_013759960	N------T---S---N-H--Q-IQV		ESN-E--L-A--C-L-A--QP-
	Plasmodiophora brassicae	CEO99081	T-D-A------K-M-EQRK---I-V		D------E-A--C-I-AD-K
	Monoraphidium neglectum	XP_013903254	D-NQ-D----IE-----HNVN-IT-		PEN-Q--E-A--C-L-A--T--
	Symbiodinium microadriaticum	OLP85606	S-N-AT----IQG--V--NV-VIDV		P-N-S--E-A--C-I--D-MP-
	Pneumocystis carinii B80	XP_018227557	S-D-E--T--IE-----HN-N-IKV		S---K--E-A--CTL-R-----
	Ceratocystis fimbriata CBS 114	PHH50613	A-E-E------I---S-HN---IKV		P-G-E--E-A--CVL-R-----
	Pneumocystis murina B123	XP_007873101	S-D-E--T---E-----HN-N-VKV		S---K--E-A--CTL-R-----
	Ceratocystis platani	KKF93096	A-E-E------I---S-HN---IKV		P-G-E--E-A--CVL-R-----
	Pneumocystis jirovecii RU7	XP_018231440	S-D-E--T---E-----HN-N-IKV		A---K--E-A--CTL-R-----
	Blumeria graminis f. sp. Hor.	AAC15802	A-E-E-----IV---S-HK---IKV		P-G-Q--E-A--CVL-R-----
	Pyronema omphalodes CBS 100304	CCX05047	N-D-E------E-----HN---IKV		A-G-K--E-A--CVL-R-----
	Aschersonia aleyrodis RCEF 249	KZZ97634	K-E-D------AG--K-HN---IKV		Q-G-Q--E-A--CVL-R-----
	Aureobasidium subglaciale EXF-	XP_013340810	A-E-E-----IV---G-HK---IKV		P-G-Q--E-A--CQI-R-----
	Coniosporium apollinis CBS 100	XP_007778769	A-E-E------V---S-HK---IKV		P-G-Q--E-A--CQI-R-----
	Cordyceps militaris CM01	XP_006666683	N-E-D------A---S-HK---IKV		Q-G-Q--E-A--CVL-R-----
	Diplodia seriata	OMP87059	A-E-E------V---S-HK---IKV		P-G-Q--E-A--CQI-R-----
	Isaria fumosorosea ARS. 2679	XP_018705383	N-E-D------A---S-HK---IKV		Q-G-Q--E-A--CVL-R-----
	Nectria haematococca mpVI 77-1	XP_003052279	N-E-E------V---N-HN---IQ-		P-G-Q--E-A--CVL-R-----
	Beauveria bassiana ARS. 2860	XP_008594417	N-E-D------A---S-HK---IKV		Q-G-Q--E-A--CVL-R-----

Figure S3. A partial sequence alignment of the 40S ribosomal protein S12 showing a one amino acid insertion that is specific for members of *Hematozoa*.

Group	Species	Accession	4		59
**Hematozoa** – ***Plasmodium* (20/20)**	*Plasmodium berghei ANKA*	CDS50559	LIGLKGKDFIILGADTYSINSIIKLK	N	DDKTKFYDINGNKCLLLGGSIGDRIQFGE
	Plasmodium chabaudi cha.	XP_016654619	--------------------------	-	-----------------------------
	Plasmodium coatneyi	XP_019916811	-------N----A-----V-------	-	-----------------------------
	Plasmodium cynomolgi str. B	XP_004223822	-------N----AV----V-------	-	-----------------------------
	Plasmodium falciparum 3D7	XP_001348850	----R-NN-VV-A-------------	-	--N------H--------------L----
	Plasmodium falciparum CAMP/Mal.	ETW58701	----R-NN-VV-A-------------	-	--N------H--------------L----
	Plasmodium falciparum HB3	KOB58315	----R-NN-VV-A-------------	-	--N------H--------------L----
	Plasmodium falciparum IGH-CR14	KNG76549	----R-NN-VV-A-------------	-	--N------H--------------L----
	Plasmodium falciparum Tan.	ETW33566	----R-NN-VV-A-------------	-	--N------H--------------L----
	Plasmodium fragile	XP_012333514	-------N----A-----V-------	-	-----------------------------
	Plasmodium gaboni	XP_018638827	----R-NN-V--A-------------	-	N-N------K-------------------
	Plasmodium gallinaceum	CRG94907	-------N-V--A---------V---	-	-----------------------------
	Plasmodium gonderi	GAW82376	-------N----A-----V-------	-	-----------------------K-----
	Plasmodium inui San Ant. 1	XP_008813913	-------N-L--A---H---------	-	-----------------------------
	Plasmodium knowlesi str. H	XP_002260154	-------N----A-----V-------	-	-----------------------------
	Plasmodium malariae	SBS88291	----------V-A-------------	-	-----------------------------
	Plasmodium ovale curt.	SBS86295	----------V---------------	-	--N--------------------------
	Plasmodium ovale wal.	SBT41727	----------V---------------	-	--N--------------------------
	Plasmodium relictum	CRH01214	-------N-V--A---------V---	-	-----------------------------
	Plasmodium vinckei pet.	EUD70425	--------------------------	-	-----------------------------
	Plasmodium vinckei vin.	XP_008622261	--------------------------	-	-----------------------------
	Plasmodium vivax Sal-1	XP_001615680	-------N----AV----V-------	-	-----------------------------
	Plasmodium yoelii yoe. 17XNL	XP_727351	--------------------------	-	-----------------------------
	Plasmodium reichenowi	XP_012765751.1	----R-NN-VV-A-------------	-	--N------H--------------L----
	Plasmodium sp. Gor. Cla. G2	SOV19909.1	----R-NN-VI-A-------------	-	--N------K--------------L----
	Plasmodium sp. DRC-Itaito	SPJ13069.1	----R-NN-VV-A-------------	-	--N-----MH--------------L----
**Hematozoa** – ***Piroplasmida* (8/8)**	*Theileria annulata*	XP_954530	---IC-R--VAVAT---EKS--VT--	-	--ES-IMQ-DDS-L---A-PS---M----
	Theileria equi	XP_004830061	---IC-P--VAIAS---EKY---T--	-	--DS-IMP-DNS-L---A-PM---A----
	Theileria orientalis str. Shi.	XP_009689007	---IR-R--VAVAT---EKYGV-TM-	-	--DG-IMQ-DDS-L---A-PL---S----
	Theileria parva str. Mug.	XP_765714	---IC-R--VAVAT---EKH--VT--	-	--DS-IMQ-DDS-L---A-PCC--N----
	Babesia bigemina	XP_012769925	---IR----VAVAC---EKY---TI-	-	--ES-ISQ-GKSTV-M-A-PL---VHW--
	Babesia bovis T2Bo	XP_001609094	---I--P--VAIAC---EKY---TI-	-	--ES-IMK-GDS-VMM-A-PL---S--SK
	Babesia microti str. RI	XP_012647857	---I----Y-L-A--C-EQY---RTN	F	Y-HG-ILKVDND-LI--A-PS---E----
	Babesia sp. Xinjiang	ORM40607	--AI--E--LAIAC---EKY---TIR	-	--ES-IMK-GQS-VIM-A-PM---A--SN
	Babesia ovata	GBE62193.1	---IR----VAVAC---EKY------	-	--ES-ISK-GKSTV-----PL---VHW--
**Other Apicomplexa**	*Besnoitia besnoiti*	PFH32590	V---R-PT-ALVAC-R-ANS--LRM-		--ED-VLLVDD--IMG-A-Q----L---D
	Cystoisospora suis	PHJ14908	----Q-PT-ALVAC-K-AHS--LRM-		S-DD-LLVVDN--V-GMA-Q----L---D
	Eimeria necatrix	XP_013438006	----CCRE-AVVA--K--S---VRM-		S-ED-LLLVDDD-L-A-A-EP---T----
	Eimeria tenella	XP_013236302	----CCRE-AVVA--K--SS--VRM-		S-ED-LLQVDDD-L-A-A-EP---S----
	Hammondia hammondi	XP_008885117	V--IR-R--ALVAC-R-ANS--LRM-		--ED-LLLVDDD-VMGFA-Q----L----
	Neospora caninum Liv.	XP_003880481	V--IR-RT-ALVAC-R-ANS--LRM-		--ED-LMLVDD--VMG-A-Q----L---D
	Toxoplasma gondii ME49	XP_002369544	V--IR-R--ALVAC-R-ANS--LRM-		--ED-LLLVDDD-VMGFA-Q---L---D
	Cryptosporidium andersoni	OII75686	---I--P--V-IA--ST--SG-SRI-		H-ED-ILS-D---LIATA-D----N----
	Cryptosporidium hominis	CUV03983	---I--P--VA-AS--VAFSNVFR-S		LKDD-IME-DE--LIGV--TL---M----
	Cryptosporidium muris RN66	XP_002141473	---I--P--V-IA--ST--SG-SRI-		H-ED-ILS-D---LIATA-D----N----
	Cryptosporidium parvum Iow. II	XP_001388136	---I--P--VA-AS--VAFSNVFR-S		LKDD-IME-DE--LIGV--TL---M----
	Cryptosporidium ubiquitum	OII74485	---I--Q--VV-AS--VAVSNVFR-S		LKDD-IME-DE--LIGI--TL---M----
	Gregarina niphandrodes	XP_011133207	V--I-----VLCA--KS-AY---R-V		-TQD-VCELD---LMACA-PDA--QN-MD
**Other Eukarya**	*Osmerus mordax*	ACO09331	---IQ-T--VLVA--NVAAS---QM-		H-YD-MFKLSEKIL--CV-EA--TV--A-
	Trematomus bernacchii	ALU10982	-V-IQ-PN-VLVA--NVAAS--LQM-		H-QD-MFKLSEKIL--CV-EA--TA--A-
	Scylla paramamosain	ACY66487	---M-FN--VLMAT-MSQLQR-MVQ-		S-DD-M-QLSSHLVMAVA-EA--TT--A-
	Oncorhynchus mykiss	ACO07896	---IQ-Q--VLVA--NVAAH--V-M-		Q-QD-MFKLSDKIL--CV-EA--TV--A-
	Salmo salar	ACI68242	---IQ-Q--VLVA--NVAAH--V-M-		Q-QD-MFKLSDKIL--CV-EA--TV--A-
	Rana catesbeiana	ACO52088	---VQ-S-YVMVA---VCA-----M-		HEVD-MFKMSEKIL--CV-EA--TV--A-
	Clunio marinus	CRK93992	IV-I-----VM-A--SSHPH--MI--		--EN-I-K-SDSLMIAAV-DS--TV--V-
	Aedes albopictus	KXJ82419	-M-IR-P--VM-A--CTHAH--MV--		--ES-I-K-SD-LMMATI-EA---V--T-
	Ictalurus punctatus	NP_001188165	---IQ-Q--VLVA--NVAAS----M-		H-YD-MFKLSEKIL--CV-EA--TV--A-
	Xenopus laevis	NP_001084761	---IQ-N--VLVA---VCA----QM-		H-MD-MFKMSEKIL--CV-EA--TV--A-
	Patagioenas fasciata monilis	OPJ71769	---IQ-P-YVLVA---VAAT--VQM-		H-HD-MFKMSEKIL--CV-EA--TV--A-
	Colinus virginianus	OXB71456	I-----PT-AM-AC-R--TS---RMQ		-TSD-ILLVDD--L-G-S-E----L----
	Xenopus tropicalis	OCA44663	IQ-N--VLVA---VCA-----M-		H-VD-MFKMSEKIL--CV-EA--TV--A-
	Gossypium arboreum	KHG19313	VF--V-N--A-VA---SAVH--LVH-		SNED-IMILDSH-LIAAS-ES---V--T-
	Arabis alpina	KFK39554	VF--V-NG-V-VA---SAVH--LLH-		NNED-IMVLDSH-LVAAS-EP---V--T-

Figure S4. A partial sequence alignment of the 20S proteasome beta 4 subunit protein showing a one amino acid insertion that is specific for members of *Hematozoa*.

		Species	Accession	90		136
Hematozoa	***Piroplasmida* (8/8)**	*Babesia bigemina*	XP_012769739	WDGVGLLGLSVRFAEFTAMDE		GAHVLKVHEGSPASKAGLIPITDYLL
		Babesia ovata	GBE62369.1	---------------------		----I---------------------
		Babesia bovis T2Bo	XP_001610686	---------------------		----IN--D----K----M-------
		Babesia microti str. RI	XP_012649138	-H-E----AR-IYD--N-LFQ		-NR--A-VPN--------VATS--II
		Babesia sp. Xin.	ORM40602	---------------------		----IN-QD----M----T-------
		Theileria annulata	XP_954941	---I-----TLK-GL-SCL--		---IVH-YDK---Q----M-------
		Theileria equi	XP_004832439	-E-------T-K-S---S---		-V--VN-YD---------M-V-----
		Theileria orientalis str. Shi	XP_009691633	---I-F---TLK-GL-NCL--		---IVH-YDK---Q----M-------
		Theileria parva str. Mug.	XP_763267	---I-----TLK-GL-NCL--		---IVH-YDK---Q----M-------
	***Plasmodium* (20/20)**	*Plasmodium gallinaceum*	CRG97184	-K-N-----NINYEFLN--N-		-TRI-DIF-N---F-SD-NEYS-FII
		Plasmodium malariae	SBS99989	-K-N----INLSYESSN--N-		-VR--EIF-----F-SN--EYS--II
		Plasmodium ovale curt.	SBS81364	-H-K----ININYESMN--N-		-IRI-EICKD---F-SS-VEYD--II
		Plasmodium ovale wal.	SBT32611	-Q-K----ININYESMN--N-		-IRI-EICKD---F-SR-VEYD--II
		Plasmodium relictum	CRG99032	-K-N----ININ-EFLN--N-		--RI-DIF-N---F-SN-NEYS-FII
		Plasmodium vivax Ind. VII	KMZ81664.1	-R-K----MNISYETAN--K-		-VEIAN-EDVYVDVRSKVTEKE-LII
		Plasmodium fragile	XP_012337202.1	-S-K----MNISYEKANT-K-		-IEIVSIED-YSDV-NRIAQEE-VII
		Plasmodium knowlesi	OTN68369.1	-R-K----MNISYEKANT-K-		-IQIVRIED-YLDV-NKVTEKE-III
		Plasmodium cynomolgi str. B	XP_004221331.1	-R-K----MNISYEKAN--K-		-IQIVRIED-YLDI-NKITEKE-III
		Plasmodium coatneyi	XP_019913667.1	-R-K----MNISYEKANT-N-		-IQIVRIED-YLDV-NKVTEKE-III
		Plasmodium inui San Ant. 1	XP_008818474.1	-S-K----MNISYEKAN--E-		-IQIVSIED-HSDVRSKVTEKE-III
		Plasmodium falciparum RAJ116	KNC36072.1	-E-N----IHISYEFLN-LN-		-VRI-EIL-N---YQSQ-IEYE-FII
		Plasmodium sp. DRC-Itaito	SPJ10584.1	-E-N----IHISYEFLN-LN-		-VRI-EIL-N---YQSQ-IEYE-FII
		Plasmodium reichenowi	XP_019970416.1	-E-N----IHISYEFLN-LN-		-VRI-EIL-N---YQSQ-IEYE-FII
		Plasmodium gaboni	SOV14829.1	-E-N----IHISYELLN-LN-		-VRI-EIL-N---YESQ-IEYE-FII
		Plasmodium sp. Gor. Cla. G2	SOV14852.1	-E-N----IHISYELLN-LN-		-VRI-EIL-N---YESQ-IEYE-FII
		Plasmodium chabaudi ada.	SCM07114.1	-S-N----ININYER-N-LY-		-VRI--TFNN-SDFECN-MEHS--II
		Plasmodium berghei ANKA	XP_022712235.1	-S-N----ININYER-N-LY-		-VRI--PFNN-SDFECN-MEHS--II
		Plasmodium yoelii 17X	ETB60825.1	-S-N----ININYER-N-LY-		-VKI--TFNN-SDFESN-MENS--II
		Plasmodium vinckei pet.	EUD69835.1	-S-N----ININYER-N-LY-		-VRI--TFNNPSDFECN-MEHS--II
		Plasmodium gonderi	GAW79875.1	-K-N----MNISYELIN--N-		-IQI--IDDKYSEI-RR--EKD-FII
**Other Eukarya**		*Thalassiosira pseudonana CCMP1*	XP_002295892	-G-A----VTI-LDNYGGA--	R	LIR--S-EHN---AI---V-ES----
		Symbiodinium microadriaticum	OLP86543	-G-A----AV--YDTLESAEG	Q	-MR--S-FPN---EA---V-NK-F--
		Schistosoma japonicum	AAX26880	-G-Q--M-V-I-YCS-DRAN-	N	VW---S-EPK---DL---K-H---II
		Lepeophtheirus salmonis	ACO12058	-G-M----I-I--CS-EGAY-	H	VW-I-E--P----EA---RSY---IV
		Tetraodon nigroviridis	CAF97646	-G-Q----V-I--CS-EGAN-	N	VW---E-EPN---AL---R-H---II
		Oikopleura dioica	CBY22133	-G-Q----V-I--CS-EGAN-	N	VW---E-ESN---YL---R-F---VI
		Echinococcus multilocularis	CDI98000	-G-Q------I-YCS-KGAS-	N	VW---D-SP----AM---Q-FS--II
		Oncorhynchus mykiss	CDQ83904	-G-Q----V-I--CS-EGAN-	N	IW---E-EPN---AL---R-H---II
		Echinococcus granulosus	CDS21409	-G-Q------I-YCS-KGAS-	N	VW---D-SP----AM---Q-FS--II
		Myotis davidii	ELK28205	-G-Q----V-I--CS-DGAN-	N	VW---E-ESN---AR---R-HS--II
		Capitella teleta	ELU00246	-G-Q----V-I--CS-EGAN-	N	VW-IMD-QNN----L----SN---II
		Myotis brandtii	EPQ02293	-G-Q----V-I--CS-DGAN-	N	VW---E-ESN---AR---R-HS--II
		Anopheles darlingi	ETN63211	-G-Q----V-I--CS-EGAN-	N	VW-I-EI-PS---EE-----F---II
		Clonorchis sinensis	GAA31000	-G-Q----V-I-YCS-DGAN-	N	VW---E-EPN---QQ-----HS--II
		Anopheles sinensis	KFB35905	-G-Q----V-I--CS-EGAN-	N	VW-I-E--PS---EE-----F---II
		Calypte anna	KFO95174	-G-Q----A----CS-QGAN-	H	VW---D-EP----AL---Q-Y---VV
		Eurypyga helias	KFV95693	-G-Q----A----CS-KGAN-	H	VW---D-EPA---AL---Q-Y---VV
		Drosophila melanogaster	NP_649160	-G-Q----V-I--CS-EGAN-	S	VW-I-E--PN---EL---RAYS--VI
		Opisthorchis viverrini	OON21089	-G-Q----V-I-YCS-DGAN-	N	VW---E-EPN---QQ-----HS--II
		Hypsibius dujardini	OQV19098	-G-Q----V-I--CS-EGANQ	N	VW---D--P-----A-Q-R-YS---I
		Danaus plexippus ple.	OWR49492	-G-Q----V-I--CS-EGAN-	N	VW---E--PS---EL---R-FS--II
		Trichomalopsis sarcophagae	OXU17945	-G-Q----V-I--CS-EGAN-	N	VW---E--PS---EL---R-F---II
		Heliothis virescens	PCG76178	-G-Q----V-I--CS-EGAN-	N	VW---E--PT---EL---R-FS--II
		Monodelphis domestica	XP_001367823	-G-Q----V-I--CS-DGAN-	N	VW---E-ESN---AL---R-YS--II
		Amphimedon queenslandica	XP_003387709	-G-Q----V-I--CS-EGAS-	N	VW---E-QPD---AV-----H---I-
		Bombus terrestris	XP_003396383	-G-Q----V----CS-EGSN-	N	VW---E--PS---EL---R-F---II
		Bombus impatiens	XP_003491728	-G-Q----V----CS-EGSN-	N	VW-I-E--PS---EL---R-F---II
		Apis dorsata	XP_006612595	-G-Q----V----CS-EGSN-	N	VW---E-YPS---EL---R-F---II
		Lepisosteus oculatus	XP_006636658	-G-Q----V-I--CS-EGAN-	N	VW---E-EPN---AL---R-H---II
		Astyanax mexicanus	XP_007244204	-G-Q----V-I--CS-EGAN-	N	VW---E-ESN---AL---R-F---II
		Poecilia formosa	XP_007540277	-G-Q----V-I--CS-EGAN-	N	VW---E-EPN---AL---R-H---II

Figure S5. A partial sequence alignment of the Golgi reassembly-stacking protein 1 showing a one amino acid deletion that is specific for members of *Hematozoa*.

Group	Species	Accession	461				515
Hematozoa: Plasmodium (20/20)	*Plasmodium berghei str. ANKA*	XP_680419	MIARGDLGIETNLSN		LPILQKKIINLCKTKY	N	KIVIVATQMLESMRFIPSPTRAE
	Plasmodium sp. Gor. Cla. G2	SOV15248.1	---------------		-------L----RI--	-	-P-------M-----L-------
	Plasmodium sp. DRC-Itaito	SPJ10778.1	---------------		-------L----RI--	-	-P-------M-----L-------
	Plasmodium chabaudi ada.	SCM03350	---------------		----------------	-	-----------------------
	Plasmodium chabaudi cha.	XP_742288	---------------		----------------	-	-----------------------
	Plasmodium coatneyi	XP_019913647	---------------		-------M----RI--	-	-P-------M-----L-------
	Plasmodium cynomolgi str. B	XP_004221522	---------------		-------M----RI--	-	-P-------M-----L-------
	Plasmodium falciparum 3D7	XP_001347647	---------------		-------L----RI--	-	-P-------M-----L-------
	Plasmodium falciparum IGH-CR14	KNG77873	---------------		-------L----RI--	-	-P-------M-----L-------
	Plasmodium falciparum RAJ116	KNC36165	---------------		-------L----RI--	-	-P-------M-----L-------
	Plasmodium falciparum Vie.	ETW18005	---------------		-------L----RI--	-	-P-------M-----L-------
	Plasmodium fragile	XP_012336820	---------------		-------L----RI--	-	-P-------M-----L-------
	Plasmodium gaboni	XP_018641747	---------------		-------L----RI--	-	-P-------M-----L-------
	Plasmodium gallinaceum	CRG97378	---------------		------------RI--	-	-P-------------L-------
	Plasmodium gonderi	GAW80064	---------------		------------RI--	-	-P-------M-----L-------
	Plasmodium inui San Ant. 1	XP_008818904	---------------		-------M----RL--	-	-P-------M-----L-------
	Plasmodium knowlesi str. H	XP_002261887	---------------		------------RI--	-	-P-------M-----L-------
	Plasmodium malariae	SBS83470	-----------D---		----------S-RI--	-	-P-------M-----L-T-----
	Plasmodium ovale curt.	SBS81758	---------------		------------RI--	H	-P-------------L-------
	Plasmodium ovale wal.	SBT33084	---------------		------------RI--	H	-P-------------L-------
	Plasmodium reichenowi	CDO64799	---------------		-------L----RI--	-	-P-------M-----L-------
	Plasmodium relictum	CRG99222	---------------		------------RI--	-	-P-------------L-------
	Plasmodium vinckei pet.	EUD71517	---------------		----------------	-	-----------------------
	Plasmodium vinckei vin.	XP_008625798	---------------		----------------	-	-----------------------
	Plasmodium vivax Bra. I	KMZ87830	---------------		-------M----RI--	-	-P-------M-----L-------
	Plasmodium vivax Ind. VII	KMZ81613	---------------		-------M----RI--	-	-P-------M-----L-------
	Plasmodium vivax Sal-1	XP_001608406	---------------		-------M----RI--	-	-P-------M-----L-------
	Plasmodium yoelii 17X	ETB58817	---------------		----------------	-	-----------------------
	Plasmodium yoelii yoe. 17XNL	XP_724095	---------------		----------------	-	-----------------------
Hematozoa: Piroplasmida (8/8)	*Babesia bigemina*	XP_012766077	--------V--EIT-		---I--RLVQ--RLV-	R	-P------------TN-K-----
	Babesia bovis	BAN65196	--------V--EIT-		--VI--RL-Q--RLV-	H	-P-----------KSN-K-S---
	Babesia sp. Xin.	ORM41826	--------V--E-T-		--VT--RLVQ--RLV-	H	-P---------T--KN-K-----
	Babesia microti strain RI	XP_012647591.1	--------V-ID-TS		IAAV--RLAHISRFVH	R	-PC----------IKN-M-----
	Babesia ovata	GBE60932.1	--------V--EIT-		---I--RLVQ--RLV-	R	-P------------SN-K-----
	Theileria annulata	XP_952576	-V---------D-A-		---V--RL-Q--RVV-	R	-PC--------T--SS-T-----
	Theileria equi	XP_004828683	-V---------E-A-		---I--RLVQ--RLV-	H	-PC--------T--SSLT-----
	Theileria orientalis str. Shi.	XP_009690152	-V---------D-A-		---V--RL-Q--RVV-	R	-PC--------T--SS-M-----
	Theileria parva str. Mug.	XP_764703	-V---------D-A-		---V--RL-Q--RVV-	R	-PC--------T--SS-T-----
Toxoplasma and related (0/11)	*Neospora caninum Liv.*	401399225	-V------V-LPNIA	W	--RV--RLVH--RKAG		-P-V---------MRA-L-----
	Hammondia hammondi	675126514	-V------V-LPNIA	W	--RV--RLVS--REAG		-P-V---------MQA-L-----
	Toxoplasma gondii ARI	1005153326	-V------V-LPNIA	W	--RV--RLVS--REAG		-P-V---------MQA-L-----
	Toxoplasma gondii TgC.	1008949693	-V------V-LPNIA	W	--RV--RLVS--REAG		-P-V---------MQA-L-----
	Toxoplasma gondii MAS	672577182	-V------V-LPNIA	W	--RV--RLVS--REAG		-P-V---------MQA-L-----
	Toxoplasma gondii p89	672274670	-V------V-LPNIA	W	--RV--RLVS--REAG		-P-V---------MQA-L-----
	Toxoplasma gondii ME49	237844955	-V------V-LPNIA	W	--RV--RLVS--REAG		-P-V---------MQA-L-----
	Toxoplasma gondii COUG	1275371326	-V------V-LPNIA	W	--RV--RLVS--REAG		-P-V---------MQA-L-----
	Toxoplasma gondii VAND	672566767	-V------V-LPNIA	W	--RV--RLVS--REAG		-P-V---------MQA-L-----
	Toxoplasma gondii RUB	672295070	-V------V-LPNIA	W	--RV--RLVS--REAG		-P-V---------MQA-L-----
	Toxoplasma gondii GT1	523572106	-V------V-LPNIA	W	--RV--RLVS--REAG		-P-V---------MQA-L-----
Proteobacteria	*Acetobacter sp. CAG:977*	CCZ21445	-V------V--PPEK		V-V-----VQA-RKAN		-P-----------VVN-T-----
	Pelagibacterium sp. SCN 63-126	ODT48952	-V------V-LP-ET		V-G---RM-RM-RRYG		-P-----------ITA-V-----
	Pelagibacterium sp. SCN 63-23	ODT67986	-V------V-LP-ET		V-G---RM-RM-RRYG		-P-----------ITA-V-----
	Azospirillum sp. 51_20	OLA81250	-V------V-CP-QT		V-V-----V-A-RKYA		RP-----------IEK-T-----
	Kiloniella laminariae	WP_020590773	-V------V-LPPE-		V-V---R--RI-REYG		-P---------T-VTS-T-----
	Poseidonocella sedimentorum	WP_092079743	-V------V-MP-Q-		V-TI--RLVRM-RKAG		-P-V---------IES-M-----
	Azospirillum sp. CAG:260	CDB40855	-V------V-CPIQT		V-VM----VAA-RKYG		RP--I--------IKA-T-----
	Azospirillum sp. CAG:239	CDB52778	-V------V-CP-PT		V-V---R-VTA-RKYA		RP--I--------IKA-T-----
	Pseudomonas aeruginosa	OOD00188	-V------V-VPAES		V-GI--R-VQV-RQLG		-P-V-----------S-A-----
	Pseudomonadales bacterium 32-6	OYW90632	-V------V-VPAE-		V-RI--N-VRT-RQLG		RP-V-----------S-A-----
	Enterobacter cloacae	SAJ32814	-V------V-VPAES		V-GI--R-VQV-RQLG		-P-V-----------S-A-----
	Pseudomonas stutzeri	WP_003285104	-V------V-VPPE-		V-RI--N-VRI-RQLG		RP-V-----------A-A-----
	Pseudomonas sp. GM60	WP_008026782	-V------V-VPAES		V-QI--N--SI-RQLG		-P-V-----------S-A-----
	Marinobacterium rhizophilum	WP_020682653	--------V-VPAE-		V-GI--S--KC-RQLG		RP-V-----------S-A-----

Figure S6. A partial sequence alignment of the Pyruvate Kinase 2 protein showing a one amino acid insertion that is specific for members of *Hematozoa* and another one amino acid insertion that is specific for *Toxoplasma* and related species.

Group	Species	Accession	273		315
Piroplasmida (8/8)	*Theileria equi*	XP_004833199	DDNAKYRQTEIFEKSPAN		LTHEEAEADKVGLNYISLDGNVACI
	Babesia ovata	GBE60307.1	----DF--KD--ASVAPP		ENEL--R-VEA-------G------
	Theileria annulata	XP_954105	--------K-L-DMT-VT		R-R--E---RA--------------
	Theileria parva str. Mug.	XP_766198	--------S-L-DMT-VI		R-K--E---RA--------------
	Theileria orientalis str. Shi.	XP_009689511	--------Q-V-AAGCEA		R-R--S--E-AD-------------
	Babesia bovis T2Bo	XP_001610970	----DF--K----SV-PA		ENEL--R-NE--------G--I---
	Babesia microti str. RI	XP_012649299	----DF--D-Y-KNA-MP		VNPA-LA-KNA-V------------
	Babesia sp. Xin.	ORM39443	----DF--R---ASV-PP		DNEL--R-SEA----V--G--I---
	Babesia bigemina	XP_012766706	----DF--KD--ASVAPP		ENEL--R-LEA-----P-G------
	Babesia divergens	LK934712.1	----E---K---ASA--P		ENAQ--R-AEA-----A-G------
Plasmodium (0/20)	*Plasmodium berghei str. ANKA*	XP_677913	----E---K-----RDLT	Q	ENP--L--K-YN---V-----I--M
	Plasmodium sp. DRC-Itaito	SOV25039.1	----E---K-----RDLT	Q	ENEQ-LL-K-FN---V--N--I--M
	Plasmodium sp. Gor. Cla. G2	SOV19117.1	----E---K-----RDLT	Q	ENKQ-LL-K-FN---V--N--I--M
	Plasmodium chabaudi ada.	SCM07089	----E---K---D-RDLT	Q	ENP--L--K-YN---V-----I--M
	Plasmodium chabaudi cha.	CAH78828	----E---K---D-RDLT	Q	ENP--L--K-YN---V-----I--M
	Plasmodium coatneyi	XP_019917198	----E---K---QQRDLS	Q	ENA--I--K-FN---V-----I--M
	Plasmodium cynomolgi str. B	XP_004224344	----E---K-V-QQRDLS	Q	ENA--IQ-K-FN---V-----I--M
	Plasmodium falciparum 3D7	XP_001348469	----E---KN----RDLT	Q	ENK--LL-K-YN---V--N--I--M
	Plasmodium falciparum FCH/4	ETW28747	----E---KN----RDLT	Q	ENK--LL-K-YN---V--N--I--M
	Plasmodium falciparum IGH-CR14	KNG74582	----E---KN----RDLT	Q	ENK--LL-K-YN---V--N--I--M
	Plasmodium falciparum Tan.	ETW34024	----E---KN----RDLT	Q	ENK--LL-K-YN---V--N--I--M
	Plasmodium fragile	XP_012337586	----E---K----QRDLS	Q	EHA--IQ-K-FN---V-----I--M
	Plasmodium gaboni	XP_018639379	----E---K-----RDLT	Q	ENEQ-LL-K-FN---V--N--I--M
	Plasmodium gallinaceum	BAM15613	----EF--K-----RDIS	Q	ENA--M--K-YN---V-----I--M
	Plasmodium gonderi	GAW82919	---SE---K---Q-RDLS	Q	ENP--IQ-K-FN---V-----I--M
	Plasmodium inui San Ant. 1	XP_008816897	----E---K---QQRDLS	Q	ENA--V--K-FN---V-----I--M
	Plasmodium knowlesi str. H	XP_002260707	----E---K---LQRDLS	Q	ENA--IQ-K-FN---V-----I--M
	Plasmodium malariae	SBS89466	----E---K---Q-RDLS	Q	EDSI-IQ-R-HN---V--N--I--M
	Plasmodium ovale curt.	SBS87272	----E---K---KNRDLS	Q	ENV--I--R-YN---V-----I--M
	Plasmodium ovale wal.	SBT30454	----E---K---KNRDLS	Q	ENS--I--R-YN---V-----I--M
	Plasmodium reichenowi	XP_012765363	----E----N----RDLT	Q	ENK--LL-K-YN---V--N--I--M
	Plasmodium relictum	CRH03836	----E---K-----RDIS	Q	EDS--M--K-YN---V-----I--M
	Plasmodium vinckei pet.	EUD73888	----E---K---D-RDLT	Q	ENP--L--K-YN---V-----I--M
	Plasmodium vinckei vin.	XP_008623854	----E---K---D-RDLT	Q	ENP--L--K-YN---V-----I--M
	Plasmodium vivax Ind. VII	KMZ78534	----E---K---Q-RDLS	Q	ENA--IQ-K-FN---V-----I--M
	Plasmodium vivax Sal-1	XP_001616658	----E---K---Q-RDLS	Q	ENA--IQ-K-FN---V-----I--M
	Plasmodium yoelii yoe. 17XNL	XP_730040	----E---K-----RDLT	Q	ENP--L--K-YN---V-----I--M
Other Apicomplexa	*Cyclospora cayetanensis*	1249162734	----QF--KAL--QRDTS	Q	ENSG-L--E-YN----K---QIG-M
	Besnoitia besnoiti	1261480414	----AF--KDV-AQRDYS	Q	EDPR-VA--AAD----G---SIG-M
	Cystoisospora suis	1268238776	----AF--KD--SQRDFT	Q	EDPR-V----AN----G---SIG-V
	Hammondia hammondi	675129222	----SF--KD--SQRDYS	Q	EDPR-VA--AAD----G-E-SIG-M
	Neospora caninum Liv.	401411083	----AF--KDV-AQRDYS	Q	EDPR-VA--AAD----G---SIG-M
	Toxoplasma gondii ME49	237829849	----GF--KD--SQRDYS	Q	EDPR-VA--AAD----G-E-SIG-M
	Cryptosporidium muris RN66	XP_002141262	----S---K-L-DIEDVE	Q	KDQI-V--A-Y-----R-N---G--
	Cryptosporidium andersoni	1098427798	----S---K-L-DIEDVE	Q	KDQI-V--A-Y-----R-N---G--
	Gregarina niphandrodes	749158289	----FF--K-L-AQEDTS	V	RCPR-LA-EAEHM--V-M----G-L
Other Eukarya	*Vitrella brassicaformis CCMP.*	CEL94328	--S-----Q-L-SMEDES	L	MDPR--A-H-A-----G----IG-L
	Perkinsus marinus ATCC 50983	XP_002783211	----QF--KD--SQRDIT	Q	EDPR-VA-S-WD----G----IG-M
	Thecamonas trahens ATCC 50062	XP_013755650	----SF--AD--GLEDKS	Q	VDPR-V--T-YD----G-------L
	Thalassiosira pseudonana CCMP.	XP_002286405	----EF--KS---RRDFS	Q	EDPR-V--S-YD----G----IG-M
	Salpingoeca rosetta	XP_004996763	----AF--Q---AMRDTT	E	EDPRDV----H-----GM---I--L
	Paramecium tetraurelia str.	XP_001440162	---SQF--HA--HMEDVR	Q	KDWK-V--Q-H-----A----IG-M
	Candida parapsilosis	CCE42831	----AF--Q-V-SWRDPT	Q	EDPQ----S-Y---F-K----I-N-
	Millerozyma farinosa CBS 7064	CCE82955	----SF--E-V-SWRDPT	Q	EDPQ----A-Y---F-K----I-N-
	Cyberlindnera fabianii	CDR37591	----S---E-V-NWRDLT	Q	EDPD-V--S-SN--F-K----IG-L
	Meyerozyma guilliermondii ATCC.	EDK39982	----SF--K-V-SWRDPT	Q	EDP--V--AEY---F-K----I-N-
	Pristionchus pacificus	PDM72854	----AF--K----MEDTS	D	KDPR-VA-N-LN----GM---I--M

Figure S7. A partial sequence alignment of a conserved region of the Succinyl-CoA synthetase beta chain protein showing a one amino acid deletion that is specific for members of *Piroplasmida* and some *Streptophyta* (not shown). The *Babesia divergens* sequence was acquired from a tblastn search conducted against the *Theileria equi* sequence. Expanded alignment of Figure 3 (A).

Group	Species	Accession	1856		1907
**Piroplasmida (8/8)**	*Theileria annulata*	XP_954617	RKVYLKSNVIPSLMEVWSLHDQNKYS	S	TKILRQVFRTLRKIVSDQNVQLMVK
	Babesia ovata	GBE62094.1	-RIF---DV-NTVVGI-DSY--Q--E	T	ARL---T--------AEGHLDI-L-
	Theileria equi	XP_004830195	-RI--N-D--NVVVKI-DAY-AKE-T	A	LRL---I--------AEEHLDI-LR
	Theileria orientalis str. Sh.	XP_009688917	------T-------D----YE-G---	N	Q--------S---V--EE-----M-
	Theileria parva str. Mug.	XP_765619	--------------------------	-	-------------------------
	Babesia bigemina	XP_012770027	-RI----D--NTVVD--DAY--H--E	T	PRL---T--------AEGHLDI-L-
	Babesia bovis T2Bo	XP_001609236	-RI--NAD--NV-VDI-DAY-KQ--T	M	SRL---T--------AEAH-DI-L-
	Babesia sp. Xin.	ORM40400	-RI----D--HVVVS--NAY-DK--V	M	-R----T--------AEGHLDI-L-
	Babesia microti str. RI	XP_012648621	--I-T-TSIVVTSIKI-NDY-KKI--	K	IRLM--L---I-NV-NETHLEALI-
	Babesia divergens	LK934714.1	-RI--N-D--YTVVDI-NAY-DK--T	T	-RL---I--S-----AEGHID—L
**Plasmodium (0/20)**	*Plasmodium sp. DRC-Itaito*	SOV24650.1	-N--DKT-AVHY-IDI-NQY-NGD--		VLL--H---AM------AH--TLL-
	Plasmodium sp. Gor. Cla. G2	SOV18437.1	-N--D-T-AVHY-IDI-NQY-NGD--		VLL--H---AM------AH--TLL-
	Plasmodium berghei ANKA	XP_022713216.1	-I-I-NQY--GY-S		VSL--H---AM------FH--TLL
	Plasmodium chabaudi ada.	SCM22824	-N--DNT-A-RH-I-I-NQY--GY--		VSL--H---AM--V---FH--TLLD
	Plasmodium chabaudi cha.	CAH78409	-N--DNT-A-RH-I-I-NQY--GY--		VSL--H---AM--V---FH--TLLD
	Plasmodium coatneyi	XP_019915577	-N--DRT-AVNH-I-I-NQY-HGD--		VSL--H---AM------AH-EMLL-
	Plasmodium cynomolgi str. B	XP_004223114	-N--DRT-AVNH-I-I-NQY-NGD--		VSL--H---AM------AH-EMLL-
	Plasmodium falciparum 3D7	CAX64377	-N--D-T-AVHY-IDI-NQY-NGD--		VLL--H---SM------AH--TLL-
	Plasmodium falciparum 7G8	EUR65319	-N--D-T-AVHY-IDI-NQY-NGD--		VLL--H---SM------AH--TLL-
	Plasmodium falciparum Dd2	KOB88580	-N--D-T-AVHY-IDI-NQY-NGD--		VLL--H---SM------AH--TLL-
	Plasmodium falciparum FCH/4	ETW29368	-N--D-T-AVHY-IDI-NQY-NGD--		VLL--H---SM------AH--TLL-
	Plasmodium falciparum HB3	KOB61509	-N--D-T-AVHY-IDI-NQY-NGD--		VLL--H---SM------AH--TLL-
	Plasmodium falciparum Mal.	ETW47222	-N--D-T-AVHY-IDI-NQY-NGD--		VLL--H---SM------AH--TLL-
	Plasmodium falciparum NF135/5.	ETW40775	-N--D-T-AVHY-IDI-NQY-NGD--		VLL--H---SM------AH--TLL-
	Plasmodium falciparum Palo Alt.	ETW55403	-N--D-T-AVHY-IDI-NQY-NGD--		VLL--H---SM------AH--TLL-
	Plasmodium falciparum RAJ116	KNC35849	-N--D-T-AVHY-IDI-NQY-NGD--		VLL--H---SM------AH--TLL-
	Plasmodium falciparum Tan.	ETW34445	-N--D-T-AVHY-IDI-NQY-NGD--		VLL--H---SM------AH--TLL-
	Plasmodium falciparum Vie.	ETW16654	-N--D-T-AVHY-IDI-NQY-NGD--		VLL--H---SM------AH--TLL-
	Plasmodium fragile	XP_012335399	-N--DRT-AVNH-V-I-NQY-HGD--		VSV--H---AM------AH-EMLL-
	Plasmodium gaboni	XP_018638865	-N--D-T-AVHY-IDI-NQY-NGD--		VLL--H---AM------AH--TLL-
	Plasmodium gallinaceum	CRG93974	-N--D-T-AVHN-I-I-NDY-NGH-T		VLL--H---SM------AH--TLL-
	Plasmodium gonderi	GAW81639	-N--DET-A-NH-I-I-NQY-HGD--		VSL--H---AM------AH-DALL-
	Plasmodium inui San Ant. 1	XP_008814936	-N--D-T-AVSH-I---NQY-NGD--		VSL--H---AM------AH-DMLL-
	Plasmodium knowlesi str. H	SBO28033	-N--DRT-AVNH-I-I-NQY-HGD--		VSL--H---AM------AH-EMLL-
	Plasmodium malariae	SBS86760	-N--DNTKA-HY-IDI-NQY-HGD--		ISL--HT--AM--V---AH-ETLL-
	Plasmodium ovale curt.	SBS84940	-N--D-T-AVHH-I-I-NQY-HGD--		VSL--H---AM------AH--TLL-
	Plasmodium ovale wal.	SBT36970	-N--D-T-SVHH-I-I-NQY-HGD--		VSL--H---AM------AH--TLL-
	Plasmodium reichenowi	CDO66398	-N--D-T-AVHY-IDI-NQY-NGD--		VLL--H---SM------AH--TLL-
	Plasmodium relictum	CRH00742	-N--D-T-AVHN-I-I-NDY-KGN-T		VLL--H---SM------AH--TLL-
	Plasmodium vinckei pet.	EUD73490	-N--DNT-AVRH-I-I-NQY--GY--		VSL--H---AM--V---FH--TLLD
	Plasmodium vinckei vin.	XP_008624212	-N--DHT-AVRH-I-I-NQY--GY--		VSL--H---AM--V---SH--TLLD
	Plasmodium vivax Sal-1	XP_001616400	-N--DRT-AVNH-I-I-NQY-HGD--		VSL--H---AM------AH-ETLL-
	Plasmodium yoelii 17X	ETB61043	-NI-NNT-AVRH-I-I-NQY--GYF-		VLL--H---AM------FH--TLLD
	Plasmodium yoelii yoe. 17XNL	XP_729560	-NI-NNT-AVRH-I-I-NQY--GYF-		VLL--H---AM------FH--TLLD
**Other Apicomplexa**	*Besnoitia besnoiti*	PFH34228	-NL-N-TPIMTE-IKT-DDY-KGA-T		STL--H---AM-RV---AH-EELL-
	Cyclospora cayetanensis	XP_022592432	-NLFN-THAVSE-IRC-NDF-KGL-T		-TL--H---AM-RV---AH-DELL-
	Cystoisospora suis	PHJ24002	-NL-N-TPIMTE-IRT-DDY-KGQ-T		STL--H---AM-RV---AH-DELL-
	Eimeria acervulina	XP_013247597	-NL-N-T-AVSE-IRC-NDY-KGL--		-AL--H---AM-R----AH-DELL-
	Eimeria brunetti	CDJ52254	-NL-N-T-AVSE-IRC-NDY-KGM-T		-AL--H---AM-R----AH-DELL-
	Eimeria maxima	XP_013335988	-NL-N-T-AVSE-IRC-DDY-AGM-T		-AL--H---AM-R----AH-DELL-
	Eimeria mitis	CDJ35744	-NL-N-T-AVSE-IRC-NDY-KGM--		-AL--H---AM-R----AH-DELL-
	Eimeria necatrix	XP_013433318	-NL-N-T-AVSE-IRC-NDF-KGM--		-TL--H---AM-R----AH-DELL-
	Eimeria praecox	CDI85787	-NL-N-T-AVSE-IRC-NDY-KGM--		NAL--H---AM-R----AH-DELL-
	Eimeria tenella	XP_013229791	-NL-N-T-AVSE-IRC-NDF-KGM--		-TL--H---AM-R----AH-DELL-
	Hammondia hammondi	XP_008885764	-NL-N-TPIMTE-INS-NDY-KGA-T		-TL--F---AM-RV---AH-EELL-
	Neospora caninum Liv.	CEL69917	-NL-N-TPIMTE-ISS-NDY-KGM-T		-TV--HI--AM-RV---AH-EELL-
	Toxoplasma gondii FOU	KFG56040	-NL-N-TPIMTE-INS-NDY-KGA-T		-TL--F---AM-RV---AH-EELL-
	Cryptosporidium ubiquitum	OII73365	-QLFIGTSMLDI-LSI-EAY-NKH--		LN---H---A--R----EF-SALLN
	Gregarina niphandrodes	XP_011134446	-TYFNNTECMNA-VGL-DDY-GG---		VDL-K---K-M-R-IN-HW-AVLL-

Figure S8. A partial sequence alignment of a Conserved hypothetical protein showing a one amino acid insertion that is specific for members of *Piroplasmida*. The *Babesia divergens* sequence was acquired from a tblastn search conducted against the *Theileria annulata* sequence.

Group	Organism	Accession	415		465
**_Piroplasmida_ (7/8)**	*Theileria parva str. Mug.*	XP_763245	FMANSRAKIYNESDGFIKLLSTEEN		KLLGAWMIGPHVSEMIHTTALAITYG
	Theileria orientalis str. Shi.	XP_009691656	-A--------G-T---V---AD-A-		R-----LV---A---V-QL----K--
	Theileria equi	XP_004832497	Y-G-------G-----V-I-ADK--		-I----IV--DA--L-AQLTI-V---
	Theileria annulata	XP_954919	------------TE--V---AN-Q-		-------V-------V-L-V------
	Babesia sp. Xin.	ORM40305	-A-----R-AG-T---V-I-ADKD-		-I--G-IV---A--L-GQITIMMAC-
	Babesia ovata	GBE62323	-A-----RVAG-T---V-I-ADND-		-I--G-IV---A--L-GQIT-MMAC-
	Babesia bovis T2Bo	XP_001610643	-A-----R-AGDV---V-I-ADKD-		-I--G-IV---A--L-GQITIMMAC-
	Babesia bigemina	XP_012769794	-A----SRVAG-T---V-I-ADND-		-I--G-I---QA--L-GQIT-MMAC-
	Babesia divergens	LK934716.1	---SR-AE-TE--V-I-ADKD-		-I--G-IV---A--LVGQLT-MMAC
B. microti	*Babesia microti str. RI*	XP_012649464	LV------LA--TT--V-V--D--S	G	-I----IF-TQA--L-A-FG------
**Other Apicom-plexa**	*Besnoitia besnoiti*	PFH35994	-A-----RANDVVQ--V-I-TDK-T	D	-I----IM--EAG-L-AQLV-GME--
	Cyclospora cayetanensis	XP_022589645	-V-----RATGD---IV-V-ACK-T	D	----V-IL-QGAG-L-AEAV--ME--
	Eimeria maxima	XP_013332622	-A-----RATGD---LV-V-TCK-T	D	-I--V-IL--NAG-L-AEAV--ME--
	Eimeria necatrix	XP_013433469	-A-----RATGD---LV-V-TCK-T	D	-I--V-IL--NAG-L-AEAV--ME--
	Eimeria praecox	CDI86766	-A-----RATGD---IV-V-TCKDT	D	-I--V-IL--NAG-L-AEAV--ME--
	Eimeria tenella	XP_013229686	-A-----RATGD---LV-V-TCK-T	D	-I--V-IL--NAG-L-AEAV--ME--
	Hammondia hammondi	XP_008886018	-A-----RANDVAT--V-V-AHKDS	D	------IM--EAG-L-GQLV-GME--
	Neospora caninum Liv.	CEL68674	-A-----RANDVAT--V-V-AHK-S	D	-I----IM--EAG-L-GQLV-GME--
	Toxoplasma gondii ME49	XP_002367828	-A-----RANDVAT--V-V-AHKDS	D	------IM--EAG-L-GQLV-GME--
**Proteo-bacteria**	*Pelagibacteraceae bac. GO.*	OCW83227	--------AID-PE--V-I-ADQST	D	-V--VHI----AG---AEM-V-MEF-
	Caulobacterales bac. RIFO.	OGN49878	-T-------NH-T---V-V-ADAAT	D	-V--VHIM--QAG----EA-VTMSF-
	Rickettsiales bac. TMED.	OUW24307	--------VN--TE--V-I-ANRDT	D	-V--VHI----CGD--AEM---MEF-
	Candidatus Pelagibacter sp. TM.	OUW48613	-L------VN---E--V-I-ADART	D	-V--VHI----CGD--AEM---MEF-
	Pelagibacteraceae bac. TM.	OUX32806	-I------AI--PE--V-I-AD-KT	D	-I--VH------G-I-GEV-V-MEF-
	Acidiphilium sp. 21-66-27	OYV66677	----G--RAMG-T---V----DKTT	D	-----HI---DAGTI-AELV---EF-
	Pelagibacterales bac. MED.	PDH20601	--------AIDDAE--V-I-AD-TT	D	-V---HI----AG-L-AEIGV-MEF-
	Robiginitomaculum sp.	PHS39316	-------RANH-T---V-I-AHA-T	D	EI---H---KG-----AEV---MEFK
	Candidatus Pelagibacter sp. HT.	WP_008544322	--------AIDDAE--V-I-AD-TT	D	-V---HI----AG-L-AEIGV-MEF-
	Commensalibacter intestini	WP_008854765	-S--G--RAM-KT---V-I--DKRT	N	-V---HI----C----AEIV--MNF-
	Candidatus Pelagibacter sp. IM.	WP_013695122	---------N-DA-----I-ADKST	D	-I--VH----D-GT--AEVV--MEF-
	Candidatus Pelagibacter ubi.	WP_023647882	--------AIDDAE--V-I-AD-TT	D	-V---HI----AG-L-AEIGV-MEF-
	Azospirillum halopraeferens	WP_029011035	-T--G--RAM-AT-----I-ADART	D	-V--VH----N----VAEL---MEF-
	Brevundimonas nasdae	WP_039243962	-T-------NH-T---V-V-ADAAT	D	-V--VHIM--QAG----EA-ITMSF-
	Brevundimonas sp. Leaf168	WP_055807593	-T-------NH-T---V-V-ADAAT	D	-V--VHIM--QAG----EA-ITMSF-
	Brevundimonas sp. Leaf363	WP_056099114	-T-------NH-T---V-V-ADATT	D	-V--VHIV--QAG----EA-ITMSF-
	Brevundimonas sp. GW460.	WP_066552900	-T-------NH-T---V-V-ADAAT	D	-V--VHIM--QAG----EA-ITMSF-
	Brevundimonas vesicularis	WP_066627425	-T-------NH-T---V-V-ADAAT	D	-V--VHIM--QAG----EA-ITMSF-
	Hyphomonas sp. Mor2	WP_070958448	-------RTNH-TA--V-I-AE-GT	D	-I---H---VG-G----EI-I-MEF-
	Candidatus Pelagibacter ubi.	WP_075534421	--------AI--AE--V-I-AD-KT	D	-V--VHL----AG-L-AEM-V-MEF-
	Brevundimonas sp. 374	WP_091750748	-T-------NH-T---V-V-ADAAT	D	-V--VHIM--QAG----EA-ITMSF-
	Candidatus Fonsibacter ubi.	WP_099340836	-A--A---VND-G---V-I--D-KT	D	RV--VH----D-GN--GEMC--MEF-
	Acidovorax sp. 16-64-162	OYZ44529	-L--G--RALGDTT-MV-F-ADAVT	D	EI--VH-V--Q---L-AEAVV-MEFK
	Nitrosospira sp. NpAV	WP_041514166	----G--RALG-TG--V-V-ADVDT	D	RI--VH----Y-----AEAVV-MEFA
	Nitrosospira multiformis	WP_074633669	----G--RALG-TG--V-V-ADADT	D	RI--IH----Y-----AEAVV-MEFA
**Other Eukarya**	*Fistulifera solaris*	GAX16732	-S-----RANATT---V-V-ADA-T	D	RI--CHIM--NAG---AEAVI--E--
	Blastocystis hominis	ABU54774	LQ-----RA-DQP--L--V-ADKKT	N	-I--VH-C-LN---L-AEAG--ME--
	Stylonychia lemnae	CDW75606	-------RTN-D-E-LV-V-ADKDT	D	-I--VHI---NAG---AEGV-GME--
	Oxytricha trifallax	EJY72722	-Q-----RAN-D---LV-I-TDQQS	G	-I---HI---NAG---AEGV-GME--
	Paramecium tetraurelia str.	XP_001457469	-L------AND-IE----V-TDKKT	D	----VHIV--NAG---AEAV-G-E--
	Saccharomyces cerevisiae YJ.	AJU39818	-A------TNQDT---V-I-IDAKT	E	RI---HI---NAG---AEAG--LE--
	Komagataella pastoris	ANZ75845	-I------TNLDTE--V-F-ADA-T	Q	RV--VHI---NAG---AEAG--LE--
	Candida parapsilosis	CCE42952	-I------TNLDT---V-I-ADA-T	Q	RV---HI---NAG---AEAG--LE--
	Zygosaccharomyces bailii CL.	CDF90894	-I------TNLDT---V-I-IDADT	E	RM---HI---NAG---AEAG--LE--
	Galactomyces candidum	CDO54912	-L------TNLDT---V-F-ADK-T	D	RV--VHI---NAG---AEAV--VE--
	Kluyveromyces dobzhanskii CBS.	CDO93767	-I------TNMDTE--V-I-IDA-S	E	R----HI---NAG---AEAG--LE--
	Talaromyces cellulolyticus	GAM40280	-S------TNL-TE-LV-F--DA-T	D	RI--VHIL--NAG---AEAT--VE--
	Talaromyces marneffei PM1	KFX51866	-S------TNLDTE-LV-F--DA-T	D	RI--VHIL--NAG---AEAT---E--
	Plicaturopsis crispa FD-325 S.	KII85484	-L------TNADTE--V-F--EK-T	D	RI--VHI---NAG---AEGV---E--
	Sphaerobolus stellatus SS.	KIJ30477	-A------TNQDT--LV-F-AEA-T	D	RV--VHIM--YAG-L-AAAS--ME--
	Pisolithus microcarpus 441	KIK26301	-A------TNLD-E--V-F--EK-T	D	RI--VHI---NAG---SEAV---E--

Figure S9. A partial sequence alignment of the Dihydrolipoamide dehydrogenase protein showing a one amino acid deletion that is specific for members of *Piroplasmida* (sans *B. microti*) and some proteobacteria (not shown). The *Babesia divergens* sequence was acquired from a tblastn search conducted against the *Theileria parva str. Mug.* sequence. Expanded alignment of Figure 3 (B).

Group	Species	Accession	19		74
***Piropla-smida* (7/8)**	*Theileria equi*	XP_004832720	EIRVTTLGRVSNYVTYAKKLL		SSGIPVITIRGTGRAMSNVVETAEILRHMIEGLHQ
	Theileria parva str. Mug.	XP_762779	---------------------		-N--------------------------N----
	Theileria orientalis str. Shi.	XP_009691252	---------------------		-N-V----------------------S-N----
	Theileria annulata	XP_955356	---------------------		-N--------------------------N----
	Babesia bigemina	XP_012768608	----SSI-V--S--A-----I		AA-E---H------------------KRVYK-M--
	Babesia bovis T2Bo	XP_001611317	-----SV-L-YG--N--R--I		DG-E--V-L------------------RAYK-M--
	Babesia sp. Xin.	ORM42214	-----SH-L-YG--N------		DG-E--V-L-------T----------RAYR-M--
	Babesia divergens	LK934711.1	----MS--L--S--S-----I		AA-E---------K-----I------KRTYK-M--
	Babesia ovata	GBE59510.1	----ASI-L--G--A-----I		A--E---H-------I-----T----KRAYK-M--
B. microti	*Babesia microti str. RI*	XP_021338413	--QI-AS--LP--LR--T---	V	KD-LST-I-K-A-K-IIM-LMVV-S-KFN----Y-
**Other Apicom-plexa**	*Plasmodium cynomolgi str. B*	XP_004224859	-M-I-ST--MT---N-GA-I-	G	EEDKKSLK-KA--N-IGKA-TL---IKRRFK----
	Plasmodium gallinaceum	CRG97642	-M-I-ST--MT---N--A---	G	EEEKKS-K-KA--N-IAKA-TLT--VKRRFK----
	Plasmodium relictum	CRH02502	-M-I-ST--MT---N--A---	G	EEEKKS-K-KA--N-IAKA-TL---VKRRFK----
	Eimeria acervulina	XP_013250767	------M---EG--S--ST-I	E	DQKKSQ---KAS-N-IGQAIAL--Q-KRRFR----
	Besnoitia besnoiti	PFH33842	---I-SA-K-M---A--AR-M	T	EQNMRK---KA--N-IARA-TLT-V-KRRFK----
	Cystoisospora suis	PHJ20053	---I-SA-K-M---A--AR-I	T	EQNMRQ---KA--N-IAPA-TLT-V-KRRFK----
	Hammondia hammondi	XP_008886287	---I-SA-K-M---A--AR--	T	EQNMRK-N-KA--N-IARA-TL--V-KRRFK--Y-
	Neospora caninum Liv.	XP_003880051	---I-SA-K-M---A--AR--	T	EQNMRK---KA--N-IARA-TL--V-KRRFK----
	Toxoplasma gondii ME49	XP_002369967	---I-SA-K-M---A--AR--	T	EQNMRK-N-KA--N-IARA-TL--V-KRRFK--Y-
**Other Eukarya**	*Vitrella brassicaformis CCMP.*	CEM10131	---I-SM---T------L---	Q	DQ-KKSV--KA--T-IDRA-HV--L-KRRYA----
	Picea sitchensis	ABR18130	---I--Q-MPR--I---MT--	R	EK-ASD-ILKAM---I-KT-TI---IKRR-P----
	Marchantia polymorpha	APT68000	---I--Q-KMR--I---TT--	Q	DK-ASE-VLKAM---INKT-TI---IKRR-A----
	Zea mays	AQL09789	-V-I--Q-L-R------TS-V	Q	EKQVKE-VLKAM-Q-I-KA-AI---IKKR-P----
	Hordeum vulgare subsp. vul.	BAJ96858	---I--Q--MR--I---TT-F	Q	DK-CDEVVFKAM---INKT-MI--LIKRR-V----
	Brassica napus	CDY08020	---I-SV-LIR--IS--IT--	H	EK-AED-VLKAM-Q-I-KT-AIS---KSKVP----
	Oryza sativa Indica Group	EEC74582	---I--Q-LIR------TS--	Q	EKRVKE-VLKAM-Q-I-KT-AI---IKKR-P----
	Triticum urartu	EMS66176	---I-AQ--TR--I---LA--	Q	EEATDE-V-KAM---INKT-AIV-L-KRR-A----
	Cephalotus follicularis	GAV87694	---I-SQ--MR--IA--MT--	Q	EK-SDE-VFKAM---INKT-AIV-LIKRR-V----
	Citrus sinensis	KDO53193	-L-I-AQ--LR--IS--IS--	E	EK-ANEVVLKA----INKT-MI--L-KRRVV----
	Arabis alpina	KFK38158	---I-SG-LIR--IS--TS--	Q	EK-VKDVVLKAM-Q-I-KT-AIS---KNK-P----
	Gossypium arboreum	KHG13806	-L-I-AQ--MR--IS--MT--	Q	EK-ANE-VLKA----INKT-MI--LIKRR-A----
	Vigna angularis	KOM49057	---I-SQ--MR--I---MS--	Q	EK-SNE-VFKAM---INKT-TIV-LIKRR-V----
	Daucus carota subsp. sat.	KZM93379	---I--Q--MR--I---TT--	Q	EK-SDE-ALKAM---I-KT-MI--LIKRR-A----
	Glycine max	NP_001235523	---I-SQ--MR--I---MS--	Q	EK-SNE-VFKAM---INKT-TIV-LIKRR-V----
	Arabidopsis thaliana	NP_001327440	---I-SK-LIR--IS--TS--	Q	EKSVKD-VLKAM-Q-I-KT-AIS---KNK-P----
	Marchantia polymorpha subsp. r.	OAE28625	---I--Q-KMR--I---TT--	Q	DK-ASE-VLKAM---INKT-TI---IKRR-A----
	Dichanthelium oligosanthes	OEL35720	---I--Q--MR--I---TA--	Q	DK-SDEVVFKAM---INKT-MI--LIKRR-V----
	Lupinus angustifolius	OIW20366	------Q--MR--I---TT--	H	ER-SDE-VLKAM---INKT-VI--LIKRR-I----
	Corchorus olitorius	OMO88329	---I-AQ-LIR--I---IS--	Q	EKQTG--VLKAM-Q-I-KT-AI---IKKR-P----
	Sorghum bicolor	OQU93168	---I--Q-LIR------TS-V	Q	EKRVKE-VLKAM-Q-I-KT-AI---IKKR-P----
	Panicum hallii	PAN13607	---I--Q--MR--I---TA--	Q	DK-SDEVVFKAM---INKT-MI--LIKRR-V----
	Physcomitrella patens	XP_001753647	---I--Q-KMR--I---TT--	Q	DK-ASA-VLKAM---INKA-TI---IKRR-A----
	Vitis vinifera	XP_002264932	---I-AQ--MR--I---TT--	Q	DK-SDE-ALKAM---INKT-MI--LIKRR-A----
	Malus domestica	XP_008361539	---I-AQ--MR--I---TT--	H	EK-SNE-VLKAM---I-KT-MI--LIKRR-V----
	Phoenix dactylifera	XP_008775139	---I--Q-LIR---S--SS--	Q	EKRVRE-VLKAM-Q-I-KA-AV---IKNK-P----
	Nelumbo nucifera	XP_010246262	---I--Q--MR--I---TS--	Q	EK-SNE-VLKAM---INKT-MI--LIKRR-A----
	Camelina sativa	XP_010464305	---I-SQ-LIR--IS--TS--	Q	EK-VKD-VLKAM-Q-I-KT-AIS---KNK-P----
	Beta vulgaris subsp. vul.	XP_010680301	---I--Q--MR--I---TT--	Q	EK-SDE-VLKAM---INKT-MI--LIKRR-V----
	Elaeis guineensis	XP_010909764	---I--Q-LIR---S--SS--	Q	EKRVRE-VLKAM-Q-I-KT-AV---IKKR-P----
	Populus euphratica	XP_011042874	---I--Q--MR--I---TT-F	Q	EK-SDE-SLKAM---INKT-MI--LIKRR-A----

Figure S10. A partial sequence alignment of a Conserved hypothetical protein showing a one amino acid deletion that is specific for members of *Piroplasmida* sans *B. microti*. The *Babesia divergens* sequence was acquired from a tblastn search conducted against the *Theileria equi* sequence.

Group	Species	Accession	334		388
**Piroplasmida (7/8)**	*Theileria equi*	XP_004832649	FGTFSRKFIAGLLDKHSQPLPLDFIRY		PSTQRNISQVLTRPWALSTFVNLLKNSS
	Babesia ovata	GBE63062.1	------R-M-K-IQR-A--QA---L--		RAG--Q-N-I-------FGY-KM--K--
	Theileria orientalis str. Shi.	XP_009691166	Y-------L-N-INR----S----F--		Q-HS--LPLI-----S-LS------S--
	Theileria parva str. Mug.	XP_762865	Y-------L-N-INRY-D-F----L--		E-HS--LPMI----SS-ISY------C-
	Theileria annulata	XP_955431	Y-------LSN-ISRY-D-F----L--		A-HS--LPMI----FS-ISY------C-
	Babesia bovis T2Bo	XP_001611236	--------M-K-IQQQAN------L--		KAH-KAPN-LV------FGY-KK--K--
	Babesia bigemina	XP_012768522	------R-L-K-IQR-A--QA---L--		RAG--Q-N-I-------FGY-KM--K--
	Babesia divergens	LK934711.1	------R-M-K-IQT--R------L--		RAG--SMN-I-------FGY-K---K-
**B. microti**	*Babesia microti str. RI*	XP_012648104	------SY--KS---SAK-RLF--V-F	SR	FGGTKKLPL---Q-RT-LGY-----DC-
**Plasmodium (0/20)**	*Plasmodium sp. DRC-Itaito*	SOV22175.1	Y-----A--VNMY--Y-KKRLF--V--	EN	NMGI-KLPNI--H-YH--SYTK--GEC-
	Plasmodium sp. Gor. Cla. G2	SOV13510.1	Y-----A--VNMY--Y-KKRLF--V--	EN	NMGI-KLPNI--H-YH--SYTK--GEC-
	Plasmodium berghei ANKA	CDS51860	Y-----S--VNMY--YFRKRLF--V--	EN	NMGI-K-PSI--H-YH--SYIK--GEC-
	Plasmodium chabaudi ada.	SCN62992	Y-----S--VNMY--YFRKRLF--V--	EN	NMGI-K-PSI--H-YH--SYIK--GEC-
	Plasmodium chabaudi cha.	CAH83313	Y-----S--VNMY--YFRKRLF--V--	EN	NMGI-K-PSI--H-YH--SYIK--GEC-
	Plasmodium coatneyi	XP_019916142	YA----S--VNMY--YFRKRLF--V--	EN	NFGI-K-PNI--H-YH--SYIK--GEC-
	Plasmodium cynomolgi str. B	XP_004224907	YA----S--VNMY--YFRKRLF--V--	ES	NFGI-K-PNI--H-YH--SYIK--GEC-
	Plasmodium falciparum 3D7	XP_001349432	Y-----A--VNMY--YFKKRLF--V--	EN	NMGI-K-PNI--H-YH--SYTK--GEC-
	Plasmodium falciparum Dd2	KOB85860	Y-----A--VNMY--YFKKRLF--V--	EN	NMGI-K-PNI--H-YH--SYTK--GEC-
	Plasmodium falciparum FCH/4	ETW31227	Y-----A--VNMY--YFKKRLF--V--	EN	NMGI-K-PNI--H-YH--SYTK--GEC-
	Plasmodium falciparum Mal.	ETW49849	Y-----A--VNMY--YFKKRLF--V--	EN	NMGI-K-PNI--H-YH--SYTK--GEC-
	Plasmodium falciparum NF135/5.	ETW43233	Y-----A--VNMY--YFKKRLF--V--	EY	NMGI-K-PNI--H-YH--SYTK--GEC-
	Plasmodium falciparum Pal. Alt.	ETW52567	Y-----A--VNMY--YFKKRLF--V--	EN	NMGI-K-PNI--H-YH--SYTK--GEC-
	Plasmodium falciparum San. Lu.	EUT87155	Y-----A--VNMY--YFKKRLF--V--	EN	NMGI-K-PNI--H-YH--SYTK--GEC-
	Plasmodium falciparum Tan.	ETW37169	Y-----A--VNMY--YFKKRLF--V--	EN	NMGI-K-PNI--H-YH--SYTK--GEC-
	Plasmodium falciparum UGT5.1	EWC77207	Y-----A--VNMY--YFKKRLF--V--	EN	NMGI-K-PNI--H-YH--SYTK--GEC-
	Plasmodium falciparum Vie.	ETW19004	Y-----A--VNMY--YFKKRLF--V--	EN	NMGI-K-PNI--H-YH--SYTK--GEC-
	Plasmodium fragile	XP_012336948	YA----S--VNMY--YFRKRLF--V--	ES	NMGI-K-PNI--H-YH--SYIK--GEC-
	Plasmodium gaboni	XP_018642190	Y-----A--VNMY--Y-KKRLF--V--	EN	NMGI-KLPNI--H-YH--SYTK--GEC-
	Plasmodium gallinaceum	CRG97691	Y-S---S--VSMY--YFKKRLF--V--	EN	NLGI-K-PNI--H-YH--SYIK--GEC-
	Plasmodium gonderi	GAW83504	YA----S--VSMY--YFKKRLF--V--	EN	NMGI-K-PNI--H-YH--SYIK--GEC-
	Plasmodium inui San Ant. 1	XP_008814511	YA----S--VSMY--YFRKRLF--V--	EN	NLGI-K-PNI--H-YH--SYIK--GEC-
	Plasmodium knowlesi str. H	XP_002262196	YA----S--VNMY--YFRKRLF--V--	EN	NFGI-KLPNI--H-YH--SYIK--GEC-
	Plasmodium malariae	SBS90624	Y-----S--VNMY--YFRKRLF--V--	EN	NMGI-K-PNI--H-YH--SYIK--GEC-
	Plasmodium ovale curt.	SBS91299	Y-----S--VNMY--YFRKRLF--V--	EN	NMGI-K-PSI--H-YH--SYIK--GEC-
	Plasmodium ovale wal.	SBT48330	Y-----S--VN-Y--YFRRRLF--V--	EH	NMGI-K-PNI--H-YH--SYIK--GEC-
	Plasmodium reichenowi	CDO63862	Y-----A--VNMY--YFKKRLF--V--	EN	NMGI-K-PNI--H-YH--SYTK--GEC-
	Plasmodium relictum	CRH02551	Y-S---S--VNMY--YFKKRLF--V--	EN	NLGI-K-PNI--H-YH--SYIK--GEC-
	Plasmodium vinckei pet.	EUD74354	Y-----S--VNMY--YFRKRLF--V--	EN	NMGI-K-PSI--H-YH--SYIK--GEC-
	Plasmodium vinckei vin.	XP_008622950	Y-----S--VNMY--YFRKRLF--V--	EN	NMGI-K-PSI--H-YH--SYIK--GEC-
	Plasmodium vivax Bra. I	KMZ84386	YA----S--VS-Y--Y--KRLF--V--	EN	NFGI-KMPNI--H-YH--SYIK--GEC-
	Plasmodium vivax Ind. VII	KMZ78045	YA----S--VS-Y--Y--KRLF--V--	EN	NFGI-KMPNI--H-YH--SYIK--GEC-
	Plasmodium vivax Mau. I	KMZ90165	YA----S--VS-Y--Y--KRLF--V--	EN	NFGI-KMPNI--H-YH--SYIK--GEC-
	Plasmodium yoelii 17X	ETB61587	Y-----S--VNMY--YFRKRLF--V--	EN	NMGV-K-PSI--H-YH--SYIK--GEC-
	Plasmodium yoelii yoe. 17XNL	XP_727627	Y-----S--VNMY--YFRKRLF--V--	EN	NMGV-K-PSI--H-YH--SYIK--GEC-
**Other Apicomplexa**	*Besnoitia besnoiti*	PFH37004	--C--KQ--VKQIERSAN-RLF--V--	AH	KGGVKPLPPIV-H-HR--SY-R--DE--
	Cyclospora cayetanensis	XP_022591787	--A--KA-L-RHIERVTN-RFF--V--	QQ	HGGL-PLPAIVRH-YR--SY-Q--GEC-
	Cystoisospora suis	PHJ17263	--C--KQ--VNQIE-STK-RFF--V--	ER	KGGIKPLPPII-H-HR--SY-RM-DE--
	Hammondia hammondi	XP_008886662	--C--KQ--VKQIERSAN-RLF--V--	AH	KGGVKPLPPIV-H-HR--SY-R--DE--
	Neospora caninum Liv.	CEL69522	--C--KQ--VQQIERSAN-RLF--V--	AH	KGGVKPLPPIV-H-HR--SY-R--DE--
	Toxoplasma gondii ME49	XP_002370856	--C--KQ--VKQIERSAN-RLF--V--	AH	KGGVKPLPPIV-H-HR--SY-R--DE--

Figure S11. A partial sequence alignment of a Conserved hypothetical protein showing a two amino acid deletion that is specific for members of *Piroplasmida* sans *B. microti*. The *Babesia divergens* sequence was acquired from a tblastn search conducted against the *Theileria equi* sequence.

Group	Species	Accession	1280		1322
**Piroplasmida (7/8)**	*Babesia bigemina*	XP_012767741	AMTCTHASLGRETMESLRYSNLV		MEEAAQVLEAETFALLAHP
	Babesia ovata	GBE61274	-----------------------		-------------------
	Babesia sp. Xin.	ORM41211	-------AIA---LS--------		-------------------
	Babesia bovis T2Bo	XP_001608843	-------AIA---LSN-------		-------M-----------
	Babesia divergens	LK934717.1	-------AIAK----N---D---		---------V----P----
	Theileria equi	XP_004831810	-------AIA-QEL-HVK--T--		L--------I---IP--QN
	Theileria annulata	XP_954844	--------ISQ-ELST-N-KT-I		F-----I--I-S-IPICNN
	Theileria parva str. Mug.	XP_763162	--------ISQ-ELST-N-KT--		F-----I--I-S-IPICNN
	Theileria orientalis str. Shi.	XP_009691746	--------IAK-ELAT-N-KS--		F-----I--I-S-IPICSN
**B. microti**	*Babesia microti str. RI*	XP_012648159	-------AIA--RLIESNFTYDT	LV	---------I---IP-TLQ
**Other Eukarya**	*Chlamydomonas reinhardtii*	XP_001691434	-------A-K-REFLQ-AFKYDN	LL	------I--I---IPMLLQ
	Volvox carteri f. nag.	XP_002949258	-------A-K-REFLQ-AFKYDN	LL	------I--I---IPMLLQ
	Chlorella variabilis	XP_005851348	-------A-K-KEFLE-GFKYDN	LL	---S--I--I---IPVLLQ
	Monosiga brevicollis MX1	XP_001749129	-----Y-A-M--DLA-YGFRYDN	IV	---S--I--V---IP-VLQ
	Salpingoeca rosetta	XP_004994011	-------A-T-SELVK-GFKYDN	VV	------I--V---IP-MLQ
	Vitrella brassicaformis CCMP.	CEM26013	-------A-T-HNLID--FKYDN	LI	---S--I--V---IPMLLQ
	Symbiodinium microadriaticum	OLQ07810	-------A-T-S-LVN-SFKYDN	LL	---S--I--V---IPMLLQ
	Lichtheimia ramosa	CDS09083	-------A-K-SELVE-NFKYDN	IV	------I--V---IPMLLQ
	Parasitella parasitica	CEP09725	-------A-K-RELAK-GFKYDN	II	I-----I--V---IP-LLQ
	Mucor circinelloides f. circ.	EPB87575	-------A-K-RELVK-GFKYDN	IV	I-----I--V---IP-LLQ
	Rhizophagus irregularis DAOM.	ESA09756	-------A-K-RELV--GFKYDN	VV	------I--V---IP-LLQ
	Mucor ambiguus	GAN09003	-------A-K-RELVK-GFKYDN	IV	I-----I--I---IP-LLQ
	Mortierella verticillata NR.	KFH70963	-------A-K-RELVN-GFKYDN	IV	------I--V---IPMLLQ
	Mucor circinelloides f. lus.	OAD06901	-------A-K-RELVK-GFKYDN	IV	I-----I--V---IP-LLQ
	Mortierella elongata AG-77	OAQ24982	-------A-K-RELVN-GFKYDN	IV	------I--V---IPMLLQ
	Hesseltinella vesiculosa	ORX52031	-------A-K-RELVD-KFKYDN	VV	---S--I--V---IPMLLQ
	Basidiobolus meristosporus C.	ORX97785	-------A-K-RELV--GFKYDN	IV	------I--V---IP-LLQ
	Absidia repens	ORZ10754	-------A-K-RELIA-KFKYDN	VV	------I--V---IP-LLQ
	Absidia glauca	SAM05972	-------A-K-RELVD-KFKYDN	VV	------I--V---IP-LLQ
	Phycomyces blakesleeanus NR.	XP_018286238	-------A-K-RELVG-NFKYDN	VV	----G-I--V---IP-LLQ
	Lobosporangium transversale	XP_021878018	-------A-K-RELVN-GFKYDN	IV	------I--V---IPMLLQ
	Drosophila melanogaster	AAM50843	-------A-K-KELVN-GFRYDN	IL	---S--I--I---IP-LLQ
	Heterodera avenae	AGO32789	-------A-R-RELVE-GFRYDN	IL	------I--V---IP-LLQ
	Homo sapiens	BAF98706	-------A-K-HDLVK-GFKYDN	IL	------I--I---IP-LLQ
	Tetraodon nigroviridis	CAG10389	-------A-K-HDLVE-GFKYDN	IL	------I--I---IP-LLQ
	Caenorhabditis briggsae	CAS00769	-------A-R-NELVK-GFKYDN	IV	------I--V---IP-LLQ
	Lichtheimia corymbifera JM.	CDH58321	-------A-K-SELLE-NFKYDN	IV	------I--V---IPMLLQ
	Haemonchus contortus	CDJ83109	-------A-R-NELVQ-GFRYDN	IL	------I--V---IP-LLQ
	Oncorhynchus mykiss	CDQ96838	-------A-K-HDLVE-GFKYDN	IL	------I--I---IP-LLQ
	Bos taurus	DAA25489	-------A-K-HDLVK-GFKYDN	IL	------I--I---IP-LLQ
	Caenorhabditis brenneri	EGT51377	-------A-R-NELVK-GFRYDN	IV	------I--V---IP-LLQ
	Capitella teleta	ELT95764	-------A-K-RDLVEQGFQYDN	IL	---S--I--I---IP-LLQ
	Anopheles darlingi	ETN63350	-------A-K-KELVNMGFKYDN	IL	------I--I---IP-LLQ
	Ancylostoma ceylanicum	EYC03617	-------A-R-NELVQ-GFRYDN	IL	------I--V---IP-LLQ
	Anopheles sinensis	KFB41306	-------A-K-KELVTMGFKYDN	IL	------I--I---IP-LLQ
	Toxocara canis	KHN88639	-------A-R-KELVE-GFRYDN	IL	------I--V---IP-LLQ
	Ancylostoma duodenale	KIH57401	-------A-R-NELVQ-GFRYDN	IL	------I--V---IP-LLQ
	Larimichthys crocea	KKF14004	-------A-K-HDLVE-GFKYDN	IL	------I--I---IP-LLQ
	Sarcoptes scabiei	KPM03788	-------A-K-KELV---FQYDN	IL	------I--I---IP-LLQ
	Alligator mississippiensis	KYO28749	-------A-K-HDLVE-GFKYDN	IL	------I--I---IP-LLQ
	Rattus norvegicus	NP_001094457	-------A-K-HDLVK-GFKYDN	IL	------I--I---IP-LLQ
	Rana catesbeiana	PIO39878	-------A-K-HDLVD-GFKYDN	IL	------I--I---IP-LLQ
	Teladorsagia circumcincta	PIO69570	-------A-R-NELVQ-GFRYDN	IL	------I--V---IP-LLQ
	Drosophila pseudoobscura pse.	XP_001359640	-------A-K-KELVN-GFRYDN	IL	---S--I--I---IP-LLQ
	Equus caballus	XP_001503693	-------A-K-HDLVK-GFKYDN	IL	------I--I---IP-LLQ
	Drosophila ananassae	XP_001964423	-------A-K-KELAN-GFRYDN	IL	---S--I--I---IP-LLQ
	Oryctolagus cuniculus	XP_002718043	-------A-K-HDLVK-GFKYDN	IL	------I--I---IP-LLQ
	Ailuropoda melanoleuca	XP_002917855	-------A-K-HDLVK-GFKYDN	IL	------I--I---IP-LLQ
	Xenopus tropicalis	XP_002932889	-------A-K-HDLVE-GFKYDN	IL	------I--I---IP-LLQ
	Nomascus leucogenys	XP_003272843	-------A-K-HDLVK-GFKYDN	IL	------I--I---IP-LLQ
	Amphimedon queenslandica	XP_003383306	-L-----A-K-KDLVD-SFKYDN	VL	------I--V---IP-MLQ

Supplementary Figure 12. A partial sequence alignment of the Intron-binding aquarius beta like protein showing a two amino acid deletion that is specific for members of *Piroplasmida* sans *B. microti*. The *Babesia divergens* sequence was acquired from a tblastn search conducted against the *Babesia bigemina* sequence.

Group	Organism	Accession	1303		1346
***Piroplasmida* (7/8)**	*Babesia bigemina*	XP_012767741	MEEAAQVLEAETFALLAHP		LKRVILSGDHYQLPPVVNNRSLLQF
	Babesia ovata	GBE61274	-------------------		-------------------------
	Babesia sp. Xin.	ORM41211	-------------------		---------------------I-Q--
	Babesia bovis T2Bo	XP_001608843	-------M-----------		---I------------I-----QR-
	Babesia divergens	LK934717.1	---------V----P----		----------F---------G--KY
	Theileria equi	XP_004831810	L--------I---IP--QN		V---V-----L------Q-KCM
	Theileria annulata	XP_954844	F-----I--I-S-IPICNN		I--L--C---L--S-IIQ-S---RY
	Theileria parva str. Mug.	XP_763162	F-----I--I-S-IPICNN		I--L--C---L--S-IIQ-S---RY
	Theileria orientalis str. Shi.	XP_009691746	F-----I--I-S-IPICSN		I--LV-C---L--S-IIQ-GA-QKY
B. microti	*Babesia microti str. RI*	XP_012648159	---------I---IP-TLQ	LQGDK	---IV-I--NR----IIQ--TVASY
**Other Eukarya**	*Chlamydomonas reinhardtii*	XP_001691434	------I--I---IPMLLQ	KPEDGVSR	----V-I---H------K-QAFQKY
	Volvox carteri f. nag.	XP_002949258	------I--I---IPMLLQ	KPEDGVSR	----V-I---H------K-QAFQKY
	Chlorella variabilis	XP_005851348	---S--I--I---IPVLLQ	RQEDGHNR	------I---H------K-MAIQ-Y
	Monosiga brevicollis MX1	XP_001749129	---S--I--V---IP-VLQ	NPQDGRNL	----T-I---N-----IR-PAFKK-
	Salpingoeca rosetta	XP_004994011	------I--V---IP-MLQ	NTEDGYNR	----M-I---H-----IK-TAFKK-
	Vitrella brassicaformis CCMP.	CEM26013	---S--I--V---IPMLLQ	QSERGVSR	----V-I---H------K--AFQRY
	Symbiodinium microadriaticum	OLQ07810	---S--I--V---IPMLLQ	NAESGVSR	----M-I---H------K--AFQKY
	Lichtheimia ramosa	CDS09083	------I--V---IPMLLQ	RSDEGESR	------I---H------K-MAFQ-Y
	Parasitella parasitica	CEP09725	I-----I--V---IP-LLQ	EPKDGQNP	----M-I---H-----IK-SAFQ--
	Mucor circinelloides f. cir.	EPB87575	I-----I--V---IP-LLQ	EPKDNQNL	----M-I---H-----IK-SAFQ--
	Rhizophagus irregularis DA.	ESA09756	------I--V---IP-LLQ	ETEDGRSR	-----MI---N------K-MAFQ-Y
	Mucor ambiguus	GAN09003	I-----I--I---IP-LLQ	EPKDGQNL	----M-I---H-----IK-SAFQ--
	Mortierella verticillata NR.	KFH70963	------I--V---IPMLLQ	EPEDGVNR	------I---N------K-TAFQ-Y
	Mucor circinelloides f. lus.	OAD06901	I-----I--V---IP-LLQ	EPKDGQNL	----M-I---H-----IK-AAFQ--
	Mortierella elongata AG-77	OAQ24982	------I--V---IPMLLQ	DPEDGVNR	------I---N------K-TAFQ-Y
	Hesseltinella vesiculosa	ORX52031	---S--I--V---IPMLLQ	EPEDGQNR	------I---H------K-QAFQ-Y
	Basidiobolus meristosporus C.	ORX97785	------I--V---IP-LLQ	DSEEGRSR	-----MI---N------Q-VGFQ-Y
	Absidia repens	ORZ10754	------I--V---IP-LLQ	EPEDGQNR	------I---H------K-LAFQ-Y
	Absidia glauca	SAM05972	------I--V---IP-LLQ	EPEDGENR	------I---N------K-LAFQ-Y
	Phycomyces blakesleeanus NR.	XP_018286238	----G-I--V---IP-LLQ	EPEDGVNR	-----MI---N------K-LAFQ-Y
	Lobosporangium transversale	XP_021878018	------I--V---IPMLLQ	EPEDGVNR	------I---N------K-T-FQ-Y
	Drosophila melanogaster	AAM50843	---S--I--I---IP-LLQ	NPLDGLNR	---W-MI---H-----IK-MAFQKY
	Heterodera avenae	AGO32789	------I--V---IP-LLQ	DPQDGRSR	---WVMI---H------Q-VAYQK-
	Homo sapiens	BAF98706	------I--I---IP-LLQ	NPQDGFSR	---W-MI---H-----IK-MAFQKY
	Tetraodon nigroviridis	CAG10389	------I--I---IP-LLQ	NPEDGYSR	---W-MI---H-----IK-MAFQKY
	Caenorhabditis briggsae	CAS00769	------I--V---IP-LLQ	NPEDGHNR	---W-MI---H------Q-QAFQKY
	Lichtheimia corymbifera JM.	CDH58321	------I--V---IPMLLQ	RSDEGESR	------I---H------K-MAFQ-Y
	Haemonchus contortus	CDJ83109	------I--V---IP-LLQ	NPQDGRNR	---WCMI---H------Q-QAFQKY
	Oncorhynchus mykiss	CDQ96838	------I--I---IP-LLQ	NPEDGYSR	---W-MI---H-----IK-MAFQKY
	Bos taurus	DAA25489	------I--I---IP-LLQ	NPQDGFSR	---W-MI---H-----IK-MAFQKY
	Caenorhabditis brenneri	EGT51377	------I--V---IP-LLQ	NPQDGHNR	---W-MI---H------Q-QAFQKY
	Capitella teleta	ELT95764	---S--I--I---IP-LLQ	NPKDGINR	---W-MI---H-----IK-MAFQK-
	Anopheles darlingi	ETN63350	------I--I---IP-LLQ	NPLDGYNR	---W-MI---H-----IK-MAFQKY
	Ancylostoma ceylanicum	EYC03617	------I--V---IP-LLQ	NPQDGRNR	---WCMI---H------Q-QAFQKY
	Anopheles sinensis	KFB41306	------I--I---IP-LLQ	NPLDGYNR	---W-MI---H-----IK-MAFQKY
	Toxocara canis	KHN88639	------I--V---IP-LLQ	NPQDGRSR	---W-MI---H------Q-VAFQKY
	Ancylostoma duodenale	KIH57401	------I--V---IP-LLQ	NPQDGRNR	---WCMI---H------Q-QAFQKY
	Larimichthys crocea	KKF14004	------I--I---IP-LLQ	NPEDGYSR	---W-MI---H-----IK-MAFQKY
	Sarcoptes scabiei	KPM03788	------I--I---IP-LLQ	NPDLGHNR	---W-MI---L-----IK-I-FQK-
	Alligator mississippiensis	KYO28749	------I--I---IP-LLQ	NPQDGFSR	---W-MI---H-----IK-MAFQKY
	Rattus norvegicus	NP_001094457	------I--I---IP-LLQ	NPQDGFSR	---W-MI---H-----IK-MAFQKY
	Caenorhabditis elegans	NP_001256831	------I--V---IP-LLQ	NPQDGHNR	---W-MI---H------Q-QAFQKY
	Mus musculus	NP_001277717	------I--I---IP-LLQ	NPQDGFSR	---W-MI---H-----IK-MAFQKY
	Hypsibius dujardini	OWA51009	------I--V---IP-LLQ	KPEHGHNR	---W-MI---N----I-Q-KVFEK-
	Onchocerca flexuosa	OZC12055	------I--V---IP-LLQ	NPQDGRSR	---W-MI---H------Q-IAFQKY
	Caenorhabditis remanei	OZF88549	------I--V---IP-LLQ	NPQDGHNR	---W-MI---H------Q-QAFQKY
	Oryctolagus cuniculus	XP_002718043	------I--I---IP-LLQ	NPQDGFSR	---W-MI---H-----IK-MAFQKY
	Ailuropoda melanoleuca	XP_002917855	------I--I---IP-LLQ	NPQDGFSR	---W-MI---H-----IK-MAFQKY
	Xenopus tropicalis	XP_002932889	------I--I---IP-LLQ	NPEDGFSR	---W-MI---H-----IK-MAFQKY
	Nomascus leucogenys	XP_003272843	------I--I---IP-LLQ	NPQDGFSR	---W-MI---H-----IK-MAFQKY

Figure S13. A partial sequence alignment of the Intron-binding aquarius beta like protein showing a five/eight amino acid deletion that is specific for members of *Piroplasmida* sans *B. microti*. The *Babesia divergens* sequence was acquired from a tblastn search conducted against the *Babesia bigemina* sequence.

			261		316
**Babesia (3/4)**	*Babesia bovis T2Bo*	XP_001608890	FWSSPWGEGRPGWHIECSAMCSNVFGK	DT	IIDIHSGGIDLKFPHHDNEIAQSEAFS
	Babesia bigemina	XP_012767680.1	--P----N--------------T----	-S	---------------------------
	Babesia ovata	GBE61326.1	--P----N--------------TV---	-S	-V------V------------------
	Babesia sp. Xin.	ORM40534	--D----N---------------I---	--	--------V---------------
	Babesia divergens	LK934717.1	T----------AS----	-S	-V------V------------------
**Theileria (0/4)**	*Theileria annulata*	XP_954780	S-E----R------------STCI---	E	VF---------R-------------YF
	Theileria parva str. Mug.	XP_763084	S-D----K------------STCI---	E	VF------V--R-------------YF
	Theileria orientalis str. Shi.	XP_009691837	S-D----P------------S-CI-E-	G	VF---------R-------------YF
	Theileria equi	XP_004833028	Y-E----L------------S-SI--D	S	VF-L-------R-------------YL
**B. microti**	*Babesia microti str. RI*	1206244458	--D----K----------V-A-HLL-D		K----------R-------V-----YF
**Plasmodium (0/20)**	*Plasmodium sp. DRC-Itaito*	SPJ10566.1	-D----K----------T-A--IL-D		VL---------R-------L------
	Plasmodium sp. Gor. Cla. G2	SOV14809.1	-D----K----------T-A--IL-D		VL---------R-------L------
	Plasmodium berghei ANKA	CDS49189	S-D----K----------T-A--IL--		VL------V--R-------L------F
	Plasmodium chabaudi ada.	SCM23052	S-D----K----------T-A--IL--		VL------V--R-------L------F
	Plasmodium chabaudi cha.	CAH76958	S-D----K----------T-A--IL--		VL------V--R-------L------F
	Plasmodium coatneyi	XP_019914392	Y-D----K----------T-A--IL-S		VL------V--R-------L------F
	Plasmodium cynomolgi str. B	XP_004222036	Y-D----K----------T-A--IL-N		-L------V--R-------L------F
	Plasmodium falciparum 3D7	CZT98403	H-D----K----------T-A--IL-D		VL---------R-------L------F
	Plasmodium falciparum HB3	KOB60818	H-D----K----------T-A--IL-D		VL---------R-------L------F
	Plasmodium falciparum Pa. Al.	ETW56568	H-D----K----------T-A--IL-D		VL---------R-------L------F
	Plasmodium fragile	XP_012337164	Y-D----K----------T-A--I--N		VL------V--R-------L------F
	Plasmodium gaboni	XP_018641535	N-D----K----------T-A--IL-D		VL---------R-------L------F
	Plasmodium gallinaceum	CRG94210	Y-D----K----------T-A--IL--		VV---------R-------L------F
	Plasmodium gonderi	GAW80575	Y-N----K----------T-A--IL-N		VL---------R-------L------F
	Plasmodium inui San Ant. 1	XP_008815940	Y-D----K----------T-A--IL-S		VL---------R-------L------F
	Plasmodium knowlesi str. H	SBO29533	Y-N----K----------T-A--IL-S		VL------V--R-------L------F
	Plasmodium malariae	SBS84520	Y-DC---K----------T-A--IL--		VL---------R-------L------F
	Plasmodium ovale curt.	SBS82865	Y-D----K----------T-A--IL--		VL---------R-------L------F
	Plasmodium ovale wal.	SBT50655	Y-D----K----------T-A--IL--		VL---------R-------L------F
	Plasmodium reichenowi	CDO64583	H-D----K----------T-A--IL-D		VL---------R-------L------F
	Plasmodium relictum	CRG99705	Y-D----K----------T-A--IL--		V----------R-------L------F
	Plasmodium vinckei pet.	EUD72541	S-D----K----------T-A--IL--		VL------V--R-------L------F
	Plasmodium vinckei vin.	XP_008623378	S-D----K----------T-A-KIL--		VL------V--R-------L------F
	Plasmodium vivax Ind. VII	KMZ80883	Y-D----K----------T-A--IL-S		VL------V--R-------L------F
	Plasmodium vivax Sal-1	XP_001614464	Y-D----K----------T-A--IL-S		VL------V--R-------L------F
	Plasmodium yoelii 17X	ETB62142	S-D----K----------T-A--IL-N		VL---------R-------L------F
	Plasmodium yoelii yoe. 17XNL	XP_724944	S-D----K----------T-A--IL-N		VL---------R-------L------F
**Other Apicom-plexa**	*Cyclospora cayetanensis*	1249176685	S-E-K--A------------A-SLLPF		PL-V-------R-------L--T--AK
	Eimeria acervulina	915008020	S-D-K--P------------A-SLL-F		PL------V--R-------L--T--AK
	Eimeria maxima	915123185	S-P-K--P------------A-SLLPF		PL---------R-------L--T--AK
	Eimeria tenella	916417628	S-D-K--P------------A-ALLPF		PL---------R-------L--T--AK
	Toxoplasma gondii ME49	1085154566	S-D----K------------ADSILPF		PL---------R-------L-----AN
	Besnoitia besnoiti	1261484150	S-D----K------------ADS-LPF		PL---------R-------L-----AA
	Cystoisospora suis	1268243837	S-D----K------------AESILPF		PL---------R-------L-----AN
	Hammondia hammondi	675133060	S-D----K------------ADSILPF		PL---------R-------L-----AN
	Neospora caninum Liv.	401412406	S-D----K------------ADSILPF		PL---------R-------L-----AN
	Cryptosporidium andersoni	1098428919	--D----K------------A--TL-F		P----------R-------L-----HY
	Cryptosporidium hominis TU502	67623595	C-D----K----------V-A--TL-F		P----------R-------L-----HY
	Cryptosporidium muris RN66	209877248	--D----K------------A--TL-F		P----------R-------L-----HY
	Cryptosporidium parvum Iowa II	66475306	C-D----K----------V-A--TL-F		P----------R-------L-----HY
	Cryptosporidium ubiquitum	1098424850	S-D----K----------V-A--TL-F		P----------R-------L-----HY
	Gregarina niphandrodes	749158971	--E-V--R------------A-DAL-F		P--V-------R-------L--T--NF
**Other Eukarya**	*Pneumocystis murina B123*	XP_007873417	K-E----K------------A-EIL-S		E----------A--------------F
	Pneumocystis jirovecii RU7	XP_018231044	A-D----R------------AAEIL-S		E----------A------------G-F
	Anaeromyces robustus	ORX79809	Q-E----Y----------V-A-R-L-S		N----------A-------------HF
	Acanthisitta chloris	KFP77156	S-D----K------------AGSIL-E		SM---G--F--R-------L-----YF
	Acyrthosiphon pisum	XP_016661759	W-D----K----------V-A-CIL-Q		TL---T----------------A--YY
	Drosophila navojoa	XP_017958637	W-D----R------------A-DI--S		-F---T--V----------L-----AF
	Folsomia candida	XP_021963310	S-D----K------------A-AIV--		EL---T--V----------------YY
	Ictalurus punctatus	XP_017314790	S-D----K------------AGSIL-S		SM---G--F--R-------L-----YF
	Kryptolebias marmoratus	XP_017296147	S-D----K------------AGSIL-E		SM---G--F--R-------L------F
	Larimichthys crocea	XP_019131694	S-D----K------------AGSIL-E		SM---G--F--R-------L------F
	Lates calcarifer	XP_018548230	S-D----K------------AGSIL-E		SM---G--F--R-------L-----YF
	Latimeria chalumnae	XP_014344864	S-D----K------------AGSIL-E		SM---G--F--R-------L-----YF

Figure S14. A partial sequence alignment of the Cysteinyl-tRNA synthetase protein showing a two amino acid insertion that is specific for members of *Babesia* (sans *B. microti*) and a one amino acid insertion that is specific for members of *Theileria*. The *Babesia divergens* sequence was acquired from a tblastn search conducted against the *Babesia bovis T2Bo* sequence.

Group	Species	Accession	225		279
***Theileria* (4/4)**	*Theileria parva str. Mug.*	XP_764692	SPKRILVKKDELTLEGIKQFYILIDKE		YKFETLCDLYESVTITQAIIYCNTRRKV
	Theileria annulata	XP_952588	---------------------------		----------------------------
	Theileria orientalis str. Shi.	XP_009690141	---------------------V--E-D		---D------------------------
	Theileria equi	XP_004828669	---------------------VMV--D		----------------------------
***Babesia* (0/4)**	*Babesia ovata*	GBE60951.1	---------------------VM----	E	---D------------------------
	Babesia sp. Xin.	ORM41817	---------------------VM----	E	---D------------------------
	Babesia bovis T2Bo	XP_001612149	---------------------VM----	E	---D------------------------
	Babesia bigemina	XP_012766066	---------------------VM----	E	F--D------------------------
	Babesia microti str. RI	XP_021337506	-------------------Y-VS-E--	E	W-------I--T----------------
***Plasmodium* (0/20)**	*Plasmodium sp. DRC-Itaito*	SOV25406.1	--T-------------R---VAVE--	E	W-LD-------TL----S-------K--
	Plasmodium sp. Gor. Cla. G2	SOV19866.1	--T-------------R---VAVE--	E	W-LD-------TL----S-------K--
	Plasmodium gonderi	GAW82398.1	--T-------------R---VAVE--	E	W-LD-------TL----S-------K--
	Plasmodium gaboni	XP_018639738.1	--T-------------R---VAVE--	E	W-LD-------TL----S-------K--
	Plasmodium cynomolgi str. B	XP_004223844.1	--T-------------R---VAVE--	E	W-LD-------TL----S-------K--
	Plasmodium yoelii yoe.	23479359	D--T-------------R---VAVE--	E	W-LD-------TL----S-------K--
	Plasmodium vivax Sal-1	156099810	D--T-------------R---VAVE--	E	W-LD-------TL----S-------K--
	Plasmodium vinckei vin.	669194458	D--T-------------R---VAVE--	E	W-LD-------TL----S-------K--
	Plasmodium vinckei pet.	577147321	D--T-------------R---VAVE--	E	W-LD-------TL----S-------K--
	Plasmodium relictum	1102625089	D--T-------------R---VAVE--	E	W-LD-------TL----S-------K--
	Plasmodium reichenowi	1145263932	D--T-------------R---VAVE--	E	W-LD-------TL----S-------K--
	Plasmodium ovale wal.	1037148027	D--T-------------R---VAVE--	E	W-LD-------TL----S-------K--
	Plasmodium ovale curt.	1036546959	D--T-------------R---VAVE--	E	W-LD-------TL----S-------K--
	Plasmodium malariae	1037138671	D--T-------------R---VAVE--	E	W-LD-------TL----S-------K--
	Plasmodium knowlesi str. H	221059063	D--T-------------R---VAVE--	E	W-LD-------TL----S-------K--
	Plasmodium inui San Ant. 1	672184964	D--T-------------R---VAVE--	E	W-LD-------TL----S-------K--
	Plasmodium gallinaceum	1103665590	D--T-------------R---VAVE--	E	W-LD-------TL----S-------K--
	Plasmodium fragile	817746352	D--T-------------R---VAVE--	E	W-LD-------TL----S-------K--
	Plasmodium falciparum Viet.	574747298	D--T-------------R---VAVE--	E	W-LD-------TL----S-------K--
	Plasmodium falciparum Tan.	574970895	D--T-------------R---VAVE--	E	W-LD-------TL----S-------K--
	Plasmodium falciparum NF135/5.	574977702	D--T-------------R---VAVE--	E	W-LD-------TL----S-------K--
	Plasmodium falciparum FCH/4	574965696	D--T-------------R---VAVE--	E	W-LD-------TL----S-------K--
	Plasmodium falciparum 3D7	124810293	D--T-------------R---VAVE--	E	W-LD-------TL----S-------K--
	Plasmodium coatneyi	1139866017	D--T-------------R---VAVE--	E	W-LD-------TL----S-------K--
	Plasmodium chabaudi cha.	56503927	A--T-------------R---VAVE--	E	W-LD-------TL----S-------K--
	Plasmodium berghei ANKA	1269289602	D--T-------------R---VAVE--	E	W-LD-------TL----S-------K--
**Other Apicom-plexa**	*Besnoitia besnoiti*	1261478019	D-------N--------R---VAVE--	D	W-L--------TL---------------
	Cyclospora cayetanensis	1249143268	N-F-V---R-----------FVAVER-	Q	W--D--T---DTL-----V-F---KT--
	Cystoisospora suis	1268226509	D-------N--------R---VAVE--	D	W-L--------TL---------------
	Eimeria acervulina	915002702	N-F-V---R-----------FVAVER-	H	W--D--T---DTL-----VVF---KT--
	Eimeria brunetti	557212117	K-R--------------A-Y--DVQ--	E	N-----V----TL-------------R-
	Eimeria maxima	915134915	K----------------A-Y--DVQ--	E	N--D--V----TL-------------R-
	Eimeria mitis	916510821	K-R--------------A-Y--DVQR-	E	N--D--V----TL-------------R-
	Eimeria necatrix	921131014	N-F-V---R-----------FVAVER-	H	W-----T---DTL-----VVF---KT--
	Eimeria tenella	357017209	K----------------A-YF-DVQR-	E	N--D--V----TL---------------
	Hammondia hammondi	675133256	D-------N--------R---VAVE--	D	W-L--------TL---------------
	Neospora caninum Liv.	XP_003886231	D-------N--------R---VAVE--	D	W-L--------TL---------------
	Toxoplasma gondii ME49	XP_002367359	D-------N--------R---VAVE--	D	W-L--------TL---------------
	Cryptosporidium ubiquitum	1098426432	D-------QE-------R---VGVE-D	E	W-MD--I----TL--V----------R-
	Cryptosporidium parvum	409710284	D-------QE-------R---VGVE-D	E	W-MD--I----TL--V----------R-
	Cryptosporidium muris RN66	209880596	D-------QE-------R-Y-VAVE-D	E	W-LA--V----TL--V---------IR-
	Cryptosporidium hominis TU502	67613960	D-------QE-------R---VGVE-D	E	W-MD--I----TL--V----------R-
	Cryptosporidium andersoni	1098423208	D-------QE-------R-Y-VAVE-D	E	W-LA--V----TL--V---------IR-
	Gregarina niphandrodes	749154387	D-FKV--NR--------R--FVSVE--	A	W--D--T---DTLV----VVF---TE--
**Other Eukarya**	*Fistulifera solaris*	GAX24517	E-V-------------------AV---	E	W-LD-------TL---------------
	Fragilariopsis cylindrus CCMP.	OEU21820	E-I-------------------AV-R-	E	W-L--------TL---------------
	Phaeodactylum tricornutum CCAP.	XP_002186399	E-V-------------------SV---	D	W-L--------TL---------------
	Blastocystis sp. subtype 4	XP_014529783	D-V------------------VPLE--	S	W--D-------FA-A-------------
	Blastocystis hominis	XP_012898360	D-V------------------VEM---	S	W----------I--A-------------
	Plasmodiophora brassicae	CEP00240	D-V-----R------------VAVE--	D	W-LD-------TL---------------
	Salpingoeca rosetta	XP_004997619	D-V-V---R-----------FVAVER-	E	W--D------DTL-----V-F-------
	Acanthamoeba castellanii str.	XP_004341467	D-I-----R-----------FVAVE--	E	W---------DTL-----V-F-------
	Jaapia argillacea MUCL 33604	KDQ61301	E-V-----R-------------AVE--	E	W-LD-------T------V-F-------
	Microdochium bolleyi	KXJ95584	D-I-------------------AVE--	E	W-LD--S----T------V-F-------
	Calocera cornea HHB12733	KZT62815	D-I-----R-----------FVAVE--	E	W--D------DTL-----V-F-------
	Moniliophthora roreri	KTB38054	D-V-----R-------------AVE--	E	W-LD-------T------V-F-------

Figure S15. A partial sequence alignment of the Eukaryotic translation initiation factor 4a protein showing a one amino acid deletion that is specific for members of *Theileria*.

Group	Species	Accession	50		103
***Plasmodium* (20/20)**	*Plasmodium berghei ANKA*	CDS50468	IRATRTREVLGDKGRRIRELTSLVQKR	F	FNKLTNSVELFAERVENRGLCAMAQA
	Plasmodium sp. DRC-Itaito	SOV25378.1	---------------------------	-	---S------------H---------
	Plasmodium sp. Gor. Cla. G2	SOV19809.1	---------------------------	-	---S------------H---------
	Plasmodium knowlesi str. H	XP_002260207.1	---------------------------	-	---S------------H---------
	Plasmodium gaboni	XP_018639713.1	---------------------------	-	---S------------H---------
	Plasmodium cynomolgi str. B	XP_004223872.1	---------------------------	-	---S------------H---------
	Plasmodium coatneyi	XP_019916126.1	---------------------------	-	---S------------H---------
	Plasmodium chabaudi cha.	XP_016654586	---------------------------	-	---S----------------------
	Plasmodium falciparum 3D7	XP_001348801	---------------------------	-	---S------------H---------
	Plasmodium falciparum FCH/4	ETW27535	---------------------------	-	---S------------H---------
	Plasmodium falciparum IGH-CR14	KNG76271	---------------------------	-	---S------------H---------
	Plasmodium fragile	XP_012337015	---------------------------	-	---S------------H---------
	Plasmodium gallinaceum	CRG94856	---------------------------	-	---S------------H---------
	Plasmodium gonderi	GAW82426	---------------------------	-	---S------------H---------
	Plasmodium inui San Ant. 1	XP_008813962	---------------------------	-	---S------------H---------
	Plasmodium malariae	SBT00403	---------------------------	-	---S------------H---------
	Plasmodium ovale curt.	SBS86392	---------------------------	-	---S------------H---------
	Plasmodium ovale wal.	SBT42083	---------------------------	-	---S------------H---------
	Plasmodium reichenowi	XP_012765701	---------------------------	-	---S------------H---------
	Plasmodium relictum	CRH01265	---------------------------	-	---S------------H---------
	Plasmodium vinckei pet.	EUD70471	---------------------------	-	---S----------------------
	Plasmodium vinckei vin.	XP_008622214	---------------------------	-	---S----------------------
	Plasmodium vivax Sal-1	XP_001615729	---------------------------	-	---S------------H---------
	Plasmodium yoelii 17X	ETB63049	---------------------------	-	---S----------------------
***Piroplasmida* (0/8)**	*Babesia bigemina*	XP_012767181	-K---A---V-E-A-------------		--FSPDT---Y-----HK--------
	Babesia bovis T2Bo	XP_001609690	-----A---V-E-A---------I---		-GFSPDT---Y-----HK--------
	Babesia microti str. RI	XP_012648260	-----------E---------------		-GFAPD----Y---I-H---------
	Babesia sp. Xinjiang	ORM41413	-----A---V---A-------------		-GFSPDT---Y-----HK--------
	Babesia ovata	GBE61796.1	-K---A---V-E-A-------------		--FSPDT---Y-----HK--------
	Theileria equi	XP_004831148	-----------E-A-------------		-GFSSD----Y---------------
	Theileria orientalis str. Shi.	XP_009692420	-----------E-A-------------		-GFSSD----Y---------------
	Theileria parva str. Mug.	XP_764056	-----------E-A-------------		-GFSSD----Y---------------
	Theileria annulata	XP_953083	-----------E-A-------------		-GFSSD----Y---------------
**Other Apicom-plexa**	*Besnoitia besnoiti*	PFH38580	----------------------V----		-GFAPG----------H---------
	Cyclospora cayetanensis	XP_022592372	-----------E---------------		-GFPPD--------------------
	Cystoisospora suis	PHJ24329	----------------------V----		-GFAPD--------------------
	Eimeria brunetti	CDJ53616	-----------E---------------		-AFAPD--------------------
	Eimeria necatrix	XP_013440444	-----------E---------------		-GFPAD--------------------
	Eimeria tenella	XP_013230536	-----------E---------------		-GFPPD--------------------
	Hammondia hammondi	XP_008885620	----------------------V----		-GFAPD--------------------
	Neospora caninum Liv.	XP_003883474	----------------------V----		-GFAPD--------------------
	Toxoplasma gondii ME49	XP_018636818	----------------------V----		-GFAPD--------------------
	Cryptosporidium andersoni	OII75791	-----------V---------------		--FPAG--------I-----------
	Cryptosporidium hominis TU502	XP_667397	-----------V-----------I---		--FPEG--------I-----------
	Cryptosporidium muris RN66	XP_002141393	-----------V---------------		--FPAG--------I-----------
	Cryptosporidium parvum Iowa II	XP_628399	-----------V-----------I---		--FPEG--------I-----------
	Cryptosporidium ubiquitum	OII74098	-----------V-----------I---		--FPEG--------I-----------
**Other Eukarya**	*Chlamydomonas incerta*	ABA01099	------QN---E----------V----		--FPPD----Y--K-SD-----I---
	Theobroma cacao	EOY30019	------QN---E----------V----		-KFPE-----Y--K-N------I---
	Klebsormidium nitens	GAQ80594	------QN---E----------V----		--FPE-T---Y--K-A------I---
	Cephalotus follicularis	GAV81193	------QN---E----------V----		-KFAE-----Y--K-N------I---
	Cynara cardunculus var. sco.	KVH95611	------QN---E---------------		-KFPE-----Y----N------I---
	Cajanus cajan	KYP74032	------QA---E----------V----		-KFPE-----Y--K-N------I---
	Dorcoceras hygrometricum	KZV30851	------QN---E----------V----		-KFPE-----Y--K-N------I---
	Glycine soja	KHN24447	------QA---E----------V----		-KFPE-----Y--K-N------I---
	Gossypium arboreum	KHG00767	------QN---E----------V----		-KFPE-----Y--K-N------I---
	Citrus sinensis	KDO35864	------QN---E----------V----		-KFPE-----Y--K-N------I---
	Zostera marina	KMZ69355	------QN---E----------V----		--FPE-----Y--K-N------T---
	Arabidopsis thaliana	NP_198403	------QN---E---------------		-KFPQD----Y--K-A------I---
	Macleaya cordata	OVA08366	------QN---E----------V----		-KFPE-----Y--K-N------I---
	Gossypium raimondii	XP_012480014	------QN---E----------V----		-KFPE-----Y--K-N------I---
	Phalaenopsis equestris	XP_020578106	------QS---E----------I----		-KFPE-----Y--K-N------I---
	Gossypium hirsutum	XP_016719678	------QN---E----------V----		-KFPE-----Y--K-N------I---
	Jatropha curcas	XP_012077022	------QN--------------V----		-KFPE-----Y--K-NS-----I---

Figure S16. A partial sequence alignment of the 40S ribosomal protein S3 showing a one amino acid insertion that is specific for members of *Plasmodium*. Expanded alignment of Figure 4 (A).

			777		826
***Plasmodium* (20/20)**	*Plasmodium berghei ANKA*	CDS50476	DAIIRYGGMFTIALAYCGLSN	YN	KHIIKKLLHFSVSDVSDDVRRAAVIAL
	Plasmodium sp. Gor. Cla. G2	SOV19817.1	----------A-GM-------	C-	---------------------------
	Plasmodium sp. DRC-Itaito	SPJ13024.1	----------A--M-------	C-	---------------------------
	Plasmodium chabaudi ada.	SCM12490	---------------------	--	--V------------------------
	Plasmodium chabaudi cha.	CAH77954	---------------------	--	--V------------------------
	Plasmodium coatneyi	XP_019916771	-------------M------S	--	-----R---------------------
	Plasmodium cynomolgi str. B	XP_004223868	-------------M------S	--	-----R---------------------
	Plasmodium falciparum 3D7	XP_001348806	----------A--M-------	C-	---------------------------
	Plasmodium falciparum Dd2	KOB87484	----------A--M-------	C-	---------------------------
	Plasmodium falciparum FCH/4	ETW27531	----------A--M-------	C-	---------------------------
	Plasmodium falciparum IGH-CR14	KNG78484	----------A--M-------	C-	---------------------------
	Plasmodium falciparum Mal.	ETW46601	----------A--M-------	C-	---------------------------
	Plasmodium falciparum NF135/5.	ETW39578	----------A--M-------	C-	---------------------------
	Plasmodium falciparum Pal. Alt.	ETW54328	----------A--M-------	C-	---------------------------
	Plasmodium falciparum Tan.	ETW33014	----------A--M-------	C-	---------------------------
	Plasmodium falciparum UGT5.1	EWC73529	----------A--M-------	C-	---------------------------
	Plasmodium falciparum Vie.	ETW15853	----------A--M-------	C-	---------------------------
	Plasmodium fragile	XP_012338224	-------------M------S	--	-----R---------------------
	Plasmodium gaboni	XP_018639717	----------A-GM-------	C-	---------------------------
	Plasmodium gallinaceum	CRG94860	---------------------	--	---------------------------
	Plasmodium gonderi	GAW82422	-------------M------S	--	-----M---------------------
	Plasmodium inui San Ant. 1	XP_008813958	-------------M------S	--	-----R---------------------
	Plasmodium knowlesi str. H	XP_002260203	-------------M------S	--	-----R---------------------
	Plasmodium malariae	SBS88379	---------------------	--	---------------------------
	Plasmodium ovale curt.	SBS86380	---------------------	--	---------------------------
	Plasmodium ovale wal.	SBT42057	---------------------	--	---------------------------
	Plasmodium reichenowi	CDO67127	----------A--M-------	C-	---------------------------
	Plasmodium relictum	CRH01261	---------------------	--	---------------------------
	Plasmodium vinckei pet.	EUD70467	--------------------S	--	--V------------------------
	Plasmodium vinckei vin.	XP_008622218	--------------------S	--	--A------------------------
	Plasmodium vivax Bra. I	KMZ85080	-------------M------S	--	-----R---------------------
	Plasmodium vivax Ind. VII	KMZ78690	-------------M------S	--	-----R---------------------
	Plasmodium vivax Mau. I	KMZ91539	-------------M------S	--	-----R---------------------
	Plasmodium vivax North Kor.	KMZ98056	-------------M------S	--	-----R---------------------
	Plasmodium vivax Sal-1	XP_001615725	-------------M------S	--	-----R---------------------
	Plasmodium yoelii 17X	ETB63041	---------------------	--	---------------------------
	Plasmodium yoelii yoe. 17XNL	XP_730477	---------------------	--	---------------------------
***Piroplasmida* (0/8)**	*Babesia bigemina*	833484190	-PVV-------Y-M----TGS		SKAL-E--YAA----------------
	Babesia bovis T2Bo	156084408	-PVV------AY-M----TGS		SKVV-A--YA---------------S-
	Babesia microti str. RI	1206244494	-D---M---YM-G----ST-I		SFAVN----HA------N---S---C-
	Babesia ovata	1314814536	-PVV-------Y-M----TGS		SKAL-D--YAA----------------
	Babesia sp. Xin.	ORM41447	-PVV------AY-M----TGS		SKA--E--YA---------------S-
	Theileria annulata	84996729	-S--------CY-M----TGS		SYAV-Q--YSA--------------S-
	Theileria equi	510905315	-S--------CY-M----TGS		TQA--D--YAA---------------I
	Theileria orientalis str. Shi.	697894357	----------CY-M----TGS		STAV-H--YSA--------------S-
	Theileria parva str. Mug.	XP_764059	----------CY-M----TGS		SYAV-Q--YAA--------------S-
**Other Apicomplexa**	*Besnoitia besnoiti*	1261484317	-PL----------M---ATA-		SSA-RR---V-----------------
	Cyclospora cayetanensis	1249161994	-PL-----V-A-G----ATGK		-RAVER---LG-A--N--------LS-
	Cystoisospora suis	1268241452	--L----------M----TA-		SSA-RR---V-----N---------S-
	Eimeria necatrix	921123295	-SL-----A-A-G----NTGK		ESAV-R---IA-------------LS-
	Eimeria tenella	916417598	-SL-----A-A-G----NTGK		ESAV-R---IA-------------LS-
	Hammondia hammondi	675121852	--L----------M---TTA-		SGA-RR---V-----------------
	Neospora caninum Liv.	401406560	--L----------M---ATA-		SSA-RR---V-----------------
	Toxoplasma gondii ME49	237832217	--L----------M---ATA-		SGA-RR---V-----------------
	Cryptosporidium andersoni	1098425167	EHF----SLYVLG-----TG-		EFALE----SC--EL---N-----F--
	Cryptosporidium hominis TU502	67595029	EHF----A-HVLG-----TGS		QFAME---QII--EL---NK----F--
	Cryptosporidium muris RN66	209882999	EHF----SLYVLG-----TG-		EFALE----SC--EL---N-----F--
	Cryptosporidium parvum Iowa II	66356476	EHF----A-HVLG-----TG-		QFAME---QII--EL---NK----F--
	Cryptosporidium ubiquitum	1098424451	EHF----A-HVLG-----TG-		EFAME----A---EL---NK----F--
	Gregarina niphandrodes	749154645	-PLV----C-AL----V-TGR		QAALRT---IA---PV----Q----SV
**Other Eukarya**	*Thecamonas trahens ATCC 500.*	XP_013752871	--LL-----Y---M----T--		NRAVR---RIA-------------T--
	Stentor coeruleus	OMJ68245	-P-L-----Y-M-T--I-TA-		TNVV------A----DY---K---T-I
	Pneumocystis jirovecii	CCJ30008	-P-L----IY---M----TE-		NKA--R---TA----N------S--S-
	Cryptococcus gattii VGII R2.	KGB79043	---L-----------FA-TG-		NKAV-----IA----N--------T--
	Malus domestica	XP_008380574	-P-L-----YAL----S-TA-		NKA-RQ----A----------T--L--

Figure S17. A partial sequence alignment the 26S proteasome regulatory subunit RPN2 showing a two amino acid insertion that is specific for members of *Plasmodium.*

Group	Organism	Accession	64		116
**Plasmodium (20/20)**	*Plasmodium berghei str. ANKA*	XP_673015	LLKLERIKDYLLLEEEFITNQEQIKTTDDKNYGKL		KIDDLRGSPMSVGTLEEL
	Plasmodium chabaudi cha.	XP_016654176.1	-----------------------------------		------------------
	Plasmodium sp. gorilla clade G2	SOV14673.1	----------------Y--------SS-----V--		------------------
	Plasmodium sp. DRC-Itaito	SOV22808.1	----------------Y--------SS-----V--		------------------
	Plasmodium coatneyi	XP_019914663	---------------------------E----V--		------------------
	Plasmodium cynomolgi str. B	XP_004221963	---------------------------E----V--		------------------
	Plasmodium falciparum 3D7	XP_001347366	----------------Y--------SS-----V--		---------V--------
	Plasmodium falciparum Dd2	KOB85505	----------------Y--------SS-----V--		---------V--------
	Plasmodium falciparum HB3	KOB60869	----------------Y--------SS-----V--		---------V--------
	Plasmodium falciparum IGH.	KNG77494	----------------Y--------SS-----V--		---------V--------
	Plasmodium falciparum NF54	EWC88460	----------------Y--------SS-----V--		---------V--------
	Plasmodium falciparum Pa. Al.	ETW56499	----------------Y--------SS-----V--		---------V--------
	Plasmodium falciparum Vie.	ETW18248	----------------Y--------SS-----V--		---------V--------
	Plasmodium fragile	XP_012335633	---------------------------E----V--		------------------
	Plasmodium gaboni	XP_018641467	----------------Y--------SS-----V--		------------------
	Plasmodium gallinaceum	CRG94141	----------------Y---------------V--		-----------------M
	Plasmodium gonderi	GAW80507	--------------------------------V--		------------------
	Plasmodium inui San Ant. 1	XP_008816013	-----------------------------------		------------------
	Plasmodium knowlesi str. H	XP_002258715	---------------------------E----V--		------------------
	Plasmodium malariae	SBS84332	--------------------------------V--		------------------
	Plasmodium ovale curt.	SBS82811	--------------------------------V--		------------------
	Plasmodium ovale wal.	SBT34013	--------------------------------V--		------------------
	Plasmodium reichenowi	XP_012763121	----------------Y--------SS-----V--		------------------
	Plasmodium relictum	CRG99635	----------------Y---------------V--		------------------
	Plasmodium vinckei pet.	EUD72611	-----------------------------------		------------------
	Plasmodium vinckei vin.	XP_008623308	-----------------------------------		------------------
	Plasmodium vivax Sal-1	XP_001614391	---------------------------E----V--		------------------
	Plasmodium yoelii yoe. 17XNL	XP_726782	-----------------------------------		------------------
**Piroplasmida (0/8)**	*Theileria annulata*	84998430	----------------Y---KSLH-PLKT--QDD-	I	-L--I------------I
	Theileria equi	510910518	----------------YL--KSLH-PVKA-TQDD-	I	-L--I------------I
	Theileria orientalis str. Shi.	697888786	----------------Y---KSLN-LLKT--QDD-	I	-L---------------I
	Theileria parva str. Mug.	71033449	----------------Y---KSLH-PLKT--QDD-	I	-L--I------------I
	Babesia ovata	GBE60464.1	----------------Y-A-KIRLNP-KN--QDDM	L	RLE-----------M--M
	Babesia bigemina	833482209	----------------Y-A-KIRLNP-KN--QDDM	L	RLE-----------M--M
	Babesia bovis T2Bo	156086888	----------------YVA-KIRLNP-KN--QDD-	L	RLE-----------M--M
	Babesia microti str. RI	1206245763	-Q--------------Y---RDRL-PAAK-KQEAR	M	-V--------G--V---I
	Babesia sp. Xin.	1181627200	----------------YVA-KIRLNP-KN--QDD-	V	RLE-----------M--M
**Other Apicom-plexa**	*Eimeria brunetti*	CDJ52010	------V---------Y-S----H-PAEER-EEEV	S	RV-E-----L--------
	Eimeria tenella	XP_013230097	------V---------Y-S----H-PAEER-EEEV	S	RV-E-----L--------
	Cyclospora cayetanensis	XP_022593180	------V---------Y-S---HH-PAEER-EEEV	S	RV-E-----L--------
	Cystoisospora suis	PHJ18309	----------------Y------R-PAEE--EEDV	T	RV-E---T-L---N---M
	Toxoplasma gondii ME49	237838979	--R-------------Y-L----R-PAEE--EEDV	N	RV-E-----L---N---I
	Hammondia hammondi	675133160	--R-------------Y-L----R-PAEE--EEDV	N	RV-E-----L---N---I
	Neospora caninum Liv.	401400795	--R-------------Y------R-PAEE--EEDV	N	RV-E-----L---N---I
	Cryptosporidium andersoni	1098423701	------V-----M-Q----TM-S-RPSAERTEEEH	T	-V---------I-N---I
	Cryptosporidium hominis	1129250869	---Y-------MM-Q----SM-SV-PSAETAEEEH	N	-V--------NI-----I
	Cryptosporidium muris RN66	209879531	------V-----M-Q----TM-S-RPSAERTEEEH	T	-V---------I-N---I
	Cryptosporidium parvum Iowa II	66357072	---Y-------MM-Q----SM-SV-PSAETAEEEH	N	-V--------NI-----I
	Cryptosporidium ubiquitum	1098423980	---Y-------MM-Q----SM-SV-PSAETAEEEH	N	-V--------NI-----I
**Other Eukarya**	*Acanthisitta chloris*	XP_009073865	------------M-----R----M-PLEE-QEEER	S	-V-----T---------I
	Acromyrmex echinatior	EGI70823	---M--------M-----R---RL-PQEE--EEER	S	-V-----T---------I
	Acropora digitifera	XP_015780035	------------------Q---RL-PQEE--EEER	S	-V-----T---------I
	Aedes aegypti	XP_001655853	------------M-----R---RL-PQ-E--EEER	S	-V-----T-----N---I
	Aethina tumida	XP_019880545	----------A-M--Q--Q---RF-PQEE--EEER	V	-V-----T---------I
	Dendroctonus ponderosae	ERL96126	---M--------M-----R---RL-PQEE--EEER	S	-V-----T-----N---I
	Diachasma alloeum	XP_015120981	------------M-----R---RL-PQEE--EEER	S	-V-----T---------I
	Diaphorina citri	XP_008477211	------------M-----R---RL-PQEE--EEER	S	RV-----T---------I
	Dinoponera quadriceps	XP_014467663	------------M-----R---RL-PQEE--EEER	S	-V-----T---------I

Figure S18. A partial sequence alignment of the 26S proteasome regulatory subunit 4 protein showing a one amino acid deletion that is specific for members of *Plasmodium*.

			35		79
***Plasmodium* (18/20)**	*Plasmodium falciparum 3D7*	XP_001348379	KLNHAVFIDKSLHSKILECKNMK		VITPSAIAEKYKVNLSVARAVI
	Plasmodium falciparum 7G8	EUR64092	-----------------------		----------------------
	Plasmodium falciparum CAMP.	ETW58996	-----------------------		----------------------
	Plasmodium falciparum Dd2	KOB87436	-----------------------		----------------------
	Plasmodium falciparum FCH/4	ETW29201	-----------------------		----------------------
	Plasmodium falciparum HB3	KOB61420	-----------------------		----------------------
	Plasmodium falciparum IGH-CR14	KNG76153	-----------------------		----------------------
	Plasmodium falciparum NF135/5.	ETW40076	-----------------------		----------------------
	Plasmodium falciparum Pal. Alt.	ETW53855	-----------------------		----------------------
	Plasmodium falciparum RAJ116	KNC38061	-----------------------		----------------------
	Plasmodium falciparum San. Lu.	EUT74966	-----------------------		----------------------
	Plasmodium reichenowi	XP_019969942.1	-----------------------		----------------------
	Plasmodium inui San Ant. 1	XP_008817456.1	----------A-Q-----S----		-----T-S-----------S--
	Plasmodium berghei	CXI53213	------------Q-----VQ---		-----T-SD----------S--
	Plasmodium chabaudi ada.	SCM10842	------------Q-----VQ---		-----T-SD-------------
	Plasmodium chabaudi cha.	SCM02475	------------Q-----VQ---		-----T-SD-------------
	Plasmodium coatneyi	XP_019917360	----------A-Q-----S----		-----T-S-----------S--
	Plasmodium cynomolgi str. B	XP_004224433	----------A-Q-----S----		-----T-S-----------S--
	Plasmodium fragile	XP_012338706	----------A-Q-----S----		-----T-S-----------S--
	Plasmodium gallinaceum	CRG95726	---------T------------		-----T-S--------------
	Plasmodium gonderi	GAW83009	----------A-Q-----S----		-----T-S-----------S--
	Plasmodium knowlesi str. H	SBO22619	----------A-Q-----S--L-		-----T-S-----------S--
	Plasmodium malariae	SBT00621	---------A------------		-----S-S--------------
	Plasmodium ovale wal.	SBT45555	-------------A---------		-----S-S-----------S--
	Plasmodium relictum	CRH03930	------------Y----D-----		-----T-S--------------
	Plasmodium vinckei pet.	EUD73802	------------Q-----VQ---		-----T-SD----------S--
	Plasmodium vinckei vin.	XP_008623940	------------Q-----VQ---		-----T-SD----------S--
	Plasmodium vivax Sal-1	XP_001616748	----------A-Q-----S----		-----T-S-----------S--
	Plasmodium yoelii 17X	ETB59629	------------Q-----VQ---		-----T-SD----------S--
**Other Apicomplexa**	*Babesia bovis T2Bo*	XP_001610942	-A-----F--ATYD-LMSDIPKA	R	L--I-T-VD-L---A-M--
	Babesia microti str. RI	XP_012648836	------IFE-ATYD-L-SEIPKS	K	L----V-C-RL---A-L--LA-
	Eimeria tenella	XP_013233041	---Y--MF-AGTLE-F-SEVPKM	K	LV--FS-C-RFRI-A-L--RG-
	Besnoitia besnoiti	PFH38670	------LF--ATYD-L-SEIPKA	R	L---HTVC-RL---A-L--QA-
	Cryptosporidium andersoni	OII76028	-A----LF--TTLERLSVDIMKL	K	M---AV-VNQL---A-A---AL
	Cryptosporidium muris RN66	XP_002140787	-A----LF--TTLERLSVDIMKL	K	M---AV-VNQL---A-A---AL
	Neospora caninum Liv.	XP_003883396	----S-LF--ATYD-L-ADIPKA	R	L---YTVC-RL---A-L--QA-
**Other Eukarya**	*Helicosporidium sp. ATCC 50.*	KDD76826	-V-NQ-LF--ATYE-LNGEVPKY	K	M----ILSDRLRI-G-L---A-
	Gonium pectorale	KXZ55502	-V-N--LF--AVYE-L-NEVPKY	K	M-S--VL-DRLRIGG-L---A-
	Chlamydomonas reinhardtii	XP_001694225	-V-N--LF--AVYE-L-NEVPKY	K	M-S--VL-DRLRIGG-L---A-
	Volvox carteri f. nag.	XP_002953072	-V-N-ILF--AVYE-L-NEVPKY	K	M-S--VL-DRLRI-G-L---A-
	Chlorella variabilis	XP_005843938	-S-NM-LF-AATYE-L-SEVPKY	K	M----VLSDRLRI-G-L---A-
	Auxenochlorella protothecoides	XP_011397629	-V-NQ-LF--ATYD-LQNEVPKY	K	L----VLSDRLR--G-L---A-
	Vitrella brassicaformis CCMP.	CEM30526	-A-N--MF--ETFD-LMKEIPKA	K	L---AI---RL---G-L---A-
	Stylonychia lemnae	CDW83774	---N---V-QK-YDR-VKEAPKF	L	NL-V-IVSD-F---GA---K-L
	Pfiesteria piscicida	ABI14382	--ANL-MF--ATYD-MMKEIPKA	K	L----VVS-RL---G-----A-
	Karlodinium veneficum	ADV03034	--ANL-MF--ATYD-L-KEIPKA	K	L----VVS-RL---G----QA-
	Amphidinium carterae	ADV03065	--ANM-MF--PTYE-MMKEIPKA	K	L----VVS-RL---G-----A-
	Acanthamoeba castellanii str.	XP_004352881	---NL-LF-QATYE-L-KEVPTY	K	L----V-S-RL-I-G-L--RA-
	Mortierella verticillata NR.	KFH62414	-A-N--IL--TTYD-LMKEVPTY	K	L-S--VLVDRLRI-G-L---A-
	Gonapodya prolifera JEL.	KXS21182	-A-N--T---A-YD-LVKEVPTY	K	L----VLVDRMR--G-I--K--
	Malassezia sympodialis ATCC.	XP_018739792	-AQN--HL--P-YE-VIKEVPTF	K	M----VLIDRL-I-G----LA-
	Lysiphlebus testaceipes	AAX62463	---N--LF--GTYD-L-KEVPQY	K	L----IVS-RL-IRG-L--RAL
	Ixodes scapularis	AAY66882	---NL-LF--ATYD-L-KEVPSY	K	L----VVS-RL--RG-L--KAL
	Barentsia elongata	ABW90430	---NL-LF--PTYD-LYKEVPNY	K	L----VVS-RL--KG-L--SAL
	Callithrix jacchus	ABZ80203	---NL-LF--ATYD-LCKEVPNY	K	L---AVVS-RL-IQG-L---AL
	Salmo salar	ACI68348	---NL-LF--ATYE-LNKEVPNY	K	L---AVVS-RL-IRG-L---AL
	Pandinus cavimanus	AEX09191	---NL-LF--ATYE-L-KEVPSY	K	L----VVS-RL--RG-L--RAL
	Callorhinchus milii	AFK10959	---NLILF--ATYD-M-KEVPNY	K	L----VVS-RL-IRG-L--SAL
	Spadella cephaloptera	CAL69114	---NL-LF--ATYD-LYKEVPSY	K	L----VVS-RL-IRG-L---AL
	Homo sapiens	EAW48944	---NL-LF--ATYD-LCKEVPNY	K	L---AVVS-RL-IRG-L---AL
	Mus musculus	EDL25591	---NL-LF--ATYD-LCKEVPNY	K	L---AVVS-RL-IRG-L---AL
	Macaca mulatta	EHH15577	---NI-LF--ATYN-LCKEVPNY	K	L---AVVS-RL-IQG-L---AL
	Myotis davidii	ELK34079	---NL-LF--ATYD-LCKEVPNY	K	L---AVVS-RL-IRG-L---AL
	Columba livia	EMC78310	---NL-LF--ATYD-LCKEVPNY	K	L---AVVS-RL-IRG-L---AL

Figure S19. A partial sequence alignment of the 40S ribosomal protein S25 showing a one amino acid deletion that is specific for members of *Plasmodium.*

			126		167
	Plasmodium berghei str. ANKA	XP_674529	LRGMCNLAHSVDKKKKILVLI		EEDNENLKKYGADYVGLDYIS
	Plasmodium sp. DRC-Itaito	SPJ09601.1	-------V-----N------V		D-EHQ------------E--N
	Plasmodium sp. Gor. Cla. G2	SOV12973.1	-------V-----N------V		D-EHQ------------E--N
	Plasmodium chabaudi ada.	SCM23132	-------T-------------		------------N-------N
	Plasmodium chabaudi cha.	XP_745300	-------T-------------		------------N-------N
	Plasmodium coatneyi	XP_019912435	-------T----RN------V		D--HQA-R-A-------E--N
	Plasmodium cynomolgi str. B	XP_004220735	-------T----RN------V		D--HQT---A-------E--N
	Plasmodium falciparum 3D7	XP_001349022	-------V-----N------V		D-EHQ------------E--N
	Plasmodium falciparum HB3	KOB63185	-------V-----N------V		D-EHQ------------E--N
	Plasmodium falciparum IGH-CR14	KNG76781	-------V-----N------V		D-EHQ------------E--N
	Plasmodium falciparum RAJ116	KNC37048	-------V-----N------V		D-EHQ------------E--N
	Plasmodium falciparum Vie.	ETW19508	-------V-----N------V		D-EHQ------------E--N
	Plasmodium fragile	XP_012334095	-------T----RN------V		D--HIT---A-------E--N
***Plasmodium* (20/20)**	*Plasmodium gaboni*	XP_018642472	-------V-----N------V		D-EHQ------------E--N
	Plasmodium gallinaceum	CRG98075	-------V-----N------V		D-E-K---------------N
	Plasmodium gonderi	GAW78946	-------T----RN------V		DDNHQ----C-------E--N
	Plasmodium inui San Ant. 1	XP_008816772	-------P----RN------V		D--HQT---A-------E--N
	Plasmodium knowlesi str. H	XP_002257587	-------P----RN------V		D--HQS---A-------E--N
	Plasmodium malariae	SBS81409	-------------N------V		D--I-I--NS--H----E--N
	Plasmodium ovale curt.	SBS79893	-------------N------V		D---Q-------H----E--N
	Plasmodium ovale wal.	SBT30433	-------------N------V		D---Q-------H----E--N
	Plasmodium reichenowi	CDO63580	-------V-----N------V		D-EHH------------E--N
	Plasmodium relictum	CRG98439	-------I-----N------V		D-E-K------------E--N
	Plasmodium vinckei pet.	EUD72458	-------T-------------		------------N-------N
	Plasmodium vinckei vin.	XP_008623456	-------T-------------		------------N-------N
	Plasmodium vivax Bra. I	KMZ89496	-----T-T----RN------V		D--HQA---A-------E--N
	Plasmodium vivax Sal-1	XP_001613477	-----T-T----RN------V		D--HQA---A-------E--N
	Plasmodium yoelii 17X	ETB62240	---------------------		--------------------N
	Plasmodium yoelii yoe. 17XNL	XP_726303	---------------------		--------------------N
	Hyphomonadaceae bac. TME.	OUV66055	V--TVS-P-GTG-SVRV---C	N	PDKEQEA-DA---F----EYI
	Candidatus Sulcia muelleri DM.	ADE35363	I--TV--PYGTG-NIRV-A--	P	K-REKEI-NS---FI--NKYI
	Apibacter adventoris	AKP61343	V--VVS-P-GTG-QT-V-A-V	T	P-KEQEA-DA--------EYL
	Alistipes sp. CAG:514	CCX52524	I--VVT-P-GTG-VVRV---C	T	PDKENEA-EA--------EYV
	Alistipes sp. CAG:435	CDD19835	I--VVS-P-GTG-VVRV---C	T	PDKENEA-EA--------EYV
	Prevotella sp. CAG:485	CDE07228	V--VVS-P-GTG-QVRV---C	N	PDMEAAA-EA--------EYI
	Candidatus Sulcia mue. Str.	EAT14117	I--TV--PYGTG-NIRV-A--	P	K-REKEI-NS---FI--NKYI
	Cryomorphaceae bac. BAC.	KRO67929	V--VVT-P-GTG-DV-V-A-V	T	PDKEAEA-EA--------EYL
	Sphingobacteriales bac. B.	KRP05714	I--TTA-P-GTG-E-RV---C	T	P-KE-EA-AA----A---EYV
	Chryseobacterium sp. 39-10	OJV48631	V--VVS-P-GTG-DV-V-A-V	T	P-KEAEA-EA--------EYL
	Sphingobacteriales bac. 5.	OJW55833	I--TVS-P-GTG-T-RV---C	T	PDKEADA-AA--------EFI
	Bacteroidetes bacterium 43-93	OJX01228	I--TVS-P-GTG-T-RV---C	T	PDKENEA-AA---F----EFV
	Sphingobacteriales bac. U.	OQY94594	I--TVT-P-GTG-T--V---C	N	PDKE-ES-NA--------EFV
	Flavobacteriales bac. 34.	OUR90363	V--VVS-P-GTG-DM-V-A-V	T	PDKEAEA-EA--------EYL
	Nonlabens dokdonensis	OUS09520	V--VVT-P-GTG-DV-V-A-V	T	PDKE-EA-NA-------NEYL
**Prokarya**	*Crocinitomicaceae bac. TM.*	OUU17469	V--TVS-P-GTG-NVRV---C	T	PDKEQEA-DA---H----EYV
	Bacteroidetes bacterium ME.	PDH46424	V--VVS-PNGTG-NM-V-A-V	S	PDKE-EA-SA---F----EYI
	Saprospira grandis	WP_002658383	---TIS-PNGTG-E--V--FC	T	PDKEQEA-EA----A--QEYI
	Bacteroides coprosuis	WP_006743888	V--VVS-P-GTG-QVRV---C	T	PDQE-AA-EA--------EYI
	Gillisia limnaea	WP_006987982	V--VVT-P-GTG-DV-V-A-V	T	PDKE-EA-QA--------EYL
	Kordia algicida	WP_007097039	V--VVT-P-GTG-DV-V-A-V	T	PDKEAEA-EA--------EYL
	Xanthomarina gelatinilytica	WP_007649508	V--VVT-P-GTG-DV-V-A-V	T	PDKEAEA-EA--------EYL
	Pontibacter	WP_007654321	V--IVT-P-GTG-DV-V-A-V	T	PDKEQEA-DA---F----DYI
	Flavobacteriales bac. ALC.	WP_008270924	V--VVS-P-GTG-DM-V-A-V	T	PDKEDEA-AA--------EYL
	Mucilaginibacter paludis	WP_008509251	V--IAT-P-GTG-TVRV---C	T	P-KEQEA-DA--------EYI
	Bizionia argentinensis	WP_008638910	V--VVT-P-GTG-DV-V-A-V	T	PDKEAEA-EA--------EYL
	Galbibacter marinus	WP_008991778	V--VVT-P-GTG-DV-V-A-V	T	PDKE-EA-AA--------EYL
	Alistipes indistinctus	WP_009133741	V--VVT-P-GTG-QVRV---C	T	PDKENEA-EA--------EYI
	Sphingobacterium sp. 21	WP_013665678	V--IAT-P-GTG-TVRV---C	T	PDKE-EA-AA--------EYI
	Lacinutrix sp. 5H-3-7-4	WP_013870448	V--VVS-P-GTG-DV-V-A-V	T	PDKEAEA-EA-------QEYL
	Owenweeksia hongkongensis	WP_014201825	V--VVT-P-GTG-DV-V-A-V	T	PDKEQEA-DA----A---EYI

Figure S20. A partial sequence alignment of the 50S ribosomal protein L1 showing a one amino acid deletion that is specific for members of *Plasmodium.* CSI region is only conserved within *Plasmodium* and Prokarya species.

Group	Species	Accession	25		64
***Plasmodium* (19/20)**	*Plasmodium falciparum 3D7*	XP_001347931	KKELSGLRISKALGNS		AKNSKIHGVRKNVARVLTVYNQKR
	Plasmodium falciparum Vie.	ETW15445	----------------		------------------------
	Plasmodium sp. DRC-Itaito	SOV23327.1	------------I---		------------------------
	Plasmodium sp. Gor. Cla. G2	SOV15708.1	------------I---		------------------------
	Plasmodium reichenowi	XP_012763652.1	----------------		------------------------
	Plasmodium cynomolgi str. B	XP_004222472.1	------------I---		------CS----I---------R-
	Plasmodium chabaudi cha.	XP_016655383.1	------------I---		-------S----------------
	Plasmodium berghei ANKA	XP_022712918.1	--------I---		-------I----------------
	Plasmodium fragile	XP_012335766	------------I---		-------S----I---------R-
	Plasmodium gaboni	XP_018638949	------------I---		------------------------
	Plasmodium gallinaceum	CRG96298	--------I---		------RS---------------K
	Plasmodium gonderi	GAW81018	------------I---		------CS----I---------R-
	Plasmodium inui San Ant. 1	XP_008817940	------------I---		T-----CS----I---------R-
	Plasmodium knowlesi str. H	XP_002259252	------------I---		------CS----I-----------
	Plasmodium malariae	SBT71351	-N-------N------		------RS----I--I------R-
	Plasmodium ovale curt.	SBS83668	------------I---		------RS----I---------R-
	Plasmodium ovale wal.	SBT35330	------------I---		------RS----I---------R-
	Plasmodium relictum	CRH00119	--------I---		-------S--------------
	Plasmodium vinckei pet.	EUD72856	------------I---		-------S----------------
	Plasmodium vinckei vin.	XP_008624791	------------I---		-------S----------------
	Plasmodium vivax Bra. I	KMZ86600	------------I---		------CS----I---------R-
	Plasmodium vivax Mau. I	KMZ93052	------------I---		------CS----I---------R-
	Plasmodium vivax Sal-1	XP_001615365	------------I---		------CS----I---------R-
	Plasmodium yoelii 17X	ETB57725	----------------		-------S----------------
	Plasmodium yoelii yoe. 17XNL	XP_723664	----------------		-------S----------------
**Other Apicom-plexa**	*Theileria equi*	XP_004833564	-Q--ATF-V--VTATG	T	S-L---TVI--AI-K-------
	Theileria orientalis str. Shi.	XP_009689759	-Q--ATF-V--VTATG	T	S-L---TL---A--K-------RK
	Babesia microti str. RI	XP_021338440	---YAA--V---T-TG	A	--L---TIIK-SI-K-------RK
	Besnoitia besnoiti	PFH31492	----AQ--VA-VT-GA	A	S-L--VTE---GI-------T--Q
	Eimeria acervulina	XP_013246655	-----H--VA-VT-AV	S	SRLA--TR---GI--I--------
	Eimeria brunetti	CDJ46693	-----R--VA-VT-AT	A	TRLA--IQ---GI---------R-
	Eimeria maxima	XP_013333977	-----Q--VA-VT-AV	A	SRLA--TR---GI-----------
	Eimeria necatrix	XP_013436599	-----L--VA-VT-AT	P	--LA--TQ---GI-----------
	Eimeria tenella	XP_013230664	-----L--VA-VT-AT	P	--LA--TQ---GI-----------
	Hammondia hammondi	XP_008888519	----AQ--VA-VT-SA	A	S-L--VTE---GI-------T--Q
	Neospora caninum Liv.	XP_003886235	----AQ--VA-VT-SA	A	S-L--VTE---GI-------T--Q
	Toxoplasma gondii ME49	XP_002367363	----AQ--VA-VT-SA	A	S-L--VTE---GI-------T--Q
**Other Eukarya**	*Pyronema omphalodes CBS 100.*	CCX30394	-N--TN--VQ-IV-GN	N	T-LT---D---S---T--II-A-Q
	Sporidiobolus salmonicolor	CEQ39519	-T--VS--VQ-IT-GN	A	S-LAR-SV---SI------I---T
	Aspergillus fumigatus A1163	EDP56285	-T---Q--VQ-IAAGA	S	S-TQR--D---SI------I-ANQ
	Gonapodya prolifera JEL478	KXS11835	-Q---S--VQ-VA-GA	T	--L----T-----------IS-TQ
	Peniophora sp. CONT	KZV65790	-N--L---VQ-IA-G-	A	S-LT--N----SI------T---Q
	Cryptococcus depauperatus CBS	ODN90037	-T--GS--VQ-VA-G-	A	S-VT--NS---SI--I---I---Q
	Cryptococcus amylolentus CBS.	ODO10498	-T---S--VQ-IA-G-	A	S-LT--NT---SI------I---Q
	Naematelia encephala	ORY29621	-T--AS--VQ-IA-G-	A	S-LT--NT---SI------I---Q
	Protomyces lactucaedebilis	ORY75415	-Q--AS--VQ-IA-GA	S	S-VT---D----I------IT
	Aspergillus fumigatus	OXN08061	-T---Q--VQ-IAAGA	S	S-TQR--D---SI------I-ANQ
	Cryptococcus gattii WM276	XP_003191713	-T--AS--VQ-IA-G-	A	S-LT--NT---SI--I---I---Q
	Wallemia mellicola CBS 633.	XP_006957816	-G--VQ--VA-VA-G-	A	S-LT---S---SI------I---Q
	Anthracocystis flocculosa PF.	XP_007879129	-T--LQ--VQ-VA-GN	S	S-LT--NT----I------I---Q
	Wallemia ichthyophaga EXF.	XP_009269405	-G--VQ--VA-VA-G-	A	S-LT---S---S-------I---Q
	Cryptococcus neoformans var. g.	XP_012046949	-T--AS--VQ-IA-G-	A	S-LT--NT---SI------I---Q
	Trichosporon asahii var. asa.	XP_014179128	RT--TS--VQ--V-G-	A	S-LT--NT---SI------V---Q
	Rhodotorula graminis WP1	XP_018270768	-T--VS--VQ-VA-GN	A	S-LAR-ST---SI------I---T
	Tsuchiyaea wingfieldii CBS.	XP_019032488	-T---S--VQ-IA-G-	A	S-LT--NT---SI------I---Q
	Kockovaella imperatae	XP_021874786	-T--A---VQ-IA-G-	A	S-LT--NT---SI------I---Q
	Cryptococcus neoformans var. n.	XP_567099	-T--AS--VQ-IA-G-	A	S-LT--NT---SI------I---Q
	Aspergillus fumigatus Af293	XP_752417	-T---Q--VQ-IAAGA	S	S-TQR--D---SI------I-ANQ
	Taphrina deformans PYCC 5710	CCG81552	-Q--AS--VQ-IA-GA	S	S-VT---D----I------I-H
	Pfiesteria piscicida	ABI14370	-G--AT--VA-VS-GA	A	S-LM-NKV----I--I-------Q
	Alexandrium fundyense	ABO47869	-A--AQ--VA-VA-GA	A	S-LA--KI---SI--I-------Q
	Symbiodinium microadriaticum	OLQ11191	-A--AQ--V--VS-GA	A	S-LA--KT---AI--I-------Q
	Nannochloropsis gaditana CCMP.	XP_005855084	-----E--VA-VT-GA	A	S-LA--KV---SI---------TQ
	Scophthalmus maximus	ABJ98654	-N---Q--VA-VT-GA	A	S-L---RV---SI------I--TQ
	Pristionchus maupasi	ABR87393	-G--AS-QV--VT-GA	A	S-L---RV----I------I--TQ

Figure S21. A partial sequence alignment the 60S ribosomal protein L35 showing a one amino acid deletion that is specific for members of *Plasmodium* and some Fungi (not shown).

Group	Species	Accession	436		487
Plasmodium (20/20)	*Plasmodium berghei ANKA*	XP_680201	FSNLNNIIFESEKYIKEMINFVLCKS		EDIEYINEHHDQNLKKNLEEILKKTF
	Plasmodium sp. Gor. Cla. G2	SOV11615.1	----KD--DV--E------TYA-Y--		--VN-L--Y--KD--NK--N--N-K-
	Plasmodium sp. DRC-Itaito	SOV21302.1	----KD--DV--E------TYA-Y--		--VN-L--Y--K---NK--N--N-K-
	Plasmodium chabaudi ada.	SCN60923	--D-----L---N----------H--		-------------I----------P-
	Plasmodium chabaudi cha.	SCM23426	--D-----L---N----------H--		-------------I----------P-
	Plasmodium yoelii 17X	ETB56873	--D--H--L--------------S--		------------------------P-
	Plasmodium yoelii yoe.	EAA22810	--D--H--L--------------S--		------------------------P-
	Plasmodium vinckei pet.	EUD73205	--D-----S---N----------H--		------------------------P-
	Plasmodium vinckei vin.	XP_008624495	--D-----S---N----------H--		-------------I----------P-
	Plasmodium coatneyi	XP_019915279	----AS--SLA-E---A-V--A-H--		--VN-LH-----T--QK-QNV-Q-P-
	Plasmodium cynomolgi str. B	XP_004222917	----ET--SLA-E-----VK-A-H--		---D--S----DK--EK-QNV-Q-P-
	Plasmodium falciparum 3D7	XP_001351654	----KD--DI--E------TYA-Y--		--VN-L--Y--KD--NK--N--N-K-
	Plasmodium falciparum 7G8	EUR77401	----KD--DI--E------TYA-Y--		--VN-L--Y--KD--NK--N--N-K-
	Plasmodium falciparum CAMP/Mal.	ETW63026	----KD--DI--E------TYA-Y--		--VN-L--Y--KD--NK--N--N-K-
	Plasmodium falciparum Dd2	KOB84922	----KD--DI--E------TYA-Y--		--VN-L--Y--KD--NK--N--N-K-
	Plasmodium falciparum FCH/4	ETW28086	----KD--DI--E------TYS-Y--		--VN-L--Y--KD--NK--N--N-K-
	Plasmodium falciparum HB3	KOB59174	----KD--DI--E------TYA-Y--		--VN-L--Y--KD--NK--N--N-K-
	Plasmodium falciparum IGH-CR14	KNG74482	----KD--DI--E------TYA-Y--		--VN-L--Y--KD--NK--N--N-K-
	Plasmodium falciparum MaliPS09	ETW50895	----KD--DI--E------TYA-Y--		--VN-L--Y--KD--NK--N--N-K-
	Plasmodium falciparum NF135/5.	ETW44517	----KD--DI--E------TYA-Y--		--VN-L--Y--KD--NK--N--N-K-
	Plasmodium falciparum NF54	PKC45762	----KD--DI--E------TYA-Y--		--VN-L--Y--KD--NK--N--N-K-
	Plasmodium falciparum Pal. Alt.	ETW53546	----KD--DI--E------TYA-Y--		--VN-L--Y--KD--NK--N--N-K-
	Plasmodium falciparum San. Lu.	EUT90588	----KD--DI--E------TYA-Y--		--VN-L--Y--KD--NK--N--N-K-
	Plasmodium falciparum Tan.	ETW38182	----KD--DI--E------TYA-Y--		--VN-L--Y--KD--NK--N--N-K-
	Plasmodium falciparum UGT5.1	EWC78256	----KD--DI--E------TYA-Y--		--VN-L--Y--KD--NK--N--N-K-
	Plasmodium falciparum Vie.	ETW20090	----KD--DI--E------TYA-Y--		--VN-L--Y--KD--NK--N--N-K-
	Plasmodium fragile	XP_012334611	--D-AS--TLA-E---A-VK-A-HN-		---N-M--Q--EK--QK-QNV-E-P-
	Plasmodium gaboni	XP_018643033	----KD--DV--E------TYA-Y--		--VN-L--Y--KD--NK--N--N-K-
	Plasmodium gallinaceum	CRG97872	-----D--SL--D------K-A-Y--		-E---LY-N--K----K--NV-NQK-
	Plasmodium gonderi	GAW81459	----EK--CI--E------K-A-Y--		--VD---NY-NK----K--NT-NAQ-
	Plasmodium inui San Ant. 1	XP_008815068	----AS--SLA-E---A-VK-A-YS-		---D--------R--QK-RQV-E-P-
	Plasmodium knowlesi str. H	XP_002259720	-C--SS--SIA-E---A--K-A-Y--		---D--------G--HK-QNV-Q-P-
	Plasmodium malariae	SBS92251	--------SL--E------K-A-Y--		D----L--N--K-I--K--D--N-K-
	Plasmodium ovale curt.	SBS84580	----DD--SL--E----I-S-A-Y--		-----L--SC-K-I--K--NV---K-
	Plasmodium ovale wal.	SBT36498	----DD--SL--E----I-S-A-Y--		-----L--NC-K-I--K--IV-N-K-
	Plasmodium reichenowi	XP_012761613	----KD--DI--E------TYA-Y--		--VN-L--Y--K---NK--N--N-K-
	Plasmodium relictum	CRH00573	-----D-VSV--D------K-A-Y--		-E---L--N--K----K--NV-NQK-
	Plasmodium vivax Bra. I	KMZ86361	----TS--SLA-E---T-VK-A-HQ-		--VD--H----RT--EK-QAV-Q-P-
	Plasmodium vivax Ind. VII	KMZ79989	----TS--SLA-E---T-VK-A-HQ-		--VD--H----RT--EK-QAV-Q-P-
	Plasmodium vivax Mau. I	KMZ92722	----TS--SLA-E---T-VK-A-HQ-		--VD--H----RT--EK-QAV-Q-P-
	Plasmodium vivax North Kor.	KMZ99047	----TS--SLA-E---T-VK-A-HQ-		--VD--H----RT--EK-QAV-Q-P-
	Plasmodium vivax Sal-1	XP_001613268	----TS--SLA-E---T-VK-A-HQ-		--VD--H----RT--EK-QAV-Q-P-
Firmicutes	*Clostridium tetani*	WP_011098467	-AD-TDYMDVA-EMV-YI--Y--ENA	P	-EMNFF-SFI-KD-F-R-DNVVNSE-
	Garciella nitratireducens	WP_087677725	-AD-KDNMMLA-EMM-YI-KY--ENA	A	-EM-FF-KFV-RE--NR--N-MNSK-
	Desulfitobacterium dehalogenan	WP_014795896	-AD-QDDMELA-EMM-YL-TY--EHA	P	-EMAFF--FV-KT-FSR--N-VNSD-
	Clostridiales bacterium KA0.	WP_066535861	-ADI-D-MDS--EML-FV-SY--E-C	P	DEMNFF-SFI-KG-LDR-DN-V-SD-
	Bacillus sp. OV166	WP_088088555	-AE-PD-MDLA-EMV-SV-GY--EQA	P	-EMNFF-SFIEKG-LDR-NNAYTAD-
	Clostridium sp. K25	WP_003375192	-AE-KDYMDVA-EMV-YI--Y-RENA	P	-EM-FF-KFI-KG-LER-DNVVNSD-
Bacteroidetes /Chlorobi group	*Bacteroidetes bacterium HGW.*	PKP19585	--D--DNMDLA-EFLQYL-RYA-ENC	S	D-LKFL-DMF-KE-ISR--FVINNR-
	Tannerella sp. oral taxon BU.	ETK02966	-YEIEDNMDLA-EF--YCVRWA-DHC	A	D-LRFL-DMF-KE-IVR--G--QAP-
	Bacteroidetes bacterium RIFOX.	OFZ07521	-YDIKDNMDLA-EF--YLVKYA-ENC	M	D-LQFL-DMF-KE-IER-RSVVNTE-
	Bacteroidetes bacterium GWE2.	OFX78079	-YDIKDNMDLA-EF--YLVKYA-ENC	M	D-LQFL-DMF-KE-IER-RSVVNTE-
	Hymenobacter psychrophilus	WP_092739915	-ND-QDNMDLA-DFL-SLVRYA-AHC	E	D-LKFL--QY-KE-LTR-QFVVDND-
	Hymenobacter actinosclerus	WP_092774111	-ND-QDNMDLA-DFL-SLVRYA-AHC	E	D-LKFL--QY-KE-LTR-QFVVDND-
	Bacteroides xylanisolvens XB1.	CBK69038	-NDI-DNMDLA-DFL-YL-RYA-ENC	E	S-LAFL-DNV-NG-MDK-HFVANNE-
	Bacteroides thetaiotaomicron C.	CDE80939	-NDI-DNMDLA-DFL-YL-RYA-ENC	E	S-LAFL-DNV-NG-MDK-HFVANNE-
	Paraprevotella clara CAG:116	CCZ02085	-NDI-DNMDLA-DFL-YL-RYA-ENC	E	S-LAFL-DNV-NG-MDK-HFVANNE-

Figure S22. A partial sequence alignment of the Asparagine-tRNA ligase protein showing a one amino acid deletion that is specific for members of *Plasmodium.* CSI region is only conserved in *Plasmodium* and various bacteria.

Group	Species	Accession	385		438
**_Plasmodium_ (20/20)**	*Plasmodium berghei ANKA*	CDS45652	LYGNSDTSFRFNQINNFTKHED	I	QFLLVTDLASRGIHIPSVQNVINYNLPFSPK
	Plasmodium sp. gorilla clade G2	SOV13816.1	-----------E------NNNH	-	-------V-----N-T---------------
	Plasmodium sp. DRC-Itaito	SPJ09988.1	-----------E-------NDH	-	-------V-----N-T---------------
	Plasmodium chabaudi ada.	SCN60705	--------------------D-	-	-------------N-T---------------
	Plasmodium chabaudi cha.	CAH75349	--------------------D-	-	-------------N-T---------------
	Plasmodium coatneyi	XP_019913407	-----------E--RM--NS-E	-	-------V-A---N-T--------S------
	Plasmodium falciparum 3D7	XP_001349256	-----------E-------N-H	-	-------V-----N-T---------------
	Plasmodium falciparum 7G8	EUR46871	-----------E-------N-H	-	-------V-----N-T---------------
	Plasmodium falciparum CAMP/Mal.	ETW62076	-----------E-------N-H	-	-------V-----N-T---------------
	Plasmodium falciparum Dd2	KOB84732	-----------E-------N-H	-	-------V-----N-T---------------
	Plasmodium falciparum FCH/4	ETW30994	-----------E-------N-H	-	-------V-----N-T---------------
	Plasmodium falciparum HB3	KOB60990	-----------E-------N-H	-	-------V-----N-T---------------
	Plasmodium falciparum IGH-CR14	KNG76216	-----------E-------N-H	-	-------V-----N-T---------------
	Plasmodium falciparum Mali.	ETW49694	-----------E-------N-H	-	-------V-----N-T---------------
	Plasmodium falciparum NF135/5.	ETW43421	-----------E-------N-H	-	-------V-----N-T---------------
	Plasmodium falciparum NF54	EWC88823	-----------E-------N-H	-	-------V-----N-T---------------
	Plasmodium falciparum Pal. Alt.	ETW52389	-----------E-------N-H	-	-------V-----N-T---------------
	Plasmodium falciparum RAJ116	KNC35823	-----------E-------N-H	-	-------V-----N-T---------------
	Plasmodium falciparum San. Lu.	EUT87343	-----------E-------N-H	-	-------V-----N-T---------------
	Plasmodium falciparum Tan.	ETW36986	-----------E-------N-H	-	-------V-----N-T---------------
	Plasmodium falciparum UGT5.1	EWC76992	-----------E-------N-H	-	-------V-----N-T---------------
	Plasmodium falciparum Vie.	ETW18826	-----------E-------N-H	-	-------V-----N-T---------------
	Plasmodium fragile	XP_012333808	-----------D--KK--NM-E	-	-------V-A---N-T---------------
	Plasmodium gaboni	XP_018642358	-----------E------NNNH	-	-------V-----N-T---------------
	Plasmodium gallinaceum	CRG93495	----A------Q------ND-K	-	-------------N-T-------------S-
	Plasmodium gonderi	GAW79636	----------LE--KK--QN-N	-	-------V-A---N-T--------S------
	Plasmodium inui San Ant. 1	XP_008816501	-----------D--KK--NS-E	-	-------V-A---N-T---------------
	Plasmodium knowlesi str. H	XP_002258052	--------Y-LD--KK--NN-E	-	--M----V-A---N-T--------S------
	Plasmodium malariae	SBT87328	----------IQ------RNNN	-	-------------N-TT---I------Y---
	Plasmodium ovale curt.	SBS80989	-----------E---T-S-N-H	V	-------------N-T----I----------
	Plasmodium ovale wal.	SBT32023	-----------E---T-I-N-H	V	-------V-----N-T---------------
	Plasmodium reichenowi	XP_012762655	-----------E------NNDN	-	-------V-----N-T---------------
	Plasmodium relictum	CRH03734	----A------Q------NDQK	-	-------------N-A--K----------S-
	Plasmodium vinckei pet.	EUD70693	----------------------	-	-------------N-T---------------
	Plasmodium vinckei vin.	XP_008626234	--------------------D-	-	-------------N-T---------------
	Plasmodium vivax Bra. I	KMZ88036	-----------D--KK--NTQE	-	-------V-A---N-T--------S------
	Plasmodium vivax Ind. VII	KMZ82012	-----------D--KK--NTQE	-	-------V-A---N-T--------S------
	Plasmodium vivax Mau. I	KMZ94414	-----------D--KK--NTQE	-	-------V-A---N-T--------S------
	Plasmodium vivax North Kor.	KNA01258	-----------D--KR--NTQE	-	-------V-A---N-T--------S------
	Plasmodium vivax Sal-1	XP_001614894	-----------D--KK--NTQE	-	-------V-A---N-T--------S------
	Plasmodium yoelii 17X	ETB58610	-------------------N--	-	-------------N-T---------------
	Plasmodium yoelii yoe. 17XNL	XP_723756	-------------------N--	-	-------------N-T---------------
**_Piroplasmida_ and other Apicom-plexa (0/8)**	*Theileria parva str. Mug.*	XP_763213.1	V--TM-MDN-VIEMSR-RSNKT		RI-----V----LD--LVDL---FDF-Y---
	Theileria orientalis	PVC53682.1	-H--LSQ-K--ESVEK-K-S-V		DY--ASE-----LD--GIKT---VD--
	Theileria equi str. WA	XP_004832001.1	V--SM-M-M-TS-MSQ-QSFKT		TI-I-----A--LDL-L-DI-V-FDF-H-S-
	Theileria annulata	XP_953408.1	-H--LSQ-K--ESVER-KNG-I		DY--ASE-----LD--G-KT---VD--
	Babesia bovis T2Bo	XP_001611999.1	-H-DLAQAK--E--EK-KNG-V		D--MASE-----LD--GISA---VH---
	Babesia bigemina	XP_012767797.1	V--AM-MTA-SQ-MSL-RSCKT		KV-I-----A--LDL-M-DC-V-FDF-H-S-
	Babesia ovata	GBE61222.1	V--AM-MTA-SQ-MSL-RSCKT		KV-I-----A--LDL-M-DC---FDF-H-S-
	Babesia microti str. RI	XP_021338374	V--SM-M-L-MQ-MA--SNSKT		N--I---V-A--LD--I-N----FDF-Y---
	Cryptosporidium andersoni	OII76590	I---M-QDA-SA-L-S-RRNKT		RVMI---I-A--LD--LLE----FDY-L-A-
	Cryptosporidium muris RN66	XP_002141594	I---M-QDA-NA-L-S-RRNKT		RVMI---I-A--LD--LLE----FDY-L-A-
	Cryptosporidium parvum Iowa II	XP_001388110	I---M-QEA-TMHL-T-R-NKS		RA-I---I-A--VD--MIKY---FDF-L---
	Cryptosporidium hominis TU502	XP_666233	I---M-QEA-TMHLST-R-NKS		RA-V---I-A--VD--MIKY---FDF-L---
	Cryptosporidium ubiquitum	OII74201	I---M-QEA-TMHLST-R-NKS		RA-I---I-A--VD--MIKY---FDF-L---
**Other Eukarya**	*Entamoeba dispar SAW760*	XP_001736127	MF-KA-QQE-EINLKK-R-Q-T		HV-----V-A--VD--ELD-----DF-AT--
	Blastocystis sp. subtype 4	XP_014524883	I--SMELTN-MHNLSL-Q-NQL		PI-V---V-A--LD--I-NT--HFDC-S---
	Vitrella brassicaformis CCMP.	CEM11820	V--TM-QTA-EQ-LAH-R-GVT		RV-V---V-A--LD--FLEY-V-FDF-T---
	Tetrahymena thermophila SB2.	XP_001013358	I--KM-AFA-KD--SE-RNKKC		NVMV---------DL-N-N---H-DY-A-T-
	Paramecium tetraurelia str.	XP_001462504	V--KM-QLD-KE-LD--KRNQV		KV-I---------DL-F-A---H-DY-SN--
	Ichthyophthirius multifiliis	XP_004036915	I--KM-PLA-KD---E-KSKRV		NV-V-----A---DL-H-----HFD--AQT-
	Rhynchosporium secalis	CZT45482	A--SL-QTA-NQ-VED-RTGRS		NI-V---V-A---D--VLA-----DF-PQ--
	Pichia membranifaciens	GAV30019	I--SL-QHA-RQ-LQ--RMGLS		SVMV---V-A---D--VLA-----S--S-S-
	Ustilaginoidea virens	KDB16953	V--SL-QTA-R--VD--R-GRT		NIIV---V-A---D--ILA-----DF-SQ--
	Stachybotrys chlorohalonata IB.	KFA61912	V--SL-QTA-RM-VED-RRGKT		NI-V---V-A---D--ILA-----DF-SQ--

Figure S23. A partial sequence alignment the ATP-dependent RNA helicase DBP10 protein showing a one amino acid insertion that is specific for members of *Plasmodium.*

Group	Species	Accession	296		338
***Plasmodium* (20/20)**	*Plasmodium falciparum 3D7*	XP_001349840	WRIKTGICLKIIN		AHNDSLISIQFNNDQTQILTSSYDKSVKIF
	Plasmodium falciparum 7G8	EUR66802	-------------		------------------------------
	Plasmodium falciparum Dd2	KOB85949	-------------		------------------------------
	Plasmodium falciparum FCH/4	ETW29683	-------------		------------------------------
	Plasmodium falciparum HB3	KOB60606	-------------		------------------------------
	Plasmodium falciparum Tan.	ETW34981	-------------		------------------------------
	Plasmodium falciparum UGT5.1	EWC74911	-------------		------------------------------
	Plasmodium falciparum Vie.	ETW17122	-------------		------------------------------
	Plasmodium sp. DRC-Itaito	SOV24165.1	-------------		----T-T--------------------V-
	Plasmodium sp. Gor. Cla. G2	SOV17492.1	-------------		----T-T--------------------V-
	Plasmodium gaboni	XP_018639918	-------------		----T-T--------------------V-
	Plasmodium reichenowi	XP_012764492	-G--R--------		------------------------------
	Plasmodium berghei ANKA	XP_680332	----S-S--RQ--		--TNAIT-M---T-------C---NT---H
	Plasmodium chabaudi cha.	SCM25942	------S--RQ--		--TNAIT-MH--A-------C---NT---H
	Plasmodium coatneyi	XP_019916682	-K----S--RT--		--TNAVT-VH--------------L-----
	Plasmodium cynomolgi str. B	XP_004224726	-K----S--RS--		--TNAVT-VH--------------L-----
	Plasmodium fragile	XP_012333281	-K----S--RT--		--TNAVT-VH--------------L-----
	Plasmodium gallinaceum	CRG97501	----N-V---T-S		--VHPIT--N--K-----------NT----
	Plasmodium gonderi	GAW83320	------S--RT--		--TNAIT--H--------------I-----
	Plasmodium inui San Ant. 1	XP_008814325	-K----S--RS--		--TNAVT-VH--------------L-----
	Plasmodium knowlesi str. H	XP_002262005	-K----S--RS--		--TNAVT--H--------------L-----
	Plasmodium malariae	SBS90176	--S--RT--		--TNAIA--S--K-----------NT---Y
	Plasmodium ovale curt.	SBS97972	------S--RQ--		--TNAIT--H--K----L------S----H
	Plasmodium ovale wal.	SBT47208	------S--RQ--		--TNAIT--H--K----L------S----H
	Plasmodium relictum	CRH02361	----S-S---T--		--THPVT--N--K-----------NT----
	Plasmodium vinckei pet.	EUD74173	------S--RQ--		--TNAIT-MH--T----V--C---NT---H
	Plasmodium vinckei vin.	XP_008623133	------S--RQ--		--TNAIT-MH--T----V--C---NT---H
	Plasmodium vivax North Kor.	KMZ97089	-K----S--RT--		--TNAVT-VH--------------L-----
	Plasmodium vivax Sal-1	XP_001617041	-K----S--RT--		--TNAVT-VH--------------L-----
	Plasmodium yoelii	XP_730731	------S--RQ--		--TNAIT-M---T-------C---NT---H
***Piroplasmida* & Other Apicomplexa (0/6)**	*Babesia ovata*	GBE59687	-K-A--E-VRKM-	N	--DGAVTCMT-SR-S-NL-AG-F--TA-VH
	Babesia bovis T2Bo	XP_001610463	-K-A--E-MRKMD	N	--DGAVTCMT-SRNSMSL--G-F--TA--H
	Babesia bigemina	XP_012767979	-K-A--E-VRKM-	N	--DGAVTCMT-SR-S-SL--G-F--TARVH
	Theileria orientalis str. Shi.	XP_009689023	-K-G--E---SM-	N	S--GAVTCAT-SR-SSNL--G-F-SLAR-H
	Theileria parva str. Mug.	XP_765729.1	-K-D--E---TM-	N	S-KGAVTCAT-SR-SSCL--G-F-SLAR-
	Theileria annulata	XP_954516.1	-K-D--E---TM-	N	S-KGAVTCAT-SR-SSCL--G-F-SLARFY
	Eimeria maxima	XP_013334335	-LVA--Q-I-K-D	K	--DAAIA--T-SK-S-H---G-F-TTAR-H
**Other Eukarya**	*Plasmodiophora brassicae*	CEO98519	--VR--A-V-RFP	A	--VKGVT-L--SR-S--V--G---G--RVH
	Ostreococcus lucimarinus CCE.	XP_001417659	--VS--T--RKFE	K	--QGGVT-VT-SK-GS-V-SG-F-GL-RVH
	Coccomyxa subellipsoidea C-16.	XP_005646689	-K----Q--RRFE	S	--SQGVT-VA-SR-GSHV-SA---GLARVH
	Chlorella variabilis	XP_005846379	--VR--Q--RRFD	S	--SQGVT-LA-SR-G-HV-SA---TL-RVH
	Salpingoeca rosetta	XP_004992494	-KVS--Q--RKYE	R	--AEGITCVA-SR-NS--AS--F-HT---H
	Vitrella brassicaformis CCMP.	CEM13105	-K-S--K---KYT	K	I-QGGIT--V-SK-SS-L--A-F-NTARLH
	Mucor ambiguus	GAN07662	-KV-S-Q-Q-R-P	A	--TEGVT--S-SK-S--V--A---QTIR-H
	Mucor circinelloides f. lus.	OAD02260	-KV-S-Q-Q-R-P	A	--TEGVT--S-SK-S--V--A---QTIR-H
	Bifiguratus adelaidae	OZJ06452	-K-QS-L-TRR-S	P	--SQGVT-VC--KEG--V-SG-F-ST--LH
	Mucor circinelloides f. cir.	EPB90502	-KV-S-Q-Q-R-P	A	--TEGVT--S-SK-S--V--G---HTIR-H
	Piromyces finnis	ORX44849	-K-Q--Q-IRRFS	N	--SQGVT-VC--R-N--V-S--F-F-IR-H
	Batrachochytrium dendrobatidis	XP_006675044	-KVH--Q-I-RFP	L	--SQGIT-LY----S--L-SA-F-GV---H
	Parasitella parasitica	CEP10218	-KV-S-Q-Q-R-P	A	--TEGVT--S-SK-S--V-SG---QT-R-H
	Neocallimastix californiae	ORY57616	-K-Q--Q-IRRFS	N	--SQGVT-VC--R-N--V-SC-F-F-IR-H
	Spizellomyces punctatus DAOM B.	XP_016610349	--VQ--Q-IRRFA	T	--TQGVT-VCLSK-G--V-S--F-QTIR-H
	Absidia glauca	SAM07248	-KVSN-T--RR-S	P	--SEGVA-LC--K-G----SG---QTIRLH
	Anaeromyces robustus	ORX85778	-K-Q--Q-IRRFS	N	--SQGVT-VC--R-N--V-SC-F-F-IR-H
	Choanephora cucurbitarum	OBZ90185	-KV-S-Q-Q-R-P	A	--TEGVTCLC-SK-S----SG---HT-RVH
	Rhizophagus irregularis DAOM 1.	EXX56789	-K-Q--Q-TRRFS	P	--SQGVT-VC--R-G----S--F-HT-RLH
	Phycomyces blakesleeanus NR.	XP_018296538	-KVQ---SQRRLS	P	--SQGVT-VC--K-G--V-SG---HT---H
	Rhizopus microsporus ATCC 52.	PHZ13526	--V---Q-E-R-A	T	G-SEAITTLC--K-N--L-SG-Q-HV-R-H
	Cimex lectularius	XP_014245302	-KLM--Q--RKFE	K	---KGITC---SR-NS----A-F-TTIR-H
	Daphnia pulex	EFX80413	-K-Q--Q--RKFE	K	--SKGVT-M--SK-NS-L--G-F-M--RVH
	Macrobrachium rosenbergii	AHJ61045	--VN--Q--RKVE	R	--SEGVT-LA--K-NS-VMST-F-NTIR-H
	Helobdella robusta	XP_009020693	-KVL--Q--RKLD	R	--SKGIT--Y-SR-NS-L--A-F-MVI---
	Trichuris suis	KHJ45516	-K-MS-Q-IRRFA	R	--TKCVT-M---K-N----S----MK-R-H

Figure S24. A partial sequence alignment of a Putative alternative splicing regulator protein showing a one amino acid deletion that is specific for members of *Plasmodium* with some exceptions in *Streptophyta* (not shown).

Group	Species	Accession	387		437
***Plasmodium* (20/20)**	*Plasmodium berghei ANKA*	XP_677822	ISTQVGARGLDLQNIKIVINFDLCSC		KEYIHRVGRTARAGKTGKSITFVTQ
	Plasmodium sp. DRC-Itaito	SOV20743	-------------D--------I---		--------------R----------
	Plasmodium sp. Gor. Cla. G2	SOV10636	-----------I-D--------I---		--------------R----------
	Plasmodium chabaudi ada.	SCM00839	--------------------------		-------------------------
	Plasmodium chabaudi cha.	XP_742705	--------------------------		-------------------------
	Plasmodium coatneyi	XP_019913265	------------KD------------		--------------R----------
	Plasmodium cynomolgi str. B	XP_004225318	------------KD------------		--------------R----------
	Plasmodium falciparum 3D7	XP_001349688	-------------D--------I---		--------------RS---------
	Plasmodium falciparum CAMP/Ma.	ETW63775	-------------D--------I---		--------------RS---------
	Plasmodium falciparum Mal.	ETW51611	-------------D--------I---		--------------RS---------
	Plasmodium falciparum NF54	EWC90863	-------------D--------I---		--------------RS---------
	Plasmodium fragile	XP_012336055	------------KD------------		--------------R----------
	Plasmodium gaboni	XP_018643839	-------------D--------I---		--------------R----------
	Plasmodium gallinaceum	CRG94387	--------------------------		-------------------------
	Plasmodium gonderi	GAW79426	------------KD--------I---		--------------R----------
	Plasmodium inui San Ant. 1	XP_008816535	------------KD------------		--------------R----------
	Plasmodium knowlesi str. H	XP_002257851	------------KD------------		--------------R----------
	Plasmodium malariae	SBT86445	------------KD----------T-		--------------R----------
	Plasmodium ovale curt.	SBS80541	-------------D------------		--------------R------L---
	Plasmodium ovale wal.	SBT31481	-------------D------------		--------------R------L---
	Plasmodium reichenowi	CDO62411	-------------D--------I---		--------------RS---------
	Plasmodium relictum	CRG98666	------------KD------------		-------------------------
	Plasmodium vinckei pet.	EUD69924	--------------------------		-------------------------
	Plasmodium vinckei vin.	XP_008626591	--------------------------		-------------------------
	Plasmodium vivax Bra. I	KMZ88339	------------KD-R----------		--------------R----------
	Plasmodium vivax Ind. VII	KMZ82214	------------KD-R----------		--------------R----------
	Plasmodium vivax Mau. I	KMZ94704	------------KD-R----------		--------------R----------
	Plasmodium vivax North Kor.	KNA01352	------------KD-R----------		--------------R----------
	Plasmodium vivax Sal-1	XP_001612863	------------KD-R----------		--------------R----------
	Plasmodium yoelii 17X	ETB57231	------------K-------------		-------------------------
	Plasmodium yoelii yoe.	EAA20538	------------K-------------		-------------------------
***Piroplasmida* & Other Apicomplexa (0/8)**	*Theileria annulata*	XP_951988	VV-D--S--I-IPFVDL-----VPNT	S	-D-------------S-I-L-LI--
	Theileria equi	XP_004829383	VV-D--S----IPSVDL-----IPQS	S	-D------------R--TA--V---
	Theileria orientalis str. Shi.	XP_009690771	VV-D--S----IPLVDL-----VPQT	S	-D-------------S-L-V--I--
	Theileria parva str. Mug.	XP_765295	VV-D--S----IPFVDL-----VPNT	S	-D-------------S-I-L-LI--
	Babesia bovis T2Bo	XP_001609069	VA-E--G-----PMVEL-----IPE-	S	-D------------RS-LAL-V---
	Babesia bigemina	XP_012769901	VA-E--G-----PMVQL-L---IPES	S	-D------------RS-LAL-----
	Babesia ovata	GBE61469	VA-D-AS----VRDV---V---FPNQ	I	ED-V--I---G-G-NK-A-Y--L-
	Babesia microti str. RI	XP_021338401	VT-E--S----IPSVDL-V---VPQS	G	-D------------RS-QA--L---
	Besnoitia besnoiti	PFH33363	VA-E--S----IPHVQM-V---VPLS	S	--------------RS-RAL-I---
	Cryptosporidium andersoni	OII77591	FT-E--S----IPHVDF-----IPMS	S	-D-V----------RS--A-SLI--
	Cryptosporidium hominis TU502	XP_668635	FT-E--S----IPHVDF-----IPTS	S	-D-V----------RS-RA-SM---
	Cryptosporidium muris RN66	XP_002140422	FT-E--S----IPHVDF-----IPMS	S	-D-V----------RS--A-SL---
	Cryptosporidium parvum Iowa II	XP_625901	FT-E--S----IPHVDF-----IPTS	S	-D-V----------RS-RA-SM---
	Cryptosporidium ubiquitum	OII72145	FT-E--S----IPHVDF-----VPTS	S	-D-V----------RS-RAVSI---
**Other Eukarya**	*Monoraphidium neglectum*	XP_013891892	VA-D-A-----IPSVDV---Y-VPAN	S	-D-V----------RS-R-V-I---
	Stylonychia lemnae	CDW75613	-A-D-AS----IPEVD------IPQH	S	-D-V-------------RA--I---
	Oxytricha trifallax	EJY73340	-A-D-AS----IPEVDF-----IPQH	S	-D-V--------------A------
	Perkinsus marinus ATCC 50983	XP_002781775	VA-D-A-----IPSVDV-----VPKN	P	E-------------R--R-V-L---
	Candida parapsilosis	CCE45114	VA-D-A-----IPSVDV---Y-IPTD	S	-A------------RS----SLI--
	Millerozyma farinosa CBS 7064	CCE84713	VA-D-A-----IPSVDV---Y-IPTD	S	-A-------------S----SL---
	Taphrina deformans PYCC 5710	CCG84237	-A-D-AS----IPLVDV-----IPTD	S	-S------------RS---LSL---
	Cyberlindnera fabianii	CDR39618	VA-D-A-----IPSVDV-V-Y-IPTD	S	-A------------RS----SL---
	Lichtheimia ramosa	CDS04815	VA-D-AS----IPMVD--V-Y-VPLS	S	-D------------RS---M-----
	Rhizopus microsporus	CEG84656	VA-D-AS----IPTVD----Y-VPQS	S	-D------------RS---------
	Candida albicans WO-1	EEQ43982	VA-D-A-----IPSVDV---Y-IPTD	S	-A------------RS----SLI--
	Brettanomyces bruxellensis AW.	EIF46047	VA-D-A-----IPSVDLI--Y-IPTD	S	-A------------R-----SL---
	Candida maltosa Xu316	EMG49265	-A-D-A-----IPSVD----Y-IPTD	S	-A------------RS----SLI--
	Mortierella verticillata NR.	KFH71721	-A-D-AS----IPSVD--L-Y--PNN	S	-D------------RS---------
	Candida albicans P94015	KGQ80445	VA-D-A-----IPSVDV---Y-IPTD	S	-A------------RS----SLI--
	Hydnomerulius pinastri MD-312	KIJ69220	VA-D-AS-----PSVDV-----IPTH	S	-D------------RA-----M---
	Choanephora cucurbitarum	OBZ90399	VA-D-AS--I-IPSVD----Y-VPQS	S	-D------------RS---------
	Pachysolen tannophilus NRRL Y.	ODV94751	VA-D-A-----IPDVD----Y-IPTD	S	-A-------------S----SL---
	Piromyces finnis	ORX52142	-A-D-AS----IPSVDV---Y-VPQS	S	-D------------RS---V-----

Figure S25. A partial sequence alignment of the Adenosinetriphosphatase protein showing a one amino acid deletion that is specific for members of *Plasmodium*.

			950		10133
***Plasmodium* (20/20)**	Plasmodium berghei ANKA	CDS49097	VGDPKQLSATVFSLFAKKHNYSRSLFERLQKI	HKFN	KCKYNLLSIQYRMHPDISHFPNRYYYNN
	Plasmodium sp. DRC-Itaito	SOV22826	-------------------K-A---------K	YLM-	ER-------------E--A--KKC--K-
	Plasmodium sp. gorilla clade G2	SOV14709	-------------------K-A---------K	YLT-	ER-------------E--A--KKC--K-
	Plasmodium chabaudi cha.	XP_743403	--------------------------------	Y---	-S--------------------KH--K-
	Plasmodium coatneyi	XP_019914493	-----------------RRK-A---------K	--M-	-S--------------------K---R-
	Plasmodium cynomolgi str. B	XP_004221986	-----------------RRK-A---------K	--M-	-Y--------------------K---R-
	Plasmodium falciparum 3D7	XP_001347384	--------------Y----K-A---------K	YLT-	ER-------------E--A--KKC--K-
	Plasmodium falciparum 7G8	EUR71833	--------------Y----K-A---------K	YLT-	ER-------------E--A--KKC--K-
	Plasmodium falciparum Dd2	KOB86968	--------------Y----K-A---------K	YLT-	ER-------------E--A--KKC--K-
	Plasmodium falciparum FCH/4	ETW30780	--------------Y----K-A---------K	YLT-	ER-------------E--A--KKC--K-
	Plasmodium falciparum HB3	KOB60886	--------------Y----K-A---------K	YLT-	ER-------------E--A--KKC--K-
	Plasmodium falciparum IGH-CR14	KNG77786	--------------Y----K-A---------K	YLT-	ER-------------E--A--KKC--K-
	Plasmodium falciparum Mal.	ETW49084	--------------Y----K-A---------K	YLT-	ER-------------E--A--KKC--K-
	Plasmodium falciparum NF135/5.	ETW42666	--------------Y----K-A---------K	YLT-	ER-------------E--A--KKC--K-
	Plasmodium falciparum NF54	EWC88479	--------------Y----K-A---------K	YLT-	ER-------------E--A--KKC--K-
	Plasmodium falciparum Pal. Alt.	ETW56517	--------------Y----K-A---------K	YLT-	ER-------------E--A--KKC--K-
	Plasmodium falciparum San. Lu.	EUT85738	--------------Y----K-A---------K	YLT-	ER-------------E--A--KKC--K-
	Plasmodium falciparum Tan.	ETW36438	--------------Y----K-A---------K	YLT-	ER-------------E--A--KKC--K-
	Plasmodium falciparum UGT5.1	EWC76454	--------------Y----K-A---------K	YLT-	ER-------------E--A--KKC--K-
	Plasmodium falciparum Vie.	ETW18266	--------------Y----K-A---------K	YLT-	ER-------------E--A--KKC--K-
	Plasmodium fragile	XP_012337375	-----------------RRK-A---------K	-RM-	-Y--------------------K---R-
	Plasmodium gaboni	XP_018641485	-------------------K-A---------K	YLM-	ER-------------E--A--KKC--K-
	Plasmodium gallinaceum	CRG94161	------------------YK-----------K	Y-L-	-S-------------E--Q--KK---K-
	Plasmodium gonderi	GAW80525	----------A-------RK-A---------K	--L-	-Y--------------------K---K-
	Plasmodium inui San Ant. 1	XP_008815992	-----------------RRK-A---------K	--M-	-S--------------------K---R-
	Plasmodium knowlesi str. H	XP_002258740	-----------------RRM-A---------K	--M-	-W----------------Y---K---R-
	Plasmodium malariae	SBS84371	----N--------ML---GK-G---------K	N-M-	-V----------------Y---KF--M-
	Plasmodium ovale curt.	SBS82749	------------------KK-----------K	Y-R-	-S-F------------------K---K-
	Plasmodium ovale wal.	SBT34068	------------------KK-----------K	Y-R-	-S-F------------------KH--K-
	Plasmodium reichenowi	XP_012763139	--------------Y----K-A---------K	YLT-	ER---M---------E--E--KKC--K-
	Plasmodium relictum	CRG99655	------------------YK-----------K	Y-L-	-S-------------E-----KK---K-
	Plasmodium vinckei pet.	EUD72591	--------------------------------	Y---	----------------------KH----
	Plasmodium vinckei vin.	XP_008623328	--------------------------------	Y---	----------------------KH----
	Plasmodium vivax Bra. I	KMZ86965	-----------------RRK-A--------RK	--M-	----------------------K---R-
	Plasmodium vivax Ind. VII	KMZ80832	-----------------RRK-A--------RK	--M-	----------------------K---R-
	Plasmodium vivax Mau. I	KMZ93398	-----------------RRK-A--------RK	--M-	----------------------K---R-
	Plasmodium vivax North Kor.	KNA00064	-----------------RRK-A--------RK	--M-	----------------------K---R-
	Plasmodium vivax Sal-1	XP_001614413	-----------------RRK-A--------RK	--M-	----------------------K---R-
	Plasmodium yoelii 17X	ETB58160	--------------------------------	----	-Y---------------------H----
***Piroplasmida* (0/8)**	Theileria annulata	84997263	----C------C-KV-IQL--DQ---K---LC		GYPV-F-KL----D-L-TR--SM-F-Q-
	Theileria equi	510905472	----C------C-KA-IQL--EQ---K---LC		GYPV-F-KL----D-Q-TR--SM-F-K-
	Theileria orientalis str. Shi.	697893781	----C------C-KV-IQL--DQ---K---LC		GYPV-F-KL----D-L-TR--SM-F-Q-
	Theileria parva str. Mug.	71029418	----C------C-KV-IQL--DQ---K---LC		GYPV-F-KL----D-L-TR--SM-F-Q-
	Babesia bigemina	833489522	----C------C-NV-VSLK-D----Q---MC		GYPV---D-----D-G--R--SM-F-KS
	Babesia bovis T2Bo	156089077	----C------C-NV-VSLK-D----Q---MC		GYPV---D-----D-L--R--SM-F-R-
	Babesia ovata	GBE58743	----C------C-NV-VSLK-D----Q---MC		GYPV---D-----D-G--R--SM-F-KS
	Babesia microti str. RI	829081855	----C--P---C-R--IQLG-NQ---Q---AC		GHFI---DV----CTE--R--SETF-HG
	Babesia sp. Xin.	1181627756	----C------C-NV-VSLK-D----Q---MC		GYPV---N-----D-S--R--SM-F-R-
**Other Apicom-plexa**	Besnoitia besnoiti	PFH36275	----R--P--I--RV-IQ-R-DQ---Q--EAA		GH-V-M--L------C--R-ASSTF-Q-
	Cystoisospora suis	PHJ25439	----R--P--I--RV-IQ-R--Q---Q--EAA		GH-V-M--V------S--K-ASSTF-Q-
	Hammondia hammondi	XP_008885237	----R--P--I--RV-IQ-R-DQ---Q--EAA		GH-V-M--V------C--K-ASSTF-Q-
	Neospora caninum Liv.	XP_003880657	----R--P--I--RV-IQ-R-DQ---Q--EAA		GH-V-M--V------C--K-ASSTF-Q-
	Toxoplasma gondii ME49	XP_018638008	----R--P--I--RV-IQ-R-DQ---Q--EAA		GH-V-M--V------C--K-ASSTF-Q-
	Cryptosporidium andersoni	OII71969	-------P---L-RI-IL-K-DI---Q---LN		GLPVKM--M------V--E--SKRF--G
	Cryptosporidium hominis TU502	XP_666063	-------P---L-RR-IERK-DI---Q---MS		GQQVVM--V------Q--A--SKHF-DG
	Cryptosporidium muris RN66	XP_002141731	-------P---L-RI-IL-K-DI---Q---LN		GLPVKM--M------V--E--SKRF--G
	Cryptosporidium parvum Iowa II	XP_627932	-------P---L-RR-IERK-DI---Q---MS		GQQVVM--V------Q--A--SKHF-DG
	Cryptosporidium ubiquitum	OII74527	-------P---L-RR-IERK-DI---Q---MS		GHQVIM--V------Q--A--SKHF-DG
	Gregarina niphandrodes	XP_011132233	----R--P-----KV-GDLH-DQ---Q--E-A		GH-I-M--V------R--E--SQA--DG
**Other Eukarya**	Thalassiosira oceanica	EJK46882	----Q--P--I-NVSG-TTKFD----Q--EEA		GHEVH--DT------M--L--R-IF-DG
	Gonium pectorale	KXZ56978	----Q--P--L--SA--ETLLE-------SQA		GTAVKM--V------E-RD--S--F-G-
	Ostreococcus lucimarinus CCE.	XP_001416216	-------P---K--N--QAKFD-------MVA		GMRC---TV------Q-RM--SSIF-S-
	Micromonas pusilla CCMP1545	XP_003059840	----Q--PS--L-TA-QGVSFQ------FTSL		GAEAV---V------E-RA--S-AF-EG
	Symbiodinium microadriaticum	OLP97942	----E--P--C--EI--E-K-D----Q---QT		QY-V-M-NT---------Q--SVNF-DG
	Ectocarpus siliculosus	CBJ28140	----N--P-----QGG-LSQ-D----Q--EAN		DHPVQM-DV------T--A--SATF-DG
	Klebsormidium nitens	GAQ79821	---HL--P---T-RL--E-R-D-------KEN		GHRSV--NT------E--RW-LGAF-GE
	Eutrema salsugineum	XP_006414482	-------P---I-TV-QDSG-GT-M------A		GYPV-M-KT------E-RS--SKEF-EE
	Vigna radiata var. rad.	XP_014490126	----A--P---I-DI--N-R-GT------MEA		GYPVKM-KT------E-RS--S-EF-KD
	Raphanus sativus	XP_018471146	-------A---I-TV-QASG-GT-M------A		GYPV-M-KT------E-RS--SKEF-ED

Fungi	*Aspergillus oryzae RIB.*	BAE62717	-------PP--L-KV-S-FQ-EQ---V-M-SN	HPRDVH--D-------E--AY-RNAF-DG
	Neurospora crassa	CAC10094	-------PP--L-QS-ARYG-DQ---V-M--N	HEKDVH--DT------E--S--RAAF-EG
	Sporisorium reilianum SR.	CBQ73179	----N--PP--I-QE-E-LG--Q---V-MFER	SPQAVH----------E--V--SKAF-DS
	Ustilago hordei	CCF54574	----N--PP--I-QQ-D-LG--Q---A-MFER	APQEVH----------E--L--AKAF-GS
	Pyronema omphalodes CBS 100.	CCX08263	----Q--PP--L-RY-A-FS-EK---V-M-EN	YPKNIH----------A--I--S-EF--A
	Lichtheimia corymbifera JMR.	CDH50232	----N--PP--I-QM-T-LQ-DQ---M----S	APDNVY----------A--A--SKLF--S
	Melanopsichium pennsylvanicum	CDI53642	----N--PP--I-QQ-E-LG--Q---V-MFER	SPQAVH----------E--V--SKAF-DS
	Penicillium roqueforti FM164	CDM27245	-------PP--L-KM-S-FQ-EQ---V-M--N	HPRDVH--D-------A-----SVTF-DG
	Sporisorium scitamineum	CDS01743	----N--PP--I-QE-E-LG--Q---V-MFER	SPQAVH----------E--V--SKAF-DS
	Parastagonospora nodorum SN.	XP_001798086	-------PP----KV-SR-Q--Q---A-ME-N	HPNDVH--DT------E--L--S-EF-DG
	Aspergillus oryzae RIB40	XP_001823850	-------PP--L-KV-S-FQ-EQ---V-M-SN	HPRDVH--D-------E--AY-RNAF-DG
	Coprinopsis cinerea oka.	XP_001835836	----Q--PP--I-QQ-ASKK-DQ---Q-FF-K	SPKAVH----------E--R--SKAF--D
	Laccaria bicolor S238N-H82	XP_001875791	----Q--PP--L-QE-CRYS-NQ---V----R	CPNAVH-------------R--S-VF-ES
	Pyrenophora tritici-repentis P.	XP_001935689	-------PP-I--KE-VRFR-AQ---M-M-QN	HPNDVH--DV------E--Q--SQTF-DG
	Aspergillus flavus NRRL3357	XP_002380905	-------PP--L-KV-S-FQ-EQ---V-M-SN	HPRDVH--D-------E--AY-RNAF-DG

Figure S26. A partial sequence alignment of the DNA2/NAM7 helicase protein showing a four/three amino acid insertion that is specific for members of *Plasmodium*.

Group	Species	Accession	335		380
***Plasmodium* (20/20)**	*Plasmodium berghei ANKA*	CDS46726	LRIPLLEGYKDNGIVAVGKIESGTL	Y	GSNMNCILMPNKVKVKVTNV
	Plasmodium sp. DRC-Itaito	SOV23311	--------------I-I--------	-	-N----T----------M--
	Plasmodium sp. Gor. Cla. G2	SOV15677	--------------I-I--------	-	-N----T----------M--
	Plasmodium chabaudi ada.	SCM21055	-------------------------	-	-N------------------
	Plasmodium chabaudi cha.	XP_735966	-------------------------	-	-N------------------
	Plasmodium coatneyi	XP_019914848	--------------I-I--------	-	-N--S-T-----L----L--
	Plasmodium cynomolgi str. B	XP_004222456	--------------I-I--------	-	-N--S-T-----L----L--
	Plasmodium falciparum 3D7	XP_001347916	--------------I-I--------	-	-N----T----------M--
	Plasmodium falciparum IGH-CR14	KNG77318	--------------I-I--------	-	-N----T----------M--
	Plasmodium fragile	XP_012335749	--------------I-I--------	-	-N--S-V-----L----L--
	Plasmodium gaboni	XP_018641152	--------------I-I--------	-	-N----T----------M--
	Plasmodium gallinaceum	CRG96282	--------------I----------	-	-N--S-T----------M--
	Plasmodium gonderi	GAW81002	--------------I----------	-	-N--S------------M--
	Plasmodium inui San Ant. 1	XP_008817956	--------------I----------	-	-N--S-T-----L----L--
	Plasmodium knowlesi str. H	XP_002259236	----------------I--------	-	-N--S-T-----L----L--
	Plasmodium malariae	SBS85533	--------------I---------I	-	-N--S-TI---------L--
	Plasmodium ovale curt.	SBS83633	--------------I---------I	-	-N----T----------L--
	Plasmodium ovale wal.	SBT35306	--------------I---------I	-	-N----T----------L--
	Plasmodium reichenowi	XP_012763636	--------------I-I--------	-	-N----T----------M--
	Plasmodium relictum	CRH00102	--------------I----------	-	-N--S-T----------M--
	Plasmodium vinckei pet.	EUD72841	-------------------------	-	-N------------------
	Plasmodium vinckei vin.	XP_008624776	-------------------------	-	-N------------------
	Plasmodium vivax Ind. VII	KMZ80479	--------------I----------	-	-N--S-T-----L----L--
	Plasmodium vivax North Kor.	KMZ99496	--------------I----------	-	-N--S-T-----L----L--
	Plasmodium vivax Sal-1	XP_001615349	--------------I----------	-	-N--S-T-----L----L--
	Plasmodium yoelii 17X	ETB57746	-------------------------	-	--------------------
	Plasmodium yoelii yoe. 17XNL	XP_724677	-------------------------	-	--------------------
***Piroplasmida* (0/8)**	*Babesia bigemina*	XP_012767072	----VTGA-R---VICM--V----V		T-GQT--V--H------Q--
	Babesia bovis T2Bo	XP_001609592	----ITGAF----V-CM--V----I		SMGQ---V--G-I-I-IQ--
	Babesia microti str. RI	XP_021338326	-----ID-------MCL--V-L--V		R-GQQ--I--DR--A--A--
	Babesia sp. Xin.	ORM41433	----ITGS-----V-CM--V----I		T-GQ--LV--G--R--IQ--
	Babesia ovata	GBE61941	----VTGA-R---VICM--V----V		T-GQT--V--Q------Q--
	Theileria orientalis	PVC59417	----VIDS-----V-CL--V---IV		RNGQS-VIL---S-TRIA-I
	Theileria annulata	XP_953171	----VIDT-RE--L-CL--V---MV		KTGQ---VV--RSRS--S-I
	Theileria equi	XP_004831080	----IIDS-R-----CL--V---V-		K-GQS-V-L-H-A---IP--
	Theileria parva str. Mug.	XP_764149	----VIDT-RE--L-CL--V---VV		KTGQ--VVV---SRS----I
**Other Apicom-plexa**	*Besnoitia besnoiti*	PFH36139	--V-I-D------VI-L--V-A---		TPG---------N---ILG-
	Eimeria necatrix	XP_013432810	----I-D------VT-L--V-A--V		TYG-EAL-L-Q-KR---SGI
	Eimeria tenella	XP_013228733	----I-D------VT-L--V-A--V		TYG-EAL-L-Q-KR---GG-
	Cyclospora cayetanensis	XP_022586986	----I-D------VT-L--V-A--V		TFG-TVV-----KR---SGI
	Cystoisospora suis	PHJ16172	--V-I-D------VI-L--V-A---		TPG---------N----LG-
	Hammondia hammondi	XP_008888004	--V-I-D----G-VI-L--V-A---		MPG---V---F-N-----G-
	Neospora caninum Liv.	XP_003880534	--V-I-D----G-VI-L--V-A---		TPG-------F-N-----G-
	Toxoplasma gondii ME49	XP_002369914	--V-I-D----G-V--L--V-A---		MPG---V---F-N-----G-
	Cryptosporidium andersoni	OII71573	V-----D--R---VI-M----L-II		NCGDSLVV--------IQSI
	Cryptosporidium hominis	CUV04563	I-----D--R---VI-M----L--I		RCGD-LVV---RA-A-IQSI
	Cryptosporidium muris RN66	XP_002141869	V-----D--R---VI-M----L-II		NCGDSLVV--------IQSI
	Cryptosporidium parvum Iowa II	XP_001388217	I-----D--R---VI-M----L--I		RCGD-LVV---RA-A-IQSI
	Cryptosporidium ubiquitum	OII71777	V-----D--R---VI-M----L--I		KCGD-LVV---RA-A-IQSI
	Gregarina niphandrodes	XP_011131144	--V---A--R---------V-G-VA		IVEQPALI---NI---I-G-
**Other Eukarya**	*Vitrella brassicaformis CCMP.*	CEL98130	------S----Q-VM-M--V-A-VV		KA-AH-L-----NR--ILS-
	Perkinsus marinus ATCC 50983	XP_002775705	----IMD--R-M-TI-M----Q-KA		KPGQQ-M-L---TITEIQS-
	Thecamonas trahens ATCC 50062	XP_013754290	--L-IADS-R-M-V-VM--L-A--I		ANGA-LV----QMP-E-IGL
	Phaeodactylum tricornutum CCA.	XP_002179052	----V-DR-T-R--ISM--V---II		RPG-KVTI--TRN-Y--DE-
	Rhizoctonia solani	CUA77442	M-IS-K---M-TIV---L---RI		YK-DSLL-----TN-E-AAI
	Rhizoctonia solani AG-3 Rhs.	EUC53884	M-IS-K---M-TIV---L---RV		YK-DSLL-----TT-E-AAI
	Jaapia argillacea MUCL 33604	KDQ64468	-MM-VS-K---M-TIV-------H-		RKGDSLV------T-E-AGI
	Echinococcus multilocularis	CDI97573	----ISDRF--M-TYI---------		VRGQILTM---R-P-E-LQI
	Echinococcus granulosus	CDS23970	----ISDRF--M-TYV---------		VRGQIL-M---R-P-E-LQI
	Hymenolepis microstoma	CDS32328	----ICDRF--M-TYV-------S-		TRG-TLTM---R-S-E-LQI
	Capitella teleta	ELU08696	V-M-VVNR-R-M-TIVL-------I		SKGQVLTM----FS-E-MQL
	Stylophora pistillata	PFX30123	----IS-R---M-T-VL--V---K-		NKGDSLL------S-E-LGI
	Nematostella vectensis	XP_001640097	V-L-IADK-R-M-TIVL--V----I		NKGQTLV------N-EL-T-

Figure S27. A partial sequence alignment of an Elongation factor Tu family protein showing a one amino acid insertion that is specific for members of *Plasmodium*.

Group	Organism	Accession	438		488
**Plasmodium (20/20)**	*Plasmodium berghei ANKA*	CDS50130	ALVGHTGSGKSTIAKLLYRFYD		AEGDIKIGGRNINKYNRNSIRSIIGIVPQ
	Plasmodium sp. DRC-Itaito	SOV25199	----------T--S--------		SK-E---------E-T-----N-------
	Plasmodium sp. Gor. Cla. G2	SOV19449	----------T--S--------		-K-E-------H--E-T-----N-------
	Plasmodium chabaudi ada.	SCM09467	----------------------		---------KCV-----K-----------
	Plasmodium chabaudi cha.	XP_741262	----------------------		---------KCV-N---K-----------
	Plasmodium coatneyi	XP_019916162	-I--------T--S--------		---E-------VTE-T---V-N-------
	Plasmodium cynomolgi str. B	XP_004224054	-I--------T--S--------		---E-------VSE-T-----N-------
	Plasmodium falciparum 3D7	XP_001348629	----------T--S--------		SK-E---------E-T-----N-------
	Plasmodium falciparum 7G8	EUR62931	----------T--S--------		SK-E---------E-T-----N-------
	Plasmodium falciparum FCH/4	ETW27184	----------T--S--------		SK-E---------E-T-----N-------
	Plasmodium falciparum HB3	KOB62451	----------T--S--------		SK-E---------E-T-----N-------
	Plasmodium falciparum IGH-CR14	KNG74758	----------T--S--------		SK-E---------E-T-----N-------
	Plasmodium falciparum Mal.	ETW46672	----------T--S--------		SK-E---------E-T-----N-------
	Plasmodium falciparum NF135/5.	ETW39786	----------T--S--------		SK-E---------E-T-----N-------
	Plasmodium falciparum Pal. Alt.	ETW54134	----------T--S--------		SK-E---------E-T-----N-------
	Plasmodium falciparum Tan.	ETW33827	----------T--S--------		SK-E---------E-T-----N-------
	Plasmodium falciparum Vie.	ETW15999	----------T--S--------		SK-E---------E-T-----N-------
	Plasmodium fragile	XP_012337440	----------T--S--------		Y--E--------AE-T---V-N-------
	Plasmodium gaboni	XP_018639536	----------T--S--------		SK-E---------E-T-----N-------
	Plasmodium gallinaceum	CRG94674	-I--------T--S--------		T--E--------S--T-----N-------
	Plasmodium gonderi	GAW82608	----------T--S--------		------------AE-T---V-N-------
	Plasmodium inui San Ant. 1	XP_008814149	----------T--S--------		---E-------VSE-T---V-N-------
	Plasmodium knowlesi str. H	XP_002260394	-I--------T--S--------		---E-------VTE-T---V-N-------
	Plasmodium malariae	SBS96407	-I--------T--S--------		---E-------VCD-T-----NV------
	Plasmodium ovale curt.	SBS86702	-I--------T--S--------		---E------T-SN-T---V-N-------
	Plasmodium ovale wal.	SBT43129	-I--------T--S--------		---E------T-SN-T---V-N-------
	Plasmodium reichenowi	CDO66943	----------T--S--------		SK-E---------E-T-----N-------
	Plasmodium relictum	CRH01447	-I--------T--S--------		---E---------E-T---V-N-------
	Plasmodium vinckei pet.	EUD71353	----------------------		---------KCV-N---K-----------
	Plasmodium vinckei vin.	XP_008622031	----------------------		---------KC--N---K-T---------
	Plasmodium vivax Bra. I	KMZ84961	-I--------T--S--------		---E-------VAE-T---V-N-------
	Plasmodium vivax Ind. VII	KMZ78883	-I--------T--S--------		---E-------VAE-T---V-N-------
	Plasmodium vivax Mau. I	KMZ91729	-I--------T--S--------		---E-------VAE-T---V-N-------
	Plasmodium vivax North Kor.	KMZ97838	-I--------T--S--------		---E-------VAE-T---V-N-------
	Plasmodium vivax Sal-1	XP_001615913	-I--------T--S--------		---E-------VAE-T---V-N-------
	Plasmodium yoelii 17X	ETB56224	----------------------		---------K-V-----------------
	Plasmodium yoelii yoe. 17XNL	XP_726637	----------------------		---------K-V-----------------
**Piroplasmida (0/8)**	*Theileria annulata*	84995074	-I--DS-------C---F----	P	NS-S-L-D-QD-KTVTLK-L-NTL-----
	Theileria equi	510900591	-I--NS-------C-M-F----	P	TA-S-F-D-QD-KTCTLK-L--SL-----
	Theileria orientalis str. Shi.	697890487	-I--DS-------S---F----	P	TS-T-L-D-QD-KNVTLR-L-DSL-----
	Theileria parva str. Mug.	71030768	-I--DS-------F---F----	P	NS-S-L-DDQD-KNVTLK-L-NTL-----
	Babesia bigemina	833490787	-I--NS-T--T----M-F---E	P	SA-Q-L-D-QD-RDVTIS-L-NA------
	Babesia bovis	156088429	-I--DS---------M-F-L--	P	S--Q-L-D-QD-KNVTLS-L-NS------
	Babesia microti str. RI	1206243296	-II-A-----T--SR--C----	V	DS-A-R-NST--AHVTQ--L--C------
	Babesia ovata	1314816607	-I--NS-T--T----M-F---E	P	SA-Q-L-D-QD-RDVTLG-L-NA------
	Babesia sp. Xin.	1181627922	-I--DS-T--T------F-L--	P	SQ-Q-L-D-QD-KHVTLS-L-NA------
**Other Apicomplexa**	*Besnoitia besnoiti*	1261482108	-I--SS-A----LI-----M--	P	KH-TVR-D-QDVKELDLT-F-QH--V---
	Cyclospora cayetanensis	1249156206	----ES----T-LIR----LA-	P	LR-VVS-D-QDLKTLPMM-F-RH--V---
	Eimeria acervulina	915007233	----ES----T-LIR----LA-	P	LS-VVS-D-QDLRTLPIM-F-QR--V---
	Eimeria brunetti	557172447	----ES----T-LIR----LA-	P	LS-VVS-D-QDLKTLPML-F-KH--V---
	Eimeria maxima	915134024	----GS-C-----LQ--Q-V--	C	EK-SVT---VP-ENI-VRTL-RQM-V-S-
	Eimeria necatrix	921130364	----ES----T-LIR----LA-	P	LS-VVS-D-QDLKTLPAM-F-RH--V---
	Eimeria tenella	916420280	----ES----T-LIR----LA-	P	LS-VVS-D-QDLKTLPAM-F-RH--V---
	Hammondia hammondi	675133470	-I--QS-A----L---MH----	P	Q--T-LLN--D-RTLRLHDL--R--F-E-
	Neospora caninum Liv.	401413496	-I--RS-A----L---MH----	P	Q--S-LLN-VD-RSLRLHHL--R--F-E-
	Toxoplasma gondii ME49	1085182642	-I--QS-A----L---MH----	P	Q--A-LLN--D-RTLRLRDL--R--F-E-
	Cryptosporidium hominis TU502	67597739	F--SS----T-L---I--IFE	P	NS-K-R-F-KE-ED-EI-EY-NCFAVL--
	Cryptosporidium parvum Iowa II	66362026	F--SS----T-L---I--IFE	P	NS-K-R-F-KE-ED-EI-EY-NCFAVL--
	Gregarina niphandrodes	749163221	----AS-C----VIQ-VM----	P	QS-LVMLD--PLTE--VKAL-ET-AW-G-
**Proteo-bacteria**	*Hoeflea phototrophica DFL-43*	EDQ32461	-I--PS-A-----SR-------	I	QS-A-S-D-Q-V-DVTQK-L-AA--M---
	Acetobacteraceae bac. AT.	EHM03552	-I--P--A-----SR--F----	A	QS-Q-R-D-HD-RQVTQE-L-HA--V---
	Caedibacter sp. 38-128	OJX07684	----PS-------S---F---E	A	NS-E-I---KD-RDFSLS-LTEV------
	Sphingobium sp. 66-54	OJY69811	-I---S-A------R-------	I	QG-R-L-D-Q--AQVQQA-L-HV------
	Rhodospirillaceae bac. TM	OUT78987	-I--SS-------SR-------	V	TN-S-S-D-QD-KSIAQ--L-NA------
	Candidatus Endolissoclinum sp.	OUU53809	-I--SS-A------R--F----	V	SS-K-E-D--D-RDITQKNL-D-------
	Acidocella sp. 20-61-6	OYV41090	-I--P--------SR--F----	T	VS-TVRVD-HDVRD-TQH-L-GA--V---
	Rhodospirillales bac. 20.	OYV51911	----P-------VSR--F----	V	TG-E-LVD-T-VRDITQT-L-GA--V---
	Sphingomonadales bac. 32.	OYX76403	-V---S-A----LSRI------	I	KG-QVR-D-QD-AHVTQD-L-RA------
	SAR116 cluster bac. MED-G	PDH55539	-I--PS----T--SR--F----	P	KT-IVR-D-Q-LTEVTQS-V--S--M---
	Sandarakinorhabdus sp. AAP62	WP_017666631	-V---S-A----LSRI------	I	QG-EVS-D-QD-AHVTQD-L-RA------
	Mesorhizobium sp. LC103	WP_047143735	-I--PS-A-----SR--F----	I	Q--A-R-D-QDVRDVTQE-L-NV--M---
	Hoeflea phototrophica	WP_052093209	-I--PS-A-----SR-------	I	QS-A-S-D-Q-V-DVTQK-L-AA--M---

Group	Species	Accession			
Other Prokarya	*Streptococcus equi subsp. zo.*	ACG62437	-----------S-MN-------	V	QS-L-TVD-Q--QE-S-E-L--QM---L-
	Streptococcus sp. F0442	WP_009731444	-----------S-MN-------	P	Q--E-R-D-Q--RHFS-E-L--HM---L-
	Streptococcus equi	WP_012677880	-----------S-MN-------	V	QS-L-T-D-Q--QE-S-E-L--QM---L-
	Streptococcus suis	WP_015646585	-----------S-MN-------	P	Q--RVR-D-K--RD-S-E-L--HM---L-
	Streptococcus ovis	WP_018377096	-----------S-MN-------	P	QK-K-W-D-QD-RE-S-E-V--QM---L-
	Paenisporosarcina sp. TG20	WP_019412761	-----------S-MN--F----	V	SK-A-T-D----QDLS-QT--DHM---L-
	Streptococcus sp. 1171_SSPC	WP_048789579	-----------S-MN-------	P	Q--E-R-D-Q--RHFS-E-L--HM---L-
	Planococcus massiliensis	WP_052652305	-----------S-MN--F----	V	T--A---D-M---DMT-QAV-EHM---L-
	Desnuesiella massiliensis	WP_055670063	----S--A--T--VN--T---E	V	SQ-E-L-D-K--KE-T-D-L-KCF---L-
	Streptococcus sp. A12	WP_061564223	-----------S-MN-------	P	Q--E-R-D-Q--RHFS-E-L--HM---L-
	Streptococcus marmotae	WP_067088488	----------TS-MN-------	P	QK-QVQ-D-Q--RD-S-E-V--HM---L-
	Cohnella sp. OV330	WP_090115850	----P--A--T--VQ--N----	V	TG-E-R----D-RD-T-S-L--AF-V-L-
	Arenibacter troitsensis	WP_085500326	----QS--------N-MT----	V	N--E---D-VD-RDFTK--L-NLM-L-T-
	Methanobrevibacter ruminantium	WP_012956426	-I--E--A--T--V---M----	V	DD-E---D-V---S-DKH-V--LV-M-L-
	Methanobrevibacter millerae	WP_058738894	-II-E--A--T--V---M----	V	DS-E---D-V---E-DK--L--L--M-L-
	Methanobrevibacter sp. YE315	WP_067042832	-I--E--A--T--V---M----	V	NS-E---D-V---R-DKH----LV-M-L-
	Methanobrevibacter olleyae	WP_067147102	-I--E--A--T--V---M----	V	DS-E---D-V---E-DK--L--LV-M-L-
	Methanosphaera sp. WGK6	WP_069592560	-I--K--A--T--I---M----	I	TS-S---D-K--EE-TK--L--K--M-L-
	Methanobrevibacter olleyae	WP_074797974	-I--E--A--T--I---M----	V	DS-E---D-V---E-DK--L--LV-M-L-
Other Eukarya	*Blastomyces dermatitidis ATCC*	EGE78059	----S--A---SLM---L----	V	NS-C---D--D-RDVTQR-L-NV--V---
	Macrophomina phaseolina MS6	EKG20220	----E-------VL---L----	V	TK-S--VD-QDLRDVTLD-L-EV--V---
	Emmonsia parva UAMH 139	KLJ11445	----Q-----T--LQM-N----	P	TS-SV----QD-SEVTLE-L--N--V---
	Kwoniella heveanensis BCC8398	OCF30343	----E--------LR-------	V	TS-H-Y-D-QD-SQVTQR-L-HA------
	Kwoniella heveanensis CBS 569	OCF39600	----E--------LR-------	V	TS-H-Y-D-QD-SQVTQR-L-HA------
	Kwoniella mangroviensis CBS 1.	OCF58072	----E--------L--I----E	V	SS-R-L-D-QD-S-VSQA-L-NV------
	Emmonsia parva	PGH00217	----S--A---SLM---L----	I	TS-S---D-HD-RDVTQ--L-EV--V---
	Aspergillus clavatus NRRL 1	XP_001268006	----A--A--TS-T---L-Y--	V	DS-S-R-D--D-RDVTQG-L-DV--V---
	Blastomyces gilchristii SLH.	XP_002624267	----S--A---SLM---L----	V	NS-C---D--D-RDVTQR-L-NV--V---
	Malassezia pachydermatis	XP_017991955	----ES------VL--------	V	SR-R-L-D-QD-RDVTQQ-L-KS------
	Cutaneotrichosporon oleag.	XP_018274906	----ES-------LR-------	I	DS-S---D-QD-S-VTQK-L-HA------
	Kwoniella mangroviensis CBS 85	XP_019004689	----E--------L--I----E	V	SS-R-L-D-QD-S-VSQA-L-NV------
	Auxenochlorella protothecoides	XP_011399202	----A--------LR--F----	P	SA-G-YV--KD-SHVTQA-L-AV--V---
	Saccoglossus kowalevskii	XP_006824110	-----S-A-----VR--F----	I	QG-C-R-D-KD-SEVTQT-L-KT--V---
	Aplysia californica	XP_012938229	-------------VR--F----	V	QS-C---D-QD-T-VTQK-L-DS--V---
	Lingula anatina	XP_013410961	-----S-------IR--F----	V	TS-T-R-D-QD-SQVQQE-V-KA--V---
	Varroa destructor	XP_022655264	-------------LR--L----	V	QS-S-L-D-Q--SAVTQR-L--H--V---
	Pythium insidiosum	GAX96586	----E--A--T--SR--F---E	C	DA-K-LVNHE--REVTQQ-L-QV------
	Ectocarpus siliculosus	CBN74933	-------A--T--SR--F----	P	VK-M-LMN-HD-KAATQQ-V-RA--V---

Figure S28. A partial sequence alignment of the Multidrug resistance protein 2 showing a one amino acid deletion that is specific for members of *Plasmodium*.

Group	Species	Accession	1174		1230
***Plasmodium* (20/20)**	*Plasmodium berghei ANKA*	XP_022714222	ESHFDLELRAAVMHDIIDMIPAGLKNNK	GK	ARLILQHLSEAWRCWKANIPWKVVGLP
	Plasmodium sp. DRC-Itaito	SPJ08735	---------------------E------	--	---------------------------
	Plasmodium sp. gorilla clade G2	SOV11188	---------------------E------	--	---------------------------
	Plasmodium chabaudi ada.	SCM06549	----------------------------	--	---------------------R-----
	Plasmodium chabaudi cha.	CAH77525	----------------------------	--	---------------------R-----
	Plasmodium coatneyi	XP_019912805	----------------------------	--	---------------------------
	Plasmodium cynomolgi str. B	XP_004220850	----------------------------	--	---------------------------
	Plasmodium falciparum 3D7	XP_001351366	---------------------E------	--	---------------------------
	Plasmodium falciparum 7G8	EUR46738	---------------------E------	--	---------------------------
	Plasmodium falciparum CAMP/Mal	ETW58140	---------------------E------	--	---------------------------
	Plasmodium falciparum Dd2	KOB84899	---------------------E------	--	---------------------------
	Plasmodium falciparum FCH/4	ETW32191	---------------------E------	--	---------------------------
	Plasmodium falciparum HB3	KOB58992	---------------------E------	--	---------------------------
	Plasmodium falciparum IGH-CR14	KNG77101	---------------------E------	--	---------------------------
	Plasmodium falciparum Mal.	ETW51282	---------------------E------	--	---------------------------
	Plasmodium falciparum Pal. Alt.	ETW57538	---------------------E------	--	---------------------------
	Plasmodium falciparum RAJ116	KNC35375	---------------------E------	--	---------------------------
	Plasmodium falciparum San. Lu.	EUT91632	---------------------E------	--	---------------------------
	Plasmodium falciparum Tanzania	ETW32981	---------------------E------	--	---------------------------
	Plasmodium falciparum UGT5.1	EWC78565	---------------------E------	--	---------------------------
	Plasmodium falciparum Vie.	ETW20357	---------------------E------	--	---------------------------
	Plasmodium fragile	XP_012335046	----------------------------	--	---------------------------
	Plasmodium gaboni	XP_018643283	---------------------E------	--	---------------------------
	Plasmodium gallinaceum	CRG98126	----------------------------	S-	---------------------------
	Plasmodium gonderi	GAW79234	----------------------------	--	---------------------------
	Plasmodium inui San Ant. 1	XP_008818570	----------------------------	--	---------------------------
	Plasmodium knowlesi str. H	XP_002261014	----------------------------	--	---------------------------
	Plasmodium malariae	SBT70343	----------------------------	S-	---------------------------
	Plasmodium ovale curt.	SBS80186	----------------------------	S-	---------------------------
	Plasmodium ovale wal.	SBT31059	----------------------------	S-	---------------------------
	Plasmodium reichenowi	XP_019970770	---------------------E------	--	---------------------------
	Plasmodium relictum	CRG98542	----------------------------	S-	---------------------------
	Plasmodium vinckei pet.	EUD71892	----------------------------	--	---------------------R-----
	Plasmodium vinckei vin.	XP_008625065	----------------------------	--	---------------------R-----
	Plasmodium vivax Ind. VII	KMZ82613	----------------------------	--	---------------------------
	Plasmodium vivax North Kor.	KNA01897	----------------------------	--	---------------------------
	Plasmodium vivax Sal-1	XP_001613367	----------------------------	--	---------------------------
	Plasmodium yoelii 17X	ETB59894	----------------------------	--	---------------------R-----
***Piroplasmida* & Other Apicomplexa (0/8)**	*Babesia sp. Xinjiang*	ORM40251	----------------L--M-E-IRA--		--T--------------------PDM-
	Babesia ovata	GBE62384	----------------L--M-E-IRA--		--T--------------------PEM-
	Babesia bigemina	XP_012769723	----------------L--M-E-IRA--		--T--------------------PEM-
	Babesia bovis T2Bo	XP_001610701	----------------L--M-E-IRA--		--T--------------------PEM-
	Babesia microti strain RI	XP_021338655	---------------V---M-E-V-AS-		-KT----------S---------P---
	Theileria annulata	XP_954981	----------------L--M-E-IRAS-		S-T--------------------PQ--
	Theileria parva strain Muguga	XP_763310	----------------L--M-E-IRAS-		S-T--------------------PQ--
	Theileria orientalis	PVC56079	----------------L--M-E-IRAS-		-KT-------------S------PE--
	Theileria equi strain WA	XP_004832404	--K------G---N--K--M-V--RAT-		VNT-F---C--------------PQ--
	Toxoplasma gondii ME49	XP_002368068	----------------L-TM-E-V-A--		--T--------------------P---
	Neospora caninum Liv.	XP_003883442	----------------L-TM-E-V-A--		--T--------------------P---
	Hammondia hammondi	XP_008885662	----------------L-TM-E-V-A--		--T--------------------P---
	Eimeria tenella	XP_013231526	----------------L-LM-E-V-AS-		--T--------------------P---
	Eimeria necatrix	XP_013434282	----------------L-LM-E-V-AS-		--T--------------------P---
	Eimeria brunetti	CDJ52439	----------------L-IM-E-V-AS-		--T--------------------P---
	Eimeria acervulina	XP_013246946	----------------L-IM-E-V-AS-		--T--------------------P---
	Cystoisospora suis	PHJ21275	----------------L--M-E-V-A--		--T--------------------P---
	Besnoitia besnoiti	PFH38612	----------------L-TM-E-V-A--		--T--------------------P---
**Other Eukarya**	*Monoraphidium neglectum*	XP_013891308	----------------L--M-E-V-Q--		-KT--------------------P---
	Vitrella brassicaformis CCMP.	CEM02373	----------------L--M-E-V-A--		--T--------------------P---
	Symbiodinium microadriaticum	OLP96264	-------------------M-E-VRA--		--T--------------------P---
	Wuchereria bancrofti	EJW86331	----------------L--M-E-I-Q--		--V--------------------P---
	Colinus virginianus	OXB72829	----------------L--M-E-V-A--		--T--------------------P---
	Ancylostoma duodenale	KIH51314	----------------L--M-E-I-Q--		--V--------------------P---
	Varroa jacobsoni	XP_022688475	----------S--------M-E-I-Q--		--T--------------------P---

Figure S29. A partial sequence alignment of the Pre-mRNA-processing-splicing factor 8 protein showing a two amino acid insertion that is specific for members of *Plasmodium*

			218		272
***Plasmodium* (20/20)**	*Plasmodium berghei ANKA*	CDS46715	YRDHVHAISKNVPIKEILNELYGNYYGST	N	HGKGGSMHIYSKKNNFIGGFGFIGE
	Plasmodium sp. Gor. Cla. G2	SOV15698.1	-------L--G-HANK-------------	-	K-----------E------------
	Plasmodium sp. DRC-Itaito	SPJ10988.1	-------L--G-HANK-------------	-	K-----------E------------
	Plasmodium chabaudi ada.	SCM21085	-------L----SA---------------	-	Q------------------------
	Plasmodium chabaudi cha.	XP_744565	-------L----SA---------------	-	Q------------------------
	Plasmodium coatneyi	XP_019914944	-------------PRKV------------	-	R-----------SE-----------
	Plasmodium cynomolgi str. B	XP_004222467	------------SP-KV------------	-	S-----------SE-----------
	Plasmodium falciparum 3D7	XP_001347927	-------L--G--AHK-------------	-	K-----------E------------
	Plasmodium falciparum FCH/4	ETW30350	-------L--G--AHK-------------	-	K-----------E------------
	Plasmodium falciparum Mal.	ETW45855	-------L--G--AHK-------------	-	K-----------E------------
	Plasmodium falciparum Vie.	ETW15452	-------L--G--AHK-------------	-	K-----------E------------
	Plasmodium fragile	XP_012335761	-------------P-KV------------	-	K-----------GE-----------
	Plasmodium gaboni	XP_018641164	-------L--G-HANK-------------	-	K-----------E------------
	Plasmodium gallinaceum	CRG96293	------------SPNK-------------	-	K--------F---E-----------
	Plasmodium gonderi	GAW81013	------------AP-KV------------	-	R-----------DH-----------
	Plasmodium inui San Ant. 1	XP_008817945	-------------P-KV------------	-	R-----------SE-----------
	Plasmodium knowlesi str. H	XP_002259247	-------------P--V------------	-	R-----------RE-----------
	Plasmodium malariae	SBS85566	----------G--P--V------------	-	K-----------TQ-----------
	Plasmodium ovale	SCP04572	----------T--PARV------------	-	G-----------EH-----------
	Plasmodium ovale curt.	SBS83654	----------T--PARV------------	-	G-----------EH-----------
	Plasmodium ovale wal.	SBT35320	----------T--PAKV------------	-	G-----------EH-----------
	Plasmodium reichenowi	XP_012763647	-------L--G-HAHK-------------	-	K-----------E------------
	Plasmodium relictum	CRH00113	-------------PNK---------N---	-	K--------F---E-----------
	Plasmodium vinckei pet.	EUD72852	-------L----SA---------------	-	Q------------------------
	Plasmodium vinckei vin.	XP_008624787	-------L----SA---------------	-	Q------------------------
	Plasmodium vivax Ind. VII	KMZ80490	-------------PRK-------------	-	R-----------SE--V--------
	Plasmodium vivax Sal-1	XP_001615360	-------------PRK-------------	-	R-----------SE--V--------
	Plasmodium yoelii 17X	ETB57729	-------------V---------------	-	Q---------N--------------
	Plasmodium yoelii yoe. 17XNL	XP_727097	-------------V---------------	-	Q---------N--------------
Piliocolobus	*Piliocolobus tephrosceles*	1297723042	-------L----SV-KV------------	-	K---------D-EK-----------
**Other Apicomplexa**	*Neospora caninum Liv.*	XP_003885894	-------T--G--VR-VMA--F-KAT-CS		K-R-----MF--EH-M----A----
	Cystoisospora suis	PHJ21125	-------T--G--VR-VMA--F-KAT-CS		K-R-----MF--EH-M----A----
	Hammondia hammondi	XP_008883669	-------T--G--VR-VMA--F-KAT-CS		R-R-----MF---H-M----A----
	Eimeria necatrix	XP_013438716	-------T--G--AR-VFA--F-KTT-CS		K-F-----MF--EW-LY---A----
	Eimeria tenella	XP_013231024	-------T--G--AR-VFA--F-KTT-CS		K-F-----MF--EW-LY---A----
	Eimeria mitis	XP_013350004	-------T--G--AR-VFA--F-KQT-CS		K-F-----MF--EW-LY---A----
	Eimeria brunetti	CDJ47615	-------T--G--ARQVFA--F-KAT-CS		K-F-----MF--EW-MY---A----
	Eimeria maxima	XP_013337887	-------T--G--PR-VFA--F-KKT-CS		K-F-----MF--EW-LY---A----
	Besnoitia besnoiti	PFH38339	-------T--G--VR-VMA--F-KAT-CS		K-R-----MF--PH-M----A----
	Toxoplasma gondii ME49	XP_002366997	-------T--G--VR-VMA--F-KAT-CS		R-R-----MF---H-M----A----
**Cyanobacteria**	*Prochlorococcus marinus str. M.*	KZR70120	-------L-AG--AR-VMS--F-KET-CS		K-R-----LF-QEHHLL---A----
	Synechococcus sp. TMED90	OUX72890	-------L-AG--AR-VMS--F-KET-CS		K-R-----LF--EHHLL---A----
	Synechococcus sp. WH 7805	WP_006041926	-------L-AG--AR-VMS--F-KET-CS		K-R-----LF--EHHLL---A----
	Thermosynechococcus sp. NK.	WP_024124449	-------L-AG--AR-VMA--F-KAT-CS		K-R-----LF-A-H--L---A-VA-
	Leptolyngbya sp. KIOST-1	WP_035985435	-------L-SG--A-NVMA--F-KET-CS		K-R-----LF-SEH-LL---A----
	Prochlorococcus sp. MIT 13.	WP_063397204	-------L-AG--AR-VMS--F-KET-CS		K-R-----LF-QEHHLL---A----
	Phormidium tenue	WP_073607834	-------L-AG--A-NVMA--F-KET-CS		K-R-----LF-SEH-ML---A----
**Rhodophyta**	*Cyanidiaceae sp. MX-AZ01*	AIA61192	-------L--G--P-QVMA--F-KQT-CS		K-R-----LF-AAHG-L---A----
	Porphyra purpurea	NP_053877	-------L--G--SQNVMA--F-KET-CS		R-R------F-APH--L---A--A-
	Pyropia perforata	YP_009027551	-------L--G--S-NVMA--F-KET-CS		K-R------F-APH--L---A--A-
	Porphyra umbilicalis	YP_009413280	-------L--G--SQNVMA--F-KET-CS		K-R------F-APH--L---A--A-
	Erythrotrichia carnea	YP_009297436	-------L--G-SSR-VMA--F-KET-CS		K-R------F-QPH--L---A----
	Membranoptera tenuis	YP_009332916	-------L--G--ARSVMA--F-KET-CS		R-R------F-S-H--L---A----
	Gracilaria firma	YP_009346843	-------L--G--A-LVMA--F-KET-CS		R-R------F-APH--L---A----
	Sheathia arcuata	YP_009390111	-------L--G--AN--MA--F-KET-CS		R-R------F-S-Y--L---A----
	Dipterocladia arabiensis	YP_009391254	-------L--G--A--VMA--F-KET-CS		R-R------F-A-H--L---A----
	Platysiphonia delicata	YP_009391456	-------L--G-SSN--MS--F-KET-CS		R-R------F-A-H--L---A----
	Acrosorium ciliolatum	YP_009391869	-------L--G--A-SVMA--F-KET-CS		--R------F-A-H--L---A----
	Caloglossa monosticha	YP_009392495	-------L--G--A-MVMA--F-KET-CS		R-R------F-APH--L---A----
	Bostrychia tenella	YP_009392696	-------L--G--S-Y-MA--F-KET-CS		R-R------F-SQH--L---A----
	Caloglossa intermedia	YP_009392905	-------L--G--S-IVMA--F-KET-CS		R-R------F-APH--L---A----
	Bostrychia moritziana	YP_009393113	-------L--G--ANCVMA--F-KET-CS		K-R------F-SAHK-L---A----
	Bostrychia simpliciuscula	YP_009393534	-------L--G--S-YVMA--F-KET-CS		R-R------F-A-H--L---A----
	Herposiphonia versicolor	YP_009395804	-------L--G--P-NVMA--F-KET-CS		K-R------F-AQH--L---A----
	Dasya naccarioides	YP_009396007	-------L--G--AESVMA--F-KET-CS		--R------F-SQH--L---A----
	Gracilaria tenuistipitata var.	YP_063628	-------L--G--ANL-MA--F-KET-CS		R-R------F-AAH--L---A----
	Corynoplastis japonica	ARO90960	-------L--G--SR-VMA--F-KET-CC		K-R------F-APH--L---A----

	Oryza sativa	AAL83994	-------L--G--ARSVMA--F-KAT-CC	R-Q-----MF-EPH-LL---A----
	Glycine max	ACU23659	-------L--G--SR-VMS--F-KAT-CC	R-Q-----MF--EH-LL---A----
	Gentiana triflora	BAQ95526	-------L--G--ARQVMS--F-KTT-CC	R-Q-----MF--EH-VL---A----
	Coffea canephora	CDP08620	-------L--G--ARAVMS--F-KTT-CC	R-Q-----MF--EH-LL---A----
	Genlisea aurea	EPS65417	-------L--G--ARQVMS--F-KTT-CC	R-Q-----MF--EH-LL---A----
	Trifolium subterraneum	GAU33604	-------L--G--SRAVMS--F-KAT-CC	R-Q-----MF--EH-VL---A----
	Cephalotus follicularis	GAV75109	------SL--G--ARAVMS--F-KTT-CC	N-Q-----MF--EH-V----A----
	Citrus sinensis	KDO86616	-------L--G--ARAVMS--F-KAT-CC	R-Q-----MF--EH-LL---A----
	Glycine soja	KHN06367	-------L--G--SRQVMS--F-KAT-CC	R-Q-----MF--EH-LL---A----
	Marchantia polymorpha subsp. r.	OAE24279	-------L--G--ARLVMA--F-KST-CC	R-Q-----MF--EHGLL---A----
	Lupinus angustifolius	OIW02357	-------L--G--ARSVMS----KAT-IC	R-Q-----MF--EH-L----A----
**Streptophyta**	*Corchorus capsularis*	OMO64187	-------L--G--AR-VMS--F-KTT-CC	R-Q-----MF-SEH-LL---A----
	Corchorus olitorius	OMO82356	-------L--G--AR-VMS--F-KTT-CC	R-Q-----MF-SEH-LL---A----
	Macleaya cordata	OVA04699	-------L--G--AR-VMS--F-KAT-CC	R-Q-----MF--EH-LL---A----
	Punica granatum	OWM85162	-------L--G--AR-VMS--F-KTT-CC	R-Q-----MF--EH-LL---A----
	Physcomitrella patens	XP_001775995	-------L--G--ARQVMA--F-KST-CC	R-Q-----MF-AEHGLL---A----
	Vitis vinifera	XP_002267676	-------L--G--ARAVMS--F-KAT-CC	R-Q-----MF--EH-VL---A----
	Populus trichocarpa	XP_002301442	-------L--G--ARAVMS--F-KTT-CC	R-Q-----MF--EH-L----A----
	Hevea brasiliensis	XP_021676041	-------L--G--ARAVMS--F-KTT-CC	R-Q-----MF--EH-LL---A----
	Prunus avium	XP_021822645	------SL--G--AR-VMS--F-KAT-CC	R-Q-----MF--EH-VL---A----
	Spinacia oleracea	XP_021865703	-------L--G--ARNVMS--F-KAT-CC	R-Q-----MF--EH-VL---A----
	Carica papaya	XP_021887556	-------L--G--ARAVMS--F-KTT-CC	R-Q-----MF--EH-VL---A----
	Momordica charantia	XP_022159128	-------L--G--AR-VMS--F-KTT-CC	R-Q-----MF--EH-L----A----

Figure S30. A partial sequence alignment of the Pyruvate dehydrogenase E1 component, alpha subunit protein showing a one amino acid insertion that is specific for members of *Plasmodium* and *Piliocolobus tephrosceles*. The presence of this CSI within the latter species is likely due to contamination. CSI region is not conserved in *Piroplasmida*.

Group	Organism	Accession	406		451
**Plasmodium (20/20)**	*Plasmodium berghei ANKA*	CDS46715	KVDGNDVLSIYKLAKKKINEIR	K	KKSGPILIEAITYRTRGHSLADP
	Plasmodium sp. Gor. Cla. G2	SOV15698.1	------------------QQ--	N	RT---VI-------AK-------
	Plasmodium sp. DRC-Itaito	SOV23322.1	------------------QQ--	N	RT---VI-------AK-------
	Plasmodium chabaudi ada.	SCN60421	------------IV----Y---	-	-----V-----------------
	Plasmodium chabaudi cha.	SCN60422	------------IV----Y---	-	-----V-----------------
	Plasmodium coatneyi	XP_019914944	------AIG----------QM-	R	REC--V--------AK-------
	Plasmodium cynomolgi str. B	XP_004222467	------AIGL---------QM-	R	REC--V--------AK-------
	Plasmodium falciparum 3D7	XP_001347927	--------T---------QQ--	N	RT----I-------AK-------
	Plasmodium falciparum FCH/4	ETW30350	--------T---------QQ--	N	RT----I-------AK-------
	Plasmodium falciparum Mal.	ETW45855	--------T---------QQ--	N	RT----I-------AK-------
	Plasmodium falciparum Vie.	ETW15452	--------T---------QQ--	N	RT----I-------AK-------
	Plasmodium fragile	XP_012335761	------AIALFR-------QM-	R	REC--V--------AK-------
	Plasmodium gaboni	XP_018641164	------------------QQ--	N	RT---VI-------AK-------
	Plasmodium gallinaceum	CRG96293	-L------T----T----DQ--	-	R-C--V--------QK-------
	Plasmodium gonderi	GAW81013	RI------R----M---VK-M-	-	REC--V--------AK-------
	Plasmodium inui San Ant. 1	XP_008817945	------AIAL---------QM-	R	REC--V--------A--------
	Plasmodium knowlesi str. H	XP_002259247	-------IGL---------QM-	R	REC--V--------AK-------
	Plasmodium malariae	SBS85566	-I-----------------D--	N	R-C--V--------SK-------
	Plasmodium ovale curt.	SBS83654	--------G---------T---	-	R-----I--------K-------
	Plasmodium ovale wal.	SBT35320	--------G---------T---	-	R-----I----------------
	Plasmodium reichenowi	CDO65048	--------T----T----QQ--	N	RT----I-------AK-------
	Plasmodium relictum	CRH00113	-I----------------DQ--	-	R-C-----------LK-------
	Plasmodium vinckei pet.	EUD72852	------------IV--------	-	-----V-----------------
	Plasmodium vinckei vin.	XP_008624787	------------IV--------	-	-----V-----------------
	Plasmodium vivax Ind. VII	KMZ80490	------AI-----------QM-	R	REC--V--------AK-------
	Plasmodium vivax Sal-1	XP_001615360	------AI-----------QM-	R	REC--V--------AK-------
	Plasmodium yoelii 17X	ETB57729	----------------------	-	-----------------------
	Plasmodium yoelii yoe. 17XNL	XP_727097	----------------------	-	-----------------------
Piliocolobus	*Piliocolobus tephrosceles*	XP_023055362	--------N---------K---	-	RQ---V---------K-------
**Other Apicomplexa**	*Cyclospora cayetanensis*	XP_022592078	E---M---AVRAA-RQA-DRA-		NGE--S----L---F--------
	Eimeria acervulina	XP_013253058	E---M---AVRAA-RQA-ERA-		-GE--S----L---F--------
	Eimeria brunetti	CDJ47615	E---M---AVRAA-RQA-ERA-		-GE--S----L---F--------
	Eimeria maxima	XP_013337887	E---M---AVRAA-RQA-ERA-		RGE--S----L---F--------
	Eimeria mitis	XP_013350004	E---M---AVRAA-RQA-DRA-		RGE--S----L---F--------
	Eimeria necatrix	XP_013438716	E---M---AVRAA-RQA-ERA-		-GE--S----L---F--------
	Eimeria praecox	CDI80905	E---M---AVRTA-RQA-ERA-		RGE--S----L---F--------
	Eimeria tenella	XP_013231024	E---M---AVRAA-RQA-ERA-		-GE--S----L---F--------
**Cyanobacteria**	*Cyanobacterium stanieri PCC.*	AFZ46314	E---M---AVRDV-Q-A-ARA-		AGE--T----L---F--------
	Calothrix sp. NIES-4071	BAZ11046	E---M---AV-SA--EAVRRA-		AGE--T----L---F--------
	Crocosphaera watsonii WH 0402	CCQ68831	E---M---AVR-V-QEAVDRA-		AGE--T----L---F--------
	Candidatus Atelocyanobacterium	KFF41119	E---M---AVRQV--EA-ARA-		AGE--T----L---F--------
	Oscillatoriales cyanobacterium	OCR01703	E---M---AVREV--EAVARA-		AGE--T----L---F--------
	Roseofilum reptotaenium AO1.	OJJ26208	E---M---AVREV--EA-ARA-		AGE--TV---L---F--------
	Coleofasciculus chthonoplastes	WP_006102809	E---M---AVHSV-QEAVARA-		AGE--T----L---F--------
	Arthrospira	WP_006623993	E---M---AVREV-Q-A-ARA-		AGE--T----L---F--------
	Microcoleus vaginatus	WP_006634162	E---M---AVREV--EAVARA-		AGE--T----L---F--------
	Crocosphaera watsonii	WP_007307445	E---M---AVR-V-QEAVDRA-		AGE--T----L---F--------
	Kamptonema	WP_007355519	E---M---AVREV-QEAVARA-		AGE--T----L---F--------
	Cyanothece sp. CCY0110	WP_008272481	E---M---AVR-V-QEAVARA-		AGE--T----L---F--------
	Cyanothece	WP_009546784	E---M---AVR-V-QEA-ARA-		AGE--T----L---F--------
	Pseudanabaena	WP_009626838	E---M----VREY-QEA-RRA-		AGE--TVL-CM---F--------
	Lyngbya sp. PCC 8106	WP_009787238	E---M-I-AVHT---EAVARA-		AGE--T----L---F--------
	Synechococcus	WP_012305987	E---M---AMRDV-Q-AVARA-		AGE--T----L---F--------
	Cyanothece sp. PCC 7424	WP_012597993	E---M---AVRNV--EA-ARA-		AGE--T----L---F--------
	Cyanothece sp. PCC 7822	WP_013321798	E---M---AVRTV--EA-ARA-		AGE--T----L---F--------
	Arthrospira platensis	WP_014276531	E---M---AVREV-Q-A-ARA-		AGE--T----L---F--------
	Nostoc sp. PCC 7107	WP_015111181	E---M---AVHSV-QEAVARA-		AGE--T----L---F--------
	Nostoc sp. PCC 7524	WP_015138839	E---M---AVHSV-QEAVARA-		AGE--T----L---F--------
	Chamaesiphon minutus	WP_015160492	Q---M---AV-SV--EAVARA-		AGE--T----L---F--------
	Oscillatoria nigro-viridis	WP_015178608	E---M---AVREV--EAVARA-		AGE--T----L---F--------
	Stanieria cyanosphaera	WP_015195309	E---M---AVRAV-QEA-ARA-		AGE--T----L---F--------
	Calothrix parietina	WP_015199024	E---M---AV-TT--EAVRRA-		AGE--T----L---F--------
	Dactylococcopsis salina	WP_015228871	E---M---AM-ST-QEAVARA-		AGE--T----L---F--------
	Geminocystis herdmanii	WP_017293986	E---M-L-AVREV-QRA-ERA-		AGE--T----L---F--------
	Spirulina subsalsa	WP_017306355	E---M---AVR-V-QEAVARA-		AGE--T----L---F--------
	Calothrix sp. PCC 7103	WP_019489774	E---M---AV-SV--EAVRRA-		SGE--T----L---F--------
	Pleurocapsa sp. PCC 7319	WP_019509314	E---M---AVNT---EAVKRA-		AGE--T----L---Y--------
	Tychonema bourrellyi	WP_096830499	E---M---AVREV--EAVARA-		AGE--TV---L---F--------
	Pyropia fucicola	AIA21502	E---M---AVRQA--QAVQRA-		-GD--T----L---F--------
	Cyanidiaceae sp. MX-AZ01	AIA61192	E---M---AVHEV-LQAVERAK		SAK--T----L---F--------
	Bangia fuscopurpurea	AKE98868	E---M---AVRQA-EQAVQRA-		QGQ--T----L---F--------

Group	Species	Accession	Sequence	Sequence
Rhodophyta	*Pyropia endiviifolia*	ALL97353	E---M---AVRQV-SQAVQRA-	QGE--T----L---F--------
	Cyanidium caldarium	NP_045197	E---M--VAVH-T--EA-LKA-	-GD--T----L---F--------
	Porphyra purpurea	NP_053877	E---M---AVRQV-E-AVERA-	QGQ--T----L---F--------
	Cyanidioschyzon merolae str.	NP_849059	E---M---AVHEV--EAVERA-	SAK--T----L---F--------
	Porphyridium purpureum	YP_008965799	E---M---AVNSA-Q-A-ERA-	TGR--T----L---F--------
	Pyropia perforata	YP_009027551	E---M---AVRQA-IQAVQRA-	QGE--T----L---F--------
	Wildemania schizophylla	YP_009237363	E---M---AVRQA-Q-A-LRA-	QGQ--T----L---F--------
	Pyropia pulchra	YP_009244786	E---M---AVRQA-SQAVQRA-	QGD--T----L---F--------
	Porphyra umbilicalis	YP_009413280	E---M---AVRQV-E-AVKRA-	QGQ--T----L---F--------
	Pyropia yezoensis	YP_536948	E---M---AVRQA--QAVQRA-	QGD--T----L---F--------
	Boldia erythrosiphon	YP_009369833	E---M---A-RSVTDEA-KRA-	NGE--T----L---F--------
	Nemalion sp. H.1444	SCW22997	E---M---A-R-VSLDA-ERA-	NGE--T----L---F--------
	Chondrus crispus	YP_007627411	E---M---A-RNVTQQA-KRA-	SGE--T----L---F--------
	Grateloupia taiwanensis	YP_008144878	E---M---AVREV-Q-S-DRA-	SGE--T----L---F--------
	Choreocolax polysiphoniae	YP_009122071	E---M---AVRDV-Q-A-FRA-	SGQ--T----L---F--------
	Tolypiocladia glomerulata	YP_009399936	E---M---AVR-I-Q-A-DRA-	SGD--T----L---F--------
	Corynoplastis japonica	ARO90960	E---M---A-HTI-REAVTRA-	LGE--T----L---F--------
	Bangiopsis subsimplex	YP_009296897	EI--M---AVREATERA-QRA-	NGE--T----L---F--------
Streptophyta	*Medicago truncatula*	ABD32705	---M---KVRQV--EA-GRA-	RGE--T---CE---F--------
	Oryza sativa	CAH65949	---M---KVREV--EA-ERA-	RGE--T-V-CE---F--------
	Brassica napus	CDY37428	---M---KVREV--EAVSRA-	RGE--T-V-CE---F--------
	Oryza sativa Indica Group	EAY92846	---M---KVREV--EA-ERA-	RGE--T-V-CE---F--------
	Citrus sinensis	KDO86616	H---M---KVREV--EA-ERA-	RGE--T-V-CE---F--------
	Glycine soja	KHN11375	---M---KVREV--EA-ERA-	RGE--T-V-CE---F--------
	Glycine max	KRG97241	---M---KVREV--EA-ERA-	RGE--T-V-CE---F--------
	Cajanus cajan	KYP55874	---M---KVREV--EA-QRA-	RGE--T-V-CE---F--------
	Zea mays	NP_001140759	---M---KVREV--EA-ERA-	RGE--T-V-CE---F--------
	Marchantia polymorpha subsp. r.	OAE24279	---M---KVREV--EA-ERA-	RGE--T---CE---F--------
	Dichanthelium oligosanthes	OEL15282	---M---KVREV--EA-ERA-	RGE--T-V-CE---F--------
	Panicum hallii	PAN44204	---M---KVREV--EA-ERA-	RGE--T-V-CE---F--------
	Vitis vinifera	XP_002267676	H---M---KVREV--EA-QRA-	RGE--T-V-CE---F--------
	Citrus clementina	XP_006444729	H---M---KVREV--EA-ERA-	RGE--T-V-CE---F--------
	Phaseolus vulgaris	XP_007139015	---M---KVREV--EA-ERA-	RGE--T-V-CE---F--------
	Elaeis guineensis	XP_010940832	---M---KVRAV--EA-ERA-	RGE--TV--CE---F--------
	Vigna radiata var. radiata	XP_014499099	---M---KVREV--EA-ERA-	RGE--T-V-CE---F--------
	Oryza sativa Japonica Group	XP_015636508	---M---KVREV--EA-ERA-	RGE--T-V-CE---F--------
	Vigna angularis	XP_017409763	---M---KVREV--EA-ERA-	RGE--T-V-CE---F--------
	Arachis duranensis	XP_020990484	---M---KVREV--EA-ERA-	RGE--T-V-CE---F--------
	Manihot esculenta	XP_021613440	---M---KVREV--EA-ERA-	RGE--T-V-CE---F--------
Other Prokarya	*Mesorhizobium sp. SEMIA 3007*	ODA93765	Q-----P-AV-NMT-EAVEKC-	RGE-------K---HM--HVN--
	Pararhizobium antarcticum	OJF90867	Q-----S-AV-NMT-DAVEKC-	RGE-------K---HM--HVN--
	Kiloniella laminariae	WP_020592143	R-----Y-AV-AV--WAAERA-	RNL--VM--YV---AG---TS-
	Ochrobactrum rhizosphaerae	WP_024897897	R-----Y-AVHAV--WAVERA-	RNL--TI--YV---AG---TS-
	Ochrobactrum sp. A44	WP_095444415	R-----Y-AVHAV--WAVERA-	RNL--TI--YV---AG---TS-
	Mesorhizobium sp. BSA136	WP_097576203	Q-----P-AV-NMT-EAVEKC-	RGE-------K---HM--HVN--
	Desulfuromonas soudanensis	ALC17519	R---M---AV-QAVHQAVQKA-	NGE--TY---R---Y----IS--
	Burkholderia sp. TNe-862	WP_092006487	----M---KV-EA-RHAMDR--	NGG--QF--CE---Y----M---
	Methylothermaceae bacteria B.	KXJ40283	R---M---KV-EATQHALQQ--	AGT--Q---CV---F----M---
	Enterovibrio calviensis	WP_028022070	I-----ADAM-EV-S-A-EKA-	SGD--S----K---HQ---R---
	Dethiosulfovibrio peptidovor.	WP_005659336	M--------V-ETS-EVVDY--	SGN--V-L-CK---IK--FVG--
	Aminobacterium colombiense	WP_013048970	------FAV-EA-QEL-ERA-	RG---A-L--K---IK--FVG--
	Dethiosulfovibrio salsuginis	WP_085544977	I------FAV-EA--EI-QS--	SGG--V-L-CK---IK--FVG--
	Planctomycetes bacterium RBG.	OHB84068	Q-------AV-EA-QTAVE-C-	GGT--V-L-LL---RT--RR--
	Theionarchaea archaeon DG-70	KYK38214	------MAV-EA---AVLRA-	RGE--S---CK---HK---RF--
	Thermococcus sp. 4557	WP_014012842	---Q---AV-EV--EA-ERA-	RGE--T----K---F---FEG--
	Thermococcus radiotolerans	WP_088867362	---Q---AV-EV--EA-ERA-	RGE--T----K---F---FEG--
	Archaeoglobales archaeon ex.	OYT33189	---Q---AV-EV--EA-ERA-	RGE--T----K---F---FEG--
	Chloroflexi bacterium RBG_16.	OGO20505	R---M---QV-NATQ-ALEHV-	-E--F-L--L---Y----MG--
	Nitrolancea hollandica	WP_008475609	Q---Q---AMHEAT--ALDHC-	SGN--FFL--M---F----M---
	Anaerolinea thermophila	WP_013559836	R---M--MAMRQA-EEM-EA--	NGA--M-M--M---F----MG--
	Thermomicrobium roseum	WP_015922675	R---Q---AV-EAT-RALEHC-	SGN--YFL--L---Y----M---
	Anaerolinea thermolimosa	WP_062189809	R-N-M-LMEVRQV-SEM-EQ--	QGA--MML------F----MG--
	Clostridiales bacterium DRI.	WP_034420390	I------EV--T--EAVEKL-	-GE--M----K---HM--YTG--
	Oceanobacillus caeni	WP_047184921	R-------AC-QV--EA-EKA-	-GE--T----V---RES-TT--
	Acidothermus cellulolyticus	WP_011719882	------AAVFDV-RRS-A-C-	TGG--V----L---QG---R---

Figure S31. A partial sequence alignment of the Pyruvate dehydrogenase E1 component, alpha subunit protein showing a one amino acid insertion that is specific for members of *Plasmodium* and *Piliocolobus tephrosceles*. The presence of this CSI within the latter species is likely due to contamination. CSI region is not conserved in *Piroplasmida*.

Group	Species	Accession	103		162
**_Plasmodium_ (20/20)**	*Plasmodium berghei str. ANKA*	XP_677676	YYRGATCAIIVFDISNSNTLDRAKTWVNQLKI		SGNYIIILVANKIDKNKFQVD
	Plasmodium sp. DRC-Itaito	SOV20671	---------V----------------------		-S-------------------
	Plasmodium sp. gorilla clade G	SOV10564	---------V----------------------		-S-------------------
	Plasmodium chabaudi cha.	SCL97483	--------------------------------		---------------------
	Plasmodium coatneyi	XP_019913303	---------V-------SS-------------		-S-------------------
	Plasmodium cynomolgi str. B	XP_004225388	---------V-------SS-------------		-S-------------------
	Plasmodium falciparum 3D7	XP_001349618	---------V----------------------		-S-------------------
	Plasmodium falciparum Dd2	KOB87895	---------V----------------------		-S-------------------
	Plasmodium falciparum FCH/4	ETW27535	---------V----------------------		-S-------------------
	Plasmodium falciparum IGH-CR14	KNG77333	---------V----------------------		-S-------------------
	Plasmodium fragile	XP_012336879	---------V-------SS-------------		-S-------------------
	Plasmodium gaboni	XP_018643768	---------V----------------------		-S-------------------
	Plasmodium gallinaceum	CRG94314	---------V--------S-------------		-S-------------------
	Plasmodium gonderi	GAW79497	---------V--------S-------------		-S-------------------
	Plasmodium inui San Ant. 1	XP_008816607	---------V-------SS-------------		-S-------------------
	Plasmodium knowlesi str. H	XP_002257925	---------V-------SS-------------		-S-------------------
	Plasmodium malariae	SBS82394	---------V--------S-------------		-S-------------------
	Plasmodium ovale curt.	SBS80669	---------V--------S-------------		-S-------------------
	Plasmodium ovale wal.	SBT31695	---------V--------S-------------		-S-------------------
	Plasmodium reichenowi	XP_012760989	---------V----------------------		-S-------------------
	Plasmodium relictum	CRG98739	---------V--------S-------------		-S-------------------
	Plasmodium vinckei pet.	EUD69995	--------------------------------		---------------------
	Plasmodium vinckei vin.	XP_008626522	--------------------------------		---------------------
	Plasmodium vivax Sal-1	XP_001612933	---------V-------SS-------------		-S-------------------
	Plasmodium yoelii yoe. 17XNL	XP_724585	----------------L---------------		---------------------
**_Piroplasmida_ & Other Apicomplexa (0/8)**	*Babesia bigemina*	XP_012769022	-----A---VIL-V-S-QS-QH-AS-----RV	AN	-NET-VV-----V-
	Babesia bovis T2Bo	XP_001611807	-----A---VIL-V-S-SS-QH-IS--D-IRM	TN	-S-TLT--------
	Babesia ovata	GBE58911	-----A---VIL-V-S-QS-QH-TS-----RV	AN	-NET-VV-----V-
	Babesia microti strain RI	XP_012649196	-----V---V-M-CTCQKSFE---N--K---Q	CP	HS-P-VV-----V-
	Theileria annulata	XP_953226	-----A---VIL-V-SPP--QH-AF----IRV	AN	NNET--V-----T-
	Theileria parva strain Muguga	XP_764210	-----A---VIL---SPP--QH-AF----IRV	AN	NNET--V--G--V-
	Theileria orientalis	PVC52558	---R-A---VIL---SPP--QH-AF----IRV	AN	NNET--V-------
	Theileria equi strain WA	XP_004830994	---K-A---VIL-VTLPQ--QH-AF----IRV	AN	NNET--V-------
	Besnoitia besnoiti	PFH37569	-----AA--V-Y-Q--MASF---QV--Q--QL	SG	NS-IV-A-A---M-LPHK---
	Cystoisospora suis	PHJ19873	-----AA--V-Y-Q--VASF---QV--Q--QL	SG	NP-IV-A-A---M-LPHR---
	Eimeria brunetti	CDJ46129	-----AA-VV-Y-QT-GPSFE--QM--Q--QL	SG	NPHIV-A-A---A-I-NK---
	Eimeria maxima	XP_013336014	-----AA----Y-QT-GPSYE---M--Q--QL	SY	NP-I--A-A---A-I-NK-I-
	Hammondia hammondi	XP_008889443	-----AA--V-Y-Q--MASF---QV--Q--QL	SG	NS-IV-A-A---M-LPHK---
	Neospora caninum Liv.	XP_003885691	-----AA--V-Y-Q--MASF---QV--Q--QL	SG	NS-IV-A-A---M-LPHK---
	Toxoplasma gondii ME49	XP_018634789	-----AA--V-Y-Q--MASF---QV--Q--QL	SG	NS-IV-A-A---M-LPHK---
**Other Eukarya**	*Vitrella brassicaformis CCMP.*	CEM04485	-----SA--V-Y-Q--RQSFE--Q---K--QL	SG	NP-IV-A-A---M-LPERATS
	Sphaerobolus stellatus SS14	KIJ54191	---N-N--VV-Y---Q-AS--K-R--IRE-QR	QA	DPSIV-L-CG--A-LEARRQV
	Gloeophyllum trabeum ATCC 11.	XP_007860601	---N-N--VV-Y--TQ-TS--K----IRE-QR	QA	DP-IV-A-CG--T-LAARRQV
	Salmo salar	ACI32882	-----QA--V-Y--T-TD-FT---N--KE-QR	QA	-P-IV-A-AG--A-LANKRAV
	Osmerus mordax	ACO09947	-----QA--V-Y--T-TD-FT---N--KE-QR	QA	-P-IV-A-AG--A-IANKRAI
	Litopenaeus vannamei	AFK08607	-----QA--V-Y--T-QD-FG------KE-QR	QA	-P-IV-A-AG--A-LANKRMV
	Macrobrachium rosenbergii	AJC97113	-----QA--V-Y--T-QD-FG------KE-QR	QA	-P-IV-A-AG--A-LATKRMV
	Tetraodon nigroviridis	CAF91320	-----QA--V-Y--T--D-FA---N--KE-QR	QA	-P-IV-A-AG--A-ITNKRAV
	Dicentrarchus labrax	CBN81473	-----QA--V-Y--T-TD-FA---N--KE-QR	QA	-P-IV-A-AG--A-IANKRAV
	Trichuris trichiura	CDW51862	-----QA--V-Y-VT-QESFTK--N---E-QR	QA	LP-IV-A-AG--A-LANKRMV
	Bos taurus	DAA33577	-----QA--V-Y--T-EESFA---N--KE-QR	QA	-P-IV-A-SG--A-LANKRAV
	Bos mutus	ELR48410	-----QA--V-Y--T-EESFA---N--KE-QR	QA	-P-IV-A-SG--A-LANKRAV
	Capitella teleta	ELU02811	-----QA--V-Y--T-QD-FG------RE-QR	QA	-P-IV-A-AG--A-LANKRMV
	Cricetulus griseus	ERE90048	-----QA--V-Y--T-EESFA---N--KE-QR	QA	-P-IV-A-SG--A-LANKRAV
	Larimichthys crocea	KKF24614	-----QA--V-Y--T-TD-FA---N--KE-QR	QA	-P-IV-A-AG--A-IANKRAV
	Sarcoptes scabiei	KPM02352	-----QA--V-Y--T-QD-FN------KE-QK	QA	KP-IV-A-AG--S-LEDKRTV
	Scleropages formosus	KPP56982	-----QA--V-Y--T-ADSFA---N--KE-QR	QG	-PYIV-T-AG--A-LASKREV
	Oryctes borbonicus	KRT83595	-----QA--V-Y--T-QD-FG------KE-QR	QA	-PTIV-A-AG--Q-LANKRMV
	Cynoglossus semilaevis	XP_008312034	-----QA-VV-Y--T-PD-FV---N--KE-QR	QA	-P-IV-A-AG--A-IANKRAV
	Calypte anna	XP_008502558	-----QA--V-Y--T-EESFA---N--KE-QR	QA	-P-IV-A-AG--A-LANKRAV
	Merops nubicus	XP_008937094	-----QA--V-Y--T-TD-FV---N--KE-QR	QA	-P-IV-A-AG--A-LATKRAV
	Cuculus canorus	XP_009568635	-----QA--V-Y--T-EESFA---N--KE-QR	QA	-P-IV-A-AG--A-LANKRAV
	Monopterus albus	XP_020461023	-----QA--V-Y--T-AD-FA---N--KE-QR	QA	NP-IV-A-AG--A-IANKRAV
	Labrus bergylta	XP_020507769	-----QA--V-Y--T-TD-FT---N--KE-QR	QA	-P-IV-A-AG--A-IANKRAV
	Pogona vitticeps	XP_020641246	-----QA--V-Y--T-MD-FV---N--KE-QR	QA	-S-IV-A-AG--A-LGSKRAV

Figure S32. A partial sequence alignment the Ras-related protein Rab-5A showing a two amino acid deletion that is specific for members of *Plasmodium.*

Group	Organism	Accession	97		142
***Plasmodium* (20/20)**	*Plasmodium berghei str. ANKA*	XP_677367	APVIDVTRVGYFKVLGNGHLE	H	NQPIVVKARYFSSMAEKKIKAVGG
	Plasmodium sp. DRC-Itaito	SOV21689.1	--------K---------K-K	-	-------------V----------
	Plasmodium sp. Gor. Cla. G	SOV12594	--------K---------K-K	-	-------------V----------
	Plasmodium fragile	XP_012335263	--------K-F-------K-K	-	-------------V----------
	Plasmodium coatneyi	XP_019915869	--------K-F-------K-K	-	-------------V----------
	Plasmodium chabaudi ada.	SCM22365	--------I---------I-K	S	D------------I-------A--
	Plasmodium chabaudi cha.	SCM23629	--------I---------I-K	S	D------------I----------
	Plasmodium cynomolgi str. B	XP_004223319	--------K-F-------K-K	-	-------------V----------
	Plasmodium falciparum 3D7	XP_966168	--------K---------K-K	-	-------------V----------
	Plasmodium falciparum FCH/4	ETW30434	--------K---------K-K	-	-------------V----------
	Plasmodium gaboni	XP_018642814	--------K---------K-K	-	-------------V----------
	Plasmodium gallinaceum	CRG93761	--------K---------K-K	-	-------------L----------
	Plasmodium gonderi	GAW81844	--------K-F-------K-K	-	G------------V----------
	Plasmodium inui San Ant. 1	XP_008814728	--------K-F-------K-K	-	G------------V----------
	Plasmodium knowlesi str. H	XP_002261493	--------K-F-------K-K	-	-------------V----------
	Plasmodium malariae	SBS87206	-------K---------A-R	C	G-----------KR----------
	Plasmodium ovale curt.	SBS85368	--------K---------K-K	S	K------------L----------
	Plasmodium ovale wal.	SBT38421	--------K---------K-K	S	K------------S----------
	Plasmodium reichenowi	XP_012761996	--------K---------K-K	-	-------------V----------
	Plasmodium relictum	CRH00953	--------R---------K-K	-	-------------L----------
	Plasmodium vinckei pet.	EUD73289	--------I---------N-K	T	D------------I-------A--
	Plasmodium vinckei vin.	XP_008624412	--------I---------N-K	S	D------------I----------
	Plasmodium vivax Sal-1	XP_001616196	--------K-F-------K-K	-	A------------V----------
	Plasmodium yoelii yoe. 17XNL	XP_731377	------------------N--	-	-------------I----------
***Piroplasmida* & Other Apicomplexa (0/8)**	*Theileria equi*	510900241	---V--IQK-F-----T-S-P		KR--I----L--KI------EA--
	Theileria parva str. Mug.	71030482	---L--MQS-------T-NMP		QR-L-----L--KT--R---EA--
	Theileria annulata	84995360	---L--MQS-------T-NMP		PR-L-----L--KT--R---EA--
	Theileria orientalis str. Shi.	697890181	---L--MKL-C-----T-KMP		PK-L-----L--KT------EA--
	Babesia ovata	GBE58936	---L-LVQK-------T-KMP		QK-L-----F--KL--R----A--
	Babesia bovis T2Bo	156088757	---L--VQK---------TMP		KK-LI-------KL------EA--
	Babesia sp. Xin.	1181628049	---L--VQR-------T-N-P		KK-LI----F--KL------EA--
	Babesia bigemina	833490096	---L-LVQR-------T-KMP		QK-L-----F--KL--R----A--
	Babesia microti str. RI	XP_012649225	---V---KA-------T---P		E--LI-------KLS-E---KA--
	Besnoitia besnoiti	1261480578	-A-----KS-------K-D-P		KV-VI----F--RI------EA--
	Toxoplasma gondii ME49	237829995	-A-----KS-------K-D-P		KI-VI----F--KI-------A--
	Cystoisospora suis	1268238688	-------KS-F-----K-EMP		KV-VI----F--RL------EA--
	Cyclospora cayetanensis	1249152249	-F-V-C--A-------K-E-P		KL-LI-R--F--KL------EA--
	Eimeria acervulina	914999367	-F-L-C--A-------K-S-P		KL-LI-R--F--KL------EA--
	Eimeria brunetti	557241833	-F-L-C-QA-------K-T-P		KL-LI-R--F--KL------EA--
	Eimeria maxima	915123037	-F-L-C-KA-------K-Q-P		KL-LI-R--F--KL------EA--
	Eimeria necatrix	921125444	-Y-V-C-QA-------K-A-P		KL-LI-R--F--KL------EA--
	Eimeria tenella	916415238	-Y-V-C-QA-------K-A-P		KL-LI-R--F--KL------EA--
	Cryptosporidium andersoni	1098428728	-----C-QA-I-----G---P		-V-V--R--S--KI------EA--
	Cryptosporidium parvum Iowa II	66359520	--I--C-KS-I-----G-N-P		-L-V--R--H--KK------EA--
	Cryptosporidium ubiquitum	1098426241	--I--C--S-I-----G-N-P		AV-V--R--H--KK------EA--
	Gregarina niphandrodes	749162345	--L----A--R------PAL		TRAF--R--E--KL-KE--E-A--
**Other Eukarya**	*Acytostelium subglobosum LB1*	XP_012747878	---V---QR-F-----H-L-P		S---I-------KV----------
	Agrilus planipennis	XP_018333064	-----IV-A--Y----K-R-P		K--VI---KF--KS--D-------
	Ailuropoda melanoleuca	AEA39520	--I---V-S--Y----K-K-P		K--VI---KF--RR--E---G---
	Albugo candida	CCI49799	--I----KA-F-----K-R-P		KI-VI---KF--QE----------
	Alligator mississippiensis	XP_006264922	------V-S--Y----K-K-P		K--VI---KF--RK--E---E---
	Alligator sinensis	XP_014372701	------V-S--Y----K-K-P		K--VI---KF--RK--E---E---
	Amazona aestiva	KQK76149	------V-S--Y----K-K-P		K--VI---KF--RR--E---E---
	Anas platyrhynchos	XP_005024779	------V-S--Y----K-K-P		K--VI---KF--RR--E---E---
	Anolis carolinensis	XP_003217239	------V-A--Y----K-K-P		K--VI---KF--RR--E---G---
	Canis lupus familiaris	XP_005633358	--I---V-S--Y----K-K-P		K--VI---KF--RR--E---G---
	Cariama cristata	KFP59275	------V-S--Y----K-K-P		K--VI---KF--RR--E---E---
	Ceratotherium simum simum	XP_014641129	--I---V-S--Y----K-K-P		K--VI---KF--RI--E---G---
	Tetrahymena thermophila SB210	XP_001016809	------KA-F-----K-R-P		---V----K---KT--RR-V----
	Tinamus guttatus	KGL84910	------V-S--Y----K-K-P		K--VI---KF--RR--E---E---
	Phaeodactylum tricornutum CC.	XP_002177467	-----LVSK----L----QIN		V-VI---K---KL------EA--

Figure S33. A partial sequence alignment of the Ribosomal protein L27a showing a one amino acid insertion that is specific for members of *Plasmodium* and some *Streptophyta* (not shown).

Group	Species	Accession	121		178
Plasmodium Isoform 1 (20/20)	*Plasmodium berghei str. ANKA*	XP_673190	KCLLLAGPSGSGKTAIAIAISKEI	S	EEAIPFCIFNASQVYSCEVKKTEILTQYIRKSI
	Plasmodium sp. DRC-Itaito	SOV22178	------------------------	-	D-S------------------------------
	Plasmodium sp. gorilla clade G	SOV13517	------------------------	-	D-S------------------------------
	Plasmodium chabaudi ada.	SCM24986	------------------------	-	--S------------------------------
	Plasmodium chabaudi cha.	XP_016654928	------------------------	-	--S------------------------------
	Plasmodium coatneyi	XP_019916551	------------------------	-	-DS------------------------------
	Plasmodium cynomolgi str. B	XP_004224904	------------------------	-	-DS------------------------------
	Plasmodium falciparum 3D7	AFP95340	------------------------	-	D-S------------------------------
	Plasmodium falciparum 7G8	EUR72859	------------------------	-	D-S------------------------------
	Plasmodium falciparum CAMP.	ETW61891	------------------------	-	D-S------------------------------
	Plasmodium falciparum FCH/4	ETW31233	------------------------	-	D-S------------------------------
	Plasmodium falciparum UGT5.1	EWC77212	------------------------	-	D-S------------------------------
	Plasmodium fragile	XP_012336951	------------------------	-	-DS------------------------------
	Plasmodium gaboni	XP_018642193	------------------------	-	D-S------------------------------
	Plasmodium gallinaceum	CRG97688	------------------------	-	--S------------------------------
	Plasmodium gonderi	GAW83501	------------------------	-	-DS------------------------------
	Plasmodium inui San Ant. 1	XP_008814508	------------------------	-	-DS------------------------------
	Plasmodium knowlesi str. H	XP_002262193	------------------------	-	-DS------------------------------
	Plasmodium malariae	SBS90616	------------------------	-	-DS------------------------------
	Plasmodium ovale curt.	SBS88341	------------------------	-	--S------------------------------
	Plasmodium ovale wal.	SBT48315	------------------------	-	--S------------------------------
	Plasmodium reichenowi	XP_012762483	------------------------	-	D-S------------------------------
	Plasmodium relictum	CRH02548	------------------------	-	-DS------------------------------
	Plasmodium vinckei pet.	EUD74350	------------------------	-	--S------------------------------
	Plasmodium vinckei vin.	XP_008622953	------------------------	-	--S------------------------------
	Plasmodium vivax Ind. VII	KMZ78042	------------------------	-	-DS------------------------------
	Plasmodium vivax North Kor.	KMZ96872	------------------------	-	-DS------------------------------
	Plasmodium vivax Sal-1	XP_001617220	------------------------	-	-DS------------------------------
	Plasmodium yoelii 17X	ETB61584	------------------------	-	---------------------------------
	Plasmodium yoelii yoe. 17XNL	XP_729450	------------------------	-	---------------------------------
Plasmodium Isoform 2 (0/20)	*Plasmodium berghei str. ANKA*	XP_675861	--I----------S-L--G--R--		NKKM--VFLSG-E---N-I----VILEAF----
	Plasmodium sp. DRC-Itaito	SOV23144	--I----------S-L--G--R--		NRKM--VFLSG-E---N-I----VILEAF----
	Plasmodium sp. gorilla clade G	SOV15340	--I----------S-L--G--R--		NRKM--VFLSG-E---N-I----VILEAF----
	Plasmodium chabaudi cha.	CAH83857	--I----------S-L--G--R--		NKKM--VFLSG-E---N-I----VILEAF----
	Plasmodium coatneyi	XP_019915132	--I----------S-L--G--R--		NKKM--VFLSG-E---N-I----VILEAF----
	Plasmodium cynomolgi str. B	XP_004222291	--I----------S-L--G--R--		NKKM--VYLSG-E---N-I----VILEAF----
	Plasmodium falciparum 3D7	XP_001347747	--I----------S-L--G--R--		NRKM--VFLSG-E---N-I----VILEAF----
	Plasmodium falciparum Dd2	KOB85310	--I----------S-L--G--R--		NRKM--VFLSG-E---N-I----VILEAF----
	Plasmodium falciparum IGH-CR14	KNG76139	--I----------S-L--G--R--		NRKM--VFLSG-E---N-I----VILEAF----
	Plasmodium falciparum Tan.	ETW36102	--I----------S-L--G--R--		NRKM--VFLSG-E---N-I----VILEAF----
	Plasmodium fragile	XP_012336553	--I----------S-L--G-CR--		NKKM--VYLSG-E---N-I----VILEAF----
	Plasmodium gaboni	XP_018640980	--I----------S-L--G--R--		NRKM--VFLSG-E---N-I----VILEAF----
	Plasmodium gallinaceum	CRG96118	--I----------S-L--G--R--		NKKM--VFLSG-E---N-I----VILEAF----
	Plasmodium gonderi	GAW80838	--I----------S-L--G--R--		NKKM--VFLSGAE---N-I----VILEAF----
	Plasmodium inui San Ant. 1	XP_008817838	--I----------S-L--G--R--		NKKM--VFLSG-E---N-I----VILEAF----
	Plasmodium knowlesi str. H	XP_002259060	--I----------S-L--G--R--		NKKM--VFLSG-E---N-I----VILEAF----
	Plasmodium malariae	SBS85123	--I----------S-L--G--R--		NKKM--VFLSG-E---N-I----VILEAF----
	Plasmodium ovale curt.	SBS83316	--I----------S-L--G--R--		NKKM--VFLSG-E---N-I----VILEAF----
	Plasmodium ovale wal.	SBT34864	--I----------S-L--G--R--		NKKM--VFLSG-E---N-I----VILEAF----
	Plasmodium reichenowi	XP_012763470	--I----------S-L--G--R--		NRKM--VFLSG-E---N-I----VILEAF----
	Plasmodium relictum	CRG99936	--I----------S-L--G--R--		NKKM--VFLSG-E---N-I----VILEAF----
	Plasmodium vinckei vin.	XP_008624617	--I----------S-L--G--R--		NKKM--VFLSG-E---N-I----VILEAF----
	Plasmodium vivax Sal-1	XP_001615188	--I----------S-L--G--R--		NKKM--VFLSG-E---N-I----VILEAF----
	Plasmodium yoelii 17X	ETB57938	--I----------S-L--G--R--		NKKM--VFLSG-E---N-I----VILEAF----
	Plasmodium yoelii yoe. 17XNL	XP_731373	--I----------S-L--G--R--		NKKM--VFLSG-E---N-I----VILEAF----
Piroplasmida & Other Apicomplexa (0/8)	*Babesia bigemina*	XP_012768949	RA-------------L-M--A--L		NTSA--T-LSSTE-F-S------V-NEAF----
	Babesia ovata	GBE58792	RA--F----------L-M--A--L		NT-A--T-LSSTE-F-T------V-NEAF----
	Babesia bovis T2Bo	XP_001611896	RA-------------L-M--AR--		STSA--ILLSSTE-F-S--------NEAF----
	Babesia microti str. RI	XP_021337180	-AV-------T----L-M--C--L		GDSV--TPISSTE-F----------NEAF----
	Babesia sp. Xin.	ORM40192	RA-------------L-M--AR-L		NTSA--TVLSSTE-F-S------V-NEAF----
	Theileria annulata	XP_953307	-A-------------L-MG-AR-L		NTSA--T-LSSTE-F-T--------NEAV----
	Theileria equi	XP_004831192	-A-------------L-M--AR-L		SAS---T-LSSTE-F-S--------NEAF----
	Theileria orientalis str. Shi.	XP_009692162	-A-------------L-MG-AR-L		STSA--T-LSSTE-F-S--------NEAF----
	Theileria parva str. Mug.	XP_764299	-A-------------L-MG-AR-L		STSA--T-LSSTE-F-T--------NEAV----
	Besnoitia besnoiti	PFH35429	-A------P-T------M--AQ-L		GPKV---PMV--E---A--------MENF-RA-
	Cyclospora cayetanensis	XP_022590922	-A--I---P-T------M--AQ-L		GAKV---PMV--E---S------V-MENF-RA-
	Cystoisospora suis	PHJ20088	-A------P-T------M--AQ-L		GPKV---PMV--E---A--------MENF-RA-
	Eimeria mitis	XP_013349420	-A------P-T------M--AQ-L		GSKV---PMV--E---S------V-MENF-RA-
	Eimeria necatrix	XP_013439206	-A--I---P-T------M--AQ-L		GSKV---PMV--E---S------V-MENF-RA-
	Eimeria tenella	XP_013233010	-A--I---P-T------M--AQ-L		GSKV---PMV--E---S------V-MENF-RA-
	Hammondia hammondi	XP_008886115	-A------P-T------M--AQ-L		GPKV---PMV--E---T--------MENF-RA-
	Neospora caninum Liv.	CEL64762	-A------P-T------M--AQ-L		GPKV---PMV--E---T--------MENF-RA-
Other Apicom-plexa	*Toxoplasma gondii ME49*	XP_018638354	-A------P-T------M--AQ-L		GPKV---PMV--E---T--------MENF-RA-
	Cryptosporidium andersoni	OII75775	-AI-F---P-T------L-MAH-L		GPKV---PMV--E---A------V-MENF-R--
	Cryptosporidium hominis TU502	XP_668483	-AV-----P-T------Q--AH-L		GPKV---PMV--E---A--------MENF-RA-
	Cryptosporidium muris RN66	XP_002141376	-AV-F---P-T------L-MAH-L		GPKV---PMV--E---A------V-MENF-R--
	Cryptosporidium ubiquitum	OII74083	-AV-----P-T------Q--AH-L		GPKV---PMV--E---A--------MENF-RA-
	Gregarina niphandrodes	XP_011131659	-AA-----P-T------F-L-RDL		GSRV---ALS-AE---Q---------ECF-RA-

Group	Species	Accession	Sequence	Sequence
**Other Eukarya**	*Phaeodactylum tricornutum CC.*	XP_002179360	RA---V--P-T----L-L-MA--L	GP-V---PMV------K------M--TSF--A-
	Cyanidioschyzon merolae str.	XP_005536013	RAV-----PCT------L---R-L	GRRV---VL-GAE---S---------SHF-RA-
	Blastocystis sp. ATCC 50177.	OAO14914	RA------P-T----L-M--AHQL	GPRV---PMS--E---S------V-MENF-RA-
	Blastocystis hominis	XP_012897480	RA------P-T----L-----HQL	GPKV---PMS--E---S--------MENF-RA-
	Blastocystis sp. subtype 4	XP_014527755	RA------P-T----L-M--AQQL	GPKV---PMS--E---S------V-MENF-RA-
	Stentor coeruleus	OMJ82899	RA--F---P-T------L-VAQ-L	GPKV---PMVG-E---S--------MENF-RA-
	Dacryopinax primogenitus	EJU05942	RA------P-T----L-M---H-L	GTKV--R-LV--E---A------V-AENF-RA-
	Allomyces macrogynus ATCC 38.	KNE58263	RA------P--------L--AQ-L	GPKV---PMVG-E---S-I----V-MENF-RA-
	Conidiobolus coronatus NRRL.	KXN71093	RA-----S--T------Y-----L	GHH----PMV--E-F-T------V-MENF-RA-
	Calocera cornea HHB12733	KZT51694	------P-T----L-L---H-L	GTKV--R-LV--E---A------V-AENF-RA-
	Amphiamblys sp. WSBS2006	OIR58565	-AV-------T----L-LGMAR-L	G-R----VLAG-E---A--------VENM--A-
	Catenaria anguillulae PL171	ORZ41559	RA------P--------L---Q-L	GPKV---PMVG-E---S-I----V-MENF-RA-
	Haemonchus contortus	CDJ83203	RA------P-T------L--AQ-L	GDKV---PIV--E---S--------MENF-RA-
	Brugia malayi	CDQ03254	RAI-F---P-T------L-VAQ-L	GDKM---PMVG-E---S------V-MENF-R--
	Parasteatoda tepidariorum	XP_015911433	RA--M---P-T------L--AQ-L	GSKV---PMVG-E---S-I----V-MENF-RA-
	Drosophila rhopaloa	XP_016969530	RA------P-T------L--AQ-L	GNKV---PMVG-E-F-N-I----V-MENF-R--
	Drosophila takahashii	XP_017005178	RA------P-T------L--AQ-L	GNKV---PMVG-E-F-N-I----V-MENF-R--
	Gossypium raimondii	XP_012485134	RA------P-T----L-LG--Q-L	GSKV---PMVG-E---S------V-MENF-RA-
	Erythranthe guttata	XP_012827852	RAV-F-----T----L-LG----L	DHKV---AMV--E---S------V-MENF-RA-
	Brassica oleracea var. ol.	XP_013625320	-A------P-T----L-LG--Q-L	GSKV---PMVG-E---S------V-MENF-RA-
	Capsicum annuum	XP_016548155	-AV-----P-T----L-L---Q-L	GSKV---PMVG-E---S------V-MENF-RA-
	Gossypium hirsutum	XP_016669039	RA------P-T----L-LG--Q-L	GSKV---PMVG-E---S------V-MENF-RA-
	Gossypium arboreum	XP_017608007	RA------P-T----L-LG--Q-L	GSKV---PMVG-E---S------V-MENF-RA-

Figure S34. A partial sequence alignment of the RuvB-like helicase 1 protein showing a one amino acid insertion that is specific for members of *Plasmodium.* The CSI is not seen in the other identified homolog.

Group	Species	Accession	96		144
Plasmodium **Isoform 1 (20/20)**	*Plasmodium berghei str. ANKA*	XP_673190	GMVGQKKAREAAGIFINLIKEKNIC		KCLLLAGPSGSGKTAIAIAISKEI
	Plasmodium sp. Gor. Cla. G2	SOV13517	-------------------------		------------------------
	Plasmodium sp. DRC-Itaito	SOV22178	-------------------------		------------------------
	Plasmodium chabaudi ada.	SCN62985	-------------------------		------------------------
	Plasmodium chabaudi cha.	CAH89104	-------------------------		------------------------
	Plasmodium coatneyi	XP_019916551	--I----------------------		------------------------
	Plasmodium cynomolgi str. B	XP_004224904	--I----------------------		------------------------
	Plasmodium falciparum 3D7	AFP95340	-------------------------		------------------------
	Plasmodium falciparum 7G8	EUR72859	-------------------------		------------------------
	Plasmodium falciparum CAMP.	ETW61891	-------------------------		------------------------
	Plasmodium falciparum FCH/4	ETW31233	-------------------------		------------------------
	Plasmodium falciparum UGT5.1	EWC77212	-------------------------		------------------------
	Plasmodium fragile	XP_012336951	--I----------------------		------------------------
	Plasmodium gaboni	XP_018642193	-------------------------		------------------------
	Plasmodium gallinaceum	CRG97688	-------------------------		------------------------
	Plasmodium gonderi	GAW83501	--I----------------------		------------------------
	Plasmodium inui San Ant. 1	XP_008814508	--I----------------------		------------------------
	Plasmodium knowlesi str. H	XP_002262193	--I----------------------		------------------------
	Plasmodium malariae	SBS90616	--I----------------------		------------------------
	Plasmodium ovale curt.	SBS88341	-------------------------		------------------------
	Plasmodium ovale wal.	SBT48315	-------------------------		------------------------
	Plasmodium reichenowi	CDO63865	-------------------------		------------------------
	Plasmodium relictum	CRH02548	--I----------------------		------------------------
	Plasmodium vinckei pet.	EUD74350	-------------------------		------------------------
	Plasmodium vinckei vin.	XP_008622953	-------------------------		------------------------
	Plasmodium vivax Ind. VII	KMZ78042	--I----------------------		------------------------
	Plasmodium vivax North Kor.	KMZ96872	--I----------------------		------------------------
	Plasmodium vivax Sal-1	XP_001617220	--I----------------------		------------------------
	Plasmodium yoelii 17X	ETB61584	-------------------------		------------------------
	Plasmodium yoelii yoe. 17XNL	XP_729450	-------------------------		------------------------
Plasmodium **Isoform 2 (0/20)**	*Plasmodium berghei str. ANKA*	XP_675861	-L---F------LFLVD---N-KLA	G	--I----------S-L--G--R--
	Plasmodium sp. DRC-Itaito	SOV23144	-L---F-----SLFLVD---Q-KLA	G	--I----------S-L--G--R--
	Plasmodium sp. Gor. Cla. G2	SOV15340	-L---F-----SLFLVD---Q-KLA	G	--I----------S-L--G--R--
	Plasmodium chabaudi cha.	CAH83857	-L---F------LFLVD---N-KLA	G	--I----------S-L--G--R--
	Plasmodium coatneyi	XP_019915132	-LI--F-----SLFLVD---Q-KLA	G	--I----------S-L--G--R--
	Plasmodium cynomolgi str. B	XP_004222291	-L---F-----SLFLVD-----KLA	G	--I----------S-L--G--R--
	Plasmodium falciparum	ADG56772	-L---F-----SLFLVD---Q-KLA	G	--I----------S-L--G--R--
	Plasmodium falciparum 3D7	XP_001347747	-L---F-----SLFLVD---Q-KLA	G	--I----------S-L--G--R--
	Plasmodium falciparum Dd2	KOB85310	-L---F-----SLFLVD---Q-KLA	G	--I----------S-L--G--R--
	Plasmodium falciparum IGH-CR14	KNG76139	-L---F-----SLFLVD---Q-KLA	G	--I----------S-L--G--R--
	Plasmodium falciparum Tan.	ETW36102	-L---F-----SLFLVD---Q-KLA	G	--I----------S-L--G--R--
	Plasmodium fragile	XP_012336553	-L---F-----SLFLVD---Q-KLA	G	--I----------S-L--G-CR--
	Plasmodium gaboni	XP_018640980	-L---F-----SLFLVD---Q-KLA	G	--I----------S-L--G--R--
	Plasmodium gallinaceum	CRG96118	-L---Y-----SLFLVD---K-KLA	G	--I----------S-L--G--R--
	Plasmodium gonderi	GAW80838	-L---F-----SLFLVD---Q-KLA	G	--I----------S-L--G--R--
	Plasmodium inui San Ant. 1	XP_008817838	-L---F-----SLFLVD---Q-KLA	G	--I----------S-L--G--R--
	Plasmodium knowlesi str. H	XP_002259060	-LI--F-----SLFLVD---Q-KLA	G	--I----------S-L--G--R--
	Plasmodium malariae	SCN12588	-L---Y-----SLFLVD---Q-KLA	G	--I----------S-L--G--R--
	Plasmodium ovale	SCP04394	-LI--F-----SLFLVD---K-KLA	G	--I----------S-L--G--R--
	Plasmodium ovale curt.	SBS83316	-LI--F-----SLFLVD---K-KLA	G	--I----------S-L--G--R--
	Plasmodium ovale wal.	SBT34864	-LI--F-----SLFLVD---K-KLA	G	--I----------S-L--G--R--
	Plasmodium reichenowi	XP_012763470	-L---F-----SLFLVD---Q-KLA	G	--I----------S-L--G--R--
	Plasmodium relictum	CRG99936	-LI--Y-----SLFLVD---Q-KLA	G	--I----------S-L--G--R--
	Plasmodium vinckei vin.	XP_008624617	-L---F------LFLVD---N-KLA	G	--I----------S-L--G--R--
	Plasmodium vivax Sal-1	XP_001615188	-L---F-----SLFLVD---Q-KLA	G	--I----------S-L--G--R--
	Plasmodium yoelii 17X	ETB57938	-LI--Y------LFLVD---N-KLA	G	--I----------S-L--G--R--
	Plasmodium yoelii yoe. 17XNL	XP_731373	-LI--Y------LFLVD---N-KLA	G	--I----------S-L--G--R--
Piroplasmida **(0/8)**	*Babesia bigemina*	XP_012768949	-L---YR-----QLAVDM----KMA	G	RA-------------L-M--A--L
	Babesia ovata	GBE58792	-L---YR-----QLAVDM----KMA	G	RA--F----------L-M--A--L
	Babesia bovis T2Bo	XP_001611896	-LI--YR-----QLAVDM--A-KMA	G	RA-------------L-M--AR--
	Babesia microti strain RI	XP_021337180	-LI--YH-----FLVVD---S-KMA	G	-AV-------T----L-M--C--L
	Theileria orientalis	PVC56793	-LI--F------L-AVDM--S-KMA	G	-A-------------L-MG-AR-L
	Theileria parva strain Muguga	XP_764299	-LI--F------L-AVDM--S-KMA	G	-A-------------L-MG-AR-L
	Theileria equi strain WA	XP_004831192	-LI--F------LLAVDM--S-KMA	G	-A-------------L-M--AR-L
	Theileria annulata	XP_955129	-L---LQ--R---VVV-ML--GK-G	G	RAI----QP--------M----AL

Group	Species	Accession	Sequence
Other Apicomplexa	*Eimeria acervulina*	XP_013247676	-----HQ------LIVE--RN-RMA G -A------P-T------M--AQ-L
	Eimeria brunetti	CDJ50959	-----HL------LIVE--RN-RMA G -A------P-T------M--AQ-L
	Eimeria maxima	XP_013338435	-----HQ------LIVE--RN-RMA G -A------P-T------M--AQ-L
	Eimeria mitis	XP_013349420	-----HQ------LIVE--RN-RMA G -A------P-T------M--AQ-L
	Eimeria necatrix	XP_013439206	-----HQ------LIVE--RS-RMA G -A--I---P-T------M--AQ-L
	Eimeria praecox	CDI75553	-----HQ------LIVE--RN-RMA G -A------P-T------M--AQ-L
	Eimeria tenella	XP_013233010	-----HQ------LIVE--RS-RMA G -A--I---P-T------M--AQ-L
	Cyclospora cayetanensis	XP_022590922	-----HH------LIVE--RN-RMA G -A--I---P-T------M--AQ-L
	Besnoitia besnoiti	PFH35429	-----E-------YVVE--RC-RMA G -A------P-T------M--AQ-L
	Cystoisospora suis	PHJ20088	-----L-------YVVE---C-RMA G -A------P-T------M--AQ-L
	Hammondia hammondi	XP_008886115	-----E-------YVVE--RC-RMA G -A------P-T------M--AQ-L
	Neospora caninum Liv.	CEL64762	-----E-------YVVE--RC-RMA G -A------P-T------M--AQ-L
	Toxoplasma gondii ME49	XP_018638354	-----E-------YVVE--RC-RMA G -A------P-T------M--AQ-L
Other Eukarya	Blastocystis hominis	XP_012897480	-L---E-------MVVDM-NL-KMA G RA------P-T----L-----HQL
	Vitrella brassicaformis CCMP.	CEM27003	-----E--------VVE--RS-KMA G -A-----AP-T------L---Q-L
	Pseudocohnilembus persalinus	KRX01084	-----EL----S-VVVD---S-KMA G RAM-----P-T----L-L---Q-L
	Tetrahymena thermophila SB.	XP_001017389	-----EN------VVVE---T-KMA G RAV-----P-T-----SL---S-L
	Paramecium tetraurelia str.	XP_001425678	-----QI--------VD-V-S-KLA G RA--M---P-T------L-VAQ-L
	Ichthyophthirius multifiliis	XP_004039089	-----EN-------ICD---T-KMA G RAV-----P-T------L-L-T-L
	Conidiobolus coronatus NRRL.	KXN71093	-F----E----C--VVDM--S-RMA G RA-----S--T------Y-----L
	Catenaria anguillulae PL1.	ORZ41559	-F---TL---G--VIVD--RS-KMA G RA------P--------L---Q-L
	Strigomonas culicis	EPY19707	-F----E-------VVE--RS-KMA G RA--F---P-T----L-LG----L
	Leptomonas pyrrhocoris	XP_015660887	-F----T-------AVD--RS-KMA G RA--F---P-T----L-LG-A--L
	Lepeophtheirus salmonis	ADD24472	-L---EQT------VLD---A-KMA G RAVI----P-T------L-V-Q-L
	Haemonchus contortus	CDJ83203	-L---TL--T---V-VD-V-M--MA G RA------P-T------L--AQ-L
	Clunio marinus	CRK91792	-L----D-------AVD---S-KMA G RA------P-T------M--AH-L
	Anopheles darlingi	ETN67049	-L----N------VVVD---S-KMS G RA------P-T------L--AH-L
	Anopheles sinensis	KFB41343	-L----D------VVVD---S-KMS G RA------P-T------L--AQ-L
	Lasius niger	KMQ92600	-L---EM-------VVDM--S-RMA G RAV-----P-T------L--AQ-L
	Lucilia cuprina	KNC30618	-L----A-------VVD---S-KMA G RA------P-T------L--AQ-L
	Operophtera brumata	KOB70746	-L---ES------LVVDM-RS-KMA G RA------P-T------L--AQDL
	Melipona quadrifasciata	KOX69059	-L---EM------VVVDM--S-KMA G RAV-----P-T------L--AQ-L
	Drosophila melanogaster	NP_652608	-L----A-------VVD---S-KMA G RA------P-T------L--AQ-L
	Trichomalopsis sarcophagae	OXU21407	-L---E--------VVDM-RT-KMS G RAI-F---P-T------L--AQ-L
	Heliothis virescens	PCG63791	-L---ES-------VVDM-RS-KMA G RA------P-T------L--AQDL
	Drosophila pseudoobscura pse.	XP_001358203	-L----A-------VVD---S-KMA G RA------P-T------L--AQ-L
	Nasonia vitripennis	XP_001603203	-L---E--------VVDM-RT-KMS G RAI-F---P-T------L--AQ-L
	Aedes aegypti	XP_001649604	-L----D-------VVD---S-KMS G RA------P-T------L--AQ-L

Figure S35. A partial sequence alignment the RuvB-like helicase 1 protein showing a one amino acid deletion that is specific for members of *Plasmodium.* The CSI is not seen in the other identified homolog.

			136		194
	Plasmodium berghei ANKA	CDS46888	RKSIHIKIKEEKLVYEGEVVDMIVEENEC	L	YSQNKAKQINAIIITLKTVKGTKSLRLAP
	Plasmodium sp. Gor. Cla. G	SOV15340	----------------------V------	-	--L------------------S-T-----
	Plasmodium sp. DRC-Itaito	SOV23144	----------------------V------	-	--L------------------S-T-----
	Plasmodium chabaudi cha.	CAH83857	----------------------V------	-	-----------------------------
	Plasmodium coatneyi	XP_019915132	---------D------------V------	M	--L------------------A-T-----
	Plasmodium cynomolgi str. B	XP_004222291	---------D------------V------	-	--L------------------A-T-----
	Plasmodium falciparum 3D7	XP_001347747	----------------------V------	-	--L--------------S---S-T-----
	Plasmodium falciparum Dd2	KOB85310	----------------------V------	-	--L--------------S---S-T-----
	Plasmodium falciparum IGH-CR14	KNG76139	----------------------V------	-	--L--------------S---S-T-----
	Plasmodium falciparum Tan.	ETW36102	----------------------V------	-	--L--------------S---S-T-----
	Plasmodium fragile	XP_012336553	---------D------------V------	-	--L------------------S-T-----
	Plasmodium gaboni	XP_018640980	----------------------V------	-	--L------------------S-T-----
***Plasmodium* (20/20)**	*Plasmodium gallinaceum*	CRG96118	----------------------V------	-	--L--------------A---S-T-----
	Plasmodium gonderi	GAW80838	---------D------------V------	-	--L------------------S-T-----
	Plasmodium inui San Ant. 1	XP_008817838	---------D------------V------	-	--L------------------A-T-----
	Plasmodium knowlesi str. H	XP_002259060	---------D------------V------	-	--L------------------A-T-----
	Plasmodium malariae	SBS85123	----------------------L------	-	--L-R----------------S-T-----
	Plasmodium ovale cur.	SBS83316	-----------------------------	-	--L------------------S-T-----
	Plasmodium ovale wal.	SBT34864	-----------------------------	-	--L------------------S-T-----
	Plasmodium reichenowi	XP_012763470	----------------------V------	-	--L------------------S-T-----
	Plasmodium relictum	CRG99936	----------------------V------	-	--L---------------A--S-T-----
	Plasmodium vinckei vin.	XP_008624617	-----------------------------	-	-----------------------------
	Plasmodium vivax Sal-1	XP_001615188	---------D------------V------	-	--L------------------A-T-----
	Plasmodium yoelii 17X	ETB57938	-------------------I---------	-	-----------------------------
	Plasmodium yoelii yoe. 17XNL	XP_731373	-------------------I---------	-	-----------------------------
	Babesia sp. Xin.	ORM40192	------IL----Q------TELVA--T-N		P-GGF--C-S-V-----------T-----
	Babesia bovis T2Bo	XP_001611896	------IL----Q------TELVA--T-N		P-GGF--C-S-V-L---------T-----
	Babesia bigemina	XP_012768949	------IL----Q------TELVA--T-N		P-GGF--CVS-VVV---------T-----
***Piroplasmida* (0/6)**	*Babesia microti str. RI*	XP_021337180	-----VR-----Q----Q--ELAA--T-N		PHGGYG-CVV-V------AR---T-----
	Theileria equi	XP_004831192	------LL-D--QI-----TELVA--T-N		P-GGF--CVS-VVV-------S-T-----
	Theileria annulata	XP_953307	------V--D--QI-----TELTA--V-N		PTGGF--CM-GVLV-------S-T-----
	Theileria orientalis str. Shi.	XP_009692162	------V--D--QI-----TELTA--VDN		PTGGFS-C--GVL--------S-T-----
	Besnoitia besnoiti	PFH37556	--A-ALR-H-V-E------LEIAA--T-N		PHGGF--CLS-V-M-----R---T-----
	Eimeria brunetti	CDJ49901	-RALGLR-R-V-E-F-----QLAA--A-N		PHGGFG-C-S-VML----A--M-T-----
	Eimeria tenella	XP_013233086	-RALGLR-R-V-E-F-----QLAA--A-N		PHGGFG-C-S-VML-------M-T-----
	Eimeria praecox	CDI84853	-RALGLR-R-V-E-F-----QLAA--A-N		PHGGFG-C-S-VML-------M-T-----
**Other Apicomplexa**	*Eimeria maxima*	XP_013337232	-RALGLR-R-V-E-F-----KLAA--A-N		PHGGFG-C-S-VML-------V-T-----
	Cryptosporidium hominis TU502	XP_668483	-RA-GLR-RDV-E-------ELVT--T-N		PHG-FG-AVS--VL---SA----T-----
	Cryptosporidium ubiquitum	OII74083	-RA-GLR-RDV-E-------ELVT--T-N		PHG-FG-AVS--VL---SA----T-----
	Cryptosporidium andersoni	OII75775	-R--GLR-RDI-E------TEL-T--T-N		PHG-FG-AVS--LV---SA----T-----
	Cryptosporidium muris RN66	XP_002141376	-R--GLR-RDI-E------TEL-T--T-N		PHG-FG-AVS--LV---SA----T-----
	Gregarina niphandrodes	XP_011131659	-RA-GLR-R-V-D------LELS---T-N		PHGGF--AMS-VVVV---SA---T-K---
	Thalassiosira oceanica	EJK49966	-RA-GLR-R-N-E------TELT---T-D		PLGGYGRS-SHVV-S--ST----T-K-D-
	Fragilariopsis cylindrus CC.	OEU22216	--A-GLR-Q-R-E------TELT---T-D		PLGGYGRT-SHV-LG---A--S-T-K-D-
	Thalassiosira pseudonana CC.	XP_002293057	-RA-GLR-R-S-E------TELT---T-D		PLGGYGRS-SHVV----ST--S-T-K-D-
	Ostreococcus tauri	CEF97733	-RA-GLR---V-E-------E-TP--T-S		T-GGYG-V-SHV-VG--S-----Q-K-D-
	Ostreococcus lucimarinus CCE.	XP_001417360	-RA-GLR---V-E-------E-TP--T-S		T-GGYG-V-SHVVVG--S-----Q-K-D-
	Bathycoccus prasinos	XP_007511746	-RA-GLR---N-E-----I-E-TP--T-S		T-GGYG-V-TSVVVG--------Q-K-D-
	Vitrella brassicaformis CCMP.	CEM27003	-RA-G-R---V-E------TELTP--T-N		PHGGFG-AVS--T-G---A----T-K---
	Symbiodinium microadriaticum	OLQ13650	-RA-G-R---T-E------TELTP--RPD		PTGGYG-KVT-VM-G---T--Q-T-K---
	Saccharomycetaceae sp. 'Ash.	AGO13750	-RA-GLR---V-E------TELTP--A-N		PLGGYG-T-SHV-VG--SA----T---D-
	Saccharomyces cerevisiae YJM.	AJU59415	-RA-GLR---T-E------TELTP-DA-N		PLGGYG-T-SHV-VG--SA----T---D-
**Other Eukarya**	*Metschnikowia bicuspidata var.*	XP_018711124	--A-GLR---T-E------IELTP--A-N		PLGGYG-T-SHV-VG---A--------D-
	Wickerhamomyces anomalus NR.	XP_019039857	-RA-GLR---T-E------TELTP--S-N		PLGGYG-T-SHV-VG--SA----T---D-
	Ascoidea rubescens DSM 19.	XP_020044797	-RA-GLR---I-E------TELTP--S-N		PLGGYG-T-SHV-LG--SA----T---D-
	Candida albicans SC5314	XP_715438	-RA-GLR---T-E------IELTP--A-N		PLGGYG-T-SHV-VG--SA----T---D-
	Amborella trichopoda	ERN15972	-RA-GLR---N-E------TELSP--TDS		I-GGYG-S-SHV--G--------Q-K-D-
	Trifolium subterraneum	GAU16928	-RA-GLR---N-Q------TELSP--TDS		ITGGYG-S-SHV--G--------Q-K-D-
	Cephalotus follicularis	GAV85060	-RA-GLR---N-E------TELSP--T-S		VTGGYG-S-SHV--G--------Q-K-D-
	Eucalyptus grandis	KCW50878	-RA-GLR---N-E------TELSP--T-S		VTGGYG-S-SHV--G--------Q-K-D-
	Citrus sinensis	KDO53640	-RA-GLR---N-E------TELSP--T-S		ITGGYG-S-SHV--G--------Q-K-D-
	Cajanus cajan	KYP54181	-RA-GLR---N-E------TELSP--T-S		VTGGYG-S-SHV--G--------Q-K-D-
	Zea mays	NP_001140836	-RA-GLR---N-E------IELSP--A-S		TTGGY--S-SHV--G--------Q-K-D-

Figure S36. A partial sequence alignment of the RuvB-like helicase 2 protein showing a one amino acid insertion that is specific for members of *Plasmodium*.

Group	Species	Accession	111				157
Plasmodium (20/20)	Plasmodium berghei	CXI63508	IGKPGYDVTKVRNKKN		QLGILFELSFPNIKD	NT	KPKFRFMSSFEQKI
	Plasmodium berghei str. ANKA	XP_676664	----------------		---------------	--	--------------
	Plasmodium reichenowi	XP_012762012	----R----R------		--------------E	--	--------------
	Plasmodium sp. DRC-Itaito	SPJ09370	----R----R------		--------------E	--	--------------
	Plasmodium sp. gorilla clade G2	SOV12610	----R----R------		--------------E	--	--------------
	Plasmodium chabaudi ada.	SCN61125	---------R------		--------------E	--	--------------
	Plasmodium chabaudi cha.	SCM23666	---------R------		--------------E	--	--------------
	Plasmodium coatneyi	XP_019915554	----R------K----		K-------------E	--	--------------
	Plasmodium cynomolgi str. B	XP_004223303	----R------K--R-		K-------------E	--	--------------
	Plasmodium falciparum 3D7	XP_966185	----R----R------		--------------E	--	--------------
	Plasmodium falciparum HB3	KOB59349	----R----R------		--------------E	--	--------------
	Plasmodium fragile	XP_012335279	----R------K--R-		K-------------E	--	--------------
	Plasmodium gaboni	XP_018642832	----R----R------		--------------E	--	--------------
	Plasmodium gallinaceum	CRG93777	----R------K----		-I------------E	--	--------------
	Plasmodium gonderi	GAW81828	----R------K--R-		K-------------E	--	-------------V
	Plasmodium inui San Ant. 1	XP_008814744	----R------K--R-		K-------------E	--	--------------
	Plasmodium knowlesi str. H	XP_002261476	----R------K--R-		K-------------E	--	-------------V
	Plasmodium malariae	SBT00115	----R------K----		-R------------E	--	--------------
	Plasmodium ovale curt.	SBS85333	----R-----------		--------------E	--	--------------
	Plasmodium ovale wal.	SBT38129	----R-----------		--------------E	--	--------------
	Plasmodium relictum	CRH00937	----R------K----		--------------E	--	--------------
	Plasmodium vinckei pet.	EUD73305	---------R------		--------------E	--	--------------
	Plasmodium vinckei vin.	XP_008624396	---------R------		--------------E	--	--------------
	Plasmodium vivax Sal-1	XP_001616212	----R------K--R-		K-------------E	--	--------------
	Plasmodium yoelii 17X	ETB56749	----------------		--------------E	--	--------------
	Plasmodium yoelii yoe. 17XNL	XP_731238	----------------		--------------E	--	--------------
Piliocolobus	Piliocolobus tephrosceles	XP_023056474	------------		-R------------E	--	--------------
Piroplasmida (0/8)	Theileria annulata	84994716	--R---RI--M-DPET	K	-PAL---IE--E-QG		T--Y----A-----
	Theileria equi	510901151	--R---RI--MKDPAT	Q	-PAL---IE--E-EG		R-RH----A----V
	Theileria orientalis str. Shi.	697890843	--R---RI--M-DPVT	K	-PAL---IE--E-QG		A--Y----A----M
	Theileria parva str. Mug.	71031120	--R---RI--M-DPET	K	-PAL---IE--E-QG		T--Y----A-----
	Babesia bigemina	833481147	--R---RI--MHDPET	K	-Y-L----EY-D-EG		--RY-L--A---RM
	Babesia bovis T2Bo	156085559	--R---RI--M-DPET	N	-FAL---IEY-D-EG		--RY-V--A---RM
	Babesia ovata	GBE60785	--R---RI--MHDPET	K	-Y-L----EY-D-EG		--RY-L--A---RM
	Babesia microti str. RI	1206244999	--R---R---M-DPET	G	-NAL---IEYTE-DG		R--H----A----V
	Babesia sp. Xin.	1181628920	--R---RI--MQDPET	K	-Y-L---IEY-D-EG		--RY-L------RM
Other Apicomplexa	Gregarina niphandrodes	749162253	--R---R---L-DPST	K	-Y-LR--IH-KDSSS		--RH----TW---V
	Toxoplasma gondii ME49	237834251	--R---R-S-L-DPDS	L	-KAL---IDY-E-NE	GA	--YH--------RV
	Hammondia hammondi	675120516	--R---R-S-L-DPDT	L	-KAL---IDY-E-NE	GA	--YH--------RV
	Cystoisospora suis	PHJ22395	--R---R-S-L-DPET	L	-KAL---IDY-E-NE	-A	--FH--------RV
Other Eukarya	Absidia glauca	SAM07920	--R---RI----DPAT	R	---M--QIQY-Q-AN	DV	L-RH----AY--HV
	Absidia repens	ORZ08340	--R---RI----DPST	R	---M--QIQY-Q-AN	DV	I-RH----AY--H-
	Acidomyces richmondensis	KXL48746	--R---KI--T-DPVT	R	-D-L--Q-QY-E-SP	--	I-RI---GAY---V
	Amborella trichopoda	XP_011625383	--R---R---QFDSDT	K	-RSL--QIEY-E-E-	--	--RH--------RV
	Anaeromyces robustus	ORX87666	--R---K-----DPIT	K	---L--QIQY-E-A-	-V	I-RY----AY--H-
	Ancylostoma ceylanicum	EPB77610	--R---K--RE-DPAT	G	-QAL--QIDY-E-AE	GV	Q-RH----AY----
	Ascochyta rabiei	KZM22758	--R---RI----DPIT	R	-N-L--QFQ--D-TP	D-	I-RV----AY---M
	Ascoidea rubescens DSM 1968	XP_020045746	----A-KI--I-DPIS	Y	EI-L--TIQ--KL-N	DI	A--Y-M--C---QV
	Aspergillus brasiliensis CB.	OJJ74791	--R---KI--I-DPLT	R	---M--Q-Q-QE-TP	GV	T--V----A----V
	Aspergillus lentulus	GAQ08317	--R---KI--I-DPLT	R	---L--Q-QYQE-TP	GV	--RV----A----V
	Aureobasidium namibiae CBS.	XP_013430298	--R---KI--T-DPLT	R	---L--Q-QY-E-SQ	DV	Q-RV----A----V
	Aureobasidium pullulans EXF.	KEQ84851	--R---KI--T-DPLT	R	---L--Q-QY-E-SQ	DV	Q-RV----A----V
	Cladophialophora carrionii CBS.	XP_008729035	--R---KI--I-DPLT	R	-V-L--Q-LY-E-TP	-V	E--V----A----V
	Cryptococcus amylolentus CBS.	XP_018992819	--R---KII-I-EPVS	Q	RM-L--TV-L-E--Q	GE	R-RR----A---RR
	Diplodia corticola	XP_020134357	--R---KI--I-DPLT	R	-H-L--Q-QY-E-NQ	-I	L-RV----A----V
	Diplodia seriata	KKY14113	--R---KI--I-DPLT	R	-H-L--Q-QY-E-NQ	-I	V-RV----A----V
	Drechslerella stenobrocha 248	EWC48409	--R---KI--C-DPVT	R	-Q-L--Q-QY-E-GT	EI	H-RY----A----V
	Drosophila serrata	XP_020799891	--R---R---Q-ELS-	G	-QSL--QIDY-E-SE	-I	V-RH----AY----
	Ectocarpus siliculosus	CBN77971	--R---K---A-DLSS	N	-RSL---VDY-EAE-	GA	Q-RH----AY---V
	Elaeis guineensis	XP_010941544	--R---R---QFDSET	K	-RSL--QIEY-E-E-	--	--RH--------RV
	Erysiphe necator	KHJ31106	--R---KI--T-DPVT	R	-Q-LV-Q-QY-E-SQ	DV	T--V----A-----

Figure S37. A partial sequence alignment of the Splicing factor 3A subunit 2 protein showing a one amino acid deletion that is specific for members of *Plasmodium* and *Piliocolobus tephrosceles*. The presence of this CSI within the latter species is likely due to contamination. A two amino acid deletion is also seen in *Piroplasmida* and *Gregarina niphandrodes*.

Group	Species	Accession	181		221
***Plasmodium* (20/20)**	*Plasmodium chabaudi ada.*	SCM24886	ITIQKTTKARKKTVTVVKGLHAY		AKLDKMAKIFSKFYACGA
	Plasmodium chabaudi cha.	SCM26222	-----------------------		------------------
	Plasmodium sp. DRC-Itaito	SPJ09867	-------R-K--V----T---P-		I--E-------------S
	Plasmodium sp. Gor. Cla. G2	SOV13601	-------R-K--V----T---P-		I--E-------------S
	Plasmodium coatneyi	XP_019917001	-------R----V----V---T-		V----------R------
	Plasmodium cynomolgi str. B	XP_004224865	-------R----V----V---T-		V----------R------
	Plasmodium falciparum 3D7	XP_001349389	-------R-K--V----T---P-		I--E-------------S
	Plasmodium falciparum Mal.	ETW49898	-------R-K--V----T---P-		I--E-------------S
	Plasmodium falciparum NF135/5.	ETW43279	-------R-K--V----T---P-		I--E-------------S
	Plasmodium falciparum NF54	EWC88929	-------R-K--V----T---P-		I--E-------------S
	Plasmodium falciparum Tan.	ETW37134	-------R-K--V----T---P-		I--E-------------S
	Plasmodium fragile	XP_012337914	-------R----V----V---T-		V----------R------
	Plasmodium gaboni	XP_018642231	-------R-K--V----T---P-		I--E-------------S
	Plasmodium gallinaceum	CRG97648	-------R----V----T---T-		V--E-I-----R-----S
	Plasmodium gonderi	GAW83462	-------R----V---IV---T-		V----------R------
	Plasmodium inui San Ant. 1	XP_008814470	-----I-R----V-I--V---T-		V----------R------
	Plasmodium knowlesi str. H	XP_002262152	-------R----V----V---T-		V----------R------
	Plasmodium malariae	SBT01039	----RK-R----V----T---Q-		V--E-I-----R-----S
	Plasmodium ovale curt.	SBS88269	-------R----V----T-----		V----------R-----S
	Plasmodium ovale wal.	SBT48021	-------R----V----T-----		V----------R-----S
	Plasmodium reichenowi	CDO63905	-------R-K--V----T---P-		I--E-------------S
	Plasmodium relictum	CRH02508	-------R----V----T---T-		---E-------R-----S
	Plasmodium vinckei pet.	EUD74313	-----------------------		T-----------------
	Plasmodium vinckei vin.	XP_008622989	-----------------------		T-----------------
	Plasmodium vivax Sal-1	XP_001617181	-------R----V----V---T-		V----------R------
	Plasmodium berghei str. ANKA	XP_670091	---------K-------------		T-----------------
	Plasmodium yoelii 17X	ETB61523	---------K-----------V-		T-----------------
***Piroplasmida* (0/8)**	*Babesia bigemina*	XP_012769218	V-V-RS-RSKR-V--S-T---LF	G	V--EAA--L-A-QF-S--
	Babesia bovis	XP_001611655	----RS-RSKR-I--TIS---LF	G	V--EVA--M-A-QF-S--
	Babesia microti str. RI	XP_012648437	V---RVSR-KR-V--A-S---LF	G	V---AAS-L---QF-T--
	Babesia sp. Xin.	ORM41923	V-V-RS-RSKR-I--T-T---LF	G	V--EVA--L-A-QF-S--
	Theileria annulata	XP_952289	-F---SARTKR-VI-SIT---LF	G	V--EQA--L---HF-T--
	Theileria equi	XP_004828986	V---RSSRTKR-VI-SIT---LF	G	V--EPLC-Q---LF-T--
	Theileria orientalis str. Shi.	XP_009690457	-V---AARTKR-AI-S-T---LF	G	V--EEAS-L---QF-T--
	Theileria parva str. Mug.	XP_764995	-F---SARTKR-VI-S-T---LF	G	V--EQA--L---HF-T--
**Other Apicomplexa**	*Besnoitia besnoiti*	PFH33521	V---RQSR-KR--A---T--ELF	G	V----A--L---Q-----
	Cystoisospora suis	PHJ16087	V---RQSR-KR--A--IT--DLF	G	V----A--L---Q-----
	Hammondia hammondi	XP_008883265	V---RQSRSKR--A--IT--DLF	G	V----A--L---Q-----
	Neospora caninum Liv.	XP_003883233	V---RQSRSKR--A--IT--DLF	G	V----A--L---Q-----
	Toxoplasma gondii ME49	XP_002364953	V---RQSRSKR--A--IT--DLF	G	V----A--L---Q-----
	Cryptosporidium andersoni	OII77027	V-VKVESRT-R-S----T--ELF	D	IS-SEA--K-A-HFS--S
	Cryptosporidium hominis TU502	XP_667998	---KVESR--R-N----N--ELF	D	IQ-NEA--K-A-QFS---
	Cryptosporidium muris RN66	XP_002140034	V-VKVESR--R-S----T--ELF	D	IS-SEA--K-A-HFS--S
	Cryptosporidium parvum Iowa II	XP_627189	---KVESR--R-N----N--ELF	D	IQ-SEA--K-A-QFS---
	Cryptosporidium ubiquitum	OII73717	---KVESR--R-N----C--ELF	D	IQ-NEA--K-A-QFS---
**Other Eukarya**	*Vitrella brassicaformis CCMP.*	CEL94144	V-V-RQNRKGR-FI-T----ETF	G	V----AS-V---AFS---
	Sordaria macrospora k-he.	CCC06248	V--KRIERNKR-Y--S-S--E-F	G	LE-K-V--D-G-KF-T-S
	Lichtheimia ramosa	CDS06859	V-VKRVERTKR-C--I-H--EVF	D	VD-K-A--M-ANRF---S
	Rhizopus microsporus	CEG67277	VL-KRNERNKR-C--TIH--DIF	G	VD-K-A--M-ANRF---S
	Rhizopus delemar RA 99-880	EIE89365	VI-KRNERNKR-C--TIY--DIF	G	VD-K-A--M-ANRF---S
	Mortierella verticillata NR.	KFH72277	VL-KRIERTKR-C--C-F--ENF	D	VD-K-A--L-ANKF---S
	Allomyces macrogynus ATCC 38.	KNE56148	VI-KSEKRKG--V---IT--DVF	G	VD-K-AS-L--QKF-T-S
	Microdochium bolleyi	KXJ88250	V--KRIERNKR-Y--S-T--E-F	N	LD-K-V--E-G-KF-T-S
	Syncephalastrum racemosum	ORZ00927	VMVKRVERTKR-CI-I-H--EIF	D	VD-K-A--M-ANRF---S
	Absidia repens	ORZ07661	V--KRVERTKR-RI-TIH--DIF	G	VD-K-A--L-ANKF---S
	Piromyces sp. E2	OUM65496	---KRCERTKR-HQII-N--DQ-	E	IE-K-A--L--SRF---S

Figure S38. A partial sequence alignment of the Translation initiation factor SUI1 protein showing a one amino acid deletion that is specific for members of *Plasmodium.*

Group	Species	Accession	135		185
Mammalian infecting ***Plasmodium*** **(18/18)**	*Plasmodium falciparum 3D7*	XP_024329092	RVSEYVGDIVKYIEKIIENKYAYVS	E	EGSVYFDIDEFKKSEKHFYARMEPL
	Plasmodium falciparum Pal. Alt.	ETW56568	-------------------------	-	-------------------------
	Plasmodium gaboni	XP_018641535	------D------------------	-	-------------------------
	Plasmodium reichenowi	SOV79741	-------------------------	-	-------------------------
	Plasmodium sp. DRC-Itaito	SPJ10566	-------------------------	-	-------------------------
	Plasmodium sp. Gor. Cla. G2	SOV77138	-------------------------	-	--------------GN---------
	Plasmodium vivax Ind. VII	KMZ80883	------DQ-IH------D-------	-	--------------P----------
	Plasmodium knowlesi str. H	SBO29533	------DQ-IH--------------	-	---------A---NP----------
	Plasmodium inui San Ant. 1	XP_008815940	------DQ-IH------D-------	-	-------------IP----------
	Plasmodium gonderi	GAW80575	------EE-----------------	-	-------------NP----------
	Plasmodium fragile	XP_012337164	------DQ-IQ--Q-----------	-	---------A---NP----------
	Plasmodium cynomolgi str. B	XP_004222036	------DQ-IH------D-------	-	-------------NP----------
	Plasmodium coatneyi	XP_019914392	------DQ-IQ------A-------	-	---------A---DP----------
	Plasmodium chabaudi cha.	SCM24562	------NE-I-F-------------	S	---------A---NPN--------M
	Plasmodium chabaudi ada.	SCN61934	------NE-I-F-------------	S	---------A---NPN--------M
	Plasmodium berghei ANKA	XP_022713336	------NE-I-F-------------	S	---------A---NPN--------M
	Plasmodium yoelii yoe.	EAA16509	------NE-I-F-------------	S	---------A---NPN--------M
	Plasmodium yoelii 17X	ETB62145	------NE-I-F-------------	S	---------A---NPN--------M
	Plasmodium vinckei vin.	XP_008623378	------NE-I-F-------------	S	---------A---NPD--------M
	Plasmodium vinckei pet.	EUD72541	------NE-I-F-------------	S	---------A---NPN--------M
	Plasmodium ovale wal.	SBT34179	-----IDE-IE--K-----------	-	---------A---NP----------
	Plasmodium ovale curt.	SBS82865	-----IDE-I---K---D-------	-	---------A---NP----------
	Plasmodium malariae	SBS84520	------NE-IS--------------	-	-------------NP--Y-------
**Avian Infecting *Plasmodium*, "*Haemamoeba*" (0/2)**	*Plasmodium gallinaceum*	CRG94210	------D--I---------------		--------E----K-N--------S
	Plasmodium relictum	CRG99705	------D--IR------------I-		--------E----N-I--------S
**Other Apicomplexa**	*Babesia bigemina*	XP_012767680	----FLPE-ID---T--K-G---E-		D-------EA-RTN-A-V--KL--T
	Babesia bovis T2Bo	XP_001608890	----FLPE-IS---T-MK-G---EA		D--------A-IG-D--V--KL--N
	Babesia ovata	GBE61326	----FLPE-ID---T--K-G---E-		--------EA-RTNKA-V--KL--T
	Babesia sp. Xin.	ORM40534	----FLPE-IS---A-MK-G---EA		D-------EA-RA----V--KL--N
	Theileria annulata	XP_954780	---D-MTE--DF-K--LD-G-----		D-------ES-RN-G--V--KL--T
	Theileria equi	XP_004833028	---D-ME----F-----A-G---E-		D-------EA-RN-G--V--KL--T
	Theileria orientalis str. Shi.	XP_009691837	---D--EE--EF--R-VS-G---E-		--------EA-RS-G--A--KL--N
	Theileria parva str. Mug.	XP_763084	---D-MVE--DF-K--LD-G-----		D-------EA-RS-G--V--KL--T
	Cyclospora cayetanensis	XP_022586743	------PEVLRL--E-EA-GF--E-		Q------TQR-S--A--T-G----G
	Besnoitia besnoiti	PFH37612	-----IPEVLSL-QR-EK-G---E-		Q------TQS-RN-D--I-G----S
	Hammondia hammondi	XP_008888417	-----IPEV-AL--R-VK-G-G-E-		Q------TQT-R-N---V-G----T
	Neospora caninum Liv.	CEL70364	-----IPEVLSL--R-VK-G-G-E-		Q------TQT-R-TD--I-G----S
	Toxoplasma gondii ME49	XP_018635210	-----IPEV-AL--R-VK-G-G-E-		Q------TQT-R-N---V-G----T
	Cryptosporidium hominis	OLQ17505	----FIPE-IDF-ST--SRGF--E-		-------VES-RAD---V-G----K
	Cryptosporidium meleagridis	POM82953	----FIPE-IDF-ST--SRGF--E-		-------VES-RAD---V-G----K
	Cryptosporidium muris RN66	XP_002140066	----FIPE-LD--ST--YRGF--EC		D-------NA-RSK-G---G----K
	Cryptosporidium parvum Iowa II	XP_627469	----FIPE-IDF-ST--SRGF--E-		-------VES-RAD---V-G----K
	Cryptosporidium ubiquitum	OII72991	----FIPE-IDF-ST--SRGF--E-		D------VET-RAD---V-G----K
	Gregarina niphandrodes	XP_011131114	----FIPE-Q-F-L---D-G---E-		G------TTA-RN----V-G----W
**Other Eukarya**	*Vitrella brassicaformis CCMP.*	CEM20223	------DEV-GF-Q---D-----E-		S------VAA--S-GQ-V-G----W
	Acinonyx jubatus	XP_014936514	------PE--NFVQ--VD-G-G---		N------TVK-AS--R-S-GKLV-E
	Apteryx australis man.	XP_013813315	------PE--EFVK--MD-G-G---		N------TMK-DSA---S--KLV-E
	Taeniopygia guttata	XP_012429737	------PE--DFVK--VD-G-G---		N------TMK-DA----S--KLV-E
	Tetrahymena thermophila SB.	XP_001010154	------PE-IS--------G----A		N-----NV-K-HE-PT-C-SKL--Q
	Trachymyrmex cornetzi	XP_018359256	-----I-E-IAF-QQ--D-G---E-		N------V-K-D-Q---S--KLV-E
	Trichogramma pretiosum	XP_014237173	------PE--AF-----A-GF--E-		N------VKA-D-K---Y--KLV-E
	Trichomalopsis sarcophagae	OXU20446	------PE--AF-----A-GL--E-		N------VKA-D-N---Y--KLV-E
	Xenopus tropicalis	OCA14793	------PE--AFVQR-VD-G-G---		N-----STAK-HA----Y--KLV-E

Figure S39. Alignment of the Cysteine-tRNA ligase protein, showing a one amino acid insertion that is exclusive to all mammalian infecting *Plasmodium*. Expanded alignment of Figure 4 (B).

Group	Species	Accession	146		197
**Avian Infecting *Plasmodium*, "*Haemamoeba*" (2/2)**	*Plasmodium relictum*	CRH00140	EGCCYVSERCYAKKSNIDNQLILGYKD	S	DPFDLDNKEMMEKLKSLAWNTDDI
	Plasmodium gallinaceum	CRG96319	-----A--K--V-EA-V--------R-	K	--Y--KKE-L-T---N-T------
**Other *Plasmodium* (0/18)**	*Plasmodium berghei ANKA*	XP_022712909	-----TI---NINNI--T-YNL--THI		N-YEI-S-IRDKE-AT-N-DS-NL
	Plasmodium sp. DRC-Itaito	SOV23348.1	---S-TA-H--N--N--FHHI---HQE		N-Y-INED-LND--N-MS----NL
	Plasmodium chabaudi ada.	SCM21149	-----TI---SV-SDM-T-YP---TSI		N-Y-V-P-IRNKE-AT-N-DS-NL
	Plasmodium chabaudi cha.	XP_016655395	-----TI---SV-SDM-T-YP---TSI		N-Y-V-P-IRNKE-AT-N-DS-NL
	Plasmodium coatneyi	XP_019915008	-----T-----V-DA--I--V-R-HSE		--YEV-A-TLENE----N-D--QL
	Plasmodium cynomolgi str. B	XP_004222493	-----T-Q----RDA--V--VL--HEE		--Y-V-S-TLENE----N-D--KL
	Plasmodium falciparum 3D7	XP_001347952	---S-T--H-FN--N--F-HI-V-HQE		N-Y-INED-IDD----MS----NL
	Plasmodium falciparum 7G8	EUR70220	---S-T--H-FN--N--F-HI-V-HQE		N-Y-INED-IDD----MS----NL
	Plasmodium falciparum FCH/4	ETW28607	---S-T--H-FN--N--F-HI-V-HQE		N-Y-INED-IDD----MS----NL
	Plasmodium falciparum Vie.	ETW15422	---S-T--H-FN--N--F-HI-V-HQE		N-Y-INED-IDD----MS----NL
	Plasmodium fragile	XP_012333086	-----T-Q--NI-DASVV-EV---HQE		--YEV-A-TLENE----N-D--KL
	Plasmodium gaboni	SOV15735	---S-TA-H--N--N--FHHI---HQE		N-Y-INED-LND--N-MS----NL
	Plasmodium gonderi	GAW81039	---S-T-------DAT-I--V---HSE		--YNV-I-TQENE----N-D--QL
	Plasmodium inui San Ant. 1	XP_008816089	-----T-Q---V-DAT-I--V---HQE		N-Y-V-A-TLENE----N-D--KL
	Plasmodium knowlesi str. H	XP_002259275	-----T-----V-DA--V--I-R-HQE		--YEV-A-TLDNE----N-D--QL
	Plasmodium malariae	SBS85607	D----TAQ--NI-DAS-I-YV---H--		N---I---TLEQE---QN-D--KL
	Plasmodium ovale curt.	SBS83709	-----T-Q---V-DA--V-YV---HSE		--Y-V-K-TLEKE-V--N-D--KL
	Plasmodium ovale wal.	SBT35839	-----T-Q---V-DA--V-YV---HSE		--Y-V-K-TLEKE-V--N-D--KL
	Plasmodium reichenowi	XP_019970328	---S-TA-H-FN--N--F-HI-V-HEE		N-Y-INED-IDD----MN----NL
	Plasmodium sp. Gor. Cla. G2	SOV78123	---S-T--H--N--N--F-HI---HQE		N-Y-INED-LND----MS----NL
	Plasmodium vinckei pet.	EUD72878	-----TI---SV-NDI-T-YP---TSI		N-YEV-P-IRDKE-AT-N-DS-NL
	Plasmodium vinckei vin.	XP_008624812	-----TI---SV-NDM-T-Y----TSI		N-YEV-P-IRDKE-AT-N-DS-NL
	Plasmodium vivax Bra. I	KMZ86622	-----T-Q----RDA--V--VL--HQE		--YEV-S-TLENE----N-D--QL
	Plasmodium vivax North Kor.	KMZ99533	-----T-Q----RDA--V--VL--HQE		--YEV-S-TLENE----N-D--QL
	Plasmodium yoelii yoe.	EAA21549	-----TI---NINNI--T-YNL--THI		N-YEV-S-IRDKE-AT-N-DS-NL

Figure S40. Alignment of a Protein phosphatase, showing a one amino acid insertion that is exclusive to the "*Haemamoeba*" subgenus. Expanded alignment of Figure 5 (A).

```
                                                                              5                                                      58
"Haemamoeba"    Plasmodium gallinaceum        CRG96454       ETQNLKATPNDGNEENVY D KDTHINSSQDN N YNEATQNLGKTNPIEGYYTVTHE
(2/2)           Plasmodium relictum           CRH00275       ----I-----N------- E ----N------ - -D-S--------NVG--------
                Plasmodium coatneyi           XP_019914922   -A-S--VVQSSA--QS--   ----TTTLPE-   ---S-I-V-----L------AQD
"Plasmodium"    Plasmodium cynomolgi str. B   XP_004222622   -A-S--VVQSNP--QSA-   ----TTALPE-   ---SAN-V-----L------AQD
(0/6)           Plasmodium gonderi            GAW81166       DA----I-QSTA--QG-R   N---T-IL-E-   -A-SSN-V-----L-------QD
                Plasmodium inui San Ant. 1    XP_008816221   -A-S--VVQSSA--Q-A-   ----TTTLPE-   ---SAS-V-----L------AQD
                Plasmodium knowlesi str. H    XP_002259406   -A-S--LVQ-SA--QSM-   ----TTTLPE-   ---S-M-V-----L------PQD
                Plasmodium vivax Ind. VII     KMZ80365       -A-S--VSQSSA--QSA-   ----TTTMAE-   ---SAS-V-----L------AQD
                Plasmodium malariae           SBS96392       -I-H--S-Q-N-K-Q-MC Q Q-G--T-L--S   ---SANTT-----L---------
                Plasmodium ovale curt.        SBS83959       -A-H--GIQGN---QCT- Q E-LN---L--T   ---SAN-------L-------R-
                Plasmodium ovale wal.         SBT35742       -A-H--G-QGN---QCT- Q D-LN---L--T   ---SGN-------L-------R-
                Plasmodium berghei ANKA       XP_679631      KS-HI-SMQ-IEHDQ--- Q E--N--L--G-   -I-SP--T-NPISMS----IP--
                Plasmodium chabaudi ada.      SCM04529       KL-YI-SMQ-NEHDH--- Q E--N-SL--G-   -V-SP--I---ISMS----LP--
Other           Plasmodium chabaudi cha.      XP_746071      KL-YI-SMQ-NEHDH--- Q E--N-SL--G-   -V-SP--I---ISMS----LP--
Plasmodium      Plasmodium falciparum 3D7     XP_001348085   ----I-TASVN-S----- L --A-M--LHE-   --VS-K-VVD---A---H-IS-D
(0/11)          Plasmodium falciparum Dd2     KOB86119       ----I-TASVN-S----- L --A-M--LHE-   --VS-K-VVD---A---H-IS-D
                Plasmodium falciparum Mal.    ETW48497       ----I-TASVN-S----- L --A-M--LHE-   --VS-K-VVD---A---H-IS-D
                Plasmodium gaboni             XP_018641316   ------TSSVN-S----- V --A-M--LHE-   --VS-K-VID---A-----IS-D
                Plasmodium reichenowi         XP_012763801   ----I-TASVN-S----- L --A-M--LHE-   --VS-K-VVD---A-----IS-D
                Plasmodium sp. DRC-Itaito     SPJ11120       ----V-TASVN-S----- V --A-M--LHE-   --VS-K-VVD---A-----IS-D
                Plasmodium sp. Gor. Cla. G2   SOV16014       ------TSSVN-S----- V --A-M--LHE-   --VS-K-VID---A-----IS-D
                Plasmodium vinckei pet.       EUD73023       KP-HM-SMQ-SEHDQ--- Q E--N-SL--G-   -V-SP-TI---MSMS----IP--
                Plasmodium vinckei vin.       XP_008624945   NS-HI-SMQ-SEHDQ--- Q E--N-SL--G-   -V-SP--I---ISMS----IP--
                Plasmodium yoelii yoe.        EAA16697       KS-HI-SMQ-IEHDQ--- Q E--N--L--G-   -I-SP--I-NPISMS----IP--
```

Figure S41. Alignment of a Conserved *Plasmodium* protein, showing a one amino acid insertion that is exclusive the "*Haemamoeba*" subgenus. Also present is a one amino acid deletion that is exclusive to the "*Plasmodium*" subgenus (excluding *P. ovale*, *P. malariae* and *P. fragile*).

Group	Species	Accession	82		130
"Laverania" (5/5)	*Plasmodium falciparum 3D7*	XP_001347362	TVDLRTGQKQKGSIFNKKKIHNKQ	N	TTQAFTQKQQEEDNLYSSRIKTAE
	Plasmodium falciparum FCH/4	ETW30754	------------------------	-	------------------------
	Plasmodium falciparum HB3	KOB60865	------------------------	-	------------------------
	Plasmodium falciparum Vie.	ETW18244	------------------------	-	------------------------
	Plasmodium gaboni	SOV14644	-----A------------------	-	------------------------
	Plasmodium reichenowi	XP_012763117	-------------------L----	-	------------------------
	Plasmodium sp. DRC-Itaito	SPJ10494	------------------------	-	------------------------
	Plasmodium sp. Gor. Cla. G	SOV14664	-----A------------------	-	------------------------
Other *Plasmodium* (0/15)	*Plasmodium berghei ANKA*	XP_675626	---------P--TL------I---		------------------------
	Plasmodium chabaudi ada.	SCM22977	---------P--TL------I---		-----A------------------
	Plasmodium chabaudi cha.	XP_733757	---------P--TL------I---		-----A------------------
	Plasmodium coatneyi	XP_019914361	-----A------PMY-----M---		-----A---E--N-----------
	Plasmodium cynomolgi str. B	XP_004221958	-----A------PMYS----M---		-----A---E--N-----------
	Plasmodium fragile	XP_012335628	-----A------PMY-----M---		-----A---E--N-----------
	Plasmodium gallinaceum	CRG94136	-----A------NLY---------		----------D-------------
	Plasmodium gonderi	GAW80502	-----S------NTY-----T---		------------N-----------
	Plasmodium inui San Ant. 1	XP_008816018	-----A------PLY----NM---		-----A---E--N-----------
	Plasmodium knowlesi str. H	XP_002258710	-----A------PMY-----M---		-----A---E--N-----------
	Plasmodium malariae	SBT00797	------------N-Y-----L---		------------------------
	Plasmodium ovale curt.	SBS82818	------------TLY-----V---		------------------------
	Plasmodium ovale wal.	SBT34002	------------TLY-----V---		------------------------
	Plasmodium relictum	CRG99630	-----AS-----NLYS--------		----------D-------------
	Plasmodium vinckei pet.	EUD72616	---------P--TL------I---		-----A------------------
	Plasmodium vinckei vin.	XP_008623303	---------P--TL------I---		-----A----D-------------
	Plasmodium vivax	XP_001614386	-----A------PMYG----M---		-----A---E--N-----------
	Plasmodium yoelii	XP_724535	---------P--TL------I---		------------------------
Other Apicomplexa	*Babesia bigemina*	XP_012768468	---G-SLV-T-TMQHYRR-VVP--		-----N---M--EAMIM-AKQKHP
	Babesia bovis T2Bo	XP_001611185	---G-SLM-A-NMQHYRR-AVP--		-----N---M--EAMIM-AKQKHP
	Babesia microti str. RI	XP_012650506	---T-SLI-P-TNA-YRRRLQL--		-----N---L--EAMMLDKQ-RS-
	Babesia ovata	GBE62753	---G-SLV-T-TMQHYRR-VVP--		-----N---M--EAMIM-AKQKHP
	Babesia sp. Xin.	ORM42099	---G-SLV-N-NIQHYRR-AVP--		-----N---M--EAMIM-AKQKHP
	Theileria annulata	XP_955469	---S--IV-I-PTTHY---TPI--		-----N--VM--EQ-QF-KQ-QYP
	Theileria equi	XP_004832600	---S-SML-VRS-QHY--RVVL--		-----N--LM--EAMLL-KQ-HP-
	Theileria orientalis	PVC49951	---S--VV-TRPTTHY---VVL--		-----N--VM--EQ-QF-KQ-QYP
	Theileria orientalis str. Shi.	XP_009691125	---S--VV-TRPTTHY---VVL--		-----N--VM--EQ-QF-KQ-QYP
	Theileria parva str. Mug.	XP_762911	---S--IV-I-PTTHY---TPI--		-----N--VM--EQ-QF-KQ-QYP

Figure S42. Alignment of the Eukaryotic translation initiation factor 3 subunit D, showing a one amino acid insertion that is exclusive to the *"Laverania"* subgenus. Expanded alignment of Figure 5 (B).

Group	Species	Accession	322		365
"*Laverania*" (5/5)	*Plasmodium falciparum 3D7*	XP_001349840	DQTQILTSSYDKSVKIFGLKSLKCLKEFRKHE	D	SVVHSAIYTLD
	Plasmodium falciparum 7G8	EUR66802	--------------------------------	-	-----------
	Plasmodium falciparum Dd2	KOB85949	--------------------------------	-	-----------
	Plasmodium falciparum FCH/4	ETW29683	--------------------------------	-	-----------
	Plasmodium falciparum HB3	KOB60606	--------------------------------	-	-----------
	Plasmodium falciparum Tanz.	ETW34981	--------------------------------	-	-----------
	Plasmodium falciparum UGT5.1	EWC74911	--------------------------------	-	-----------
	Plasmodium falciparum Vie.	ETW17122	--------------------------------	-	-----------
	Plasmodium gaboni	XP_018639918	---------------V-----------I-I--	N	AI---------
	Plasmodium reichenowi	XP_012764492	--------------------------------	-	-----------
	Plasmodium sp. DRC-Itaito	SPJ11834.1	--------------------------------	-	-----------
	Plasmodium sp. Gor. Cla. G2	SOV17492.1	---------------V-----------L-I--	N	VI---------
Other *Plasmodium* (0/15)	*Plasmodium berghei ANKA*	XP_680332	-------C---NT---H-----N-----TN-S		-L---------
	Plasmodium chabaudi cha.	SCM25942	-------C---NT---H-----N-----TN-S		-L---------
	Plasmodium coatneyi	XP_019916682	-----------L-------------------S		T-----V-S--
	Plasmodium cynomolgi str. B	XP_004224726	-----------L-------------------S		T----SV-S--
	Plasmodium fragile	XP_012333281	-----------L-------------------S		T----CV-S--
	Plasmodium gallinaceum	CRG97501	-----------NT------------------S		-A---S-----
	Plasmodium gonderi	GAW83320	-----------I-----------S-------S		T---TSL-S--
	Plasmodium inui San Ant. 1	XP_008814325	-----------L-------------------S		T----SV-S--
	Plasmodium knowlesi str. H	XP_002262005	-----------L-------------------S		T----CA-S--
	Plasmodium malariae	SBS90176	-----------NT---Y--------------S		T----S-----
	Plasmodium ovale curt.	SBS97972	----L------S----H-----------T--T		T--N--V----
	Plasmodium ovale wal.	SBT47208	----L------S----H-----------T--T		T--N-------
	Plasmodium relictum	CRH02361	-----------NT------------------S		-----S-----
	Plasmodium vinckei pet.	EUD74173	----V--C---NT---H-----N-----TN-S		-L---------
	Plasmodium vinckei vin.	XP_008623133	----V--C---NT---H-----N-----TN-S		-L---------
	Plasmodium vivax North Kor.	KMZ97089	-----------L-------------------S		T-----V-S--
	Plasmodium vivax Sal-1	XP_001617041	-----------L-------------------S		T-----V-S--
	Plasmodium yoelii	XP_730731	-------C---NT---H-----N-----TN-S		-L---------
Other Apicomplexa	*Babesia bovis T2Bo*	XP_001610463	NSMSL--G-F--TA--H----GRSI---KG-H		-I-NA---SY-
	Theileria orientalis str. Shi.	XP_009689023	-SSNL--G-F-SLAR-H----G-P-----G-T		-I-NT-V-SN-
	Babesia bigemina	XP_012767979	-S-SL--G-F--TARVH----G-P-RT-KG-H		-I-NA---SY-
	Babesia ovata	GBE59687	-S-NL-AG-F--TA-VH----G-PIRT-KG-H		-I-NA---SY-
	Eimeria maxima	XP_013334335	-S-H---G-F-TTAR-H---AG-T-----G-L		TF-NC-L-LP-
Other Eukarya	*Plasmodiophora brassicae*	CEO98519	-S--V--G---G--RVH--R-GRT-----G-R		-F-N-VTFSE-
	Ostreococcus lucimarinus CCE.	XP_001417659	-GS-V-SG-F-GL-RVH----G-L-----G-T		-Y-N-VAF-E-
	Coccomyxa subellipsoidea C.	XP_005646689	-GSHV-SA---GLARVH----G-M-----G-T		-Y-NH---SP-
	Chlorella variabilis	XP_005846379	-G-HV-SA---TL-RVH-I--GRM-----G-S		-F-NA-V-SA-
	Salpingoeca rosetta	XP_004992494	-NS--AS--F-HT---H--R-GRMM----G-K		-F-N-VC-SM-
	Vitrella brassicaformis CCMP.	CEM13105	-SS-L--A-F-NTARLH----G-T-----G-T		-Y-NC--F-G-
	Mucor ambiguus	GAN07662	-S--V--A---QTIR-H----G-V-----G-T		-FINAVV-ST-
	Mucor circinelloides f. lus.	OAD02260	-S--V--A---QTIR-H----G-M-----G-T		-FINTVV-SA-
	Bifiguratus adelaidae	OZJ06452	EG--V-SG-F-ST--LH----G-A-----G-T		-F-N--LFST-
	Mucor circinelloides f. cir.	EPB90502	-S--V--G---HTIR-H----G-M-----G-T		-FINAV--NS-
	Piromyces finnis	ORX44849	-N--V-S--F-F-IR-H----G-T-----G-T		-F-NDVA-SI-
	Batrachochytrium dendrobatidis	XP_006675044	-S--L-SA-F-GV---H----G-T-R---G-A		-F-NR-VFST-
	Parasitella parasitica	CEP10218	-S--V-SG---QT-R-H----G-V-----G-T		-FINAVM-CS-
	Neocallimastix californiae	ORY57616	-N--V-SC-F-F-IR-H----G-T-----G-T		-F-NDVA-S--
	Spizellomyces punctatus DAOM.	XP_016610349	-G--V-S--F-QTIR-H----G-M-----G-T		-F-ND-VFS--
	Absidia glauca	SAM07248	-G----SG---QTIRLH----G-T-----G-T		-F-N-VLFSK-
	Daphnia magna	KZS21086	-NS-L--G-F-M--RVH----G-T-----G-T		-F-NE--F-A-
	Dendroctonus ponderosae	ENN71702	-NS-V-SA-F-H-IR-H----G-T-----G-S		-F-NEV-F-Q-
	Macrobrachium rosenbergii	AHJ61045	-NS-VMST-F-NTIR-H----G-T-----G-S		-F-NQ-TF-Q-
	Helobdella robusta	XP_009020693	-NS-L--A-F-MVI-------G-A-----G-T		-F-ND--FSY-
	Trichuris suis	KHJ45516	-N----S----MK-R-H--RNG-L--D--G--		-M-NC-MFAQ-

Figure S43. A partial sequence alignment of a Conserved hypothetical protein showing a one amino acid insertion that is specific for members of the "*Laverania*" subgenus.

			450		494
**"*Laverania*" (5/5)**	*Plasmodium falciparum 3D7*	XP_001350142	CTNTSNSSSMIAAGLLAKKAIE	E	FGLKSLPYIKSSLSPGSKTVQK
	Plasmodium falciparum Mal.	ETW47454	----------------------	-	----------------------
	Plasmodium falciparum Pal. Alt.	ETW55559	----------------------	-	----------------------
	Plasmodium falciparum RAJ.	KNC36853	----------------------	-	----------------------
	Plasmodium gaboni	XP_018640196	--------------------V-	-	----------------------
	Plasmodium reichenowi	XP_012764789	--------------------V-	-	------------------A---
	Plasmodium sp. DRC-Itaito	SOV24458	--------------------V-	-	----------------------
	Plasmodium sp. Gor. Cla. G	SOS80527	----------------------	-	----------------------
**Other *Plasmodium* (0/15)**	*Plasmodium berghei ANKA*	XP_676833	-------C------------V-		L-I-PI------------A---
	Plasmodium chabaudi ada.	SCM11150	--------------------V-		L-I-PI------------A---
	Plasmodium chabaudi cha.	XP_745570	--------------------V-		L-I-PI------------A---
	Plasmodium coatneyi	XP_019916717	--------------------V-		N-IE-I----------------
	Plasmodium cynomolgi str. B	XP_004223595	--------------------V-		N-IE-I----------------
	Plasmodium fragile	XP_012338142	----------------------		H-IEPI----------------
	Plasmodium gallinaceum	BAM15611	--------------------V-		M-----------------I---
	Plasmodium gonderi	GAW82154	--------------------V-		N-I-PI------------S---
	Plasmodium inui San Ant. 1	XP_008815728	--------------------V-		H-IEPI----------------
	Plasmodium knowlesi str. H	XP_002259934	--------------------V-		H-IEPI----------------
	Plasmodium malariae	SBS87829	--------------------V-		L-I---------------A---
	Plasmodium ovale curt.	SBS96194	--------------------V-		L-IEP-----------------
	Plasmodium ovale wal.	SBT40377	--------------------V-		L-IEP-----------------
	Plasmodium relictum	CRH01671	--------------------V-		M-----------------I---
	Plasmodium vinckei pet.	EUD71732	--------------------V-		L-I-PI------------A---
	Plasmodium vinckei vin.	XP_008622476	--------------------V-		L-I-PI------------A---
	Plasmodium vivax Mau. I	KMZ91269	--------------------V-		H-IEAI----------------
	Plasmodium yoelii	XP_731148	-------C------------V-		L-I-PI------------A---
**Other Apicomplexa**	*Babesia bovis*	XP_001612299	------P-V-L---M---A-V-		H--EVA----T---------TR
	Babesia ovata	GBE62582	------P-V-L---M---N-V-		H--EVA----T---------TR
	Eimeria acervulina	XP_013251267	------P-V-VG-----R--V-		L--SVA----T------HV--R
	Eimeria necatrix	XP_013433071	------P-V--G-----RR-V-		L--SVA----T------HV--R
	Eimeria praecox	CDI83749	------P-V-VG-----R--V-		L--SVA----T------HV--R
	Eimeria tenella	XP_013234199	------P-V--G-----RR-V-		L--CVA----T------HV--R
	Theileria annulata	XP_953725	------P-V-L-------N-V-		H--SVK----T---------TR
	Theileria orientalis str. Shi.	XP_009689838	------P-V-L-------N-V-		H--SVK----T---------TR
	Theileria parva str. Mug.	XP_766571	------P-V-L-------N-V-		H--SVK----T---------TR
**Other Eukarya**	*Caenorhabditis briggsae*	CAP35039	------P-V-L----V----V-		L--NVQ--V-T------GV-T-
	Caenorhabditis brenneri	EGT30572	------P-V-L----V----V-		L--NVQ--V-T------GV-T-
	Dictyocaulus viviparus	KJH47135	------P-V-L----V----V-		L--S-Q-FT-T------GV-T-
	Caenorhabditis elegans	NP_509898	------P-V-L----V----V-		L--NVQ--V-T------GV-T-
	Caenorhabditis remanei	OZG07085	------P-V-L----V----V-		L--NVQ--V-T------GV-T-
	Caenorhabditis latens	OZG25401	------P-V-L----V----V-		L--TVQ--V-T------GV-T-
	Diploscapter pachys	PAV55654	------P-V-L----V--R---		L--NVQ-FV-T------GV-T-
	Caenorhabditis nigoni	PIC16682	------P-V-L----V----V-		L--NVQ--V-T------GV-T-
	Chlorocebus sabaeus	XP_007967222	------P-V-LG--------VD		A---VM----T------GV-TY
	Drosophila biarmipes	XP_016959725	------P-V-LG--------V-		K--DV-----T------GV-SY
	Paramecium tetraurelia str.	XP_001462506	------PE---G------N-V-		K---VK----TT-----NV-T-
**Prokarya**	*Haematospirillum jordaniae*	WP_066134048	------P-VL-----V------		K---RA-WV----A---QV--D
	Chthonomonas calidirosea	WP_016483851	------P-V-----------V-		L--E-K-WV-T--A---RV-TR
	Noviherbaspirillum massiliense	WP_019141279	------P-VLL---------V-		A---VA-H--T--A---RV-T-
	Pandoraea thiooxydans	WP_047214377	------P-VLL---------V-		A---VA-H--T--A---RV-T-
	Herbaspirillum chloro.	WP_050467920	------P-VLL---------V-		A---VA-H--T--A---RV-T-
	Nitrosospira briensis	WP_074793466	------P-VLL---------V-		K--SVK-H--T--A---RI-TE
	Variovorax sp. RO1	WP_101492754	------P-V-L---------V-		A---VQ-H--T--A---RI-TE
	Pseudomonas punonensis	WP_070883580	------P-V-M---------V-		K--QRK-WV----A----V-TE
	Methanosarcina sp. MTP4	WP_048181799	------P-VL-G--------V-		R--NVK--V-T------RV-TE

Figure S44. Alignment of the Aconitate hydratase protein, showing a one amino acid insertion that is exclusive to the *"Laverania"* subgenus.

Group	Species	Accession	528		575
"Laverania" (5/5)	*Plasmodium falciparum 3D7*	XP_024329193	KYNIKHFNMFQLLLIIKGLYESR	M	LINNKMEELKLIILKEIQLNVYNV
	Plasmodium falciparum 7G8	EUR64886	-----------------------	-	------------------------
	Plasmodium falciparum FCH/4	ETW29259	-----------------------	-	------------------------
	Plasmodium falciparum Mal.	ETW46957	-----------------------	-	------------------------
	Plasmodium falciparum NF135/5.	ETW40247	-----------------------	-	------------------------
	Plasmodium falciparum Pal. Alt.	ETW53726	-----------------------	-	------------------------
	Plasmodium falciparum Tan.	ETW34237	-----------------------	-	------------------------
	Plasmodium falciparum UGT5.1	EWC74087	-----------------------	-	------------------------
	Plasmodium falciparum Vie.	ETW16458	-----------------------	-	------------------------
	Plasmodium gaboni	SOV18668	---------------M-------	-	----------I---N--HK----I
	Plasmodium reichenowi	CDO66581	---------------M-------	-	W-----------------E-----
	Plasmodium sp. DRC-Itaito	SPJ12494	---------------M-------	-	----------F------EE-----
	Plasmodium sp. Gor. Cla. G	SOS80935	-----------------------	-	------------------E-----
Other *Plasmodium* (0/15)	*Plasmodium yoelii yoe.*	EAA19904	----SN--PY-IY--F---C-TK		-E-D-VID--TN-I---NKHI-TM
	Plasmodium yoelii 17X	ETB59468	----SN--AY-IY--F---C-TK		-E-D-VID--TN-I---NKHI-TM
	Plasmodium vivax Mau. I	KMZ90762	----TN-SP--VY--F------K		MR-D-V-Q--KE-T-R-CRD--TM
	Plasmodium vivax Ind. VII	KMZ78321	----TN-SP--VY--F------K		MR-D-V-Q--KE-T-R-CRD--TM
	Plasmodium vivax Bra. I	KMZ83926	----TN-SP--VY--FR-----K		MR-D-V-Q--KE-T-R-CRD--TM
	Plasmodium vinckei vin.	XP_008624049	----SN--AY-IY--F-----TK		-E-D-VAD--INMM---NKHI-TM
	Plasmodium vinckei pet.	EUD73693	----SN--AY-IY--F---C-TK		-E-D-VAD--INMM---NKHI-IM
	Plasmodium relictum	CRH04040	-C-TNN--I--IF--F---S-TN		-R-T-IDD--S-L-EH-RK-I-TI
	Plasmodium ovale wal.	SBT46282	----GN-TS--MY--F---H-T-		-R-D-V-Q--N-CI---NK-I-SI
	Plasmodium ovale curt.	SBS87692	----GN-TS--MY--F---H-TG		-R-D-I-Q--N-FI---NK-I-SI
	Plasmodium malariae	SBT72476	----SN-SI--IY--F-----TK		-C-D-I-Q--AHFV---NKKI-SI
	Plasmodium knowlesi str. H	XP_002260901	----NN-TL--VY-FF------K		FT-D-V-Q--KE-I-R-CRD-HTM
	Plasmodium inui San Ant. 1	XP_008817583	----TN-SL--VY--FR--C--K		-T-D-V-Q--KE-I-R-CRD-HTM
	Plasmodium gonderi	GAW83119	-------RI--FY--F------K		-Q---I----MD-TNV-KK--HCM
	Plasmodium gallinaceum	CRG95616	---ANN-SI--IF--F---S-TK		-Q---I-D--V-L-DH-CK-I-TI
	Plasmodium fragile	XP_012334827	----NN-SL-HVY--F------K		-T-D-V-L--KEVT-R-CRD-HTM
	Plasmodium cynomolgi str. B	XP_004224543	----TN-SL--VY--F------K		FT-D-V-Q--KE-T-R-CRD-HAM
	Plasmodium coatneyi	XP_019917322	----NN-TI--VY--F------K		-A-D-V-Q--EE-T-R-CRD-HSM
	Plasmodium chabaudi cha.	XP_016655652	----SN--AY-IY--F---C-TK		-E-D-VAD--VTMM---NKHI-TM
	Plasmodium chabaudi ada.	SCM08223	----SN--AY-IY--F---C-TK		-E-D-VAD--VTMM---NNHI-TM
	Plasmodium berghei ANKA	XP_022714379	----SN--AY-IY--F---C-TK		-E-D-VVD--MN-I---NKHI-KM

Figure S45. Alignment of a Conserved Plasmodium protein, showing a one amino acid insertion that is exclusive to the *"Laverania"* subgenus.

			576				633
**“*Laverania*” (5/5)**	*Plasmodium falciparum 3D7*	124512084	DVYVYRNLRWTIMKSNKLLPGD	IY	ILTNDMTATDN	N	ICTCETLLIDGTCITDESILTG
	Plasmodium falciparum 7G8	579125018	----------------------	--	-----------	-	----------------------
	Plasmodium falciparum FCH/4	574968324	----------------------	--	-----------	-	----------------------
	Plasmodium falciparum IGH-CR14	910270561	----------------------	--	-----------	-	----------------------
	Plasmodium falciparum Mal.	574988788	----------------------	--	-----------	-	----------------------
	Plasmodium falciparum NF135/5.	574981864	----------------------	--	-----------	-	----------------------
	Plasmodium falciparum NF54	583226789	----------------------	--	-----------	-	----------------------
	Plasmodium falciparum Pal. Alt.	574995978	----------------------	--	-----------	-	----------------------
	Plasmodium falciparum RAJ116	906522595	----------------------	--	-----------	-	----------------------
	Plasmodium falciparum San. Lu.	579337094	----------------------	--	-----------	-	----------------------
	Plasmodium falciparum Tan.	574975249	----------------------	--	-----------	-	----------------------
	Plasmodium falciparum UGT5.1	583216420	----------------------	--	-----------	-	----------------------
	Plasmodium falciparum Vie.	574751269	----------------------	--	-----------	-	----------------------
	Plasmodium gaboni	1084823940	----------N-----M-----	--	------SM---	-	----------------------
	Plasmodium reichenowi	1145258310	----------NV----------	--	-----------	-	----------------------
	Plasmodium sp. Gor. Cla. G2	SOV13227	----------N-----M-----	--	------SM---	-	----------------------
	Plasmodium sp. DRC-Itaito	SPJ09145	----------N-I---M-----	--	-------M---	-	----------------------
**Other *Plasmodium* (0/15)**	*Plasmodium berghei ANKA*	1269283641	N-------Q-K-I---Y-----	--	-----INGN--		--------LE-M----------
	Plasmodium chabaudi ada.	1061790433	N-------Q-K-I---L-----	--	--S--INGN--		--------LE-M----------
	Plasmodium chabaudi cha.	56517841	N-------Q-K-I---L-----	--	--S--INGN--		--------LE-M----------
	Plasmodium coatneyi	1139858563	N-------Q-KVI---M-----	--	--S-ETSGG--		V-------LE-V----------
	Plasmodium cynomolgi str. B	457867401	NL------Q-K-I---M-----	--	--S-ETSGG--		V-------LE-V----------
	Plasmodium fragile	817738457	NI------Q-K-I--SM-----	--	--S-ESSGG--		V-------LE-V----------
	Plasmodium gallinaceum	1103667142	N------MQ-K-I---M-----	--	--A-ELNGN--		---------E-I----------
	Plasmodium gonderi	1194445222	NL------Q-K-I--SM-----	--	--S-ETNGG--		L-------L--V----------
	Plasmodium inui San Ant. 1	672201408	NL------Q-K-I---M-----	--	--S-ETSSG--		V-------LE-V----------
	Plasmodium knowlesi str. H	221052441	N-------Q-K-I---M-----	--	--S-ETNSV--		V-------LE-V----------
	Plasmodium malariae	1037141790	N-------Q-K-I---M-----	--	--S-EIRGS--		--------LE-V----------
	Plasmodium ovale curt.	1036550677	N-------E-KTI---M-----	--	--S-ETS-N-H		V-------LE-I----------
	Plasmodium ovale wal.	1037151973	N------WE-KSI---M-----	--	--S-ETS-N-L		V-------LE-I----------
	Plasmodium relictum	1102622340	N------MQ-K-I---M-----	--	--S-ELNGN--		---------E-I----------
	Plasmodium vinckei pet.	577147141	N-------Q-K-I---F-----	--	--S--INGN--		--------LE-M----------
	Plasmodium vinckei vin.	669202644	N-------Q-K-I---L-----	--	-----INGN--		--------LE-M----------
	Plasmodium vivax Bra. I	901875094	NL------Q-KVI---M-----	--	--S-ETSGG--		V-------LE-V----------
	Plasmodium vivax Ind. VII	901866085	NL------Q-KVI---M-----	--	--S-ETSGG--		V-------LE-V----------
	Plasmodium vivax Mau. I	901883755	NL------Q-KVI---M-----	--	--S-ETSGG--		V-------LE-V----------
	Plasmodium vivax North Kor.	901893113	NL------Q--VI---M-----	--	--S-ETSGG--		V-------LE-V----------
	Plasmodium vivax Sal-1	156095157	NL------Q-KVI---M-----	--	--S-ETSGG--		V-------LE-V----------
	Plasmodium yoelii 17X	564279186	N-------Q-N-I---Y-----	--	--S--INGN--		--------LE-M----------
	Plasmodium yoelii yoe.	23486357	N-------Q-N-I---Y-----	--	--S--INGN--		--------LE-M----------
**Other Apicomplexa**	*Babesia bigemina*	833487515	--L---DGK--TIS-AQ-Y---	-F	L--H-AA-EST		VVRADC-ILS-EVVV-------
	Babesia ovata	1314817370	--A---DGK-ATIS-AH-Y---	-F	L--H-AA-EST		VVRADC-ILS-EVVV-------
	Babesia sp. Xin.	1181629383	KI----DGK-NNIS-TYMY---	-F	L-SH-SSIEAS		VAPADC-ILS-EVVV-------
	Babesia bovis T2Bo	156083909	N-T---DGK-HSIS-TG-Y---	LF	L-SH-PASSAT		-APADC-ILS-EVVV-------
**Other Eukarya**	*Agrilus planipennis*	1069791085	NI-----R--RMLPTDQ-I---		-VSITRSQN--		LVP-DI--LR-S--V---L---
	Amyelois transitella	913303128	NIN----R--RQIM-DQ-----		VVSLTRSVNE-		LVP-DI--LR-S--V---M---
	Aplysia californica	524881999	LIQ----RK-IKIMTDE-----		-VSIVRSQD-K		-VP-DM--LR-P--V---M---
	Aschersonia aleyrodis RCEF.	1024760042	--W---LGQ--EVQTDR-----		LVSVGR-KE-S		GVA-DM--VE--A-VN-AM-S-
	Aspergillus aculeatus ATCC.	1147183006	--W---EKK-QKIT-D------		LMSVNR-KE-G		GVA-DI---E-SA-VN-AM-S-
	Aspergillus calidoustus	972232751	--W---ERK-QEIT-D------		LMSVNR-KE-G		GVA-DI---E-SV-VN-AM-S-
	Baudoinia panamericana UAMH.	627805435	------ENQ--ET--DA-----		LVSVGR-SD-S		GVA-DMV-VE-SA-VN-AM-S-
	Blastomyces dermatitidis AT.	893695220	--W---KNA--EIT-D------		V-SVNR-KE-S		GVA-DI-M-E-SV-VN-AM-S-
	Bombyx mori	512918783	NIN----R--RQIT-DQ-----		-VSLTRSVNE-		LVP-DII-LR-S--V---M---
	Capronia coronata CBS 617.	628274434	-IW---VGK-VQI--D------		LVSVGR-QD-S		GVA-DM---E--V-VN-AM-S-
	Capronia epimyces CBS 606.	628305888	-IW---VRK-VQIT-D------		LVSVGR-QE-S		GVA-DM---E--A-VN-AM-S-
	Cladophialophora bantiana C.	1027055455	-IW---VNK-VEI--D------		LVSVGR-QE-S		GVA-DM---E--A-VN-AM-S-
	Danaus plexippus ple.	1209688854	NIN----R--RQIV-DQ-----		-VSLTRSLN--		LVP-DIV-LR-S--V---M---
	Diaphorina citri	662183385	NMQ----R--KSIL-DD-V---		-ISIGRSTN--		LVP-DV--LR-S--V---M---
	Fusarium pseudograminearum C.	685852615	-MW-F-LGK--EVQ-DQ-I---		LVSVNR-KE-S		GVA-DM--VE--A-VN-AM-S-
	Helicoverpa armigera	1199376969	NIN----R--RQIM-DQ-----		VVSLIRSTN--		LVP-DIV-LR-S--V---M---
	Meliniomyces variabilis F.	1325553101	-IW---KHK-EETQ-D------		LVSVGR-KE-S		GVA-DM--VE-SA-VN-AM-S-
	Metarhizium acridum CQ.	629684491	-IW-F-LGK--EIQTDA-----		LVSV-R-KE-S		GVA-DM--VE--A-VN-AM-S-

Figure S46. A partial sequence alignment of a Cation-transporting ATPase protein showing a one amino acid insertion that is specific for members of the *“Laverania”* subgenus and a potential two amino acid insertion specific for *Hematozoa* (latter is not noted in tables).

Group	Species	Accession	175		222
"*Laverania*" (5/5)	*Plasmodium falciparum 3D7*	XP_001349887	HFQNYVCLIMEYAINGDLKNYIK	NK	FNGFLSEKEAHDLFLQIVKGVYY
	Plasmodium falciparum Vie.	ETW17165	-----------------------	--	-----------------------
	Plasmodium gaboni	XP_018639957	-----------------------	--	-----------------------
	Plasmodium reichenowi	XP_012764531	-----------------------	--	-----------------------
	Plasmodium sp. DRC-Itaito	SPJ11874	-----------------------	--	-----------------------
	Plasmodium sp. Gor. Cla. G2	SOV81096	-----------------------	--	-----------HI----------
Other *Plasmodium* (0/15)	*Plasmodium berghei ANKA*	XP_676213	-------------V-S---K--L	-	N--Y-----TYF-----------
	Plasmodium chabaudi ada.	SCM24612	-------------V-S---K--L	-	N--Y-----TYS-----------
	Plasmodium chabaudi cha.	XP_016654862	-------------V-S---K--L	-	N--Y-----TYS-----------
	Plasmodium coatneyi	XP_019916832	--E-------------------L	-	N--Y----------V--IR----
	Plasmodium cynomolgi str. B	XP_004224764	--E-------------------L	-	N--Y-------N--I--I-----
	Plasmodium fragile	XP_012335227	--E-------------------L	-	N--Y----------V--IR----
	Plasmodium gallinaceum	CRG97541	-------------V--------L	-	N--Y-------------------
	Plasmodium gonderi	GAW83358	----------------------L	-	N--Y----Q-----V--------
	Plasmodium inui San Ant. 1	XP_008814363	--E-------------------L	-	KK-Y----------V--IR----
	Plasmodium knowlesi str. H	XP_002262047	--E-------------------L	-	N--Y----------I--IR----
	Plasmodium malariae	SBT80682	----------------------L	-	N--Y-------------------
	Plasmodium ovale curt.	SBS88089	---------------------ML	-	N--Y-K-----Y-----------
	Plasmodium ovale wal.	SBT47420	---------------------ML	-	N--Y-K-----Y-----------
	Plasmodium relictum	CRH02401	-------------V--------L	-	N--Y-------------------
	Plasmodium vinckei pet.	EUD74210	-------------V-S---K--L	-	N--Y-----TYL-----------
	Plasmodium vinckei vin.	XP_008623094	-------------V-S---K--L	-	N--Y-----TYL-----------
	Plasmodium vivax	XP_001617078	--E-H-----------------L	-	N--Y----------V--IR----
	Plasmodium yoelii 17X	ETB61391	-------------V-S---K--Q	-	N--Y-----TYF-----------
	Plasmodium yoelii yoe.	EAA16511	-------------V-S---K--Q	-	N--Y-----TYF-----------
Other Apicomplexa	*Besnoitia besnoiti*	PFH36478	DNPK-I-F-----T--E-RD-VS		KRTR-K-D--RQF-E--I---H-
	Cystoisospora suis	PHJ21990	DNPT-I-FV----TH-E-RD-VS		KRIK-K-D--RHF-E--I---H-
	Hammondia hammondi	XP_008884342	DNPK-I-F-----T--E-R--VS		KKTR-K-D--RQF-E--I---H-
	Neospora caninum Liv.	XP_003882065	DNPK-I-F-----T--E-R--VS		KKTR-K-D--RQF-E--I---H-
	Toxoplasma gondii ME49	XP_018637513	DNPK-I-F-----T--E-R--VS		KKTR-K-D--RQF-E--I---H-
Other Eukarya	*Vitrella brassicaformis CCMP.*	CEL93112	DHPS-I-F---F-S--E-RE-VA		NRQR-T-D--RHF-Q------H-
	Stentor coeruleus	OMJ73593	DLE-KS-FV---CSG-E--E-VQ		NS-P-----VYRIVI---DAIR-
	Symbiodinium microadriaticum	OLQ02618	DHPDSI-F-----AG-E-RG-VE		EHS--E-D--RTF-K---RA-H-
	Trametes cinnabarina	CDO69976	ETDK-IGI-L---SG-E-FDH-L		AHRY-R--D-CK--S-LIS--W-
	Trichosporon asahii var. asa.	EKD03714	DTAK-IGIVL-F-GG-E-FD--L		A-R--K--D-SR--A-LIS--D-
	Gelatoporia subvermispora B.	EMD32832	ETDK-IGI-L---SG-E-FDH-L		AHRY-R--D-CK--S-LIS--W-
	Mycena chlorophos	GAT60984	ETDK-IGI-I---SG-E-FDF-L		AHRY-R-RD-SK--S-LIS--W-
	Hypsizygus marmoreus	KYQ38522	ETDK-IGI-L---SG-E-FDH-L		AHRY-R-RD-AK--S-LIS--W-
	Fibularhizoctonia sp. CBS 10.	KZP27993	ETDK-IGI-L---SG-E-FDH-L		AHRY-K--D-AK--S-LIS--W-
	Danio rerio	XP_005174173	ESRERIVMV----SG-E-YE--Q		DKQR---D--RHF-R--TSA-H-
	Xiphophorus maculatus	XP_005799928	ENKDKIVIV----SQ---YD--C		DKKN---Y--RHF-R---SA-H-
	Latimeria chalumnae	XP_006006319	ENKDKIIIV----S--E-YDHVN		NRHRIT-N--RNF-R---SA-H-
	Acanthochromis polyacanthus	XP_022058822	ENKDKIVIV----SR---YD--C		DKRNI--R--RHF-R---SA-H-
	Amphiprion ocellaris	XP_023134684	ENKDKIVIV----SR---YD--C		DKRNI--R--RHF-R---SA-H-
	Oryzias melastigma	XP_024124959	ENKDKIVIV----SR---YD--C		DKRK---R--RHF-R---SA-H-
	Oncorhynchus tshawytscha	XP_024300562	ETE-TLY------SG-EVFD-LV		SH-RMK----RAK-R---SA-H-
	Desmodus rotundus	XP_024417764	ETKDMLYIVT-F-Q--EMFD-LT		S--H---N--RKK-W--LSA-E-
	Perkinsus marinus ATCC 50.	XP_002784620	DHPE-I-FV--L-SG-E-RRFVE		RH-P-D-NISRHI-N--A-A-H-
	Spinacia oleracea	XP_021861361	YT---T--V--H-SK--FFD-VS		VRVK-P-D--RPF-R-LIL--E-

Figure S47. Alignment of a Serine/threonine protein kinase, showing a two amino acid insertion that is exclusive to the *"Laverania"* subgenus and a one amino acid insertion exclusive to other *Plasmodium* (latter in not noted in tables).

Group	Species	Accession	548		594
"*Laverania*" (5/5)	*Plasmodium falciparum Dd2*	KOB86259	KEKDIEMNDVIYDCIDNYFHHNE	N	ILIEWFVCIKCLFLVDKNIILNY
	Plasmodium falciparum 3D7	XP_001348369	-----------------------	-	-----------------------
	Plasmodium falciparum FCH/4	ETW29193	-----------------------	-	-----------------------
	Plasmodium falciparum Mal.	ETW50854	-----------------------	-	-----------------------
	Plasmodium falciparum San. Lu.	EUT78700	-----------------------	-	-----------------------
	Plasmodium falciparum Tan.	ETW34120	-----------------------	-	-----------------------
	Plasmodium falciparum Vie.	ETW16289	-----------------------	-	-----------------------
	Plasmodium gaboni	XP_018639275	----------------------D	-	-----------------------
	Plasmodium reichenowi	CDO66681	-----------------------	-	-----------------------
	Plasmodium sp. DRC-Itaito	SOV24938	----------------------D	-	-----------------------
	Plasmodium sp. Gor. Cla. G2	SOV82609	-----------------------	-	-----------------------
Other *Plasmodium* (0/15)	*Plasmodium yoelii yoe.*	EAA20490	T---T-IS-L-HE-V-L--NNS-		-K-----FV--------G-V---
	Plasmodium yoelii 17X	ETB59613	T---T-IS-L-HE-V-L--NNS-		-K-----FV--------G-V---
	Plasmodium vivax North Kor.	KMZ97345	S---T----L--E-V-L--KPED		-GV---LSM--------S-P---
	Plasmodium vivax Mau. I	KMZ90660	S---T----L--E-V-L--KPED		-GV---LSM--------S-P---
	Plasmodium vivax Ind. VII	KMZ78219	S---T----L--E-V-L--KPED		-GV---LSM--------S-P---
	Plasmodium vinckei vin.	XP_008623950	T---T-IG-L-HE-V-L--QN--		-K-----FV--------G-V---
	Plasmodium vinckei pet.	EUD73792	T---T-IG-L-HE-V-L---N--		-K-----FV--------G-V---
	Plasmodium relictum	CRH03940	S---T---NL--ES--L--DIK-		-SL--C--V--------D-V---
	Plasmodium ovale wal.	SBT45618	S---TQ---L--E-V-L---KD-		-C-----F-----------V---
	Plasmodium ovale curt.	SBS87482	S---TQ---L--E-V-L---K--		-C-----F-----------V---
	Plasmodium malariae	SBT72316	S---T----L--ENVNT--QTD-		-CV---I-V----------V---
	Plasmodium knowlesi str. H	XP_002260808	S---T----L--E---L--KPQD		-GV---LSV----------V---
	Plasmodium inui San Ant. 1	XP_008817466	S---T----L--E---L--KPED		-GL---LSV--------S-V---
	Plasmodium gonderi	GAW83019	S--------L--E--NL--KT--		-CV---LSV----------V---
	Plasmodium gallinaceum	CRG95716	N---T----L--ES--L--DIK-		-S------V--------D-V---
	Plasmodium fragile	XP_012333583	S---T----L--E---L--KPQD		-GV---LSV----------V---
	Plasmodium cynomolgi str. B	XP_004224443	S---T----L--E-V-L--KPED		-GV---LSV----------V---
	Plasmodium coatneyi	XP_019917452	S---T----L--E---L--KPGD		-GV---LSL----------V---
	Plasmodium chabaudi cha.	XP_738593	T-----IS-L-HE-V-L---N--		-K-----FV--------G-V---
	Plasmodium chabaudi ada.	SCM07687	T-----IS-L-HE-V-L---N--		-K-----FV--------G-V---
	Plasmodium berghei ANKA	XP_675671	I---T-IG-L-HE-V-L---N--		-K-----FV--------G-V---

Figure S48. Alignment of a Tetratricopeptide repeat family protein, showing a one amino acid insertion that is exclusive to the "*Laverania*" subgenus.

Group	Species	Accession	125		171
"*Laverania*" (5/5)	*Plasmodium falciparum 3D7*	XP_001348359	LYKHNNKLLLSNVLDNERSHNIF	Y	YIINNIFNNIRMFINFYLNIKKV
	Plasmodium falciparum 7G8	EUR64069	-----------------------	-	-----------------------
	Plasmodium falciparum Dd2	KOB87595	-----------------------	-	-----------------------
	Plasmodium falciparum FCH/4	ETW29181	-----------------------	-	-----------------------
	Plasmodium falciparum IGH-CR14	KNG78602	-----------------------	-	-----------------------
	Plasmodium falciparum NF135/5.	ETW40098	-----------------------	-	-----------------------
	Plasmodium falciparum San. Lu.	EUT78687	-----------------------	-	-----------------------
	Plasmodium falciparum UGT5.1	EWC74029	-----------------------	-	-----------------------
	Plasmodium falciparum Vie.	ETW16277	-----------------------	-	-----------------------
	Plasmodium gaboni	SOV18839	-----S---M---------Q--I	-	---T-------------------
	Plasmodium reichenowi	XP_012765251	-----------------------	-	-----------------------
	Plasmodium sp. DRC-Itaito	SPJ12585	------------------P----	-	-----------------------
	Plasmodium sp. Gor. Cla. G	SOS81028	-----------------------	-	-----------------------
Other *Plasmodium* (0/14)	*Plasmodium yoelii yoe.*	EAA15308	----D-SIVAK-IY--DK-F---		---T--YY-LLAYV---I-A---
	Plasmodium yoelii 17X	ETB59599	----D-SIVAK-IY--DK-F---		---T--YY-LLAYV---I-A---
	Plasmodium vivax	SCO69402	----DSS-IQGSNYEQ-KTY-L-		---HA-YS---TL----M-V---
	Plasmodium vinckei vin.	XP_008623960	----DETIVPKIIY--DK-F---		---TS-YY-LLAYV---I-A---
	Plasmodium vinckei pet.	EUD73782	----DETIVPKIIY--DK-F---		---TS-YY-LLAYV---I-A---
	Plasmodium relictum	CRH03950	----E-DQ-IR-YVEEDKPL--Y		NKF---Y--FITYL---M-----
	Plasmodium ovale curt.	SBS97640	----EKSFNFG-DY-EGK-YK--		-L-S--Y--VLT-----I-V---
	Plasmodium malariae	SBS89654	----EKN-FFG-NY-D-KPY---		---S--Y--VIT-----I-V---
	Plasmodium knowlesi str. H	SBO22583	----E-N-IQG-NYEE-K-Y-L-		-L-HA-Y----TL----M-V---
	Plasmodium inui San Ant. 1	XP_008817476	----E-N-IQGSNYEEDK-Y-L-		-L-HA-YS---TL----M-V---
	Plasmodium gonderi	GAW83029	----EKN-Q-G-HY-E-KPY---		---SSVY---LAL----M-V---
	Plasmodium gallinaceum	CRG95706	----E-NQFIE-GFEEDK-F-MY		NK----Y--FLTYL---M-V---
	Plasmodium fragile	XP_012333593	----E-N-IQG-NYED-K-Y-L-		---HA-Y----TL----M-V---
	Plasmodium coatneyi	XP_019917181	----E-N-TQGSNYEE-K-Y-L-		---HA-Y----TL----M-V---
	Plasmodium chabaudi cha.	SCM02576	----DETIVPKIIY--DK-F---		---TS-YY-LLAYV---I-A---
	Plasmodium chabaudi ada.	SCM10912	----DETIVPKIIY--DK-F---		---TS-YY-LLAYV---I-A---
	Plasmodium berghei ANKA	XP_022714334	----D-SIVAK-IY--DK-F---		---T--YY-LLAYV---I-A--I

Figure S49. Alignment of a Thioredoxin-like protein, showing a one amino acid insertion that is exclusive to the *"Laverania"* subgenus. CSI region is not conserved in *P. cynomolgi*.

Group	Species	Accession	2635		2685
***"Laverania"* (2/5)**	*Plasmodium falciparum 3D7*	XP_001351366	LILRSLHINPQQTKILLQPNKNIVT	T	QPHHIWPSFNNNQWIHLEVQLKDLI
	Plasmodium falciparum 7G8	EUR46738	-------------------------	-	-------------------------
	Plasmodium falciparum Dd2	KOB84899	-------------------------	-	-------------------------
	Plasmodium falciparum FCH/4	ETW32191	-------------------------	-	-------------------------
	Plasmodium falciparum HB3	KOB58992	-------------------------	-	-------------------------
	Plasmodium falciparum IGH-CR14	KNG77101	-------------------------	-	-------------------------
	Plasmodium falciparum Mal.	ETW51282	-------------------------	-	-------------------------
	Plasmodium falciparum Pal. Alt.	ETW57538	-------------------------	-	-------------------------
	Plasmodium falciparum RAJ116	KNC35375	-------------------------	-	-------------------------
	Plasmodium falciparum San. Lu.	EUT91632	-------------------------	-	-------------------------
	Plasmodium falciparum Tan.	ETW32981	-------------------------	-	-------------------------
	Plasmodium falciparum UGT5.1	EWC78565	-------------------------	-	-------------------------
	Plasmodium falciparum Vie.	ETW20357	-------------------------	-	-------------------------
	Plasmodium reichenowi	XP_019970770	-------------------------	-	-------------------------
	Plasmodium sp. DRC-Itaito	SPJ08735.1	-------------------------		-------------------------
	Plasmodium sp. Gor. Cla. G2	SOV11188.1	-----------------------I-		-------------------------
	Plasmodium gaboni	XP_018643283	-----------------------I-		-------------------------
**Other *Plasmodium* (0/15)**	*Plasmodium berghei ANKA*	XP_022714222	-----------E-----------I-		---------------N---------
	Plasmodium chabaudi ada.	SCM06549	-----------E-----------I-		---------------N---------
	Plasmodium chabaudi cha.	SCM03516	-----------E-----------I-		---------------N---------
	Plasmodium coatneyi	XP_019912805	-------------------------		---------------N---------
	Plasmodium cynomolgi str. B	XP_004220850	-------------------------		---------------N---------
	Plasmodium fragile	XP_012335046	-------------------------		---------------N---------
	Plasmodium gallinaceum	CRG98126	-----------------------I-		-------------------------
	Plasmodium gonderi	GAW79234	---------------------S-I-		-------T-------N---------
	Plasmodium inui San Ant. 1	XP_008818570	-------------------------		---------------N---------
	Plasmodium knowlesi str. H	XP_002261014	-------------------------		---------------N---------
	Plasmodium malariae	SBS81878	------------S----------I-		---------------N---------
	Plasmodium ovale curt.	SBS80186	-----------E-----------I-		---------------T---------
	Plasmodium ovale wal.	SBT31663	-----------E-----------T-		--------L------T---------
	Plasmodium relictum	CRG98542	-----------------------I-		-------------------------
	Plasmodium vinckei pet.	EUD71892	-----------E-----------I-		---------------N---------
	Plasmodium vinckei vin.	XP_008625065	-----------E-----------I-		---------------N---------
	Plasmodium vivax Ind. VII	KMZ82613	-------------------------		---------------N---------
	Plasmodium vivax North Kor.	KNA01897	-------------------------		---------------N---------
	Plasmodium vivax Sal-1	XP_001613367	-------------------------		---------------N---------
	Plasmodium yoelii 17X	ETB59894	-----------E-----------I-		---------------N---------
**Other Apicomplexa**	*Toxoplasma gondii ME49*	XP_002368068	-L--AM-V-TER---I-R---TT--		-S------LTDEE---V--A-----
	Neospora caninum Liv.	XP_003883442	-L--AM-V-TER---I-R---TT--		-S------LTDEE---V--A-----
	Hammondia hammondi	XP_008885662	-L--AM-V-TER---I-R---TT--		-S------LTDEE---V--A-----
	Eimeria acervulina	XP_013246946	-L--A-QV-TER--VV-K---STI-		--------LTDEE---V--T-----
	Besnoitia besnoiti	PFH38612	-L--AM-V-TES--VI-K---TT--		-S------LTDEE---V--A-----
	Cystoisospora suis	PHJ21003	-L--AM-V-TER--VI-K---ST--		-Q-----TLTDEE---V--T-----
	Cryptosporidium parvum Iowa II	XP_626847	-----M----ER--VI-K-----I-		MHN-----LTDEE-ANV--AM--I-
	Cryptosporidium hominis	CUV05213	-----M----ER--VI-K-----I-		MHN-----LTDEE-ASV--AM--I-
**Other Eukarya**	*Symbiodinium microadriaticum*	OLP96264	----A--V--DR--VI-K-D-TT--		----V---LTDDE---V--------
	Gonium pectorale	KXZ48777	----A--V--EKARMI-R-D-S---		----V---LTDE---KV--A-----
	Chlamydomonas reinhardtii	XP_001689471	----A--V--EKARMI-R-D-S---		----V---LTDE---KV--A-----
	Volvox carteri f. nag.	XP_002956689	----A--V--EKARVI-R-D-S---		----V---LTDE---KV--A-----
	Acanthamoeba castellanii str.	XP_004344089	-------V-HEK-RVI-K-D-TV--		--------L-DE---KV--S-----
	Reticulomyxa filosa	ETO20865	--F-A--V--DK--MI-K--RSVI-		-S------LTDEE--NV-I------
	Verticillium longisporum	CRK42910	----A--V--DK--LI-R-D-TVI-		-D-----TLSDED-VKV----R---
	Histoplasma capsulatum H143	EER43075	----A--V--EK-----R-D-TVI-		-E-----TLSDED--KV----R---
	Trichoderma parareesei	OTA05502	----A--V--DK--LI-R-D-SVI-		LE------LSDEE--KV-T--R---
	Rasamsonia emersonii CBS 393.	XP_013324817	----A--V--DK---I-R-D-SVI-		-E------LSDEE--KV----R---
	Trichoderma reesei QM6a	XP_006965408	----A--V--DK--LI-R-D-TVI-		LE------LSDEE--KV-T--R---
	Histoplasma capsulatum H88	EGC46008	----A--V--EK-----R-D-TVI-		-E-----TLSDED--KV----R---
	Cordyceps confragosa RCEF 10.	OAA78370	----A--V--DK--LI-R-D-T-I-		LE------LTDEE--KV-T--R---
	Cordyceps militaris CM01	XP_006666823	----A--V--DK--LI-R-D-T-I-		LE------LTDEE--KV-T--R---
	Histoplasma capsulatum NAm1	XP_001540722	----A--V--EK-----R-D-TVI-		-E-----TLSDED--KV----R---
	Cordyceps brongniartii RCEF.	OAA50840	----A--V--DK--LI-R-D-T-I-		LE------LTDEE--KV-T--R---
	Histoplasma capsulatum G186AR	EEH11039	----A--V--EK-----R-D-TVI-		-E-----TLSDED--KV----R---

Figure S50. A partial sequence alignment of the Pre-mRNA-processing-splicing factor 8 protein showing a one amino acid insertion that is specific for members of the *"Laverania"* genus, however not seen in *P. gaboni, Plasmodium sp. DRC-Itaito* and *Plasmodium sp. Gorilla Clade G2.*

Group	Species	Accession	44		92
**"*Vinckeia*" (4/4)**	*Plasmodium berghei ANKA*	XP_022714285	GRIETTLTKAKELQGYAEELIYLA	K	KKNNSENSLLVESILRTAQGRRKL
	Plasmodium chabaudi ada.	SCM07120	------------------------	-	------------------------
	Plasmodium chabaudi cha.	XP_745795	------------------------	-	------------------------
	Plasmodium vinckei pet.	EUD73883	------------------------	-	------------------------
	Plasmodium vinckei vin.	XP_008623859	------------------------	-	------------------------
	Plasmodium yoelii 17X	ETB59742	------------------------	-	-------------M---P------
**Other *Plasmodium* (0/16)**	*Plasmodium reichenowi*	XP_012765357.1	------------------------		--D-V--N-K---M--------R-
	Plasmodium coatneyi	XP_019917193	------------------------		----A--N-K---M----------
	Plasmodium cynomolgi str. B	XP_004224350	------------------------		----V--N-K---M----------
	Plasmodium falciparum 3D7	XP_001348463	------------------------		--D-V--N-K---M--------R-
	Plasmodium falciparum HB3	KOB58754	------------------------		--D-V--N-K---M--------R-
	Plasmodium falciparum IGH-CR14	KNG74576	------------------------		--D-V--N-K---M--------R-
	Plasmodium falciparum Mal.	ETW46848	------------------------		--D-V--N-K---M--------R-
	Plasmodium falciparum UGT5.1	EWC73920	------------------------		--D-V--N-K---M--------R-
	Plasmodium fragile	XP_012338668	------------------------		----V--N-K---M----------
	Plasmodium gaboni	XP_018639373	------------------------		--D-V--N-K---M--------R-
	Plasmodium gallinaceum	CRG95814	------------------------		--D-V--N-K---M--------R-
	Plasmodium gonderi	GAW82925	------------------------		----A--N-K---M----------
	Plasmodium inui San Ant. 1	XP_008816891	--------------------V---		----V--N-K---M----------
	Plasmodium knowlesi str. H	XP_002260713	------------------------		----V--N-K---M----------
	Plasmodium malariae	SBS89476	------------------------		--D-VQ-N-K---M----------
	Plasmodium ovale curt.	SBS87294	------------------------		--DSE--K-K---M----------
	Plasmodium ovale wal.	SBT30446	------------------------		--D-V--K-K---M----------
	Plasmodium relictum	CRH03842	----------------------F-		---TI--N-K---M--------R-
	Plasmodium sp. DRC-Itaito	SPJ12688	------------------------		--D-V--N-K---M----------
	Plasmodium sp. Gor. Cla. G	SOS81136	------------------------		--D-V--N-K---M--------R-
	Plasmodium vivax Ind. VII	KMZ78540	------------------------		----A--N-K---M----------
	Plasmodium vivax Mau. I	KMZ90929	------------------------		----A--N-K---M----------
**Other Apicomplexa**	*Babesia bigemina*	XP_012766702	----L--PR-----Q----VVFH-		--DTP-SD-I---M--SPEA-Q--
	Babesia bovis T2Bo	XP_001610966	--V-L--PR-----Q----V-FH-		--DTP-SD-V---M---PEA-HQ-
	Babesia microti str. RI	XP_012649295	-------ER---I-Q------IHS		RNDCK-SDII---LITS-EA-SA-
	Babesia ovata	GBE60311	----L--PR-----Q----VVFH-		--DTP-SD-----M--SPEA-Q--
	Babesia sp. Xin.	ORM39458	--V-V--PR-----Q----VVFH-		--DTP-SD-V---M---PEA-HE-
	Theileria annulata	XP_954101	----L--PR-----Q-I--I-FH-		-QDKM-SDI-----I--SEC-SL-
	Theileria equi	XP_004833195	----L--PR-----Q----I-FH-		-RDCR-SD-I---V---PES-SL-
	Theileria orientalis str. Shi.	XP_009689506	----V--PR-----Q-I-----H-		-RDTH-SD-I---VI--PES-SL-
	Besnoitia besnoiti	PFH35414	D-L-L--PR-Q---Q------HF-		-Q-TP-S--I---MIF-PAA----
	Cystoisospora suis	PHJ19724	D-L-L--PR-R---Q------HF-		-Q-TP-S--IT--LIV-PAA----
	Hammondia hammondi	XP_008886134	D-L-L--SR-H---Q-----VHF-		-Q-TP-S--I---MIF-PAA----
	Neospora caninum Liv.	XP_003880187	D-L-L--PR-Q---Q-----VHF-		-Q-TP-S--I---MIF-PAA----
	Toxoplasma gondii ME49	XP_002371147	D-L-L--PR-H---Q-----VHF-		-Q-TP-S--I---MIF-PAA----
**Other Eukarya**	*Vitrella brassicaformis CCMP.*	CEM12687	Q------S------Q----IVF--		--DTP-SD-K---M---PAA--I-
	Thermus aquaticus	WP_003043956	---T--VP-----T-FVDH--H--		-RGDLHARR--LRD-QDVKLV---
	Meiothermus silvanus	WP_013159253	---T--VP-----R-FV-H--TV-		--GDLSARR--LRD-HDPALV---
	Oceanithermus profundus	WP_013458222	---R--IP-----RRFV-P--TK-		-RGDLSARRQ-IREIHDLDVV-R-
	Marinithermus hydrothermalis	WP_013704479	---T--VP-----R-FV-H--NV-		-RGDLAARRR-IRDIHDLTVV---
	Thermus sp. CCB_US3_UF1	WP_014514900	---T---P----IT-FVDH--H--		-RGDLHARR--LRD-QDVKLV---
	Blastomonas sp. CCH1-A6	WP_066282224	EQ-K---P--R--RP-V-K--T--		-RGGLS-RR-AMAR-MDDAQLV--
	Thalassiosira pseudonana CCM.	XP_002290797	E------P-----RHL-DKVVGY-		--GDVHAKQ-ALRVV-EKPVVT--
	Ignavibacteriales bac. UT.	OQY77150	K------A-----RSF--P--TK-		R-GTLHDQRI-M-V--NKEAAKE-
	Candidatus Paraburkholderia ca.	KMY86325	EV-K---P-----RKVV-P--T-G		--PSLA-RR--FNR--DRDSVT--
	Plasmodiophora brassicae	CEP04002	-------P--RQ-SKV-DR--TY-		-RGTAGSRMQATR-V-STDMID--
	Mucispirillum schaedleri	WP_023276846	-K-----D---V-R-VV-P--T--		--GDVPARR-ALKK-PH-VSV---

Figure S51. Alignment of the Mitochondrial ribosomal protein L17-2 precursor, showing a one amino acid insertion that is exclusive to the "*Vinckeia*" subgenus. Expanded alignment of Figure 5 (C).

Group	Species	Accession	119		156
**"*Vinckeia*" (4/4)**	*Plasmodium berghei str. ANKA*	XP_678388	KFYNASTSELYG	N	NIQSQCHNENTPFNPVSPYGTAKLY
	Plasmodium chabaudi ada.	SCM24880	------------	-	T--TE-Q-------------I----
	Plasmodium chabaudi cha.	XP_745006	------------	-	T--TE-Q-------------I----
	Plasmodium vinckei pet.	EUD74311	------------	-	-T----Q-------------I----
	Plasmodium vinckei vin.	XP_008622991	------------	-	------Q-------------I----
	Plasmodium yoelii 17X	ETB61521	------------	-	-------------S------I----
	Plasmodium yoelii yoe. 17XNL	XP_727120	------------	-	-------------S------I----
**Other *Plasmodium* (0/16)**	*Plasmodium sp. Gor. Cla. G2*	SOV13605.1	----------F-		KV-CSIQ--S---Y-----AI----
	Plasmodium sp. DRC-Itaito	SPJ09869.1	----------F-		KV-CSIQ------Y-----AI----
	Plasmodium coatneyi	XP_019916663	------------		CCSEDIQ--S---------AI----
	Plasmodium cynomolgi str. B	XP_004224863	------------		CSTDDIQ--C---------AI----
	Plasmodium falciparum 3D7	XP_001349387	----------F-		KV-CPIQ------Y-----AI----
	Plasmodium falciparum San. Lu.	EUT87205	----------F-		KV-CPIQ------Y-----AI----
	Plasmodium fragile	XP_012337916	------------		CSTDDMQH-C---------AI----
	Plasmodium gaboni	XP_018642233	----------F-		KV-CSIQ--S---Y-----AI----
	Plasmodium gallinaceum	CRG97646	----------F-		KVKCIPQK-D---Y-----AI----
	Plasmodium gonderi	GAW83460	----------F-		K-K-NIQ--Y---------AI----
	Plasmodium inui San Ant. 1	XP_008814468	------------		CCTDDIQQ-C---------AI----
	Plasmodium knowlesi str. H	XP_002262150	------------		CCNDHVQ--S---------AI----
	Plasmodium malariae	SBS90518	----------F-		KVKAAIQD-----Y-----AI----
	Plasmodium ovale curt.	SBS88266	------------		KTRCNSQ--D---F-----AI----
	Plasmodium ovale wal.	SBT48008	------------		KARCNSQ--D---F-----AI----
	Plasmodium reichenowi	XP_012762525	----------F-		KV-CSIQ------Y-----AI----
	Plasmodium relictum	CRH02506	----------F-		KVK-IPQ--D---Y-----AI----
	Plasmodium vivax Ind. VII	KMZ78001	----------F-		SSTDDRQH-S---------AI----
	Plasmodium vivax Sal-1	XP_001617179	----------F-		SSTDDRQH-S---------AI----
**Other Apicomplexa**	*Besnoitia besnoiti*	1261479511	RV-Q------F-		RV-ETPQT-M---Y-R----IS---
	Cyclospora cayetanensis	1249155048	FQ--S--MF-		SSDAELQ--G---K-C----IS---
	Cystoisospora suis	1268230518	RL-Q------F-		RV-ETPQT-A---Y-R----I----
	Eimeria mitis	557245717	RVFQ--S--MF-		SSTVELQ--D---K-C----VS---
	Eimeria necatrix	921118124	RVFQ--S--MF-		SSDWDLQ--D---K-C----VS---
	Eimeria praecox	557143732	RVFQ--S--MF-		SSTVELQ--E---K-C----IS---
	Eimeria tenella	916414414	RVFQ--S--MF-		SSDWDLQ--D---K-C----VS---
	Hammondia hammondi	675131274	RI-Q------F-		RV-DSPQC-T---Y-R----I----
	Neospora caninum Liv.	401404738	RI-Q------F-		RV-ETPQS-T---Y-R----I----
	Toxoplasma gondii ME49	237834497	RI-Q------F-		RV-ESPQS-T---Y-R----I----
**Prokarya**	*Helicobacter ailurogastricus*	WP_053940870	R--Q------F-		EVLQTPQ--------R---AV----
	Helicobacter bizzozeronii CC.	CCF80029	R--Q--------		EVLQTPQ--------R---AV----
	Helicobacter cetorum	WP_014658575	R--Q--------		EVLETPQ--------R---AV--M-
	Helicobacter cetorum MIT 00.	AFI03331	R--Q--------		EVLETPQ--------R---AV--M-
	Helicobacter felis	WP_013469962	R--Q--------		EVLETPQ--------R---AV----
	Helicobacter heilmannii	WP_053828698	R--Q--------		AVLQTPQ--------R---AV----
	Helicobacter pylori 908	ADN79178	R--Q--------		EVLETPQ--------R---AV--M-
	Helicobacter pylori FD430	EQL50559	R--Q--------		EVLETPQ--------R---AV--M-
	Helicobacter pylori SouthAf.	EQD89564	R--Q--------		EVLETPQ--------R---AV--M-
	Helicobacter pylori XZ274	AFJ82229	R--Q--------		EVLETPQ--------R---AV--M-
	Helicobacter suis	WP_006564079	R--Q--------		EVLETPQ--------R---AV----
	Methanobrevibacter arbori.	WP_080460266	---Q--------		LV-ETPQ--L---Y-R----V----
	Methanobrevibacter cuticularis	WP_084270686	---Q--------		LV-ETPQT-S---Y-R----V----
	Pseudomonas aeruginosa	WP_033974977	R--Q-----MF-		L--AE-QD-----Y-R----V----
	Rhizobium alamii	WP_037100618	R--Q--------		KVVETPQK-T---Y-R----V----
	Rhizobium sp. CF142	WP_007813255	R--Q--------		KVVETPQK-T---Y-R----V----
	Epulopiscium sp. Nuni2H.	OOB79503	FYQASTSELFG-		LPETAPQ--L---Y-K----V----
	Epulopiscium sp. SCG-B11WGA.	ONI42438	FYQASTSELFG-		LPGTAPQ--K---Y-K----V----

Figure S52. A partial sequence alignment of a conserved region of the GDP-mannose 4, 6 dehydratase protein showing a one amino acid insertion that is specific for members of the "*Vinckeia*" subgenus.

Group	Species	Accession	385		431
"*Vinckei*" (4/4)	*Plasmodium berghei ANKA*	XP_022714317	IKKEFLLNCEVIINIVNCIISTI	T	NDDKNKIFYIHLNANQYKIVSNI
	Plasmodium chabaudi ada.	SCM07539	-----------------------	P	--NE-------------------
	Plasmodium chabaudi cha.	XP_736985	-----------------------	P	--NE-------------------
	Plasmodium vinckei pet.	EUD73817	--N--------------------	P	---E-------------------
	Plasmodium vinckei vin.	XP_008623928	--N--------------------	P	--NE-------------------
	Plasmodium yoelii 17X	ETB59646	-----------------------	A	-E---------------------
	Plasmodium yoelii yoe.	EAA21217	-----------------------	A	-E---------------------
Other *Plasmodium* (0/16)	*Plasmodium vivax Mau. I*	KMZ90634	--DK------A---V----LC-T		-EERE--Y-T--------TI-DV
	Plasmodium vivax Ind. VII	KMZ78191	--DK------A---V----LC-T		-EERE--Y-T--------TI-DV
	Plasmodium vivax Bra. I	KMZ83797	--DK------A---V----LC-T		-EERE--Y-T--------TI-DV
	Plasmodium sp. Gor. Cla. G	SOV82650	--NQ----S-A---LI---FY--		KEER--F--TY--------I-DM
	Plasmodium sp. DRC-Itaito	SOV24963	--NK----S-A---LI---FY--		K-ER--F--TY--------I-DM
	Plasmodium relictum	CRH03914	--TQ----------LI---LY--		-EEQ-R-Y--Y--G-----I-D-
	Plasmodium reichenowi	XP_019969949	--NQ----S-A---LI---FY--		KEER--F--TY--------I-DM
	Plasmodium ovale wal.	SBT45462	--SQ-----------I---FY--		SEE-K--Y--Y----L---I-D-
	Plasmodium ovale curt.	SBS87429	--SQ-----------I---FY--		SEE-K--Y--Y----L---I-D-
	Plasmodium malariae	SBS89591	--ND------AL---I-L-F---		DE----FY-----------I-D-
	Plasmodium knowlesi str. H	XP_002260782	--EN------A---V----LC--		-EE-E--Y-T--------TI-D-
	Plasmodium inui San Ant. 1	XP_008817441	--DK------AM--M----L---		-EE-E--Y-SK-------TI-D-
	Plasmodium gonderi	GAW82993	--DK------AM---L---LCM-		-N--D--Y-----------I-D-
	Plasmodium gallinaceum	CRG95742	--IH------I---LI---LY--		-E-Q---Y--Y--G-----I-D-
	Plasmodium gaboni	XP_018639300	--NK----S-A---LI---FY--		K-ER--F--TY--------I-DM
	Plasmodium fragile	XP_012333559	--DK------A---V-----C--		-EE-E--Y-T-------RTI-D-
	Plasmodium falciparum Vie.	ETW16314	--NQ----S-A---LI---FY--		KEER--F--TY--------I-DM
	Plasmodium falciparum San. Lu.	EUT78728	--NQ----S-A---LI---FY--		KEER--F--TY--------I-DM
	Plasmodium falciparum Mal.	ETW46201	--NQ----S-A---LI---FY--		KEER--F--TY--------I-DM
	Plasmodium falciparum 7G8	EUR64105	--NQ----S-A---LI---FY--		KEER--F--TY--------I-DM
	Plasmodium falciparum 3D7	XP_001348394	--NQ----S-A---LI---FY--		KEER--F--TY--------I-DM
	Plasmodium cynomolgi str. B	XP_004224417	--DK------A---V----LC--		-EE-E--Y-T-------RTI-D-
	Plasmodium coatneyi	XP_019917421	--DK------A---V----LC--		SEE-E--Y-T--------TI-D-

Figure S53. Alignment of the 14-3-3 protein, showing a one amino acid insertion that is exclusive to the "*Vinckeia*" subgenus.

Group	Species	Accession	380		429
***"Vinckeia"* (4/4)**	*Plasmodium berghei ANKA*	XP_679884	VGGNPFFKNSRLIDINEIDNNPEIN		DNFVNSINKKNMQFETVSALGGLMQ
	Plasmodium chabaudi ada.	SCM09609	-------------------------		-----------I-------------
	Plasmodium chabaudi cha.	XP_733340	-------------------------		-----------I-------------
	Plasmodium vinckei pet.	EUD71381	----T--------------------		---I-------I-------------
	Plasmodium vinckei vin.	XP_008622058	----T--------------------		---I-------I-----------L-
	Plasmodium yoelii 17X	ETB56188	-------E-----------------		-----------V-------------
**Other *Plasmodium* (0/16)**	*Plasmodium vivax North Kor.*	KMZ97910	---DA--QD-K----SGDNPERDK-	Y	SEVLT-LHNGKPE-----L---FL-
	Plasmodium vivax Mau. I	KMZ91699	---DA--QD-K----SGDNPERDK-	Y	SEVLT-LHNGKPE-----L---FL-
	Plasmodium vivax Ind. VII	KMZ78852	---DA--QD-K----SGDNPERDK-	Y	SEVLT-LHNGKPE-----L---FL-
	Plasmodium sp. Gor. Cla. G2	SOV83417	---DA--H--K---L-DTNE-IQND	H	KQ-L-TL-NDKVE---I-L----L-
	Plasmodium sp. DRC-Itaito	SOV25228	---DA--N--K---L-DTNQ-IQD-	Y	KQ-L-TL-NDKA----I-L----L-
	Plasmodium relictum	CRH01418	---DA-----Q---L--D-MISKN-	Y	-D-LQ-L-NNKIE-----L----L-
	Plasmodium reichenowi	CDO66972	---DA--P--N---L-DTNE-IQND	Y	KQ-L-TL-NDKVE---I-L----L-
	Plasmodium ovale wal.	SBT42956	---DN--E-DK----HQK-FISDN-	E	-Y-LK-L-E-DT------L----L-
	Plasmodium ovale curt.	SBS97061	---DK--E-DK----QKK-FISDN-	E	-Y-LK-L-E-DT------L----L-
	Plasmodium malariae	SBS88734	---DL--QD-K-VHA--HTI-S-D-	Y	-K-I----NNTTE--K--L----L-
	Plasmodium knowlesi str. H	XP_002260365	---DA--QD-K---TSDDNPDLDK-	Y	NQLLT-LQQDKAD--P--L---FL-
	Plasmodium inui San Ant. 1	XP_008814116	---DA--Q--K---TSDENSDLDK-	Y	HELLT-LHHDKAE-----L----L-
	Plasmodium gonderi	GAW82579	---DI--QD-K-----DENSDSSK-	Y	EQIFR--HPNKE------L----L-
	Plasmodium gallinaceum	CRG94703	---DV-----Q---LDDN-MISKN-	N	-D-LK-L-NNKVE-----L----L-
	Plasmodium gaboni	XP_018639563	---DA--N--K---L-DTNQ-IQD-	Y	KQ-L-TL-NDKA----I-L----L-
	Plasmodium fragile	XP_012336488	---DA--QD-K---TSDDNPDLDK-	Y	HELLT-L-HDKAE-----L---VL-
	Plasmodium falciparum Pal. Alt.	ETW54166	---DA--P--N---L-DTNE-IQND	Y	KQ-L-TL-NDKVE---I-L----L-
	Plasmodium falciparum NF.	ETW39754	---DA--P--N---L-DTNE-IQND	Y	KQ-L-TL-NDKVE---I-L----L-
	Plasmodium falciparum FCH.	ETW29033	---DA--P--N---L-DTNE-IQND	Y	KQ-L-TL-NDKVE---I-L----L-
	Plasmodium falciparum Dd2	KOB85498	---DA--P--N---L-DTNE-IQND	Y	KQ-L-TL-NDKVE---I-L----L-
	Plasmodium falciparum 7G8	EUR62964	---DA--P--N---L-DTNE-IQND	Y	KQ-L-TL-NDKVE---I-L----L-
	Plasmodium falciparum 3D7	XP_001348657	---DA--P--N---L-DTNE-IQND	Y	KQ-L-TL-NDKVE---I-L----L-
	Plasmodium cynomolgi str. B	XP_004224025	---DA--QD-K---TSDDNPDLDK-	Y	HELLT-LHHDKAE-----L---FL-
	Plasmodium coatneyi	XP_019916129	---DA--QD-K---TSDDNPDLDK-	Y	NQLLT-LHQDKA------L---FL-

Figure S54. Alignment of a Conserved *Plasmodium* protein, showing a one amino acid deletion that is exclusive to the *"Vinckeia"* subgenus.

	Species	Accession	113		167
**“*Vinckeia*” (4/4)**	*Plasmodium berghei ANKA*	XP_022714261	YYVKFLNNAIQKIKVTPIINKIYNNI	YNNI	ILKYTANIHIFISPIYERMNNESLF
	Plasmodium chabaudi ada.	SCM06855	--------------A-----------	----	F--------V--------I------
	Plasmodium chabaudi cha.	XP_016655521	--------------A-----------	----	F--------V--------I------
	Plasmodium vinckei pet.	EUD71948	--------------A-----------	----	-----T------------I---N--
	Plasmodium vinckei vin.	XP_008625121	--------------T-----------	----	-----T---V--------I---N--
	Plasmodium yoelii	XP_022813271	--------------------------	----	-------------------------
**Other *Plasmodium* (0/16)**	*Plasmodium coatneyi*	XP_019913105	----I--SLME-VRNV-V--YL----		V-R-RV---V-V--L---I---K--
	Plasmodium cynomolgi str. B	XP_004224302	----I--SLME-VRDV-L--YL----		V-R-RV---V-V--L---I---K--
	Plasmodium falciparum 3D7	XP_001348514	--LN-V-KFMK---NIS-V-YFT-I-		NS--KI------------I--QV-L
	Plasmodium falciparum HB3	KOB58523	--LN-V-KFMK---NIS-V-YFT-I-		NS--KI------------I--QV-L
	Plasmodium falciparum NF54	EWC85722	--LN-V-KFMK---NIS-V-YFT-I-		NS--KI------------I--QV-L
	Plasmodium fragile	XP_012334343	----II-SLME-VRDV-L--YV--K-		V---RV---V-V--L-------K--
	Plasmodium gaboni	XP_018639421	--LN-V-KFMKN--QIS-V-Y-T-I-		NS-FKI------------I--QE-L
	Plasmodium gallinaceum	CRG95862	--F-P-S-I-K-F-EI-V--YL-K--		FF--RV------------I---K--
	Plasmodium gonderi	GAW82877	F---I--SL---F-DV--V-YV----		--R-RT---V-V--L---I---E--
	Plasmodium inui San Ant. 1	XP_008816940	----I--SLME-VRNV----YL----		V-R-RV---V-V--L-D-I---K--
	Plasmodium knowlesi str. H	XP_002260653	----I--SLME-VRDV-L--YL-K--		V-R-RV---V-V--L-------K--
	Plasmodium malariae	SBS89349	--FN---DFV---MGI----YL----		F---RI---T--------I---E--
	Plasmodium ovale curt.	SBS87182	--MN--S-IVK---HV----YV---F		--RHCV------------I---N--
	Plasmodium ovale wal.	SBT45245	--MN--S-IV----QV----Y----F		---HCV------------I---N--
	Plasmodium reichenowi	XP_012765405	--LN-I-KFMK---NIS-V-Y-T-I-		NS--KI------------I--QE-L
	Plasmodium relictum	CRH03793	--LNP--YIKK-CMEI-L--YF----		F---RI-V----------I---K-L
	Plasmodium sp. DRC-Itaito	SOV25082	--LN-V-KFMKN--QIS-V-Y-T-I-		NS-FKI------------I--QE-L
	Plasmodium sp. Gor. Cla. G	SOV83134	--LN-V--FMK---KIS-V-Y-T-I-		NS-FKI------------I--QE-L
	Plasmodium vivax Bra. I	KMZ83675	----I--SLME-VRDV-L--YL----		V-R-RV---V-V--L---I---K--

Figure S55. Alignment of a Conserved Plasmodium protein, showing a 4 amino acid deletion that is exclusive to the “*Vinckeia*” subgenus.

Group	Species	Accession	893		932
"*Vinckei*" (4/4)	*Plasmodium berghei ANKA*	XP_678492	WVIIILIYIIMSQEIWRHE	L	LNPLHKLLLSPSIIRLSSFI
	Plasmodium chabaudi ada.	SCM09373	-I-----------------	F	--------------------
	Plasmodium chabaudi cha.	XP_741775	-I-----------------	F	--------------------
	Plasmodium vinckei pet.	EUD71335	-I-----------------	F	--------------------
	Plasmodium vinckei vin.	XP_008622015	-I-----------------	F	--------------------
	Plasmodium yoelii 17X	ETB56247	-I-----------------	-	--------------------
Other *Plasmodium* (0/16)	*Plasmodium ovale*	SCP05819	-L----V-FMFVKNV--QM		----IR---FT-P-----YV
	Plasmodium falciparum 3D7	XP_001348614	-L--NIV---LV-KM--QM		-----R--I---L----FY-
	Plasmodium falciparum 7G8	EUR62912	-L--NIV---LV-KM--QM		-----R--I---L----FY-
	Plasmodium falciparum Dd2	KOB86298	-L--NIV---LV-KM--QM		-----R--I---L----FY-
	Plasmodium falciparum FCH/4	ETW29102	-L--NIV---LV-KM--QM		-----R--I---L----FY-
	Plasmodium falciparum HB3	KOB62310	-L--NIV---LV-KM--QM		-----R--I---L----FY-
	Plasmodium falciparum IGH-CR14	KNG77339	-L--NIV---LV-KM--QM		-----R--I---L----FY-
	Plasmodium falciparum Mal.	ETW46652	-L--NIV---LV-KM--QM		-----R--I---L----FY-
	Plasmodium falciparum NF135/5.	ETW39804	-L--NIV---LV-KM--QM		-----R--I---L----FY-
	Plasmodium falciparum Pal. Alt.	ETW54117	-L--NIV---LV-KM--QM		-----R--I---L----FY-
	Plasmodium falciparum San. Lu.	EUT78975	-L--NIV---LV-KM--QM		-----R--I---L----FY-
	Plasmodium falciparum Tan.	ETW33809	-L--NIV---LV-KM--QM		-----R--I---L----FY-
	Plasmodium falciparum UGT5.1	EWC73762	-L--NIV---LV-KM--QM		-----R--I---L----FY-
	Plasmodium falciparum Vie.	ETW15983	-L--NIV---LV-KM--QM		-----R--I---L----FY-
	Plasmodium gaboni	XP_018639521	-L--NIV---LV-KM--QM		-----R--I---L----FY-
	Plasmodium gallinaceum	CRG94658	-L--NIV---LV-KM--QM		-----R--I---L----FY-
	Plasmodium reichenowi	SOV83027	-L--NIV---LV-KM--QM		-----R--I---L----FY-
	Plasmodium relictum	CRH01463	-L--NIV---LV-KM--QM		-----R--I---L----FY-
	Plasmodium sp. DRC-Itaito	SOV25183	-L--NIV---LV-KM--QM		-----R--I---L----FY-
	Plasmodium sp. Gor. Cla. G	SOV83324	-L--NIV---LV-KM--QM		-----R--I---L----FY-
	Plasmodium vivax North Kor.	KMZ97769	-LLLNVV--VYV-NV--QM		----YR-M--------V-Y-
	Plasmodium vivax Mau. I	KMZ91745	-LLLNVV--VYV-NV--QM		----YR-M--------V-Y-
	Plasmodium vivax Ind. VII	KMZ78899	-LLLNVV--VYV-NV--QM		----YR-M--------V-Y-
	Plasmodium vivax Bra. I	KMZ87107	-LLLNVV--VYV-NV--QM		----YR-M--------V-Y-
	Plasmodium malariae	SCO94110	-L--NVV-L-LV-N---QI		F----RF-I---L-----Y-
	Plasmodium knowlesi str. H	XP_002260411	-LLLNVV---YV-NV--QM		----YR-M--------A-Y-
	Plasmodium inui San Ant. 1	XP_008814165	-LLLNVV--VYVKNV--QM		----YR-M--------A-Y-
	Plasmodium gonderi	GAW82624	-LL-TIV--LHV-N--GQM		----YR-M--------A-YV
	Plasmodium fragile	XP_012337456	-LLLNVV--VYVHSV--QM		----YR-M--------A-Y-
	Plasmodium cynomolgi str. B	XP_004224070	-LLLNVV--VYV-NV--QM		----YR-M--------A-Y-
	Plasmodium coatneyi	XP_019916810	-LLLNVV---YV-NV--QM		----YR-M--------A-Y-

Figure S56. Alignment of a Conserved *Plasmodium* protein, showing a one amino acid insertion that is exclusive to the *"Vinckeia"* subgenus.

Group	Species	Accession	199		249
"Vinckei" (4/4)	*Plasmodium berghei ANKA*	XP_678034	KSFYMANEKNKYINLSFIRCGTIDE	E	MTEFEIRKIVSSLVQILHDNKSVST
	Plasmodium chabaudi ada.	SCM11927	----IY---------T-----GV--	-	---C-----AP--A-V-----PT-A
	Plasmodium chabaudi cha.	XP_744254	----IS------V--T-----GV--	-	---C-----AP--A-V-----PT-A
	Plasmodium vinckei pet.	EUD71334	----IS---D-----T-----GV--	-	---Y---T------RVV----PA-A
	Plasmodium vinckei vin.	XP_008622014	----IS------V--------GV--	-	---Y----------RVV----PA-A
	Plasmodium yoelii 17X	ETB56248	----IS-D-----------------	-	-S--------------------A-
**Other *Plasmodium*** (0/16)	*Plasmodium malariae*	SBS88844	----IS---K-----AY-G--SAN-		L--L-V-R-A----S------FAKV
	Plasmodium gonderi	GAW82625	----LK------VS-AYLG--LAN-		L--L---R------VM-----YTKV
	Plasmodium ovale wal.	SBT43229	-T--IS--NK---S-AY-G--PVN-		L----V-RM-T-VSTL-----FSKV
	Plasmodium cynomolgi str. B	XP_004224071	----I--D-K--VS-AY-G--PAN-		E--L-----AYA--TM-----YKKV
	Plasmodium vivax	XP_001615930	----I--DQK--VS-AYVG--PAN-		E--L----VAYA--TL---S-HKKV
	Plasmodium knowlesi str. H	XP_002260412	----V----K---S-AYVG--PAN-		E--L-----AYT-ATL--E--NKKV
	Plasmodium fragile	XP_012337457	----I--D-K--VS-AYVG--PAN-		E--L-----TYAIATM-----YKKV
	Plasmodium coatneyi	XP_019916508	----V--D-K---S-AYVG--AAH-		E--L-----ANA-AIM--EH-NKKV
	Plasmodium inui San Ant. 1	XP_008814166	----I--D-K--VS-AYVG--PAK-		E--L-----TYA-ATM-L---YKKV
	Plasmodium relictum	CRH01464	-M-CIL-D-KE---VAC-G--SVND		L--AD--R------LM---YRNSKV
	Plasmodium gallinaceum	CRG94657	-M-CIL-D-RE-V-MA--G--SVN-		LS-AD--R--A---P--QEY-ISKV
	Plasmodium gaboni	SOV19335	-H-F-F-D-KTSVAVGYVG--SVAD		L--ADVKRV-L---SM-----LPKL
	Plasmodium sp. DRC-Itaito	SOV25182	-H-F-F-D-KTSVAVGYVG--SVAD		L--ADVKRV-L---SM-----LPKL
	Plasmodium sp. Gor. Cla. G	SOV83322	-H---F-D-KNAVAVGYVG--SVAD		LN-ADMKRV-L---SM-----LSKL
	Plasmodium reichenowi	XP_012765505	-H---F--NKNSVAVGYVG--SVAD		LN-ADMKRV-L---TM-----LSKL
	Plasmodium falciparum FCH/4	ETW29101	-H---F-DNKNSVAVGYVG--SVAD		LS-ADMKRV-L---TM-----LSKL
	Plasmodium falciparum NF54	EWC85449	-H---F-DNKNSVAVGYVG--SVAD		LS-ADMKRV-L---TM-----LSKL
	Plasmodium falciparum Mal.	ETW46651	-H---F-DNKNSVAVGYVG--SVAD		LS-ADMKRV-L---TM-----LSKL
	Plasmodium falciparum 3D7	XP_001348613	-H---F-DNKNSVAVGYVG--SVAD		LS-ADMKRV-L---TM-----LSKL
	Plasmodium falciparum IGH-CR14	KNG77629	-H---F-DNKNSVAVGYVG--SVAD		LS-ADMKRV-L---TM-----LSKL
	Plasmodium falciparum Vie.	ETW15982	-H---F-DNKNSVAVGYVG--SVAD		LS-ADMKRV-L---TM-----LSKL
	Plasmodium falciparum 7G8	EUR62911	-H---F-DNKNSVAVGYVG--SVAD		LS-ADMKRV-L---TM-----LSKL
	Plasmodium falciparum RAJ116	KNC37661	-H---F-DNKNSVAVGYVG--SVAD		LS-ADMKRV-L---TM-----LSKL

Figure S57. Alignment of the M17 leucyl aminopeptidase protein, showing a one amino acid insertion that is exclusive to the "*Vinckeia*" subgenus.

Group	Organism	Accession	181		232
"*Vinckeia*" (4/4)	*Plasmodium berghei ANKA*	1269284983	KIRNCNADSRKKYVKIIEKVKKNGK		VFIFSDNHISGEQLNSLTGIAAILKFP
	Plasmodium yoelii 17X	564277019	--------I----------------		---------------A--------R--
	Plasmodium chabaudi cha.	70945302	-----S-----------S------		--------T------------------
	Plasmodium vinckei vin.	669201123	-----SV----------S------		--------T------------------
	Plasmodium vinckei pet.	577147901	-----SV----------S------		--------T------------------
Other *Plasmodium* (0/15)	*Plasmodium sp. Gor. Cla. G2*	SOV13170.1	-S-DVKT--E--QVVQH--NT-G	K	-Y------T------------------
	Plasmodium sp. DRC-Itaito	SOV22006.1	-S-DVKT--E--QVVQH--NT-G	K	-Y------T------------------
	Plasmodium coatneyi	1139858901	TF---DVVT--E--AMVSN--NS-G	R	----P---TT-----A-----------
	Plasmodium cynomolgi str. B	457868113	TF---DVVR--E--SMVRD--NS-G	H	----P---TT-----A-----------
	Plasmodium falciparum 3D7	124511978	TF-S-DVKT--E--QVVQY--NT-G	Q	-Y------T------------------
	Plasmodium falciparum FCH/4	574968388	TF-S-DVKT--E--QVVQH--NT-G	Q	-Y------T------------------
	Plasmodium falciparum IGH-CR14	910270685	TF-S-DVKT--E--QVVQH--NT-G	Q	-Y------T------------------
	Plasmodium falciparum NF54	583226870	TF-S-DVKT--E--QVVQY--NT-G	Q	-Y------T------------------
	Plasmodium falciparum UGT5.1	583216512	TF-S-DVKT--E--QVVQH--NT-G	Q	-Y------T------------------
	Plasmodium falciparum Vie.	574751209	TF-S-DVKT--E--QVVQH--NT-G	Q	-Y------T------------------
	Plasmodium fragile	817741125	TF---DVVT--E--AMVRD--GT-G	R	----P---TT-----A-----------
	Plasmodium gaboni	1084821956	TF-S-DVKT--E--QVVQH--NT-G	K	-Y------T------------------
	Plasmodium gonderi	1194445049	TF---DVHT--V-ASM-CQ-RNT-G	T	-YM-----TT-----A-----------
	Plasmodium inui San Ant. 1	672201788	TF---DVVT--A--AMLRD--NS-G	R	----P---TT-----A-----------
	Plasmodium knowlesi str. H	221052834	TF---DVVT--E--AMVRD-RNS-G	R	-Y--P---TT-----A-----------
	Plasmodium malariae	1037141617	TF---DVNR--E---M--N---F-G	S	-Y------T------A-S---------
	Plasmodium ovale curt.	1037175794	IF---DVLR--A--SMV-Q---T-G	K	-Y----Q-T------------------
	Plasmodium ovale wal.	1037163818	IF---DVLR--A--NMV-Q---T-G	K	-Y----Q-T------------------
	Plasmodium reichenowi	1145258209	TF-S-DVKT--E--QVVQH--NT-G	Q	-Y------T------------------
	Plasmodium relictum	1102622191	TL---DLNR--R--QLLQN--NT-G	K	--------T----------V-------
	Plasmodium vivax Bra. I	901874939	TF---DVVT--A--AMVRE--GS-G	R	--V-P---A------A-----------
	Plasmodium vivax Ind. VII	901865805	TF---DVVT--A--AMVRE--GS-G	R	--V-P---A------A-----------
	Plasmodium vivax Mau. I	901883494	TF---DVVT--A--AMVRE--GS-G	R	--V-P---A------A-----------
	Plasmodium vivax North Kor.	901892870	TF---DVVT--A--AMVRE--GS-G	R	--V-P---A------A-----------
Other Apicomplexa	*Toxoplasma gondii ME49*	1084918154	LL-SSDTAE-RRFLRLV-E-ERT-G	E	-LT---Q-T------M-S-V-------
	Neospora caninum Liv.	401398961	LL-SS-TTE-RRF-RLV-E-ERA-G	E	-LT---Q-T------M-S-V-------
	Hammondia hammondi	675126358	LL-SSDTAE-RRFLRLV-E-ERT-G	E	-LT---Q-T------M-S-V-------
Other Eukarya	*Absidia glauca*	1021057638	LF-SADIPT-----ALV-E-RGA-G	K	-YV--SL-V------Q---V----T--
	Acanthaster planci	1229160768	LF-SQDIAQ-QR--ALV-SARE--G	D	-K---SL-V-----DL---V----R--
	Acromyrmex echinatior	332028264	LF-CQDIAQ--E--ELV-N--DS-G	D	-K---SL-V-----DQ------L-R--
	Acyrthosiphon pisum	193664632	LF-SQ-IAE--R---LVDS--ES-G	D	-K---SM-VT----SQI--V----R--
	Aegilops tauschii subsp. taus.	1149727866	LF--TDIA--R---NLV-S---Y-G	T	-H---SM-V--D--AQ--------R--
	Apteryx australis mantelli	926518848	LF-HQDVAT-TR---LVDS-RE-MG	T	-R---SL-V-----GQ---V----R--
	Aquilegia coerulea	1273163331	LF--ADIPT-----DLVSS--DL-G	T	AL---SM-V-----TQ--------R--
	Arabidopsis lyrata subsp. lyr.	1190999052	LF--SDVKT-----NLV-S--DS-G	E	A----AM-V-----AQ------L-R--
	Arabidopsis thaliana	1032291736	LF--SDVKT-----DFV-S--DS-G	E	-----SM-A-----AQH-------R--
	Arabis alpina	674240107	LF--SDVKV-----NLV-S--DF-G	E	-----SR-V--I--AQH-------R--
	Asparagus officinalis	1150747322	LF--ADILT------LV-S--DA-G	T	-HL--SM-V-----AQ--------R--
	Atta colombica	1068402112	LF-CQDIAQ--E--ELV-N--DS-G	D	-K---SL-V-----DQ------L-R--
	Besnoitia besnoiti	1261479163	LL-SSD-AE-RR--RLV-E-ERG-G	E	ALT---Q-T------M-S-V-------
	Blastocystis hominis	855315779	L--GKT--M---IIQLMDA--ES-G	K	S-L--SL-P--Q---N--------R--
	Brassica oleracea var. oler.	922551634	LFK-SDVKE-----DFV-S---L-G	E	A----SM-V-----AMH-----L-R--
	Brassica rapa	685343329	LFK-SDVKE-----DFV-S---L-G	E	A----SM-V-----EMH-----L-R--
	Caenorhabditis brenneri	341889889	LF-SQDINT------LV-S-REQNG	K	-L---SM-V-----DQ---C----RY-
	Genlisea aurea	527204713	LF--ADVAT-Q----LV-S--ACKG	K	-----SM-V-----AQ---V--T-R--
	Glycine max	356570796	LY--EDVET----ASLVKS--EG-G	K	ALVY-SM-V-AP--AQ---V----R--
	Handroanthus impetiginosus	1276263729	LF--SDIAT-----DLVDS--AS-G	S	-H---SM-V-----AQ--------R--
	Heliothis virescens	1247043134	LF-CQDIQQ-RE--A-VDS-RD--G	E	-R---SM-V-----DQ--------R--
	Herrania umbratica	1204914525	LL--KEIAL----MELVKS---A-G	K	A-L--PK-V-----AQ--------R--

Figure S58. A partial sequence alignment of the PelOta protein homologue showing a one amino acid deletion that is specific for members of the *"Vinckeia"* subgenus.

Group	Species	Accession	166		212
***“Vinckeia”* (4/4)**	*Plasmodium berghei ANKA*	XP_679041	VQTGKDIESGTTGIIDIILLGNN	N	KRSNTKMLHEGFISGGLKKIKFQ
	Plasmodium chabaudi ada.	SCM11332	--------A--------------	-	------------T----------
	Plasmodium chabaudi cha.	SCM03023	--------A--------------	-	------------T----------
	Plasmodium vinckei pet.	EUD73669	--------A--------------	-	------------T----------
	Plasmodium vinckei vin.	XP_008624073	--------A--------------	-	------------T-A--------
	Plasmodium yoelii 17X	ETB59440	--------A--------------	-	-----------------------
	Plasmodium yoelii yoe.	EAA20072	--------A--------------	-	-----------------------
**Other *Plasmodium* (0/16)**	*Plasmodium coatneyi*	XP_019917114	------VQA--N-TV--V-----		------V-----T--V-------
	Plasmodium cynomolgi str. B	XP_004224567	-------QA--N-TVE-V----D		------V-Q---K--V-------
	Plasmodium falciparum 3D7	XP_001348240	--------AA---T-E-------		------V-----SA---------
	Plasmodium falciparum 7G8	EUR64855	--------AA---T-E-------		------V-----SA---------
	Plasmodium falciparum FCH/4	ETW29282	--------AA---T-E-------		------V-----SA---------
	Plasmodium falciparum IGH-CR14	KNG76570	--------AA---T-E-------		------V-----SA---------
	Plasmodium fragile	XP_012334852	------VQA--N-TVE-V----D		------V-----T--V-------
	Plasmodium gaboni	XP_018639148	--------AA---T-E-------		------V-----N----------
	Plasmodium gallinaceum	CRG95592	--------A----T-E------E		------I-----N--S-------
	Plasmodium gonderi	GAW83144	--------A----T-E-V-----		------V----------------
	Plasmodium inui San Ant. 1	XP_008817608	------VQA--N-TVE-V----D		------V-----T--V-------
	Plasmodium knowlesi str. H	XP_002260922	-K--NEVQA--S-TV--V-V---		R-----V-----Q--V-------
	Plasmodium malariae	SCP02865	------M-A----T-E-----S-		------V-----NA---------
	Plasmodium ovale curt.	SBS87747	--------A--I-S-E-----E-		------I-----A----------
	Plasmodium ovale wal.	SBT47003	------L-A--I-S-E-----K-		------I-----P----------
	Plasmodium reichenowi	XP_012765134	--------AA---T-E-------		------V-----SA---------
	Plasmodium relictum	CRH04064	------L-A----T-EVV----E		------I-----N--S----R--
	Plasmodium sp. DRC-Itaito	SPJ12467	--------AA---T-E-------		------V-----SA------R--
	Plasmodium sp. Gor. Cla. G2	SOV82376	--------AA---T-E-------		------V-----SA---------
	Plasmodium vivax Bra. I	KMZ83949	------VQA--S-TVE-V----D		------V-----T--V-------
	Plasmodium vivax Ind. VII	KMZ78345	------VQA--S-TVE-V----D		------V-----T--V-------
	Plasmodium vivax North Kor.	KMZ97307	------VQA--S-TVE-V----D		------V-----T--V-------
**Other Apicomplexa**	*Babesia bigemina*	XP_012769523	I---SYKD---S-R-SLM-I-PL		GK----S-GTN-YA-SYLTFVLK
	Babesia bovis	XP_001612018	I---SHKG---S-KVSVM---PL		GK----I-GTN-Y--SYLTFVVE
	Babesia divergens	ADB80151	I---SHKD---S-TVSLM---TL		G-----VMGAN-YT-SYLTFVVK
	Babesia microti str. RI	XP_021337760	LK--SQ-GA--E-NVR-K-I-E-		GQTGSIY-TNHLKR-SVNIYHIK
	Babesia ovata	GBE58642	I---SYKD---S-R-SLM-I-SL		GK----V-GTN-YT-SYLTFVLK
	Babesia sp. Xinjiang	ORM39994	I---SHKD---S-KVSLM---PL		GK----V-GTN-YT-SYLTFVIE
	Theileria annulata	XP_953429	----GQKDA-SN-T-SLT---SS		G-T-E-T-SNS-YPASYQTFQVK
	Theileria equi	XP_004831308	I---IQKDA--N-T-GLT-I-S-		GKT-E-K-SNS-D--SYLTFQVK
	Theileria orientalis str. Shi.	XP_009692052	----GQKDA-SS-T-AMT---TS		G-T-Q-T-SNA-Y-ASYLTFQLK
	Theileria parva str. Mug.	XP_764433	----GQKDA-SN-T-SLA---SS		GKT-E-T-SNA-YPASYQTFQVK
	Besnoitia besnoiti	PFH37729	L---N-EHA--S-G-ELT-I-SV		GQ-AP-L-IQDVRP-AERRVR-R
	Eimeria necatrix	XP_013440506	----N-EHA--V-P-ELTMS-SY		RQASP-L-LL-VKP-AEERVA-R
	Hammondia hammondi	XP_008887346	M-A-N-EHA--S-G-ELT-I-SE		RQ-SP-L-VQDVRP-AERR-R-R
	Neospora caninum Liv.	CEL66641	L---N-EHA--S-G-ELT-I-SE		RQ-AP-L-VQDVRP-AERRVR-R
	Toxoplasma gondii ME49	XP_018637162	M---N-EHA--S-G-ELT-I-SE		RQ-SP-L-VQDVRP-AERR-R-R
	Cryptosporidium andersoni	OII76058	-M---SG-A--K-GVE-S-I-ES		AH-DKVL-KQ--Y-STVRR-I-R
	Cryptosporidium hominis TU502	XP_667778	IM---SG-A--K-GVE-SIS-EA		GQ-DKIL-KQ--Y-ST-RRML-R
	Cryptosporidium meleagridis	POM85607	IM---SG-A--K-GVE-SIS-DA		GQ-DKIL-KQ--Y-ST-RRML-R
	Cryptosporidium muris RN66	XP_002140817	-M---SG-A--K-GVE-S-V-ES		AH-DKIL-KQ--Y-STVRR-I-R
	Cryptosporidium parvum Iowa II	XP_626313	IM---SG-A--K-GVE-SIS-EA		GQ-DKIL-KQ--Y-ST-RRML-R
	Cryptosporidium ubiquitum	OII72463	IM---SG-A--R-GVE-SIS-DA		GQ-DKIL-KQ--Y-ST-RRML-R

Figure S59. Alignment of the LCCL domain-containing protein, showing a one amino acid insertion that is exclusive to the *“Vinckeia”* subgenus.

Group	Species	Accession	442	Insertion	491
“Plasmodium” (6/7)	*Plasmodium coatneyi*	XP_019913407	TDVAARGINITSVQNVINYSLPFSPKLFIHRVGRACRDDAM	PS	GYAVSLV
	Plasmodium fragile	XP_012333808	-------------------N---------------------	--	-------
	Plasmodium gonderi	GAW79636	-------------------------------I-------NT	-H	-F-I---
	Plasmodium inui San Ant. 1	XP_008816501	-------------------N---------------------	S-	-------
	Plasmodium knowlesi str. H	XP_002258052	---	--	-------
	Plasmodium vivax Bra. I	KMZ88036	---	-A	-------
	Plasmodium vivax Ind. VII	KMZ82012	---	-A	-------
	Plasmodium vivax Mau. I	KMZ94414	---	-A	-------
	Plasmodium vivax North Kor.	KNA01258	---	-A	-------
	Plasmodium vivax Sal-1	XP_001614894	---	-A	-------
“Vinckeia” (0/4)	*Plasmodium berghei ANKA*	CDS45652	--L-S---H-P--------N------I-L--I-----TECQ	-	--GI-I-
	Plasmodium chabaudi ada.	SCN60705	--L-S--------------N------I-L--I-----MEGQ	-	--GI-I-
	Plasmodium chabaudi cha.	CAH75349	--L-S--------------N------I-L--I-----MEGQ	-	--GI-I-
	Plasmodium vinckei pet.	EUD70693	--L-S--------------N------I-L--I-----TEGQ	-	--GI-I-
	Plasmodium vinckei vin.	XP_008626234	--L-S--------------N------I-L--I-----TEGQ	-	--GI-I-
	Plasmodium yoelii 17X	ETB58610	--L-S--------------N------I-L--I-----V-NQ	-	--G--I-
	Plasmodium yoelii yoe. 17XNL	XP_723756	--L-S--------------N------I-L--I-----V-NQ	-	--G--I-
Other *Plasmodium* (0/9)	*Plasmodium falciparum 3D7*	XP_001349256	----S--------------N-----------I-----T-IS		--GI--L
	Plasmodium falciparum 7G8	EUR46871	----S--------------N-----------I-----T-IS		--GI--L
	Plasmodium falciparum CAMP.	ETW62076	----S--------------N-----------I-----T-IS		--GI--L
	Plasmodium falciparum Dd2	KOB84732	----S--------------N-----------I-----T-IS		--GI--L
	Plasmodium falciparum FCH/4	ETW30994	----S--------------N-----------I-----T-IS		--GI--L
	Plasmodium falciparum HB3	KOB60990	----S--------------N-----------I-----T-IS		--GI--L
	Plasmodium falciparum IGH-CR14	KNG76216	----S--------------N-----------I-----TNIS		--GI--L
	Plasmodium falciparum Mal.	ETW49694	----S--------------N-----------I-----T-IS		--GI--L
	Plasmodium falciparum NF135/5.	ETW43421	----S--------------N-----------I-----T-IS		--GI--L
	Plasmodium falciparum NF54	EWC88823	----S--------------N-----------I-----T-IS		--GI--L
	Plasmodium falciparum Pal. Alt.	ETW52389	----S--------------N-----------I-----T-IS		--GI--L
	Plasmodium falciparum RAJ116	KNC35823	----S--------------N-----------I-----T-IS		--GI--L
	Plasmodium falciparum San. Lu.	EUT87343	----S--------------N-----------I-----T-IS		--GI--L
	Plasmodium falciparum Tan.	ETW36986	----S--------------N-----------I-----T-IS		--GI--L
	Plasmodium falciparum UGT5.1	EWC76992	----S--------------N-----------I-----T-IS		--GI--L
	Plasmodium falciparum Vie.	ETW18826	----S--------------N-----------I-----T-IS		--GI--L
	Plasmodium sp. Gor. Cla. G2	SOV13816.1	----S--------------N------I----I-----T-IN		--GI--L
	Plasmodium sp. DRC-Itaito	SOV22350.1	----S--------------N-----------I-----T-IN		--GI--L
	Plasmodium gaboni	XP_018642358	----S--------------N-----------I-----T-IN		--GI--L
	Plasmodium gallinaceum	CRG93495	--L-S--------------N----S------I-----NNMK		---I-I-
	Plasmodium malariae	SBS82697	--L-S------T---I---N--Y--------------NNLT		--GI---
	Plasmodium ovale curt.	SBS80989	--L-S----------I---N------V----I-----GTQS		--GI-II
	Plasmodium ovale wal.	SBT32573	----S--------------N------V----I-----GTQN		--GI-II
	Plasmodium reichenowi	CDO64037	----S--------------N-----------I-----T-IN		--GI--L
	Plasmodium relictum	CRH03734	--L-S-----A--K-----N----S------I-----NNFK		---I-I-
Other Eukarya	*Meyerozyma guilliermondii AT.*	A5DLR3	--------D-PVLA--V--T--G-S-I-------TA-AGNK		-W-Y-I-
	Kluyveromyces marxianus	BAP73007	-------VD-PLLA--------G-S-I-V-----TA-AGNR		-W-F-I-
	Penicillium roqueforti FM.	CDM31467	--------D-PVLA-----DF-SQ--I-------TA-AGQK		-WSY---
	Geotrichum candidum	CDO55597	--------D-PVLA--------S---V-------TA-AGRR		-W-YTI-
	Kluyveromyces dobzhanskii C.	CDO96468	-------VD-PLLA--------G-S-I-------TA-AGNR		-W-F-I-
	Histoplasma capsulatum G1.	EEH06888	--------D-PILS-----DF-SQ--I-V-----TA-AGKT		-WSY--I
	Macrophomina phaseolina MS6	EKG19568	--------D-PILA----FDF-AQ--I-------TA-AGKK		-W-Y---
	Metarhizium anisopliae BRIP.	KJK80380	--------D-PVLA-----DF-SQ--I-V-----TA-AGQR		-WSY---
	Thielaviopsis punctulata	KKA30234	--------D-PVLK-----DF-PQ--V-V-----TA-AGQT		-W-Y---
	Ceratocystis platani	KKF97604	--------D-PVLK-----DF-PQ--V-V-----TA-AGQT		-WSY---
	Trichoderma harzianum	KKO99071	--------D-PVLA-----DF-PQ--V-------TA-AGQR		-W-Y---
	Emmonsia crescens UAMH 30.	KKZ66844	--------D-PILS-----DF-SQ--I-V-----TA-AGKT		-WSY--I
	Penicillium nordicum	KOS39283	--------D-PVLA-----DF-SQ--I-------TA-AGQK		-WSY---
	Penicillium freii	KUM59734	--------D-PVLA-----DF-SQ--I-------TA-AGQK		-WSY---

Figure S60. A partial sequence alignment of a DEAD-box family helicase 4 protein showing a two amino acid insertion that is specific for members of the *“Plasmodium”* subgenus (sans *P. ovale* and *P. malariae*) and a one amino acid insertion in the same position that is specific for members of the *“Vinckeia”* subgenus. CSI region is not conserved in *P. cynomolgi*.

Group	Species	Accession	346		401
**"*Plasmodium*" (7/7)**	Plasmodium vivax	SCO68989	ELKMGAYLSVGKGSMYPNKFIHLTYKG	AQTGA	SQNEKKKIALIGKGITFDSGGYNL
	Plasmodium knowlesi str. H	XP_002260412	D--------------------------	-KK-D	KG-T--------------------
	Plasmodium inui San Ant. 1	XP_008814166	-----SF--------------------	--K-D	N--KR-----V-------------
	Plasmodium gonderi	GAW82625	-----------------S---------	-KN-D	NN-I------V-------------
	Plasmodium fragile	XP_012337457	D--------------------------	--K-D	NK----------------------
	Plasmodium cynomolgi str. B	XP_004224071	D--------------------------	--K-D	N--KR-------------------
	Plasmodium coatneyi	XP_019916508	---------------------------	-HK-D	K--KV-------------------
**Other *Plasmodium* (0/13)**	Plasmodium berghei ANKA	XP_678034	-----------------R--------		KGDI------V-------------
	Plasmodium chabaudi ada.	SCM11913	-----------------HR--------		KGEI------V-------A-----
	Plasmodium chabaudi cha.	SCM07105	-----------------HR--------		KGDI------V-------A-----
	Plasmodium falciparum 3D7	XP_001348613	--------------------------S		KGDV------V-------------
	Plasmodium falciparum 7G8	EUR62911	--------------------------S		KGDV------V-------------
	Plasmodium falciparum FCH/4	ETW29101	--------------------------S		KGDV------V-------------
	Plasmodium falciparum IGH-CR14	KNG77629	--------------------------S		KGDV------V-------------
	Plasmodium falciparum Mal.	ETW46651	--------------------------S		KGDV------V-------------
	Plasmodium falciparum NF54	EWC85449	--------------------------S		KGDV------V-------------
	Plasmodium falciparum RAJ116	KNC37661	--------------------------S		KGDV------V-------------
	Plasmodium falciparum Vie.	ETW15982	--------------------------S		KGDV------V-------------
	Plasmodium gaboni	SOV19335	--------------------------S		KGDV---V--V-------------
	Plasmodium gallinaceum	CRG94657	------------------R--------		NGEI---V--V------------I
	Plasmodium malariae	SBS88844	--------------L------------		KGDI------V-------------
	Plasmodium ovale wallikeri	SBT43229	------------------H--------		KGDI------V-------------
	Plasmodium reichenowi	XP_012765505	--------------------------S		KGDV------V-------------
	Plasmodium relictum	CRH01464	------F----------HR--------		KGEI------V-------------
	Plasmodium sp. DRC-Itaito	SPJ12831	--------------------------S		KGDV------V-------------
	Plasmodium sp. Gor. Cla. G	SOV83322	D-------------------------S		KGDV------V-------------
	Plasmodium vinckei pet.	EUD71337	-----------------PR--------		KGEI------V-------A-----
	Plasmodium vinckei vin.	XP_008622014	-----S-----------HR--------		KGDI------V-------------
	Plasmodium yoelii 17X	ETB56248	------------------R--------		KGDI------V-------------
**Other Eukarya**	Ectocarpus siliculosus	CBJ32669	-G----MG-AQ-AVF-P--------P		-GTP---V------L--------I
	Guillardia theta CCMP.	XP_005835498	-----A-SR--AQ-P----V---P		KGEV-----IV---LC--------
	Brassica rapa	XP_009117239	-----S--A-AAA-AN-PF----V-RP	S	-GDV-T-L--V---L--------I
	Raphanus sativus	XP_018464138	-----S--A-AAA-AN-PF----V-RP	P	GGDV-T-L--V---L--------I
	Nicotiana tabacum	AGW47884	-----S--A--EA-AN-PH----C--P	T	GGEI---L--V---L--------I
	Nicotiana sylvestris	XP_009795110	-----S--A--EA-AN-PH----C--P	T	GGEI---L--V---L--------I
	Brassica oleracea var. ole.	XP_013605509	-----S--A-AAA-AN-PF----V-RP	P	-GDV-T-L--V---L--------I
	Capsella rubella	XP_023640636	-----S--A-AAA-AN-PH----I--P	-	-GSV-T-L--V---L--------I
	Nicotiana tomentosiformis	XP_009614630	-----S--A--EA-AN-PH----C--P	T	GGEI---L--V---L--------I
	Brassica napus	CDY34790	-----S--A-AAA-AN-PF----V-RP	P	-GDV-T-L--V---L--------I
	Ananas comosus	OAY69341	-----S--A-AAA-SN-PH----R-TP	P	NGDV-R-L-IV---L--------I
	Trema orientalis	PON99391	-----S--G-AAA-AN-PH-------P	P	-GPV-T-L--V---L--------I
	Nicotiana attenuata	XP_019246753	-----S--A--EA-AN-PH----C--P	T	GGEI---L--V---L--------I
	Morus notabilis	XP_010103674	-----S--G-AAA-AN-PR-------P	P	-GDV-T-L--V---L--------I
	Erythranthe guttata	XP_012839866	-----S--G-AAA-AN-PY----C--P	L	-GEV-T-L--V---L--------I
	Eutrema salsugineum	XP_006412653	-----S--A-AAA-AN-PH----V--P	S	-GPV-T-L--V---L--------I
	Oryza brachyantha	XP_006648044	-----S--A-AAA-AN-PH----C--P	P	GG-A-R-L-IV---L--------I
	Medicago truncatula	AFK42034	-----S--G-AAA-AN-PR-------P	P	-GSV-V-L--V---L--------I
	Setaria italica	XP_022684649	-----S--A-AAA-AN-PH----C--P	T	DG-V-R-L-IV---L--------I
	Arabidopsis thaliana	NP_194821	-----S--A-AAA-AN-PH----I--P	S	-GPV-T-L--V---L--------I
	Helianthus annuus	XP_021981455	-----S--G-AAA-AN-P-----C--P	T	-GTI---L--V---L--------I
	Oryza sativa Japonica Group	XP_015624560	-----S--G-AAA-AN-PH----C--P	P	GG-A-R-L-IV---L--------I
	Camelina sativa	XP_010447643	-----S--A-AAA-AN-PH----V--P	S	-GPV-T-L-IV---L--------I
	Beta vulgaris subsp. vul.	KMT03558	-----S--A-AAA-AN-PH----C--P	L	-GPVTA-L--V---L--------I
	Sorghum bicolor	XP_002454700	-----S--G-AAA-AN-PH----C--P	T	DG-V-R-L-IV---L--------I
	Galdieria sulphuraria	XP_005704316	KMN--C--G-AQ--SE-P-----K-SP	V	QGPV---------A----------

Figure S61. Alignment of the Leucine aminopeptidase protein, showing a five/four amino acid insertion that is exclusive to the *"Plasmodium"* subgenus (sans *P. ovale* and *P. malariae*). Expanded alignment of Figure 5 (D).

Group	Species	Accession	384		433
"*Plasmodium*" (7/7)	*Plasmodium vivax*	XP_001616802	ESIEVYLASSDIYNIFFSIMEPRI	K	KLLGLFIFYPAHIFIMRKGLRFAIT
	Plasmodium knowlesi str. H	SBO22512	--------P---------T-Q--V	-	-------------------------
	Plasmodium inui San Ant. 1	XP_008817514	--------P---------------	-	-------------------------
	Plasmodium gonderi	GAW83065	-------S---------FLVKS--	N	-I------------V----------
	Plasmodium fragile	XP_012333630	--------P---------V--S--	-	-------------------------
	Plasmodium cynomolgi str. B	XP_004224489	--------P---------------	-	-------------------------
	Plasmodium coatneyi	XP_019917145	--V-----P---------------	-	------------L------------
Other *Plasmodium* (0/13)	*Plasmodium berghei ANKA*	XP_022714352	-F-QI--S----------YID-KT		HI---------YL----T-------
	Plasmodium chabaudi ada.	SCM07970	-F-QI--S----------YID-KT		HI---------YL----T-------
	Plasmodium chabaudi cha.	XP_743998	-F-QI--S----------YID-KT		HI---------YL----T-------
	Plasmodium falciparum 3D7	XP_001348322	H--QIF-S-D--------SID-K-		NV--------CTL-L--S-I-----
	Plasmodium falciparum 7G8	EUR64185	H--QIF-S-D--------SID-K-		NV--------CTL-L--S-I-----
	Plasmodium falciparum CAMP.	ETW58934	H--QIF-S-D--------SID-K-		NV--------CTL-L--S-I-----
	Plasmodium falciparum FCH/4	ETW29215	H--QIF-S-D--------SID-K-		NV--------CTL-L--S-I-----
	Plasmodium falciparum NF135/5.	ETW40308	H--QIF-S-D--------SID-K-		NV--------CTL-L--S-I-----
	Plasmodium falciparum NF54	EWC86032	H--QIF-S-D--------SID-K-		NV--------CTL-L--S-I-----
	Plasmodium falciparum Pal. Alt.	ETW53787	H--QIF-S-D--------SID-K-		NV--------CTL-L--S-I-----
	Plasmodium falciparum San. Lu.	EUT78646	H--QIF-S-D--------SID-K-		NV--------CTL-L--S-I-----
	Plasmodium falciparum Tan.	ETW34178	H--QIF-S-D--------SID-K-		NV--------CTL-L--S-I-----
	Plasmodium falciparum Vie.	ETW16236	H--QIF-S-D--------SID-K-		NV--------CTL-L--S-I-----
	Plasmodium gaboni	SOV18774	H--QIF-S-D--------SID-K-		NV--------CNL-L--S-I-----
	Plasmodium gallinaceum	CRG95670	---QI--S----------SS--DK		NII--------Y-------------
	Plasmodium malariae	SBT72395	-C-QI--S----------YTK-K-		NII--------N-------------
	Plasmodium ovale curt.	SBS87574	-A-QI--S----------YTQ-K-		NI---------N-------------
	Plasmodium ovale wal.	SBT45808	-A-QI--S----------YTQ-K-		NI---------N-------------
	Plasmodium reichenowi	CDO66635	H--QIF-S-D--------SID-K-		NV--------CTL-L--S-I-----
	Plasmodium relictum	CRH03986	---QI--S----------SS--DK		YVI--------Y-------------
	Plasmodium sp. DRC-Itaito	SOV24892	H--QIF-S-D--------SID-K-		NV--------CNL-L--S-I-----
	Plasmodium sp. Gor. Cla. G	SOV18824	H--QIF-S-D--------SID-K-		NI--------CNL-L--S-I-----
	Plasmodium vinckei pet.	EUD73747	-F-QI--S----------YID-KT		HI---------YL----T-------
	Plasmodium vinckei vin.	XP_008623996	-F-QI--S----------YID-K-		HT---------YL----S-------
	Plasmodium yoelii 17X	ETB59547	-F-QI--S----------YID-KT		HI---------YL----T-------
	Plasmodium yoelii yoe.	EAA16321	-F-QI--S----------YID-KT		HI---------YL----T-------

Figure S62. Alignment of a Conserved hypothetical protein, showing a one amino acid insertion that is exclusive to the "*Plasmodium*" subgenus (sans *P. ovale* and *P. malariae*).

			879		922
**"*Plasmodium*" (7/7)**	*Plasmodium vivax North Kor.*	KMZ97769	SIQNFLKVKLSFVRSVRMMN	S	WEESLKIKLGMYKLYLLIIVSFF
	Plasmodium vivax Mau. I	KMZ91745	--------------------	-	-----------------------
	Plasmodium vivax Ind. VII	KMZ78899	--------------------	-	-----------------------
	Plasmodium vivax Bra. I	KMZ87107	--------------------	-	-----------------------
	Plasmodium knowlesi str. H	XP_002260411	-------LR----------D	-	---------S---M---------
	Plasmodium inui San Ant. 1	XP_008814165	-------L-----------D	-	-----R---S--RM---------
	Plasmodium gonderi	GAW82624	--L----L-------IGI-D	T	----I----N---S-----IA--
	Plasmodium fragile	XP_012337456	-------W-----------D	-	---------R---V----V----
	Plasmodium cynomolgi str. B	XP_004224070	-------L-----------D	-	-----T---N-------------
	Plasmodium coatneyi	XP_019916810	-------LR----------D	-	---------S---M---------
**Other *Plasmodium* (0/13)**	*Plasmodium berghei ANKA*	XP_678492	--I---NE--AY--NIG-Y-		--R-IY--IN---H--F-VII--
	Plasmodium chabaudi ada.	SCM09373	--I---NK--AY--DIG-Y-		--Q-ICT-IN---H--C-VIM--
	Plasmodium chabaudi cha.	XP_741775	--I---NK--AY--DIG-Y-		--Q-ICT-IN---H--C-VIM--
	Plasmodium falciparum 3D7	XP_001348614	--I----L------D-GIDA		----IR---N---V--SV-II--
	Plasmodium falciparum 7G8	EUR62912	--I----L------D-GIDA		----IR---N---V--SV-II--
	Plasmodium falciparum Dd2	KOB86298	--I----L------D-GIDA		----IR---N---V--SV-II--
	Plasmodium falciparum IGH-CR14	KNG77339	--I----L------D-GIDA		----IR---N---V--SV-II--
	Plasmodium falciparum Mal.	ETW46652	--I----L------D-GIDA		----IR---N---V--SV-II--
	Plasmodium falciparum NF135/5.	ETW39804	--I----L------D-GIDA		----IR---N---V--SV-II--
	Plasmodium falciparum Pal. Alt.	ETW54117	--I----L------D-GIDA		----IR---N---V--SV-II--
	Plasmodium falciparum San. Lu.	EUT78975	--I----L------D-GIDA		----IR---N---V--SV-II--
	Plasmodium falciparum Tan.	ETW33809	--I----L------D-GIDA		----IR---N---V--SV-II--
	Plasmodium falciparum UGT5.1	EWC73762	--I----L------D-GIDA		----IR---N---V--SV-II--
	Plasmodium falciparum Vie.	ETW15983	--I----L------D-GIDA		----IR---N---V--SV-II--
	Plasmodium gaboni	XP_018639521	--I----L------D-GIDA		----IC---N---V--TV-II--
	Plasmodium gallinaceum	CRG94658	--IK---L------DA-IHA		----INV--N---A--TVV-I--
	Plasmodium malariae	SCO94110	--IK---L------NFGLRE		--Q-IH---S---V--CL-IL--
	Plasmodium ovale	SCP05819	C-IK------AL--NFG-YT		----ILL--S--RY--SL-IV--
	Plasmodium reichenowi	CDO66927	--I----L------D-GIDA		----IR---N---V--SV-II--
	Plasmodium relictum	CRH01463	--IK---L------DA-IHA		----IS---N---A--TV--I--
	Plasmodium sp. DRC-Itaito	SPJ12832	--I----L------D-GIDA		----IR---N---V--TV-II--
	Plasmodium sp. Gor. Cla. G	SOS81287	--I----L------D-GIDA		----IR---N---V--SV-II--
	Plasmodium vinckei pet.	EUD71335	--ID--NK--AY--DIG-Y-		--Q-IYT-IN---H--C-V-T--
	Plasmodium vinckei vin.	XP_008622015	--ID--SK--AY--DIG-Y-		--Q-IYT-IN---H--C-V-T--
	Plasmodium yoelii 17X	ETB56247	--I---NE--AY--DIG-Y-		--Q--YT-IN---H--Y-VII--
**Other Apicomplexa**	*Besnoitia besnoiti*	PFH32585	RNMRH-QLR-AY--L-QVQD		---A--V--R-FRSLYRLSAV--
	Cystoisospora suis	PHJ21345	RNIRH-QLR-AY-QM--VHD		--AA-NV--W-LRSLYRVATA--
	Hammondia hammondi	XP_008885121	RNIRH-ELR-AY--L-QVQD		---A-NV--E-FQSLYRFAAA--
	Neospora caninum Liv.	XP_003880484	RNVRH-ELR-AY--L-QVQD		---A-NV--K-FRSLYRFSTA--
	Toxoplasma gondii ME49	XP_018638105	RNIRH-ELR-AY--L-QVQD		---A-NV--E-FQSLYRFAAA--

Figure S63. Alignment of the Hypothetical protein PVBG_03892, showing a one amino acid insertion that is exclusive to the *"Plasmodium*" subgenus (sans *P. ovale* and *P. malariae*).

Group	Species	Accession	140		179
***"Plasmodium"* (7/7)**	*Plasmodium vivax North Kor.*	KMZ97919	FSVINIFVPNCNNNVKKKKL	I	LKLLQKKVKNILALNLLTL
	Plasmodium vivax Mau. I	KMZ91708	--------------------	-	-------------------
	Plasmodium knowlesi str. H	XP_002260374	-------------------K	-	-------------------
	Plasmodium inui San Ant. 1	XP_008814130	-------------------I	-	-N-----------------
	Plasmodium gonderi	GAW82588	--A-IMY-------T-----	-	----K--------------
	Plasmodium fragile	XP_012336497	-------------------A	-	-T--E-----V--------
	Plasmodium cynomolgi str. B	XP_004224034	-------------------A	-	-------------------
	Plasmodium coatneyi	XP_019916901	-------------D---R-A	-	--S----------------
**Other *Plasmodium* (0/12)**	*Plasmodium malariae*	SBS88766	-----LY--T--T-L-R-SI		----K-H-ND-------I-
	Plasmodium relictum	CRH01427	--I---Y-----T-L-R-II		----K-Q-ND----K----
	Plasmodium ovale wal.	SBT43056	T-I--MY-----SKT-R-II		F---KRQ-ND----K----
	Plasmodium ovale curt.	SBS86673	T-I--MY-----SKT-R-II		F-H-KRQ-ND----K----
	Plasmodium falciparum HB3	KOB61622	-YT-S-Y--S--S-I-R-VI		I-V-R-HLN-V---K----
	Plasmodium falciparum NF135/5.	ETW39764	-YT-S-Y--S--S-I-R-VI		I-V-R-HLN-V---K----
	Plasmodium falciparum 3D7	XP_001348649	-YT-S-Y--S--S-I-R-VI		I-V-R-HLN-V---K----
	Plasmodium falciparum Mal.	ETW46422	-YT-S-Y--S--S-I-R-VI		I-V-R-HLN-V---K----
	Plasmodium sp. Gor. Cla. G	SOS81323	-YT-S-Y--S--S-I-R-VI		I-V-R-HLN-V---K----
	Plasmodium reichenowi	CDO66963	-YT-S-Y--S--S-I-R-VI		I-V-R-HLN-V---K----
	Plasmodium gallinaceum	CRG94694	--T---Y-------F---II		----K-Q-ND----K----
	Plasmodium berghei ANKA	XP_022713584	---V-NY-----S-L-R-V-		----K-Y-NE----K----
	Plasmodium yoelii	XP_728181	L--V-NY-----S-I-R-V-		----K-Y--E----K----
	Plasmodium gaboni	SOV19410	-YT-S-Y--S--TSI-R-VI		-R--K-QLN-V--------
	Plasmodium chabaudi ada.	SCM09561	-----NY-----T-F---VI		----K-Y-SD--S-K----
	Plasmodium chabaudi cha.	XP_016654491	-----NY-----T-F---VI		----K-Y-SD--S-K----
	Plasmodium vinckei pet.	EUD71371	-----NY-----S-L---VV		----K-Y-SD--S-K----
	Plasmodium vinckei vin.	XP_008622049	-----NY-----T-L---VV		----K-Y-SG--S-K----

Figure S64. Alignment of the Hypothetical protein PVMG_00581, showing a one amino acid insertion that is exclusive to the *"Plasmodium*" subgenus (sans *P. ovale* and *P. malariae*). CSI region is not conserved in *Plasmodium sp. DRC-Itaito*.

“*Plasmodium*” (7/7)

```
                                                        278                                              323
Plasmodium vivax North Kor.       KMZ97919         LDYLIYAKKLSYVDFSSD H QYDETSLDGFYCVPTARFESEVNMTQY
Plasmodium vivax Mau. I           KMZ91708         ------------------ - ---------------------------
Plasmodium knowlesi str. H        XP_002260374     ------S-----I----- - ----S-------I-----------K--
Plasmodium inui San Ant. 1        XP_008814130     ------------I----- Y ------------I--P-----------
Plasmodium gonderi                GAW82588         ------S-----I---NN - ----N-M-----I------K-I-IRH-
Plasmodium fragile                XP_012336497     ------------I---NN - ----S-------I---------H-K--
Plasmodium cynomolgi str. B       XP_004224034     ------------I----- - ------------I-----------K--
Plasmodium coatneyi               XP_019916901     --F---------I---T- - ----S-------I------N-I--K--
```

Other *Plasmodium* (0/13)

```
Plasmodium sp. DRC-Itaito         SOV25219.1       I-LI--------IE-NNE   ----N------II--L--QY-I--KA-
Plasmodium relictum               CRH01427         ---I--V--------YN-   ----N-------T--I---Y-I--KN-
Plasmodium gallinaceum            CRG94694         ---I--------I--YN-   ----N-------T--I-Y-Y-I--KN-
Plasmodium malariae               SBS88766         ---I--------I---N-   ----H------IT--I---Y-I--EY-
Plasmodium berghei ANKA           XP_022713584     I--I--S-----I--IN-   ----Y-------I--L---Y---TKH-
Plasmodium ovale wal.             SBT43056          --IV-S---A-I---N-   ----N-------I--V--DY-M-TKE-
Plasmodium ovale curt.            SBS86673          --IV-S---A-I--IN-   ----N-------I--V---C-M-TKE-
Plasmodium sp. Gor. cla. G        SOV83396         --FI--------IE-NNE   ----N------II--L--QY-I--KA-
Plasmodium vinckei pet.           EUD71371         I--I--S-----I--TN-   -H-NK----L-----L---Y---NVN-
Plasmodium reichenowi             CDO66963         --LI--------IE--NE   ----N-I----II--L--QY-I--KA-
Plasmodium falciparum NF135/5.    ETW39764         --LI--------IE--NE   ----N-I----II--L--QY-I--KA-
Plasmodium falciparum Mal.        ETW46422         --LI--------IE--NE   ----N-I----II--L--QY-I--KA-
Plasmodium falciparum 3D7         XP_001348649     --LI--------IE--NE   ----N-I----II--L--QY-I--KA-
Plasmodium vinckei vin.           XP_008622049     I--I--S-----I--II-   ---NK----L-----L---Y---NVH-
Plasmodium gaboni                 SOV19410         I-LI--------IE-NNE   ----N------II--L--QY-I--KA-
Plasmodium chabaudi ada.          SCM09561         V--I--S-----I--II-   -H-NK----Y-----L---Y---NVH-
Plasmodium chabaudi cha.          XP_016654491     V--I--S-----I--II-   HH-IK----L-----L---Y---NVH-
Plasmodium yoelii                 XP_728181              S-----I--AN-   ----Y-------I--L---Y---TKY-
```

Figure S65. Alignment of the Hypothetical protein PVNG_02680, showing a one amino acid insertion that is exclusive to the *‘‘Plasmodium*” subgenus (sans *P. ovale* and *P. malariae*).

Group	Organism	Accession	546		598
**"*Plasmodium*" (7/7)**	*Plasmodium vivax*	SCO74371	EAYEDRKYIYMILEYVSNGDLLAYVC	K	KKRRINEDTARRIFYQLISAVDYLHK
	Plasmodium vivax Ind. VII	KMZ78818	--------------------------	-	--------------------------
	Plasmodium vivax Sal-1	XP_001615847	--------------------------	-	--------------------------
	Plasmodium gonderi	GAW82544	-V--N--N--------------T-IY	-	N-K------T----------------
	Plasmodium inui San Ant. 1	XP_008814080	-----G-N--------P--------Y	-	R-------------------------
	Plasmodium knowlesi str. H	XP_002260332	-----G-N-------IP-----S---	-	--------------------------
	Plasmodium fragile	XP_012336456	-----G-N--------P-----E---	-	--------------------------
	Plasmodium coatneyi	XP_019916229	-----G-N--------P-----T---	-	--K------------------N----
	Plasmodium cynomolgi str. B	XP_004223991	-----G-N--------P-----T---	-	--------------------------
**Other *Plasmodium* (3/13)**	*Plasmodium berghei ANKA*	CDS50246	-VH-NKNN-------LG-----T-I-		-NSN---NI-K--L------IE----
	Plasmodium chabaudi ada.	SCM09778	-VH-NKNN-------LG-------I-		-NNN---NI-K--L------IE----
	Plasmodium chabaudi cha.	SCM04321	-VH-NKNN-------LG-------I-		-NNN---NI-K--L------IE----
	Plasmodium falciparum	CAA80490	DV-QNKNN--------E--N--T-IY		NNYN---NN----L----N-IE---E
	Plasmodium falciparum 3D7	XP_001348690	DV-QNKNN--------E--N--T-IY		NNYN---NN----L----N-IE---E
	Plasmodium falciparum FCH/4	ETW28146	DV-QNKNN--------E--N--T-IY		NNYN---NN----L----N-IE---E
	Plasmodium falciparum HB3	KOB58913	DV-QNKNN--------E--N--T-IY		NNYN---NN----L----N-IE---E
	Plasmodium gallinaceum	CRG94738	-VH-NI-NL------LP-----T-IY		-NNY---NK----L-------E----
	Plasmodium malariae	SBS99683	-VH-NKNNL------LPK----T-IY		E-KK----S--T--------I-----
	Plasmodium ovale curt.	SBS86604	-VH-SKNNL------LP-----T--Y		-NEN---NS----L------I-----
	Plasmodium ovale wal.	SBT42784	-VH-SKNNL------LP-----T--Y		-NEN---NS----L------I-----
	Plasmodium reichenowi	CDO67007	DV-QNKNN--------E--N--T-IY		NNYN---NN----L----N-IE----
	Plasmodium relictum	CRH01383	-VH-N--NL------LP-----T-IY		-NNY---NK----L----N-IE----
	Plasmodium vinckei pet.	EUD71414	-VH-NKNN-------LG-----T-I-		-NSN---SI-K--L------IE----
	Plasmodium vinckei vin.	XP_008622092	-VH-NKNN-------LG-----T-I-		-NSN---SI-K--L------IE----
	Plasmodium yoelii	CDU19838	-VH-NKNN-------LG-----T-I-		-NSN---NIGK--L------IE----
	Plasmodium yoelii 17X	ETB56137	-VH-NKNN-------LG-----T-I-		-NSN---NIGK--L------IE----
	Plasmodium yoelii yoe. 17XNL	XP_727636	-VH-NKNN-------LG-----T-I-		-NSN---NIGK--L------IE----
**Other Apicomplexa**	*Babesia microti str. RI*	829084949	-FF---G-Y-LVT--Y-G-E-FDEIS		ARK-F--FD-AV-IK-VLNGIN-M--
	Theileria parva str. Mug.	71033905	-FF--E--Y-FVT-LYTG-E-FDEIV		SRK-L--YD-A--IK-IL-GIT-M--
	Besnoitia besnoiti	1261478530	-II-TQQHL-L-M--A-G-E-YD-IV		NRQ-VE-ME-CKF-R-IL-G-EQI-V
	Cyclospora cayetanensis	1249162920	-FF--KG-F-LVT-VYTG-E-FDEII		SRK-FS-VD-A--IR-VL-GIT-M--
	Cystoisospora suis	1268239803	-LIDTPTD-F-VM---QG-E-FDHIV		Q-S-LP-YE---F-Q-IV-G---C--
	Eimeria acervulina	915004205	-FF--KG-F-LVT-VYTG-E-FDEII		SRK-FS-VD-A--IR-VL-GIT-M--
	Eimeria bovis	228205206	-FF--KG-F-LVT-VYTG-E-FDEII		SRK-FS-VD-A--IR-VL-GIT-M-R
	Eimeria maxima	1279423	-FF--KG-F-LVT-VYTG-E-FDEII		NRK-FS-AD-A--VR-VL-GIN-M-R
	Eimeria necatrix	921116306	-FF--KG-F-LVT-VYTG-E-FDEII		SRK-FS-VD-A--IR-VL-GIT-M--
	Eimeria praecox	557154722	-LVDTPTD-FV-M---AG-E-FDHII		Q-A-LS-SE---F-Q-IV---EHC-Q
	Eimeria tenella	1279425	-FF--KG-F-LVT-VYTG-E-FDEII		SRK-FS-VD-A--IR-VL-GIT-M--
	Hammondia hammondi	675121684	-LIDTPTD-F-VM---QG-E-FDHIV		Q-S-LP-HE---F-Q-IV-G---C-R
	Neospora caninum Liv.	401396111	-I---E-QLAIV---C-G---FSHL-		ARG-FP-Q---ALVH-MLTVLY---S
	Toxoplasma gondii ARI	1005149484	-I---E-QLALV---CPG---FSHL-		DRG-FP-Q---V-VQ-MLAVLC---A
	Cryptosporidium andersoni	1098428951	-VIDTPSD-F-VM--ING-E-FD-IV		Q-G-L--NES--L-Q----GIE-CYI
	Cryptosporidium hominis TU502	67624447	-TF--NTD--LVM-LCTG-E-FER-V		H--VFR-SD-A--MKDVL---A-C--
	Cryptosporidium muris RN66	209877312	-VIDTPSD-F-VM--ING-E-FD-IV		Q-G-L--NES--L-Q----GIE-CYI
	Cryptosporidium parvum Iowa II	126649261	-VIDTXSD--I-M---TG-E-FD-II		Q-G--S--ES--L-Q-I--GME-CYI
	Cryptosporidium ubiquitum	1098425940	-TF--NAD--LVM-LCTG-E-FER-V		H--VFR-SD-A--MKDVL---A-C--
**Other Eukarya**	*Acanthamoeba castellanii str.*	XP_004338484	DVI-SEDR-NIVM--SEG-E--T-IT		-QGKL--NE---L-L--L---Q-C-S
	Colletotrichum higginsianum	CCF45512	DIW-N-SE--LV----EQ---FD-IN		NNG-FI-EG-MFL-R-MM--LQ-C-S
	Sporothrix schenckii ATCC 58.	ERT00074	DIW-N-NE--L-----E----FT--S		QNGAMT-EVSIFV-R-MM---Q-C-A
	Ophiostoma piceae UAMH 11346	EPE08447	DIW-N-NE--L-----E----FT--S		QNGALT-EASVFV-R-MM---Q-C-A
	Colletotrichum orbiculare MA.	ENH87002	DIW-N-SE--LV----EQ---FD-IN		TNG-FS-ES-MF--R-MM--LQ-C-S
	Colletotrichum salicis	KXH69206	DIW-N-SE--LV----EQ---FD-IN		TNG-FT-EG-MFL-R-MM--LQ-C-S
	Acremonium chrysogenum ATCC 1.	KFH46516	DIW-N-SE--L----IDQ---FTFIN		SRG-LT-EV-LYF-R-M---IT-C-S
	Trichechus manatus lat.	XP_004382547	QVM-TKNML-LVT--AK--EIFD-LA		NHG-L--SE---K-W-IL-----C-G
	Camelus ferus	XP_014419538	QVM-TKNML-LVT--AK--EIFD-LA		NHG-L--SE---K-W-IL-----C-S
	Tursiops truncatus	XP_019797468	QVM-TKSML-LVT--AK--EIFD-LA		NHG-L--SE---K-W-IL-----C-G
	Ursus maritimus	XP_008688436	QVM-TKNML-LVT--AK--EIFD-LA		NHG-L--SE---K-W-IL-----C-G
	Vicugna pacos	XP_015106338	QVM-TKNML-LVT--AK--EIFD-LA		NHG-L--SE---K-W-IL-----C-S

Figure S66. A partial sequence alignment of a Serine/threonine protein kinase showing a one amino acid insertion that is specific for members of the *"Plasmodium"* subgenus (sans *P. ovale* and *P. malariae*) with exceptions found within *P. gaboni*, *Plasmodium sp. DRC-Itaito* and *Plasmodium sp. Gorilla Clade G2*. (not shown).

			28		68
	Plasmodium berghei ANKA	68069783	STQLYCKRNMKMFIENGELKND	K	NMIIVSCNSQTNGIGTRD
	Plasmodium chabaudi ada.	1061790962	-----------L--Q------E	E	------------------
	Plasmodium chabaudi cha.	1061786145	-----------L--Q------E	E	------------------
	Plasmodium coatneyi	1139860911	----------ARLLQ--I-QDE	-	-I-V-T--E---------
	Plasmodium fragile	817746710	----------ARLLQ--I-QD-	-	--VL-T--E---------
	Plasmodium gonderi	1194444316	------R----Q-MQ--D-RD-	-	---V----D---------
	Plasmodium inui San Ant. 1	672197907	----------ARLLQ--T-QD-	-	SIVV-T--E---------
**"*Vinckeia-Plasmodium*" clade (12/13)**	*Plasmodium knowlesi str. H*	221054079	----------ARLLQ--I-QDE	E	--VV-T--E---------
	Plasmodium malariae	1037133606	-----SR-HL-E--DDAK--D-	-	-I----------------
	Plasmodium ovale curt.	1036549794	------R----T-LQSEK--D-	D	S-----------------
	Plasmodium ovale wal.	1037150998	------R----T-LQSKK--D-	D	S-----------------
	Plasmodium vinckei pet.	577147218	-----------L--Q------E	E	------------------
	Plasmodium vinckei vin.	669201425	-----------L--Q------E	E	------------------
	Plasmodium vivax Bra. I	901873339	----------ARLLQ--S-QD-	-	H-VV-T--E---------
	Plasmodium yoelii	82704655	-----------T----------	E	------------------
	Plasmodium falciparum 3D7	258549083	---Q--I----K---DNK-RD-		-------DK-YH------
	Plasmodium falciparum FCH/4	574964318	----FS---K-F--KS-K--EN		-T--I---I---------
	Plasmodium falciparum RAJ116	906523206	-P-Q--I----K---DNK-RD-		-------DK-YH------
	Plasmodium falciparum UGT5.1	583211778	----FS---K-F--KS-K--EN		----I---I---------
**Other *Plasmodium* (0/7)**	*Plasmodium gaboni*	1370972970	---Q--R----E---ENK-RD-		--------K-Y-------
	Plasmodium reichenowi	1145259908	---Q--R---NR---ENK-RD-		--------K-NH------
	Plasmodium sp. DRC-Itaito	1370977934	---Q--R----E---ENK-RD-		--------K-Y-------
	Plasmodium sp. Gor. Cla. G	1370962886	---Q--I----K---DNK-RD-		-------DK-YH------
	Plasmodium gallinaceum	1103663165	----------QL---T-K--D-		------------------
	Plasmodium relictum	1102622983	----------QL---T-K--E-		------------------
	Besnoitia besnoiti	1261478615	---KW-L--LGDLCATHG-SPS		AWVA--ASR--AAV----
	Cystoisospora suis	1268232116	---KW-L-SLQTLCDEHG-SPS		LWVA--ATQ--AAV---A
**Other Apicomplexa**	*Hammondia hammondi*	675134520	---KW-L--LETLCSVHG-SPS		TWVA--ASR--AAV----
	Neospora caninum Liv.	401402057	---KW-L--LENLRSVHG-SPS		TWVA--ASY--A-V----
	Toxoplasma gondii ME49	1085066106	---KW-L--LETLCSVHG-SPS		TWVA--ASR--AAV----

Figure S67. A partial alignment of the Biotin-acetyl-CoA-carboxylase ligase protein, showing a one amino acid insertion that is exclusive for the *"Vinckeia-Plasmodium"* clade. Expanded alignment of Figure 6 (A). CSI region is not conserved in *P. cynomolgi*.

Group	Species	Accession	347		394
"Vinckeia-Plasmodium" clade (13/13)	*Plasmodium vivax Ind. VII*	KMZ81663	ELLKKAQVTKKLYNQMTQNSYKV	V	PTTREEVSLAELKFAIDSELLVKA
	Plasmodium vinckei vin.	XP_008625616	----R--I-N--W-DLIK----I	A	YI-K--I-M----KT--NA-FI--
	Plasmodium vinckei pet.	EUD69834	----R--I-N--W-DLIK----I	A	YI-K--I-M----KT--NA-FI--
	Plasmodium knowlesi str. H	XP_002261687	----------NI-----------	-	---K----------T------T--
	Plasmodium inui San Ant. 1	XP_008818475	----------------------L	-	---K--------R--------L--
	Plasmodium gonderi	GAW79874	----------N--KE-I-----I	T	S-----L--S---Y------F---
	Plasmodium fragile	XP_012337203	-----------I----I------	-	---K----------T------L--
	Plasmodium cynomolgi str. B	XP_004221330	D---------------I------	-	---K---------LT------I--
	Plasmodium coatneyi	XP_019913820	-----------I----------I	-	---K----------T------L--
	Plasmodium yoelii yoe.	EAA21407	D---R----N--W-DLIK----M	A	YI-K--I-M----KT--N--FI--
	Plasmodium yoelii 17X	ETB60826	----R----N--W-DLIK----M	A	YI-K--I-M----KT--N--FI--
	Plasmodium chabaudi cha.	SCL99835	----R--I-N--W-DLIK----I	A	YI-K--I-M----KT--NA-FI--
	Plasmodium chabaudi ada.	SCM02317	----R--I-N--W-DLIK----I	A	YI-K--I-M----KT--NA-FI--
	Plasmodium berghei ANKA	XP_022712234	----R--I-N--W-DLIK----I	A	YI-K--I-M----KT--N--FI--
	Plasmodium malariae	SBT70170	D------I--N-WTA-IK-T--I	T	---K-Q--MN---YT--K--FI--
	Plasmodium ovale curt.	SBS81361	-------I----WKEILK-----	T	-K-K----MC---NT--K--FI--
	Plasmodium ovale wal.	SBT32609	-------I----WKEILK-----	T	-K-K----MC---N---K--FI--
Other *Plasmodium* (0/7)	*Plasmodium falciparum 3D7*	XP_001347452	--------N-R-W-E-IK-----		---K-QI-MN---YT--K--FI-S
	Plasmodium falciparum HB3	KOB60836	--------N-R-W-E-IK-----		---K-QI-MN---YT--K--FI-S
	Plasmodium falciparum Mal.	ETW49160	--------N-R-W-E-IK-----		---K-QI-MN---YT--K--FI-S
	Plasmodium gaboni	SOV14827	--------N-R-W-E-IK-----		---K-QI-MN---YT--K--FI--
	Plasmodium gallinaceum	CRG97183	-----C--N-N-WKE-IK----I		-R-K----MN---YT--R--FN--
	Plasmodium reichenowi	CDO64603	--------N-R-W-E-IK-----		---K-QI-MN---YT--K--FI-S
	Plasmodium relictum	CRG99031	Q----C--N-N-WKEIIK----I		-K------MN---YT--K--FI-S
	Plasmodium sp. DRC-Itaito	SPJ10583	--------N-R-W-E-IK-----		---K-QI-MN---YT--K--FI-S
	Plasmodium sp. Gor. Cla. G	SOS78823	--------N-R-W-E-IK-----		---K-QI-MN---YT--K--FI-S

Figure S68. Alignment of the Hypothetical protein PVIIG_05030, showing a one amino acid insertion that is exclusive to the *"Vinckeia-Plasmodium"* clade.

Group	Species	Accession	227		276
"*Vinckeia-Plasmodium*" clade (13/13)	*Plasmodium berghei ANKA*	XP_679973	NSTSFKNLLKEEKKYKKLIGEATQI		NEFFSNYKKTIPYGINIIIGIFLTF
	Plasmodium chabaudi ada.	SCM07444	--A---------------V--T---		------------------V------
	Plasmodium chabaudi cha.	XP_744494	--A---------------V--T---		------------------V------
	Plasmodium yoelii	XP_022813314	--A------------------T---		-------------------------
	Plasmodium vinckei pet.	EUD73834	--A---------------V--T---		------------------V------
	Plasmodium vinckei vin.	XP_008623911	--P---------------V--T---		------------------V------
	Plasmodium coatneyi	XP_019917176	--K----------A-N----KTNNL		-D--------Y-----L-L------
	Plasmodium cynomolgi str. B	XP_004224400	--K----------A-N----KTNN-		-D--------Y-----L-L------
	Plasmodium fragile	XP_012333544	--K----------A-NR---KTNN-		-D--------Y-----L-L------
	Plasmodium gonderi	GAW82977	--K----------A----V-NTNN-		-D--------Y-----L--------
	Plasmodium inui San Ant. 1	XP_008817422	--N----------A-N----KTNN-		-D--------Y-----L-L------
	Plasmodium knowlesi str. H	XP_002260766	--K----------A-NR---KTNN-		-D--------Y-----L-L-L----
	Plasmodium vivax Bra. I	KMZ83779	--KA---------A------KTNHL		-D--------C-----L-L------
	Plasmodium vivax Ind. VII	KMZ78591	--KA---------A------KTNHL		-D--------C-----L-L------
	Plasmodium vivax Mau. I	KMZ90615	--KA---------A------KTNHL		-D--------C-----L-L------
	Plasmodium vivax North Kor.	KMZ97521	--KA---------A------KTNHL		-D--------C-----L-L------
	Plasmodium malariae	SCP02697	--K---D--N---A--R-M-NTNN-		-D--------Y---L-L--------
	Plasmodium ovale curt.	SBS99446	--K-------------R---QTGN-		-D--T-----Y-----L-L------
	Plasmodium ovale wal.	SBT45331	--K-----Q-------R---QTGN-		-D--T-----Y-----L-L------
Other *Plasmodium* (0/7)	*Plasmodium falciparum 3D7*	XP_001348411	--KT-----R--RE--RIV-DTNNQ	T	-D-------SY-----L--------
	Plasmodium falciparum Dd2	KOB87739	H-KT-----R--RE--RIV-DTNNQ	T	-D-------SY-----L--------
	Plasmodium falciparum Pal. Alt.	ETW53888	--KT-----R--RE--RIV-DTNNQ	T	-D-------SY-----L--------
	Plasmodium falciparum Tan.	ETW34045	--KT-----R--RE--RIV-DTNNQ	T	-D-------SY-----L--------
	Plasmodium gaboni	XP_018639320	--KT-----R--RE--RIV-NTNNQ	A	-D-------SY-----L--------
	Plasmodium reichenowi	SOV82811	--KT-----R--RE--RIV-DTNNQ	T	-D-------SY-----L--------
	Plasmodium sp. DRC-Itaito	SOV24980	--KT-----R--RE--RIV-NTNNQ	A	-D-------SY-----L--------
	Plasmodium sp. Gor. Cla. G	SOV82678	--KT-----R--RE--RIV-DINNQ	T	-D-------SY-----L--------
	Plasmodium relictum	CRH03897	--K---------R----IM-NKNNG	L	-D--F----AF-----L-L-L----
	Plasmodium gallinaceum	CRG95759	--R---------RE---IMKKENNK	I	-D--F-----F-----L-M-L----

Figure S69. Alignment of a Conserved Plasmodium protein, showing a one amino acid deletion that is exclusive to the *"Vinckeia-Plasmodium"* clade.

Clade	Species	Accession	688				737
"*Plasmodium-Malariae*" clade (8/8)	*Plasmodium vivax North Kor.*	KMZ97769	IIKNMKKYMKEGSNFSVYIDHM	P	PPEVYQ	NN	CKKFNTIKWALILFFIPAW
	Plasmodium vivax Mau. I	KMZ91745	----------------------	-	------	--	-------------------
	Plasmodium vivax Ind. VII	KMZ78899	----------------------	-	------	--	-------------------
	Plasmodium vivax Bra. I	KMZ87107	----------------------	-	------	--	-------------------
	Plasmodium knowlesi str. H	XP_002260411	------------N---------	-	SS---H	-D	-D-----------------
	Plasmodium inui San Ant. 1	XP_008814165	--E------E--N-------DV	-	--K--D	KG	-N-----------------
	Plasmodium gonderi	GAW82624	L-N--N-E--G-N-----ME-R	T	-----N	Y-	-----------M-------
	Plasmodium fragile	XP_012337456	------R-----N---L----V	-	-----H	KD	-N-----------------
	Plasmodium cynomolgi str. B	XP_004224070	-----Q------N--------V	-	--Q-NN	-G	-N-----------------
	Plasmodium coatneyi	XP_019916810	------------N---------	-	-----H	-D	-N-----------------
	Plasmodium malariae	SCO94110	LLN-RR--V-D-NT-TI---E-	S	TNNI-K	E-	-R-----------------
Other "*Vinckeia-Plasmodium*" clade (0/5)	*Plasmodium ovale*	SCP05819	L-N---Q-I-D-G-Y-I---KI		T-DI-S	I-	---------V----L--S-
	Plasmodium berghei ANKA	XP_678492	V-E---ARLNYSID-PL-LNKI		Y-DI-N	TD	-NI--KL--V-SI-L--S-
	Plasmodium chabaudi ada.	SCM09373	V-E---ARFNYTID-PL-LNKI		Y-DI-N	TD	-NI--RL--V-SI-L--S-
	Plasmodium chabaudi cha.	XP_741775	V-E---ARFNYTID-PL-LNKI		Y-DI-N	TD	-NI--RL--V-SI-L--S-
	Plasmodium vinckei pet.	EUD71335	V-E---TRLNYTID-PL-LNKI		Y-DI-N	TD	-NI--RL--V-SI-L--S-
	Plasmodium vinckei vin.	XP_008622015	V-E---ARSNYTID-PL-LNKI		Y-DI-N	SD	-NI--RL--V-SI-L--S-
	Plasmodium yoelii 17X	ETB56247	--E---THLNYSID-PL-LNKI		Y-DI-N	TD	-NI--KL--V-SI-L--S-
Other *Plasmodium* (0/7)	*Plasmodium falciparum 3D7*	XP_001348614	L-N-----I---KS----V-QL		R-DIFN		-N---K-------------
	Plasmodium falciparum 7G8	EUR62912	L-N-----I---KS----V-QL		R-DIFN		-N---K-------------
	Plasmodium falciparum Dd2	KOB86298	L-N-----I---KS----V-QL		R-DIFN		-N---K-------------
	Plasmodium falciparum FCH/4	ETW29102	L-N-----I---KS----V-QL		R-DIFN		-N---K-------------
	Plasmodium falciparum HB3	KOB62310	L-N-----I---KS----V-QL		R-DIFN		-N---K-------------
	Plasmodium falciparum IGH-CR14	KNG77339	L-N-----I---KS----V-QL		R-DIFN		-N---K-------------
	Plasmodium falciparum Mal.	ETW46652	L-N-----I---KS----V-QL		R-DIFN		-N---K-------------
	Plasmodium falciparum NF135/5.	ETW39804	L-N-----I---KS----V-QL		R-DIFN		-N---K-------------
	Plasmodium falciparum Pal. Alt.	ETW54117	L-N-----I---KS----V-QL		R-DIFN		-N---K-------------
	Plasmodium falciparum San. Lu.	EUT78975	L-N-----I---KS----V-QL		R-DIFN		-N---K-------------
	Plasmodium falciparum Tan.	ETW33809	L-N-----I---KS----V-QL		R-DIFN		-N---K-------------
	Plasmodium falciparum UGT5.1	EWC73762	L-N-----I---KS----V-QL		R-DIFN		-N---K-------------
	Plasmodium falciparum Vie.	ETW15983	L-N-----I---KS----V-QL		R-DIFN		-N---K-------------
	Plasmodium gaboni	XP_018639521	L-N-----I---KS----V-QL		R-DIFN		-N--KK-------------
	Plasmodium gallinaceum	CRG94658	L-R-----L---IKI-I-----		S--I-N		-N-YKK------I------
	Plasmodium reichenowi	SOV83027	L-N-----I---KS----V-QL		R-DIFN		-N---K-------------
	Plasmodium relictum	CRH01463	--R-----L-D-IK--I---Q-		A--I-N		-N-YKK------I------
	Plasmodium sp. DRC-Itaito	SOV25183	L-N-----I---KS----V-QL		R-DIFN		-N--KK-------------
	Plasmodium sp. Gor. Cla. G	SOV83324	L-N-----I---RT----V-QL		R-DIFN		-N---K-------------

Figure S70. Alignment of a Conserved Plasmodium protein, showing a one amino acid insertion that is exclusive to the *"Plasmodium-Malariae"* clade. A two amino acid insertion is also present that is exclusive to the "*Vinckeia-Plasmodium"* clade.

Group	Species	ID	153		204
**"*Plasmodium-Malariae*" clade (7/8)**	*Plasmodium vivax Mau. I*	901876239	FDVIVCCDVSHSLLVKYSKMVRSIS	PV	KKIAFLCCNIYGLCGYLFVDFGKGF
	Plasmodium vivax Bra. I	901868132	-------------------------	--	-------------------------
	Plasmodium vivax Ind. VII	901858533	-------------------------	--	-------------------------
	Plasmodium vivax North Kor.	901885266	-------------------------	--	-------------------------
	Plasmodium cynomolgi str. B	457877603	---V--------H------------	-A	-------------------------
	Plasmodium inui San Ant. 1	672187706	---V-------T-------------	-A	-------------------------
	Plasmodium coatneyi	1139869084	---V----L--TH---------N--	LT	-------------------------
	Plasmodium fragile	817740807	---V-------TE------------	S-	---------------C------N--
	Plasmodium gonderi	1194440806	---V----M-NTDI--VNNL---VE	EP	----------------I----N---
	Plasmodium malariae	1070256161	--IV----INNENIL--NN-I---E	NK	----------------I----N-N-
**Other *Plasmodium* (0/12)**	*Plasmodium ovale wal.*	1037146568	---V------NDVIL--N-L--N-Q	T	----------------I----NTN-
	Plasmodium ovale curt.	1037171074	---V-----NNDAI---NNL--N-Q	T	----------------I----NTN-
	Plasmodium vinckei pet.	577151446	---V----TKD-DII--NNLI---E	T	-N---IS--V------I-N--N-N-
	Plasmodium chabaudi cha.	1027959752	---V----TKD-DII--NNL--G-E	T	-N----S--V------M-N---SN-
	Plasmodium chabaudi ada.	1068927661	---V----TKD-DII--NNL--G-E	T	-N----S--V------M-N---SN-
	Plasmodium vinckei vin.	669195630	---V----TKD-DII--NNL----E	T	-N----S--V------I-N--N-N-
	Plasmodium yoelii 17X	564280364	---V----AKD-DII--NNL----E	N	-N----S---------I-N---NN-
	Plasmodium yoelii yoe.	23488286	---V----AKD-DII--NNL----E	N	-N----S---------I-N---NN-
	Plasmodium berghei ANKA	1269290753	---V----TKD-DII--NNLI---E	T	-N----S--V------I-N---NN-
	Plasmodium gallinaceum	1103666945	---V-A--INNNDII--NNL----K	T	----------------I----NNE-
	Plasmodium relictum	1102626529	---V-A--INNEDII--NNL----K	T	----------------I----NNE-
	Plasmodium sp. Gor. Cla. G2	1370983594	---VI---INKED-I--NN-I-G-D	K	------S---------I----N-E-
	Plasmodium falciparum Vie.	574749408	---VI---INKED-I--NN-I-G-D	K	-R----S---------I----N-E-
	Plasmodium falciparum 3D7	124806199	---VI---INKED-I--NN-I-G-D	K	-R----S---------I----N-E-
	Plasmodium falciparum Mal.	574986793	---VI---INKED-I--NN-I-G-D	K	-R----S---------I----N-E-
	Plasmodium falciparum RAJ116	906525105	---VI---INKED-I--NN-I-G-D	K	-R----S---------I----N-E-
	Plasmodium falciparum Dd2	914579694	---VI---INKED-I--NN-I-G-D	K	-R----S---------I----N-E-
	Plasmodium falciparum Tan.	574973180	---VI---INKED-I--NN-I-G-D	K	-R----S---------I----N-E-
	Plasmodium falciparum 7G8	579119326	---VI---INKED-I--NN-I-G-D	K	-R----S---------I----N-E-
	Plasmodium falciparum CAMP.	574999809	---VI---INKED-I--NN-I-G-D	K	-R----S---------I----N-E-
	Plasmodium falciparum Pal. Alt.	574994879	---VI---INKED-I--NN-I-G-D	K	-R----S---------I----N-E-
	Plasmodium falciparum FCH/4	574966855	---VI---INKED-I--NN-I-G-D	K	-R----S---------I----N-E-
	Plasmodium sp. DRC-Itaito	1370978736	---VI---INKED-I--NN-I---D	K	------S---------I----N-D-
	Plasmodium gaboni	1370973765	---VI---INKED-I--NN-I---D	K	------S---------I----N-D-
	Plasmodium reichenowi	641580202	---VI---INKQD-I--NN-I-G-D	K	-R----S---------I----N-E-
**Other Apicomplexa**	*Toxoplasma gondii ME49*	1085069265	---VIVTEAGNEE-K-TNAFC--A-		-PVG-IAA-VF--AASV---L-ER-
	Cystoisospora suis	1268243662	---VIMTEA-NAE-KRFNEFC-QQQ		PR-G-ISACVF--ASSI-----DQ-
	Hammondia hammondi	675134598	---VIVTEAGNEE-K--NAFC--A-		-PVG-VAA-VF--AASV-----ER-
	Neospora caninum Liv.	401402305	---VIVTEAGNEE-KL-NRFC-AAP		RPVG-VAT-VF--AASI-----ER-
	Cryptosporidium parvum Iowa II	66357282	H-----A--PL--QI--NELC-DHT		PN-G-ISA-SL----SI-----DS-
	Cryptosporidium hominis TU502	67623861	H-----A--PL--QI--NELC-DHT		PN-G-ISA-SL----SV-----DS-
	Cryptosporidium meleagridis	1338323102	H-----A--PL-QQI--NELC-DHT		PN-G-ISA-SL----SI-----NS-
	Cryptosporidium andersoni	1098429810	-IV--S-IPLREQLL-NSYC-LRT		PS-K-ISA-SL----SI-----PE-
	Cryptosporidium muris RN66	209877771	--V--S-IPLRQQLL-NSYC-LRT		PSVK-ISA-SL----SI-----PE-
	Cryptosporidium ubiquitum	1098423783	H-L---A--PL-QQI--NEVC-GHT		PN-G-ISA-CL----SI-----DS-

Figure S71. Alignment of the Ubiquitin-activating enzyme E1 protein, showing a two amino acid insertion that is exclusive to the "*Plasmodium-Malariae"* clade. Exceptions were seen in *Babesia* and *Theileria*, however the insert was not seen in other Apicomplexa. Other eukaryotes contained a 4 amino acid deletion in the same position, however they were excluded to allow a more accurate alignment to a more closely related outgroup (other Apicomplexa). Expanded alignment of Figure 6 (B). A 3 amino acid insert is present in P. *knowlesi* (not shown).

Group	Species	Accession	277		323
"***Plasmodium-Malariae***" **clade (8/8)**	*Plasmodium vivax North Kor.*	KNA00692	CTWVVLFLLCGAAAAVYFADMKS	H	GEEVPHFVIMYFLLYSVLMSLTM
	Plasmodium vivax Ind. VII	KMZ81689	-----------------------	-	-----------------------
	Plasmodium vivax Bra. I	KMZ87733	-----------------------	-	-----------------------
	Plasmodium knowlesi str. H	SBO23817	Y---I--FV-AM-VTM-LNE--I	Q	--DI---I---IV-------I--
	Plasmodium inui San Ant. 1	XP_008818806	Y---I--PVSVV------T----	-	RGD------I----------I--
	Plasmodium gonderi	GAW79897	YL-IF--SISSVSSLL--I-IRR	-	RNGI----V---V--N----I--
	Plasmodium fragile	XP_012336893	Y--A----VSV---SL--PH---	-	----S---T-----------M--
	Plasmodium cynomolgi str. B	XP_004221353	F-------V-VL--V---T--RN	-	--D-----LT----------I--
	Plasmodium coatneyi	XP_019913813	Y--A---FV-VM--TL--N----	-	--D----I------------I--
	Plasmodium malariae	SBT70685	Y--TI--FISSLSTVISLL-LRK	D	IGKI----VI---I-N----I-A
**Other *Plasmodium* (0/12)**	*Plasmodium ovale wal.*	SBT32681	YI-LF--SMS-VLTII-LL-LRK		EVPI-Q--FI-L-I------I-V
	Plasmodium berghei ANKA	XP_022712245	YI-MFI-SISTTLMFI-ISNLRK		IHDI-QYLLI-W-A-T----MSI
	Plasmodium chabaudi ada.	SCM02466	YI-M-I-S-ST-LMLI-VSNLRK		VHDI-QYLLI-W-A-T----ISI
	Plasmodium chabaudi cha.	XP_016655147	YI-M-I-S-ST-LMLI-VSNLRK		IHDI-QYLLI-W-A-T----ISI
	Plasmodium falciparum 3D7	XP_024329097	Y--ML--CISTILSII-YLEI-K		II---D---I---I-I----I-I
	Plasmodium falciparum 7G8	EUR71939	Y--ML--CISTILSII-YLEI-K		II---D---I---I-I----I-I
	Plasmodium falciparum IGH-CR14	KNG78488	Y--ML--CISTILSII-YLEI-K		II---D---I---I-I----I-I
	Plasmodium falciparum Mal.	ETW49058	Y--ML--CISTILSII-YLEI-K		II---D---I---I-I----I-I
	Plasmodium falciparum NF54	EWC85790	Y--ML--CISTILSII-YLEI-K		II---D---I---I-I----I-I
	Plasmodium falciparum Pal. Alt.	ETW56620	Y--ML--CISTILSII-YLEI-K		II---D---I---I-I----I-I
	Plasmodium falciparum San. Lu.	EUT85512	Y--ML--CISIILSII-YLEI-K		II---D---I---I-I----I-I
	Plasmodium falciparum Tan.	ETW36396	Y--ML--CISTILSII-YLEI-K		II---D---I---I-I----I-I
	Plasmodium falciparum Vie.	ETW18368	Y--ML--CISTILSII-YLEI-K		II---D---I---I-I----I-I
	Plasmodium gaboni	SOV14870	Y--MF--CISTIL-II-YL-I-K		II---D---I---I-I----I-I
	Plasmodium gallinaceum	CRG97208	YM-II--SISSVLSII--L-I-K		SIDI-----I---I-NI---I-L
	Plasmodium reichenowi	CDO64626	Y--ML--CISTILSII-YLEI-K		II---D---I---I-I----I-I
	Plasmodium relictum	CRG99056	YI-IT--SISSILSVI--L-INK		DIDI-----I---I-N----V-L
	Plasmodium sp. DRC-Itaito	SOV22919	Y--MF--CISTIL-II-YL-I-K		II---D---I---I-I----I-I
	Plasmodium sp. Gor. Cla. G	SOV77180	Y--ML--CISTILSII-YL-I-K		II---D---I---I-I----I-I
	Plasmodium vinckei pet.	EUD69857	YI-LFI-S-ST-LMFI-VSNLRK		VHDI-QYLLI-W-A-T----MSI
	Plasmodium vinckei vin.	XP_008625639	YI-LFI-S-ST-LMFI-VSNLRK		VHDI-QYLLI-W-A-T----MSI
	Plasmodium yoelii 17X	ETB60785	YI-IFI-SIST-LMFI-VSNLRK		IRDI-QYLLI-W-A-T----MSI

Figure S72. Alignment of a Hypothetical protein PVNG_01558, showing a one amino acid insertion that is exclusive to the "*Plasmodium-Malariae*" clade.

Clade	Species	ID	190		238
"*Vinckeia-Ovale*" clade (5/5)	*Plasmodium berghei ANKA*	1269288476	IQRWKMWLFGLMYYHYKLVNKSSR	E	RKKLKPFIYESNKLYDNYIISGLK
	Plasmodium chabaudi ada.	1061793446	------------------------	-	------------------------
	Plasmodium chabaudi cha.	1068921557	------------------------	-	------------------------
	Plasmodium vinckei pet.	577149484	------------------------	-	------------------------
	Plasmodium vinckei vin.	669196702	------------------------	-	------------------------
	Plasmodium yoelii 17X	564276761	------------------------	-	------------------------
	Plasmodium ovale curt.	1036544874	------------------------	-	------------------------
	Plasmodium ovale wal.	1037150332	--------------Q------G-E	K	K-IM------N-R-----------
Other *Plasmodium* (0/13)	*Plasmodium coatneyi*	1139862609	--------------Q--FI--G-G		K-RM-S--C-G-------------
	Plasmodium cynomolgi str. B	457870197	--------------Q--FI--G-G		K--M-S------------------
	Plasmodium falciparum 3D7	258597029	--------------Q------GNG		K--M-S-L----------------
	Plasmodium falciparum 7G8	579123252	--------------Q------GNG		K--M-S-L----------------
	Plasmodium falciparum FCH/4	574967623	--------------Q------GNG		K--M-S-L----------------
	Plasmodium falciparum HB3	914549232	--------------Q------GNG		K--M-S-L----------------
	Plasmodium falciparum IGH-CR14	910270711	--------------Q------GNG		K--M-S-L----------------
	Plasmodium falciparum NF135/5.	574980935	--------------Q------GNG		K--M-S-L----------------
	Plasmodium falciparum RAJ116	906525114	--------------Q------GNG		K--M-S-L----------------
	Plasmodium falciparum San. Lu.	579334912	--------------Q------GNG		K--M-S-L----------------
	Plasmodium falciparum Vie.	574750193	--------------Q------GNG		K--M-S-L----------------
	Plasmodium fragile	817744384	--------------Q--FI--G-G		K--M-S------------------
	Plasmodium gaboni	1084858816	--------------Q------GNG		K--M-S-L----------------
	Plasmodium gonderi	1194443604	--------------Q------G-G		K--M-------K------------
	Plasmodium inui San Ant. 1	672192942	--------------Q--FI--G-G		K--M-S--C---------------
	Plasmodium knowlesi str. H	1047805107	--------------Q--FI--G-G		K-RM-S--C-G-------------
	Plasmodium malariae	1037140543	--------------Q---C--G-G		K--MN-------------------
	Plasmodium reichenowi	1145259669	--------------Q------GNG		K--M-S-L----------------
	Plasmodium sp. DRC-Itaito	1370977801	--------------Q------GNG		K--M-S-L----------------
	Plasmodium sp. Gor. Cla. G	1370962748	--------------Q------GNG		K--M-S-L----------------
	Plasmodium vivax Bra. I	901872403	--------------Q--FI--G-G		K--M-S--C---------------
	Plasmodium vivax Ind. VII	901862590	--------------Q--FI--G-G		K--M-S--C---------------
	Plasmodium vivax Mau. I	901881473	--------------Q--FI--G-G		K--M-S--C---------------
"*Haemamoeba*" (0/2)	*Plasmodium gallinaceum*	1103666210	--------------Q------GTG		--M---F--N-------------
	Plasmodium relictum	1102623540	--------------Q---A--GAG		--M---F--N-------------

Figure S73. Alignment of the Phosphoinositide-specific phospholipase C protein, showing a one/two amino acid insertion that is exclusive to the "*Vinckeia-Ovale*" clade. Expanded alignment of Figure 6 (C).

Group	Species	ID	144		192
**“*Vinckeia-Ovale*” clade (5/5)**	*Plasmodium berghei ANKA*	1269289107	KKISENNINELLVEIVYTIFTKNE	T	LENNMLNLLGESNVDLIINIIKNK
	Plasmodium chabaudi ada.	1061793836	-R----------------------	-	---S---------I----------
	Plasmodium chabaudi cha.	1068921920	-R-----------I----------	-	---S---------I----------
	Plasmodium vinckei petteri	577148171	-R-----------I----------	-	---S---------I----K-----
	Plasmodium vinckei vin.	669193872	-------------I----------	-	---S---------I----------
	Plasmodium yoelii 17X	564274861	------------------------	-	---S--T-----------------
	Plasmodium yoelii yoe.	23478640	------------------------	-	---S--T-----------------
	Plasmodium ovale wal.	1037159769	---KI-DM----T--I-VL-KGE-	N	IQ-GL----S-KY-----R-----
	Plasmodium ovale curt.	1036546639	---QI-DM----T--I-VLLKDE-	N	IQ-GL----S-KY-----S--Q--
**Other *Plasmodium* (0/15)**	*Plasmodium vivax North Kor.*	901886233	----L-EM---IT--I--TM--DH		---SL-D----EHI-F-F------
	Plasmodium vivax Mau. I	901878312	----L-EM---IT--I--TM--DH		---SL-D----EHI-F-F------
	Plasmodium vivax Ind. VII	901859892	----L-EM---IT--I--TM--DH		---SL-D----EHI-F-F------
	Plasmodium vivax Bra. I	901872842	----L-EM---IT--I--TM--DH		---SL-D----EHI-F-F------
	Plasmodium cynomolgi str. B	457874933	----L-EM---IT--I--TM-RDN		---SL-D----EHI-F-F------
	Plasmodium coatneyi	1139867415	----L-EM---IT--I--TL-RDN		---SL-D----EHI-F-F------
	Plasmodium relictum	1102625386	---YI-EL----T--I--TL-EDD		---GL------KYL-F--------
	Plasmodium reichenowi	641578801	---YI-E-----I--I-ITL-TEN		---SLVD---DRFL-F--Q-----
	Plasmodium malariae	1070255122	-E-----T---HITL--DN		--SSL-D--N-NY--F--------
	Plasmodium knowlesi str. H	221059673	----L-EM---IT--I--TM-RDN		---SL-D----EHI-F-F------
	Plasmodium inui San Ant. 1	672186156	----L-EM----T--I--TM-RDN		---SL-D----EHI-F-F------
	Plasmodium gonderi	1194442240	----L-E----IT--I-ITMI-ED		---SL-D----EHI-F------C-
	Plasmodium gallinaceum	1103665292	LG---T--I-ITL--DD		I--GL-I----KYL-F--------
	Plasmodium gaboni	1370975043	---YI-E-----I--I-ITL-REN		---SLVD---DRFL-F--Q-----
	Plasmodium fragile	817741370	----L-EM---IT--I--TM-RDN		---SL-D----EHI-F-F------
	Plasmodium falciparum Vie.	574747799	---FI-E-----I--I-ITL-REN		---SLVD---DRFL-F--Q-----
	Plasmodium falciparum UGT5.1	583212046	---YI-E-----I--I-ITL-REN		---SLVD---DRFL-F--Q-----
	Plasmodium falciparum Tan.	574971639	---YI-E-----I--I-ITL-REN		---SLVD---DRFL-F--Q-----
	Plasmodium falciparum San. Lu.	579327957	---YI-E-----I--I-ITL-REN		---SLVD---DRFL-F--Q-----
	Plasmodium falciparum RAJ116	906522552	---YI-E-----I--I-ITL-REN		---SLVD---DRFL-F--Q-----
	Plasmodium falciparum Pal. Alt.	574992922	---YI-E-----I--I-ITL-REN		---SLVD---DRFL-F--Q-----
	Plasmodium falciparum NF54	583222497	---YI-E-----I--I-ITL-REN		---SLVD---DRFL-F--Q-----
	Plasmodium falciparum NF135/5.	574978037	---FI-E-----I--I-ITL-REN		---SLVD---DRFL-F--Q-----
	Plasmodium falciparum Mal.	574985348	---YI-E-----I--I-ITL-REN		---SLVD---DRFL-F--Q-----
	Plasmodium falciparum IGH-CR14	910272599	---FI-E-----I--I-ITL-REN		---SLVD---DRFL-F--Q-----
	Plasmodium falciparum HB3	914545233	---YI-E-----I--I-ITL-REN		---SLVD---DRFL-F--Q-----
	Plasmodium falciparum FCH/4	574965939	---YI-E-----I--I-ITL-REN		---SLVD---DRFL-F--Q-----
	Plasmodium falciparum CAMP.	574997774	---YI-E-----I--I-ITL-REN		---SLVD---DRFL-F--Q-----
	Plasmodium falciparum 7G8	579114109	---YI-E-----I--I-ITL-REN		---SLVD---DRFL-F--Q-----
	Plasmodium falciparum 3D7	124809317	---YI-E-----I--I-ITL-REN		---SLVD---DRFL-F--Q-----
	Plasmodium sp. Gor. Cla. G	1370984852	---YI-E-----N--I-ITL-IEN		---SLVD---DRFL-F--Q-----
	Plasmodium sp. DRC-Itaito	1370980041	---YI-E-----I--I-ITL-TEN		---SLVD---DRFL-F--Q-----

Figure S74. Alignment of a DEAD/DEAH helicase protein, showing a one amino acid insertion that is exclusive to the *“Vinckeia-Ovale”* clade.

Group	Species	Accession	764		811
"*Vinckeia-Ovale*" clade (5/5)	*Plasmodium berghei ANKA*	XP_022712410	QGNMSEGPIIVAAAKAGYNIFNN	SE	NNEENYLKKFKRLEDLEITFNSS
	Plasmodium chabaudi ada.	SCM19403	---------V-------------	--	-I-D-------------------
	Plasmodium chabaudi cha.	XP_731589	---------V-------------	--	-I-D-------------------
	Plasmodium vinckei pet.	EUD71100	---------V--------S----	--	-D-D-------------------
	Plasmodium vinckei vin.	XP_008625470	---------V--------S----	--	-A-N---T----------P----
	Plasmodium yoelii 17X	ETB58344	---------V------------D	--	-DS--------------------
	Plasmodium yoelii yoe.	EAA15769	---------V------------D	--	-DS--------------------
	Plasmodium ovale curt.	SBS97393	---------V--------SFVS-	PQ	EHHHAFYRDYT--DS--V-----
	Plasmodium ovale wal.	SBT39769	---------V--------SFVSH	PQ	EHHHAFYRDYT--DS--V-----
Other *Plasmodium* (0/15)	*Plasmodium coatneyi*	XP_019913082	H--------V-----V---FLT-		DSQQS-V-EYT--D---V-----
	Plasmodium cynomolgi str. B	XP_004224278	H--------V---------FITS		GSNHS-V-EYT--D---V-----
	Plasmodium falciparum 3D7	XP_024329152	H--------V-----V--SFI--		P-HKS--DN-Q--D---V-----
	Plasmodium falciparum 7G8	EUR69264	H--------V-----V--SFI--		P-HKS--DN-Q--D---V-----
	Plasmodium falciparum FCH/4	ETW30121	H--------V-----V--SFI--		P-HKS--DN-Q--D---V-----
	Plasmodium falciparum HB3	KOB61061	H--------V-----V--SFI--		P-HKS--DN-Q--D---V-----
	Plasmodium falciparum IGH-CR14	KNG76774	H--------V-----V--SFI--		P-HKS--DN-Q--D---V-----
	Plasmodium falciparum Mal.	ETW48289	H--------V-----V--SFI--		P-HKS--DN-Q--D---V-----
	Plasmodium falciparum NF135/5.	ETW41770	H--------V-----V--SFI--		P-HKS--DN-Q--D---V-----
	Plasmodium falciparum Tan.	ETW35530	H--------V-----V--SFI--		P-HKS--DN-Q--D---V-----
	Plasmodium falciparum Vie.	ETW17726	H--------V-----V--SFI--		P-HKS--DN-Q--D---V-----
	Plasmodium fragile	XP_012336786	H--------V-----V--SSLT-		E-HHS-V-QYT--D---V-----
	Plasmodium gaboni	XP_018638931	H--------V-----V--TFI--		P-HKS--DD----D---V-----
	Plasmodium gonderi	GAW82852	H--------V--------SFIT-		E-HNLCEMEYT--D---V-----
	Plasmodium inui San Ant. 1	XP_008816965	H--------V---------FIT-		DSHHSHI-EYI--D---V-----
	Plasmodium knowlesi str. H	XP_002260625	H--------V-----V--SFITD		DSHHSDV-EYT--D---V-----
	Plasmodium malariae	SBT72046	---------V-----T---FLG-		TSSNI---DYV--DN--V----C
	Plasmodium reichenowi	SOV80563	H--------V-----V--SFI--		P-HKS--DN-Q--D---V-----
	Plasmodium sp. DRC-Itaito	SOV23666	H--------V-----V--TFI--		P-HKS--DD----D---V-----
	Plasmodium sp. Gor. Cla. G	SOV79202	H--------V-----V--TFI--		P-HKS--DN----D---V-----
	Plasmodium vivax Bra. I	KMZ83652	H--------V--------SFVT-		GSHHS-V-EYT--D---V-----
	Plasmodium vivax Ind. VII	KMZ78465	H--------V--------SFVT-		GSHHS-V-EYT--D---V-----
	Plasmodium vivax Mau. I	KMZ90852	H--------V--------SFVT-		GSHHS-V-EYT--D---V-----
	Plasmodium vivax North Kor.	KMZ97635	H--------V--------SFVT-		GSHHS-V-EYT--D---V-----
	Plasmodium relictum	CRH03767	---------V-----V---FN--		TSNNS---EYI--DN--V-----
	Plasmodium gallinaceum	CRG95888	---------V-----V-FCFN-T		SNNS---EYV--DN--------

Figure S75. Alignment of an ATPase protein, showing a two amino acid insertion that is exclusive to the *"Vinckeia-Ovale"* clade.

Table S1. Some characteristics of the Hematozoa genomes used in Phylogenetic/Comparative Genomic Studies.

Group	Organism	Bioproject	Size (Mb)	GC%	Proteins	Level
Plasmodium	*Plasmodium berghei ANKA*	PRJNA317456	18.5607	22.29	4928	Chromosome
	Plasmodium chabaudi adami DK	PRJEB11993	19.3504	23.58	5378	Chromosome
	Plasmodium coatneyi Hackeri	PRJNA329102	27.6855	39.65	5516	Chromosome
	Plasmodium cynomolgi B	PRJDA49901	26.1813	40.59	5776	Chromosome
	Plasmodium falciparum 3D7	PRJNA430688	23.2703	19.36	5339	Chromosome
	Plasmodium fragile nilgiri	PRJNA282950	25.9145	42.10	5672	Scaffold
	Plasmodium gaboni SY75	PRJNA329100	20.3855	18.86	5354	Chromosome
	Plasmodium gallinaceum 8A	PRJEB9073	25.034	16.9994	5280	Scaffold
	Plasmodium gonderi ATCC30045	PRJDB5590	33.0063	26.90	5916	Scaffold
	Plasmodium inui San Antonio 1	PRJNA257224	27.405	42.40	5832	Scaffold
	Plasmodium knowlesi H	PRJEA28803	23.4622	39.17	5101	Chromosome
	Plasmodium malariae	PRJEB14392	33.618	24.39	5930	Chromosome
	Plasmodium ovale wallikeri	PRJEB12679	36.407	29.40	8646	Scaffold
	Plasmodium reichenowi CDC	PRJEB4434	24.0555	19.40	5630	Scaffold
	Plasmodium relictum SGS1	PRJEB9074	22.6074	18.3625	5137	Chromosome
	Plasmodium vinckei vinckei	PRJNA163123	18.2216	23.40	4954	Scaffold
	Plasmodium vivax Sal-1	PRJNA150	27.0137	42.28	5392	Chromosome
	Plasmodium yoelii yoelii 17XNL	PRJNA1436	23.1254	24.70	7861	Contig
	Plasmodium sp. Gorilla clade G2	PRJEB13584	22.1907	18.63	5321	Chromosome
	Plasmodium sp. DRC-Itaito	PRJEB13584	20.9342	18.63	5090	Chromosome
Piroplasmida	*Babesia bigemina Bond*	PRJEB5046	13.8409	50.61	5079	Chromosome
	Babesia bovis T2Bo	PRJNA20343	8.17971	41.6127	3706	Chromosome
	Babesia ovata Miyake	PRJDB5725	14.4534	49.3	5031	Contig
	Babesia microti RI	PRJEA72411	6.43448	36.18	3610	Chromosome
	Theileria annulata Ankara	PRJNA16308	8.35812	32.5492	3795	Chromosome
	Theileria equi WA	PRJNA38023	11.6745	39.47	5329	Chromosome
	Theileria orientalis Shintoku	PRJNA260532	9.01036	41.5803	4002	Complete Genome
	Theileria parva Muguga	PRJNA16136	8.34761	34.0411	4061	Chromosome

Table S2**.** *P. falciparum* protein sequences utilized to construct the tree shown in Figure 1 (A).

**Protein Name**	**Accession Number**
Gas41 homologue, putative	XP_002808824.1
Pre-mRNA splicing factor, putative	XP_001351366.1
DNA-directed RNA polymerase III subunit, putative	XP_001350475.1
Pre-mRNA splicing factor, putative	XP_001348243.1
DNA-directed RNA polymerase II second largest subunit	KNG78249.1
RNA-binding protein of pumilio/mpt5 family, putative	XP_001351478.1
Hypothetical protein PFFVO_03658	ETW17507.1
Nucleolar preribosomal assembly protein	XP_002808857.1
40S ribosomal protein S19, putative	XP_001351527.1
60S ribosomal protein L3, putative	XP_001347556.1
Eukaryotic translation initiation factor 5, putative	XP_001350476.1
tRNA delta(2)-isopentenylpyrophosphate transferase, putative	XP_001350485.1
Glycine-tRNA ligase	EWC86012.1
Bifunctional aminoacyl-tRNA synthetase, putative	XP_001350543.1

Table S3. *P. falciparum* protein sequences utilized to construct the tree shown in Figure 1 (B).

**Protein Name**	**Accession Number**
Flavoprotein subunit of succinate dehydrogenase	XP_001347618.1
Glycerol-3-phosphate acyltransferase	XP_001350533.1
Adenylosuccinate lyase	CZT98079.1
enolase	XP_001347440.1
Phosphomannomutase, putative	XP_001347454.2
Citrate synthase mitochondrial precursor, putative	XP_001347502.1
glucose-6-phosphate isomerase	XP_001348515.1
fructose-bisphosphate aldolase	XP_001348599.1
glyceraldehyde-3-phosphate dehydrogenase	XP_001348772.1
Adenylosuccinate synthetase	XP_001350257.1

Table S4. Summary of some characteristics of *Plasmodium* species featured in Figure 1.

Group	Taxonomic Subgenus	Member species	Pathogenicity Towards Humans	Host Specificity
"Laverania"	*Laverania*	*P. falciparum*	**Yes**	Humans and Great Apes (Hominidae)
		P. gaboni	No	
		P. reichenowi	No	
		P. sp. DRC-Itaito	No	
		P. sp. gorilla clade G2	No	
"Vinckeia"	*Vinckeia*	*P. berghei*	No	Rodents
		P. yoelii	No	
		P. chabaudi	No	
		P. vinckei	No	
"Plasmodium"	*Plasmodium*	*P. vivax*	**Yes**	Humans and "Old World Monkeys" (Cercopithecidae)
		P. gonderi	No	
		P. cynomolgi	No	
		P. inui	No	
		P. fragile	No	
		P. knowlesi	**Yes**	
		P. coatneyi	No	
"Vinckeia-Ovale" clade	*Plasmodium*	*P. ovale*	**Yes**	Humans
"Plasmodium-Malariae" clade	*Plasmodium*	*P. malariae*	**Yes**	Humans
"*Haemamoeba*"	*Haemamoeba*	*P. gallinaceum*	No	Avian species
		P. relictum	No	
